# One-Pot Synthesis and Photochemical Diversification of Pyrazolo[1,2‑*a*]pyridazinones into 3D-Rich Scaffolds

**DOI:** 10.1021/acs.joc.5c02313

**Published:** 2026-01-17

**Authors:** Ines Babnik, Nejc Petek, Uroš Grošelj, Jurij Svete, Bogdan Štefane

**Affiliations:** Faculty of Chemistry and Chemical Technology, 37663 University of Ljubljana, Večna pot 113, 1000 Ljubljana, Slovenia

## Abstract

We report a one-pot, three-component synthesis of pyrazolo­[1,2-*a*]­pyridazinones from tetrahydropyridazines, a transformation that was historically challenging due to competing ring contractions. The resulting compounds undergo photoinduced transformations, without the need for external photocatalysts, to afford diverse 3D-rich derivatives, including tricyclic cyclobutenes and γ-(pyrazol-1-yl)­butanals. Enabled by mild conditions, this strategy offers an efficient, atom-economical route to structurally diverse pyrazolo­[1,2-*a*]­pyridazinones and their derivatives.

## Graphical abstract

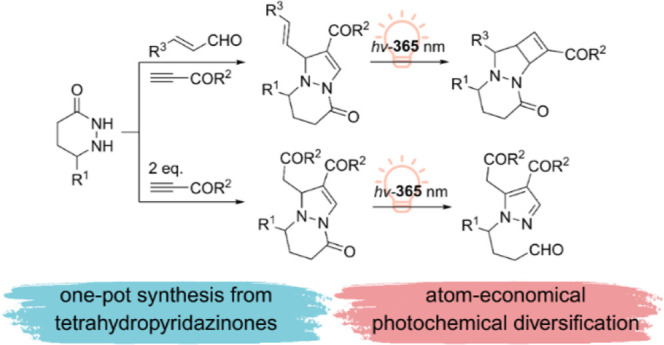

## Introduction

Pyrazoles have emerged as attractive scaffolds in pharmaceutical chemistry and agrochemical research due to their diverse biological activities.^1^ Among these, pyrazolopyridazines have attracted significant attention for their anticancer, analgesic, antihypoxic, antipyretic, antiinflammatory, antiviral, antifungal, and insecticidal properties, as well as their role as various enzyme inhibitors.^2–13^ Despite the potential of novel pyrazolopyridazine derivatives, their synthesis remains a challenge, with only a limited number of these frameworks being readily accessible. To date, most synthetic efforts have predominantly focused on the more accessible pyrazolo­[1,2-*b*]­phtalazines and pyrazolo­[3,4-*d*]­pyridazine-5,8-diones, which can be efficiently obtained via various multicomponent reactions.^2,3,5–7,9,11,13–18^

In contrast, [1,2-*a*]-bridged derivatives have been synthetically less explored. Most established procedures rely on five-membered pyrazolone-derived azomethine imines, which can be reacted with various diploarophiles in [3 + 2] cycloadditions to afford the desired scaffolds (representative example in Scheme 1A).^19–24^ Alternative synthetic pathways include organocatalyzed [3 + 4 + 5] multicomponent-reactions,^25^ reactions of azomethine ylides,^26,27^ [3 + 3] annulations to spirooxindoles,^28^ and transformations of 1,5-diazabicyclo[3.1.0]­hexanes.^29^

**Scheme 1:**
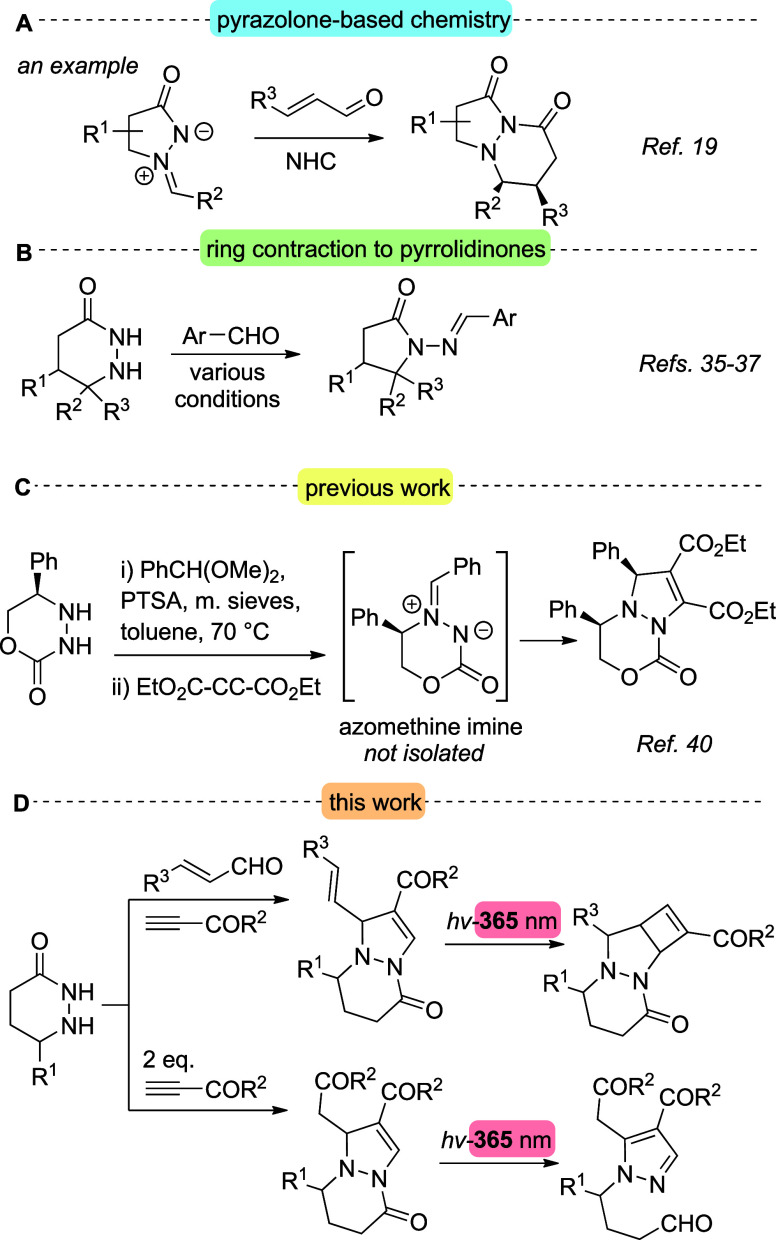
Ring Contractions of Tetrahydropyridazinones and Formations of Pyrazolo­[1,2-*a*]­pyridazinones

On the other hand, synthetic procedures employing tetrahyropyridazinones as starting materials are scarce. These compounds are known to be susceptible to ring-contraction to pyrrolidinones under various conditions, including treatment with LiHMDS,^30^ heating in acidic media,^31–33^ UV-irradiation,^34^ or condensation with aromatic aldehydes (Scheme 1B).^35–37^ The latter prevents the isolation of pyridazine-based azomethine imines, which would otherwise be suitable substrates for [3 + 2] cycloadditions leading to pyrazolo­[1,2-*a*]­pyridazinones. Although examples of successful synthesis from tetrahydropyridazinones or related carbazates have been reported, these methods remain much less explored (Scheme 1C).^38–42^

Partially saturated hetero­(bi)­cyclic frameworks serve as versatile 3D building blocks with broad applicability in catalysis, materials science, chemical biology, and drug discovery.^43–46^ Over the past decades, medicinal chemists have favored flat, sp^2^-rich molecules due to their straightforward preparation via established coupling reactions. However, such compounds often show limited shape diversity, poor solubility, and suboptimal interactions with three-dimensional biological targets.^47^ Increasingly, efforts are directed toward sp^3^-rich, 3D scaffolds that occupy distinct regions of chemical space, enhancing potency, selectivity, and pharmacokinetic profiles.^48–51^ Yet, their synthesis remains considerably more demanding, often requiring precise stereochemical control and transformations beyond simple cross-coupling chemistry.^52,53^

In the course of our studies on visible-light induced transformations of pyrazolo­[1,2-*a*]­pyrazolones leading to structurally diverse 3D-rich scaffolds, we became interested in extending similar strategies to pyridazine-containing systems.^54^ Inspired by existing reports utilizing tetrahydropyridazinones as precursors, we envisioned that their transformation via in situ formation of azomethine imines, followed by [3 + 2] cycloaddition with terminal ynones, could provide access to pyrazolo­[1,2-*a*]­pyridazines while bypassing issues related to ring contraction. We further hypothesized that these compounds could serve as valuable substrates for subsequent photochemical transformations, enabling access to diverse pyrazolo­[1,2-*a*]­pyridazine derivatives. In this work, we report a three-component approach to pyrazolo­[1,2-*a*]­pyridazines starting from tetrahydropyridazinones. We further demonstrate that these heterocycles can undergo UV-A induced transformations into structurally distinct 3D-rich derivatives without external photocatalysts, thereby expanding the accessible chemical space (Scheme 1D).

## Results and Discussion

Our study commenced with the synthesis of the model pyrazolo­[1,2-*a*]­pyridazinone **2a**. In the initial attempt, the reaction of tetrahydropyridazinone **1a** with cinnamaldehyde in the presence of trifluoroacetic acid (TFA) in dichloromethane (DCM) afforded a complex mixture of products, which impeded the subsequent [3 + 2] cycloaddition with methyl propiolate, resulting in no detectable formation of **2a**. Interestingly, when a one-pot procedure was employed – involving **1a**, cinnamaldehyde, methyl propiolate, copper, and TFA—the desired product **2a** was obtained, albeit in low 20% yield (Table 1, entry 2).

**Table 1:**
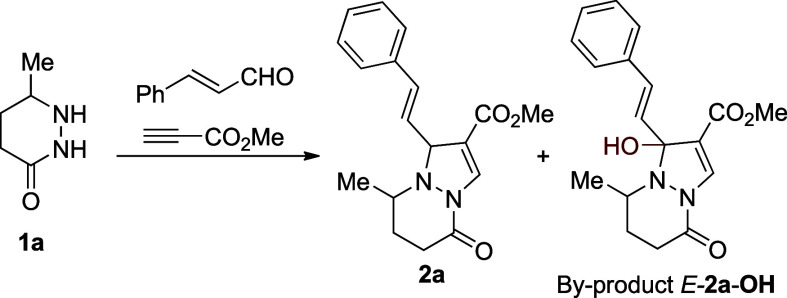
Optimization of the Reaction Conditions

entry	deviation from standard reaction conditions^a^	yield **2a** (*E*-2a-OH) [%]^b^
1	none	81 (n.d.)
2	TFA instead of MS, 25 °C	20 (n.d.)
3	TFA instead of MS, 50 °C	41 (n.d.)
4	25 °C instead of 50 °C	59 (n.d.)
5	Et_3_N instead of MS, 25 °C	n.d. (n.d.)
6	no Cu(0), 50 °C	75 (n.d.)
7	Zn(OTf)_2_ instead of Cu(0),^c^ 50 °C	22 (n.d.)
8	Zn(OTf)_2_ instead of Cu(0),^c^ 25 °C	21 (n.d.)
9	MeOH instead of DCM	78 (n.d.)
10	ACN instead of DCM	51 (n.d.)
11	THF instead of DCM	49 (n.d.)
12	air atmosphere (16 h)	73 (10)
13	air atmosphere (72 h)	n.d. (80)^d^
14	purged with oxygen	43 (n.d.)
15	purged with oxygen, no Cu(0)	65 (10)

a **1a** (0.2 mmol), cinnamaldehyde (0.3 mmol), methyl propiolate (0.22 mmol), Cu(0) (0.14 mmol), DCM (1.0 mL), MS 4 Å (30 mg), Ar, 50 °C, 16 h.

b NMR yields of **2a** (*E-* **2a**-**OH** in brackets).

c Zn­(OTf)_2_ (0.1 equiv).

d Byproduct *E-* **2a-OH** was isolated in 73% yield (80% NMR yield). n.d. = not detected.

To improve the reaction efficiency, we optimized the reaction conditions. We found that replacing TFA with molecular sieves (MS, 4 Å) to promote azomethine imine formation, along with heating the reaction mixture in DCM, significantly increased the yield (Table 1, entries 1–4). In contrast, replacing TFA with Et_3_N resulted in no formation of **2a** (Table 1, entry 5). Performing the reaction in the absence of Cu(0) led to a slightly lower yield, while substituting Cu(0) with a Lewis acid further diminished product **2a** formation, both at room temperature and upon heating (Table 1, entries 6–8). Changing the solvent from dichloromethane to methanol had little effect (Table 1, entry 9), whereas the use of acetonitrile or tetrahydrofuran substantially reduced the formation of **2a** (Table 1, entries 10 and 11).

When the reaction was carried out under air for a prolonged period, a byproduct, *E-* **2a**-**OH**, was isolated (Table 1, entry 13), likely resulting from oxidation at the allylic position (vide infra, Scheme 5). Moreover, purging the reaction vial with oxygen led to a complex product mixture with a reduced level of formation of **2a** and *E-* **2a**-**OH**, indicating that the reaction is oxygen sensitive (Table 1, entry 14). Interestingly, when copper was omitted and the reaction mixture was purged with oxygen, no additional side products were observed (Table 1, entry 15). This suggests that under oxygen, Cu(0) is likely oxidized and thereby promotes formation of side products, likely via radical intermediates.^55^

Based on these findings, we were able to significantly improve the reaction outcome, achieving an 81% yield for compound **2a** under the optimized conditions (DCM, MS 4 Å, 50 °C, Ar) (Table 1, entry 1).

With the optimized reaction conditions in hand, we extended the methodology to a series of substituted pyrazolo­[1,2-*a*]­pyridazinones (Scheme 2). The protocol tolerated various aromatic, heteroaromatic, and aliphatic substituents on α,β-unsaturated aldehydes, affording product **2** in moderate to good yields (Scheme 2). We anticipate that isolated yields may be lower than expected (e.g., **2a**: 69% isolated yield vs 81% NMR yield), likely due to partial oxidation during chromatographic purification. The reaction proceeded with high regioselectivity, furnishing only a single regioisomer in all cases. Most reactions gave two separable diastereoisomers except for the phenyl-substituted derivative **2i**, which was formed as a single isomer. The method was also compatible with polar functional groups, as demonstrated by the amide-substituted product **2g**/**2g′**. Various tetrahydropyridazinone scaffolds **1** were tolerated as well, although their scope was limited by the challenging synthesis of the starting materials **1** (see synthetic procedures in the Supporting Information). Notably, *trans*-2-hexen-1-al gave **2m/2m′**, but the major diastereoisomer **2m** rapidly oxidized and was isolated as *E*-**2m**-**OH**. Products **2** exhibit yellow fluorescence and a pronounced Stokes shift, averaging 190 nm (see Supporting Information).

**Scheme 2:**
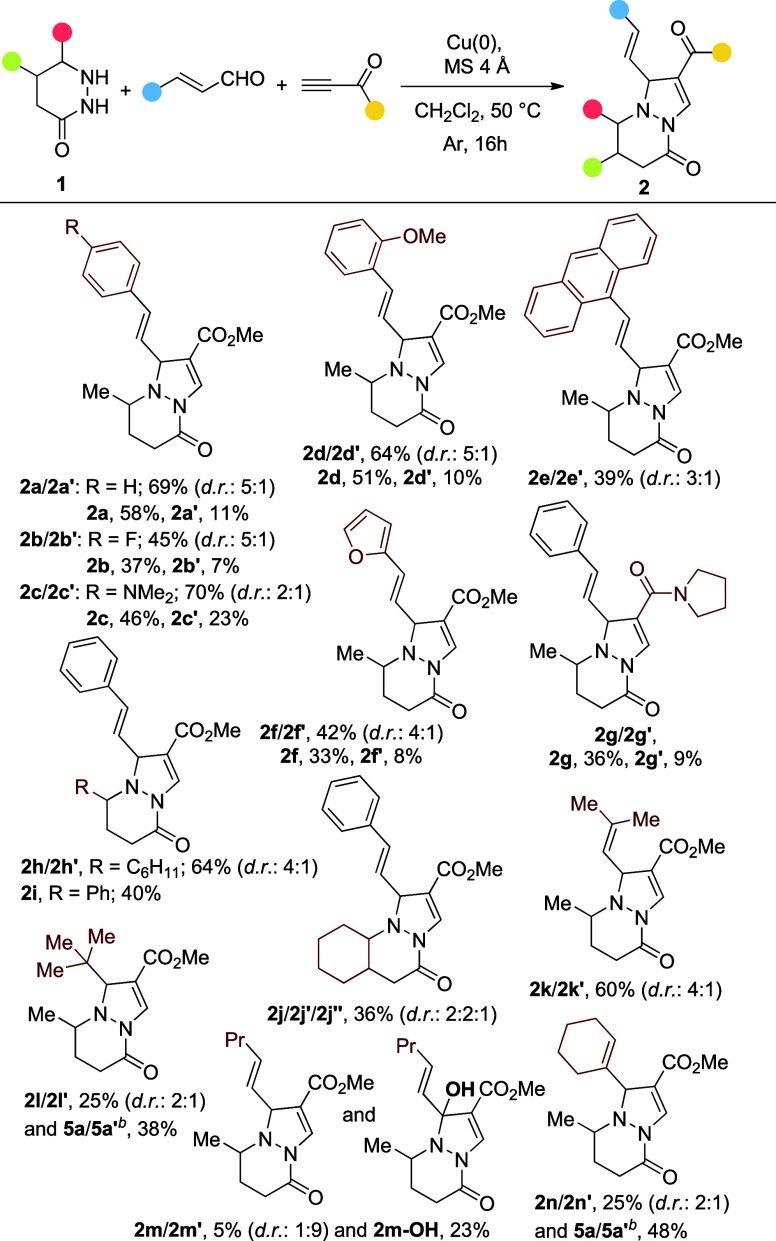
One-Pot Synthesis of Pyrazolo­[1,2-*a*]­pyridazinones[Fn s2fn1]

a
**1** (1.0 mmol), 3-(hetero)­aryl-enal (1.5 mmol), terminal ynone (1.1 mmol), DCM (5.0 mL), Cu(0) (0.7 mmol), MS 4 Å (100 mg), 50 °C, Ar, 16 h.
b For structure of **5a**/**5a′** see Scheme 6

In contrast, α,β-unsaturated aldehydes bearing a β-substituent (R ≠ H) failed to yield products **2** but instead led to compounds **5a**/**5a′** via double incorporation of methyl propiolate (vide infra, Scheme 6). Similarly, 1-cyclohexene-1-carboxaldehyde furnished **2n**/**2n′** in only 25% yield, with **5a**/**5a′** as the major product (48%, Scheme 2). Nonconjugated aldehydes behaved analogously, giving **5a**/**5a′** as dominant products, as seen with benzaldehyde (90% of **5a**/**5a′**, see Supporting Information) and pivaldehyde (25% of **2L**/**2L′** and 38% of **5a**/**5a′**). The less activated phenylacetylene also reacted in negligible amounts (see Supporting Information). This observation is consistent with literature reports^56,57^ indicating that such copper(0)-catalyzed reactions typically proceed via coppera acetylides, making electron-deficient alkynes markedly more reactive than nonactivated ones like phenylacetylene.

These examples prompted us to further investigate the double incorporation of methyl propiolate, leading to an optimized reaction to access product **5** (vide infra, Scheme 6).

Based on our previous work^54^ and considering that the obtained products **2** absorb light in the 300–500 nm range, we envisioned that irradiation of **2** could trigger their transformation into 3D-rich structures. Upon irradiation of **2a** with blue light (450 nm), both 1,2-diazepine **3a** and the tricyclic cyclobuta­[*c*]­pyrazolo­[1,2-*a*]­pyridazinone **4a** were formed (Scheme 3a). Time-course monitoring of the reaction at 365 and 450 nm, respectively, revealed that the ring expansion from **2a** to **3a** is outpaced by the subsequent 4π-electrocyclization of **3a** to **4a**, limiting the accumulation and high-yield isolation of **3a** (Scheme 3b). In contrast, irradiation of **2a** at 365 nm afforded the tricyclic product **4a** in a synthetically useful yield (85% NMR, 67% isolated; Scheme 4 and Supporting Information). Notably, irradiation at 450 nm also produced various byproducts, whereas irradiation at 365 nm proved more selective. With subsequent optimization studies, DCM was chosen as the optimal solvent, affording **4a** in the highest NMR yield (85%) compared to other solvents tested (see Supporting Information). The reaction tolerates the presence of water but is oxygen sensitive. Furthermore, on-and-off light-switching experiments showed that continuous light irradiation is essential for these transformations.

**Scheme 3:**
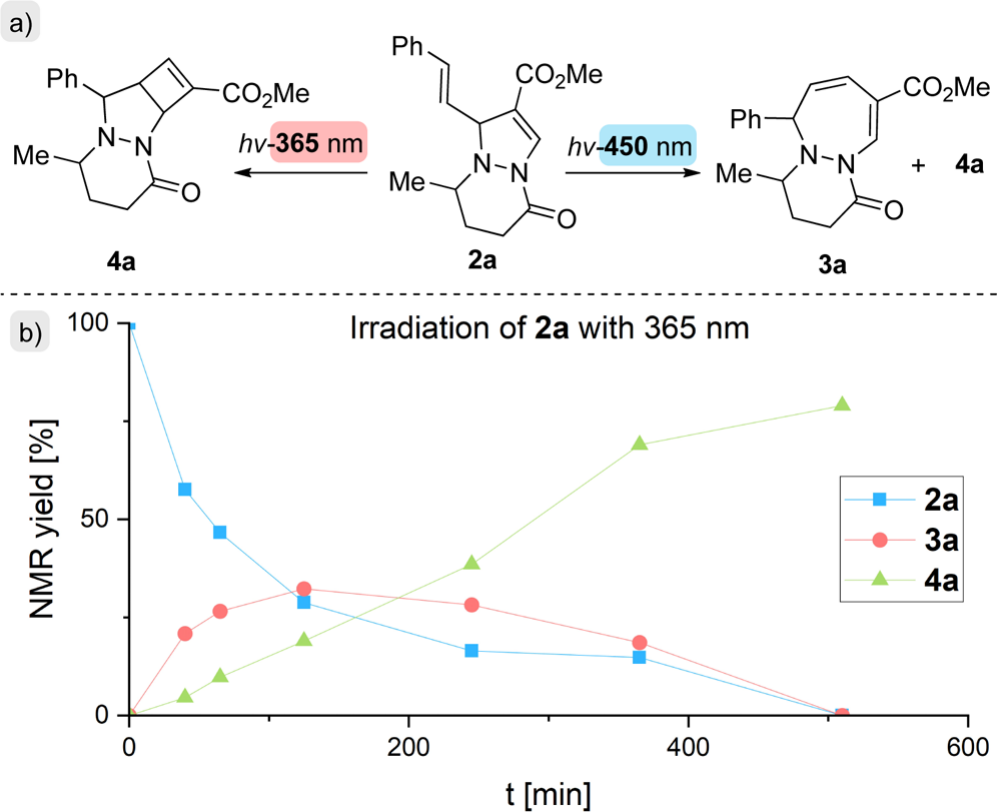
Photoinduced Transformations of **2a**

a Formation of **3a** and **4a** under blue/UV-A light.
b Time-dependend NMR yields of **2a**, **3a** and **4a** during irradiation of **2a** with 365 nm light [**2a** (0.1 mmol), CD_2_Cl_2_ (0.6 mL), Ar]. For irradiation with 450 nm light see (Figure S1).

**Scheme 4:**
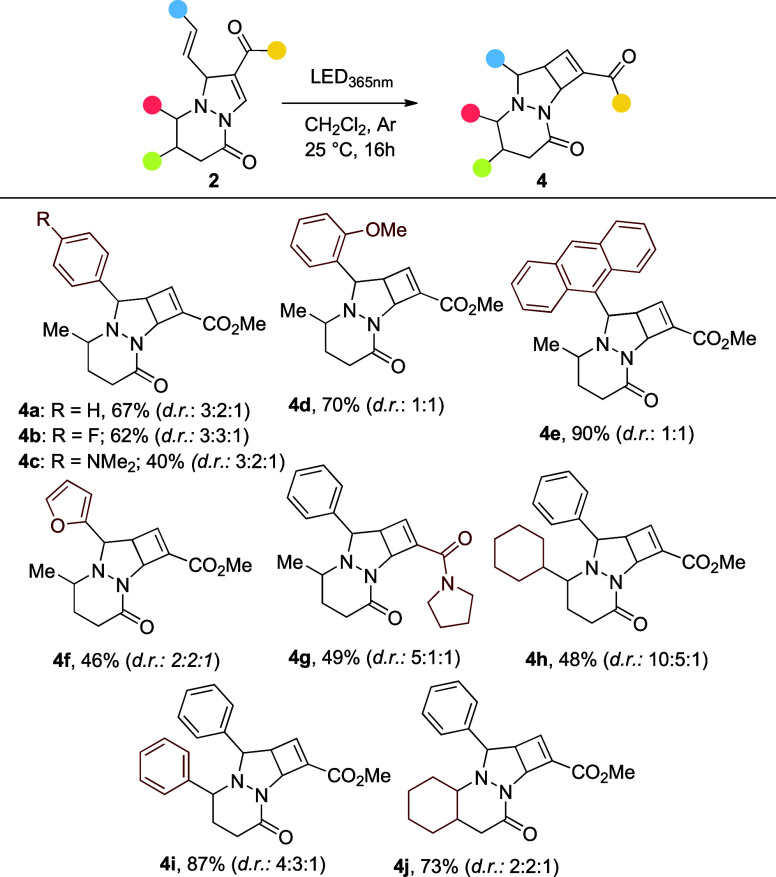
Synthesis of Tricyclic Products from 2[Fn s4fn1]

a
**2** (0.3 mmol), DCM (2.0 mL), 25 °C, Ar, LED-365 nm, 16 h.

Under the optimized reaction conditions, a series of cyclobuta­[*c*]­pyrazolo­[1,2-*a*]­pyridazines **4** was synthesized (Scheme 4). Here, most functional groups were well tolerated. Interestingly, the anthracene-substituted pyrazolo­[1,2-*a*]­pyridazine **2e** afforded the desired product **4e** in the highest yield (90%) despite being the most sterically hindered. Notably, most transformations yielded three diastereoisomers of **4**. In contrast to **2**, products **4** showed no signs of susceptibility to oxidation, even after over 6 months of storage under air in the dark (see Supporting Information). These tricyclic frameworks are stable, rigid, 3D-rich structures with multiple stereocenters, which may serve as versatile scaffolds for further functionalization.

As observed previously with pyrazolo­[1,2-*a*]­pyrazolones,^54,58^ irradiation of alkyl-substituted compounds **2k**–**n** was expected to induce the formal 1,6-hydrogen atom transfer (HAT), yielding the corresponding 4-(1*H-*pyrazol-1-yl)­butanals **6**. Consistent with this expectation, irradiation of **2k** and **2n** at 365 nm produced only trace amounts of the tricyclic products **4**, while efficiently giving the aldehydes **6e** and **6f** in 75% and 99% yield, respectively (Scheme 6-II).

Having demonstrated the photochemical diversification of **2**, we next turned our attention to oxidation processes that could further expand the accessible structural space. Given the potential functional relevance of the additional OH group in *E-* **2a**-**OH**, we sought to investigate its formation and reactivity in more detail. During the synthesis of products **2**, we observed that the reaction was oxygen sensitive. When reaction of **1a** with cinnamaldehyde was carried out under air for an extended period, the byproduct *E-* **2a**-**OH** (^3^ *J*_HC=CH_ = 16.67 Hz) was formed in 80% NMR yield (Table 1, entry 13). As observed with pyrazolo­[1,2-*a*]­pyrazolones,^58,59^ the allylic position appears to be prone to oxidation. Prolonged reaction of **1a** with cinnamaldehyde and methyl propiolate under air therefore gave *E-* **2a**-**OH**, which was isolated in 73% yield (Scheme 5, path A). To verify that this transformation proceeds also directly from **2a**, we heated **2a** under air (DCM, 50 °C, 72 h) and obtained *E-* **2a**-**OH** in 85% isolated yield (Scheme 5, path B).

**Scheme 5:**
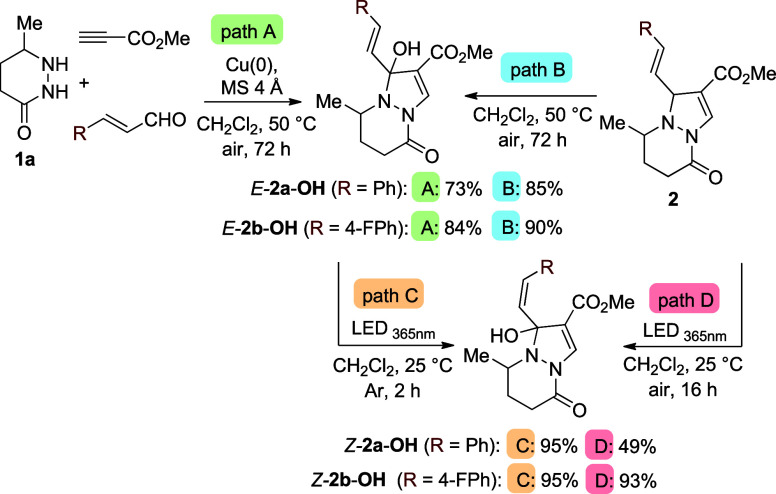
Formation of Products *Z-* **2-OH** and *E-* **2a**-**OH** ^a^

a Isolated yields. Path A: **1a** (1.0 mmol), 3-(hetero)­aryl-enal (1.5 mmol), methyl propiolate (1.1 mmol), Cu(0) (0.7 mmol), DCM (5.0 mL), MS 4 Å (100 mg), 50 °C, air, 72 h. Path B: **2** (1.0 mmol), DCM (5.0 mL), 50 °C, air, 72 h. Path C: *E-* **2**-**OH** (0.3 mmol), DCM (2.0 mL), 25 °C, Ar, LED-365 nm, 2 h. Path D: **2** (0.3 mmol), DCM (2.0 mL), 25 °C, air, LED-365 nm, 16 h.

Since *E-* **2a**-**OH** absorbs visible light, we investigated its potential for photochemical transformations. Notably, the irradiation of *E-* **2a**-**OH** at 365, 450, or 500 nm resulted in quantitative photoisomerization to the corresponding *Z*-isomer *Z-* **2a**-**OH** (^3^ *J*_HC=CH_ = 12.25 Hz), which was isolated in 95% yield (Scheme 5, path C). In contrast to **2a**, which readily undergoes C–N bond cleavage under 365 nm irradiation to give diazepine **3a** and tricyclic products **4a**/**4a′**/**4a″**, the oxidized analogue *E* **-2a**-**OH** selectively undergoes photoisomerization to *Z* **-2a**-**OH**.

To gain more information about the observed differences in reactivity, the isomerization process was studied experimentally and theoretically using a DFT approach. A comparison of reaction kinetics for the transformation of **2a** to **4a** and *E* **-2a**-**OH** to *Z* **-2a**-**OH** reveals fast isomerization of *E* **-2a**-**OH** (85% in 20 min) compared to the ring transformation of **2a** (see Supporting Information for details). TD-DFT calculations demonstrate that, for both substrates, the hole NTO associated with the S^0^ to S^1^ transition is mostly localized on the –N–N– moiety lone pairs, with only a minor π contribution, while the particle NTO resembles a π* orbital of the conjugated –C=C–C=O substructure of **2a** and *E* **-2a**-**OH**. The corresponding NTO pairs account for approximately 98% and 99% of the transition, respectively. Both substrates are also characterized by a similar S^1^ to T^1^ energy gap (ΔΔ*E* = 0.2 eV), with the T^1^ state of *E* **-2a**-**OH** being higher in energy by 3.1 kcal/mol. However, the corresponding “phantom” triplet (torsion angle Ph–C = C-, 90°) is considerably lower in energy (ΔΔ*G* = 3.7 kcal/mol) for *E* **-2a**-**OH**, which makes it more prone to isomerization. Moreover, the absorption maximum of *Z* **-2a**-**OH** (λ_max_ = 254 nm) is notably blue-shifted relative to that of *E* **-2a**-**OH** (λ_max_ = 298 nm), indicating a less extended conjugated system in the *Z* isomer. As a result, *Z* **-2a**-**OH** is no longer efficiently excited at 365 nm, which suppresses the corresponding ring transformation reaction (see Supporting Information for details).

*Z-* **2a**-**OH** could also be prepared by direct irradiation of **2a** under air (49%, Scheme 5, path D). Under analogous reaction conditions (paths A–D), products *E-* **2b**-**OH** (^3^ *J*_HC=CH_ = 16.62 Hz) and *Z-* **2b**-**OH** (^3^ *J*_HC=CH_ = 11.85 Hz) were obtained in high yields (84–95%) (Scheme 5). These results reveal that mild aerobic oxidation and subsequent photoisomerization enable access to distinct structures and further expand the structural space accessible from **2**.

To further demonstrate the versatility of our platform for the synthesis of pyrazolo­[1,2-*a*]­pyridazinones and their derivatives, we next explored the formation and derivatization of compounds **5a**/**5a′**. As noted earlier, reactions of **1a** with β-substituted (*R* ≠ *H*) aldehydes did not yield the expected products **2**. Instead, two equivalents of methyl propiolate were consumed, affording (pyrazolo­[1,2-*a*]­pyridazine-1-yl)­acetate diastereoisomers **5a**/**5a′** (Scheme 6-Ia). Further investigation revealed that under standard conditions (DCM, MS 4 Å, 50 °C, Ar, 16 h), in the absence of cinnamaldehyde and with excess methyl propiolate (2.5 equiv), **1a** underwent the same transformation, giving **5a/5a′** in 91% isolated yield (Scheme 6-Ib). Using 1.5 equiv of methyl propiolate gave a mixture of **5** and intermediate **Int**, the latter isolated in 15% yield (see Supporting Information). Applying the optimized protocol with 2.5 equiv of methyl propiolate, a series of products **5** was prepared, with separable diastereoisomers in some cases (**5a**–**c**). Like compounds **2**, products **5** exhibit a remarkably large Stokes shift (avg. 188 nm, see Supporting Information).

**Scheme 6:**
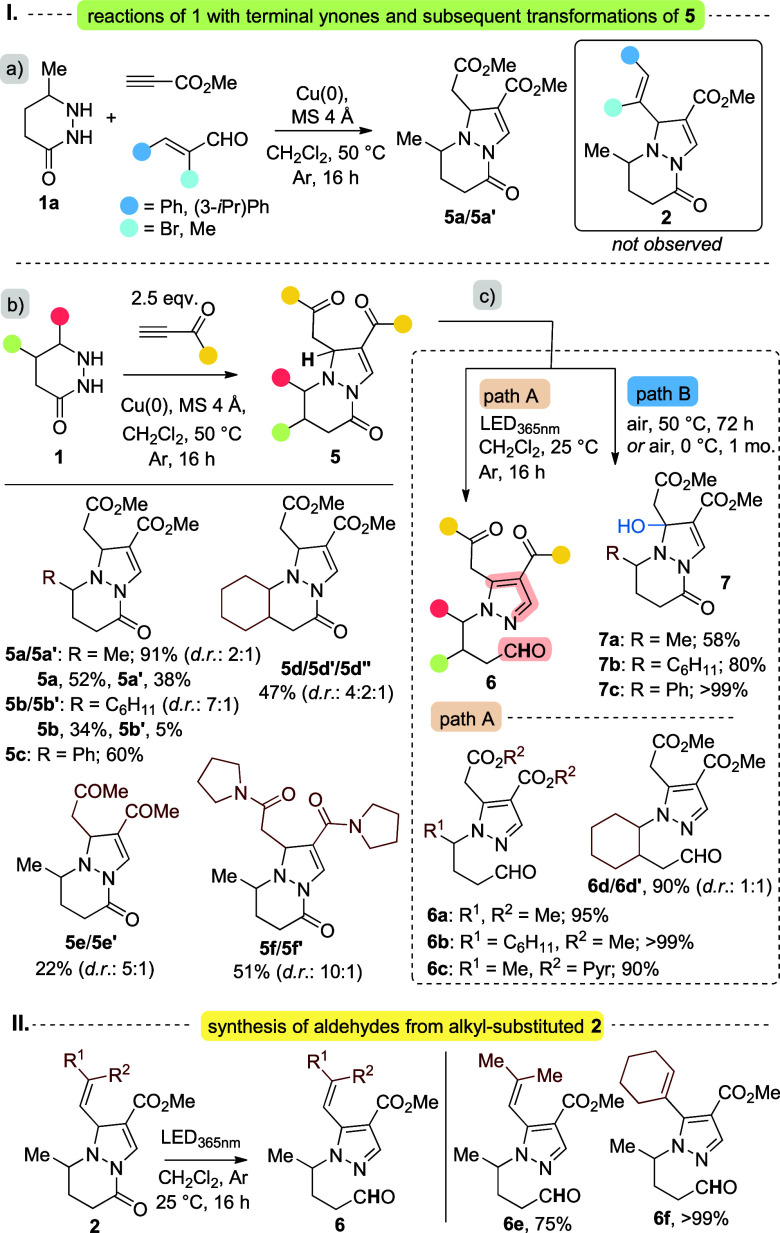
Synthesis of Products **5**, **6**, and **7**

a β-substituted (light blue ≠ H) aldehydes gave **5a**/**5a′**, not **2**.
b
**1** (1.0 mmol), ynone (2.5 mmol), Cu(0) (0.7 mmol), DCM (5.0 mL), MS 4 Å (100 mg), 50 °C, Ar, 16 h.
c Path A: **5** (0.2 mmol), DCM (4.0 mL), 25 °C, Ar, LED-365 nm, 16 h. Path B: **5** (0.2 mmol), air; [ACE vial, DCM (4.0 mL), 50 °C, 72 h] or [no solvent, 0 °C, 1 month].

Irradiation of **5a**/**5a′** (λ_abs_ = 330 nm) with 365 nm light led to the formal 1,6-hydrogen atom transfer (HAT), quantitatively yielding the corresponding **6a** (Scheme 6-Ic, path A), analogous to the alkyl-substituted compounds **2**. Additionally, **6a** could be obtained in a high yield (93%) upon irradiation at 450 nm for an extended period (48 h). Similarly, **5b**, **5d** and **5f** gave aldehydes **6b**, **6c** and **6d**/**6d′** in high yields (90–99%), respectively (Scheme 6-Ic, path A). The photogenerated aldehydes provide reactive sites for possible further functionalization, allowing access to additional structural diversity from this platform.

Alternatively, analogous to the formation of *E-* **2**-**OH**, heating **5a** in air (DCM, 50 °C, 72 h) gave the oxidized product **7a** in 58% isolated yield. Under the same conditions, **5b** gave **7b** in 80% yield (Scheme 6-Ic, path B). Compounds **7** can also be obtained at lower temperatures; for example, **7c** was formed quantitatively from **5c** by slow oxidation in the dark at 0 °C over 1 month (Scheme 6-Ic, path B).

## Conclusion

In conclusion, we have developed an efficient three-component synthesis of pyrazolo­[1,2-*a*]­pyridazinones **2** from tetrahydropyridazinones **1** – a transformation historically challenging due to the ring contraction tendency of **1**.^30–37^ The synthesized products **2** undergo visible-light-induced ring expansion to 1,2-diazepines **3**, followed by a 4π-electrocyclization to afford novel tricyclic cyclobuta­[*c*]­pyrazolo­[1,2-*a*]­pyridazinones **4**. Thermal oxidation and photoisomerization further provide access to products *E-* **2**-**OH** and *Z-* **2**-**OH** via multiple pathways. Additionally, reactions with excess terminal ynones yield (pyrazolo­[1,2-*a*]­pyridazine-1-yl)­acetates **5**, which can be converted into functionalized (1*H-*pyrazol-1-yl)­butanals **6** or oxidized products **7**. Both **2** and **5** display remarkably large Stokes shift (avg. 190 nm).

The variety of scaffolds accessible through subtle changes in reaction conditions, combined with mild conditions and broad functional group tolerance, underscores the synthetic versatility of this platform. Overall, it provides modular, atom-economical access to structurally diverse, 3D-rich pyrazolo­[1,2-*a*]­pyridazinone scaffolds, including tricyclic frameworks, photogenerated aldehydes, and oxidation products, expanding chemical space and enabling further exploration of functionalized derivatives.

## Supplementary Material



## Data Availability

The data underlying this study are available in the published article and its online Supporting Information.

## Notes

Materials and methods, experimental procedures, optimization studies, ^1^H NMR and ^13^C NMR spectra, HRMS, IR, m. p., optical properties measurement, time-course monitoring (PDF)
