# Supplementary material for: One-Pot Synthesis and Photochemical Diversification of Pyrazolo[1,2‑a]pyridazinones into 3D-Rich Scaffolds
Source: J Org Chem. 2026 Jan 17;91(4):1563–70. doi: 10.1021/acs.joc.5c02313 (PMC12865765; doi:10.1021/acs.joc.5c02313)
Supplement: Supplementary file 1 [file jo5c02313_si_001.pdf]

SUPPLEMENTARY INFORMATION

One-Pot Synthesis and Photochemical Diversification of Pyrazolo[1,2-*a*]pyridazinones into 3D-Rich Scaffolds

Ines Babnik, Nejc Petek, Uroš Grošelj, Jurij Svete and Bogdan Štefane[a]*

[a] Faculty of Chemistry and Chemical Technology, University of Ljubljana, Večna pot 113, 1000 Ljubljana.
E-mail: Bogdan.Stefane@fkkt.uni-lj.si.

Contents

1. General information S3
2. Synthesis and characterization of the precursors **PYR-a** and **PYR-b** S4
3. Synthesis of tetrahydropyridazin-3(2*H*)-ones **1** S6
   3.2 Characterization data of compounds **1a**, **1b**, and **1d** S6
4. Optimization studies for the synthesis of **2a** S8
   4.1 Optimization of the reaction conditions S8
5. Synthesis of pyrazolo[1,2-*a*]pyridazinones **2** S9
   5.1 General procedure for the synthesis of compounds **2** S9
   5.2 Characterization data of compounds **2** S9
6. Synthesis and characterization of 1,2-diazepine **3a** S19
7. Optimization studies for the synthesis of **4a** S20
   7.1 Optimization of the reaction conditions S20
   7.2 Air sensitivity test of **4a** S20
8. Synthesis and characterization of tricyclic products **4** S21
   8.1 General procedure for the synthesis of compounds **4** S21
   8.2 Characterization data of compounds **4** S21
   8.3 Time-course monitoring of the reaction of **2a** to **3a** and **4a** S30
      8.3.1 Irradiation of **2a** with 450 nm light S30
      8.3.2 Irradiation of **2a** with 365 nm light S30
      8.3.3 On-off experiment (365 nm) S31
9. Synthesis of products *E*-**2-OH** and *Z*-**2-OH** S32
   9.1 General procedures S32
   9.2 Characterization data of products *E*-**2-OH** and *Z*-**2-OH** S32
10. Synthesis and characterization of pyrazolo[1,2-*a*]pyridazinones **5** S35
   10.1 Reaction of **1a** with β-substituted (R ≠ H) aldehydes, non-conjugated aldehydes and less activated terminal ynones S35
   10.2 General procedure for the synthesis of compounds **5** S36

10.3 Characterization data of compounds **5** S36
11. Synthesis and characterization of intermediate **Int** S41
12. Synthesis and characterization of aldehydes **6** S42
12.1 General procedure for the synthesis of compounds **6** S42
12.2 Characterization data of compounds **6** S42
13. Synthesis and characterization data of oxidized products **7** S45
13.1 General procedure for the synthesis of compounds **7** S45
13.2 Characterization data of compounds **7** S45
14. Optical properties of compounds **2**, **3** and **5** S47
14.1 Optical properties of compounds **2** S47
14.2 Optical properties of compound **3a** S51
14.3 Absorption spectra of **2a**, **3a** and **4a** S51
14.4 Absorption spectra of *E*-**2a-OH** and *Z*-**2a-OH** S51
14.5 Optical properties of compounds **5** S52
15. Investigating photoisomerization of *E*-**2a-OH** to *Z*-**2a-OH** S53
16. References S66
17. NMR spectra S68
17.1 Compounds **PYR** S68
17.2 Tetrahydropyridazin-3(2*H*)-ones **1** S70
17.3 Pyrazolo[1,2-*a*]pyridazinones **2** S74
16.4 1,2-diazepine **3a** S95
17.5 6,5,4-tricyclic products **4** S96
17.6 Products *E*-**2-OH** and *Z*-**2-OH** S117
17.7 Pyrazolo[1,2-*a*]pyridazinones **5** S122
16.8 Intermediate **Int** S131
17.9 Aldehydes **6** S132
16.10 Oxidized products **7** S138

1. **General information**

Photoinduced experiments were performed with a commercially available Penn PhD Photoreactor m2 (Penn Photon Devices, USA). Reactions were performed with stirring in borosilicate vials, which were placed approximately 1 cm from the light source. UV-*a* (365 nm) and blue (450 nm) lights were used at 100% intensity, unless specified differently.

NMR spectra were recorded with Bruker Avance III 500 MHz and 600 MHz NMR instruments at 300 K. Proton spectra were referenced to residual $CHCl_3$ in deuterated chloroform (δ = 7.26 ppm). Carbon spectra were referenced to the ^{13}C signal of $CDCl_3$ (δ = 77.16 ppm) in deuterated chloroform. Chemical shifts (δ) are given in ppm. Coupling constants are given in Hz. Multiplicities are indicated as: s (singlet), d (doublet), t (triplet), q (quartet), quint (quintet), sept (septet), m (multiplet) and bs (broad singlet).

Mass spectra were recorded on Agilent 6224 Accurate Mass TOF LC/MS spectrometer (Agilent Technologies, Santa Clara, CA, USA) and IR spectra on a Bruker FTIR Alpha Platinum spectrophotometer (Bruker, Billerica, MA, USA).

Absorption spectra were recorded on Cary 60 UV-VIS Spectrophotometer (Agilent Technologies, Santa Clara, CA, USA). Photoluminescence spectra were recorded on Cary Eclipse Fluorescence Spectrophotometer (Agilent Technologies, Santa Clara, CA, USA).

Thin-layer chromatography (TLC) was performed on aluminum backed silica plates (0.2 mm, 60 F254, Sigma-*a*ldrich, St. Louis, MO, USA). Visualization of TLC (254 nm and/or 366 nm, Camag, Muttenz, Switzerland) was performed by fluorescence. Column chromatography (CC) was performed on silica gel (particle size: 35–70 µm, Sigma-*a*ldrich, St. Louis, MO, USA).

Commercially available compounds were purchased from Sigma-*a*ldrich, BLD Pharm Germany, TCI and ABCR, and were used without further purification. Dichloromethane (CH_2Cl_2) was purchased from J.T. Baker in CYCLE-TAINER® delivery drums. All other solvents were used as obtained from Sigma-*a*ldrich and Honeywell.

2. Synthesis and characterization of the precursors PYR-a and PYR-b

Compound PYR-a: 4,4a,5,6,7,8-hexahydrocinnolin-3(2*H*)-one

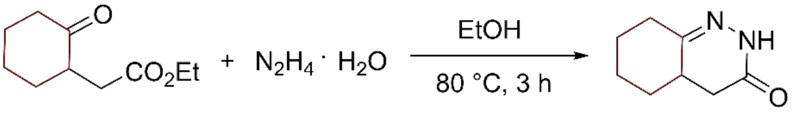

PYR-a

Scheme S1: Synthesis of the precursor **PYR-a**.

A round flask was charged with ethyl-2-(2-oxocyclohexyl)acetate (1.0 mmol), hydrazine monohydrate (1.5 mmol) and EtOH (8.0 mL). The reaction mixture was reacted at 80 °C (heating mantle) for 3 h. After the reaction was completed, the solvent was removed under reduced pressure and the crude reaction mixture was purified by column chromatography – CC (DCM/MeOH=30:1, R*f* = 0.35), affording **PYR-a** as a white crystalline solid (149 mg, 98% yield). The NMR data are in accordance with the literature data.[1,2]

^{1}H NMR (500 MHz, $CDCl_3$) δ 8.25 (br s, 1H), 2.66 (dd, *J* = 17.2, 8.6 Hz, 1H), 2.58–2.50 (m, 2H), 2.22–2.13 (m, 2H), 2.10–2.07 (m, 1H), 1.97–1.95 (m, 1H), 1.87–1.84 (m, 1H), 1.50–1.38 (m, 2H), 1.33–1.25 (m, 1H).

^{13}C{1H} NMR (500 MHz, $CDCl_3$) δ 166.5, 155.7, 34.46, 33.90, 33.81, 33.10, 25.7, 24.6.

IR (neat, cm^{-1}): $\tilde{\nu}$= 3208, 2932, 1668, 1378, 1356, 1325, 785, 732.

HRMS (ESI): *m*/*z* calcd for $C_8H_{13}N_2O$: 153.1022 [*M*+H]$^+$; found: 153.1022.

mp.: 106–108 °C.

Compounds PYR-b, 1b and 1c

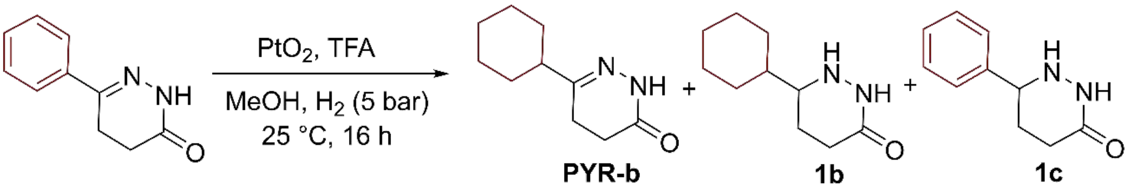

PYR-b **1b** **1c**

Scheme S2: Synthesis of compounds **PYR-b**, **1b**, and **1c**.

A hydrogenation flask was charged with 6-phenyl-4,5-dihydropyridazin-3(2*H*)-one (2.9 mmol), Adams' catalyst (PtO_2, 10 wt%, 50 mg), trifluoroacetic acid (TFA, 5 drops), and MeOH (40 mL). The reaction mixture was purged with argon and subsequently hydrogenated under 5 bar H_2 at 25 °C for 24 hours. The crude reaction mixture was filtered through a pad of Celite (washed with MeOH) and partially separated by CC (DCM/MeOH=30:1), affording pure **PYR-b** (157 mg, 30% yield) and a mixture of **1b** and **1c** (308 mg, **1b**/**1c** = 1.2:1). The **1b**/**1c** mixture (1.2:1) was used directly in the synthesis of **2i** and **5c** without further purification or full characterization.

PYR-b: 6-cyclohexyl-4,5-dihydropyridazin-3(2*H*)-one: isolated as a white crystalline solid. The NMR data are in accordance with the literature data.[3,4]

^{1}H NMR (600 MHz, $CDCl_3$) δ 8.60 (br s, 1H), 2.45 (ddd, *J* = 8.6, 7.0, 1.7 Hz, 2H), 2.39 (ddd, *J* = 9.1, 7.0, 1.8 Hz, 2H), 2.24–2.19 (m, 1H), 1.84–1.75 (m, 4H), 1.71–1.67 (m, 1H), 1.33–1.24 (m, 4H), 1.22–1.15 (m, 1H).

^{13}C{1H} NMR (600 MHz, $CDCl_3$) δ 168.0, 159.8, 45.1, 29.9, 26.56, 26.04, 25.99, 23.2.

IR (neat, cm^{-1}): $\tilde{\nu}$ = 3245, 3112, 1703, 1672, 1333, 1233, 958, 738.

HRMS (ESI): *m*/*z* calcd for $C_{10}H_{17}N_2O$: 181.1335 [*M*+H]$^+$; found: 181.1333.

Compound 1b: 6-cyclohexyltetrahydropyridazin-3(2*H*)-one

For full characterization of compound **1b**, see Section 3.2.

Compound 1c: 6-phenyltetrahydropyridazin-3(2*H*)-one

Corresponding ^{1}H NMR resonances were extracted from the spectrum of the **1b**/**1c** mixture (1.2:1). The NMR data are in accordance with the literature data.[3,5]

^{1}H NMR (600 MHz, $CDCl_3$) δ 7.50 (br s, 1H), 7.37–7.29 (m, 5H), 7.23 (br s, 1H), 4.16 (dd, *J* = 10.2, 4.3 Hz, 1H), 2.65–2.58 (m, 2H), 2.42–2.37 (m, 1H), 2.22–2.15 (m, 1H).

HRMS (ESI): *m*/*z* calcd for $C_{10}H_{13}N_2O$: 177.1022 [*M*+H]$^+$; found: 177.1017.

3. Synthesis of tetrahydropyridazin-3(2*H*)-ones 1

3.1 General procedure for the synthesis of compounds **1a**, **1b**, and **1d**

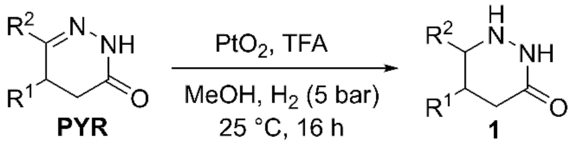

Scheme S3: Synthesis of tetrahydropyridazin-3(2*H*)-ones **1**.

A hydrogenation flask was charged with **PYR** (2.0 mmol), Adams' catalyst (PtO_2, 10 wt%), trifluoroacetic acid (TFA, 3–5 drops), and MeOH (30 mL). The reaction mixture was purged with argon and subsequently hydrogenated under 5 bar H_2 at 25 °C for 16 hours. The crude mixture was filtered through a pad of Celite (washed with MeOH) and was purified by CC (DCM/MeOH=30:1) to afford compound **1**.

3.2 Characterization data of compounds **1a**, **1b**, and **1d**

Compound 1a: 6-methyltetrahydropyridazin-3(2*H*)-one

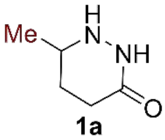

1a

Prepared according to the general procedure from 6-methyl-4,5-dihydropyridazin-3(2*H*)-one (15.0 mmol), Adams' catalyst (164 mg), TFA (5 drops), and EtOH (40 mL). The reaction mixture was purged with argon and subsequently hydrogenated under 5 bar H_2 at 25 °C for 18 h. Some of the staring material was left unreacted and was not hydrogenated even when the reaction mixture was reacted for a prolonged time. After the reaction reached 65% conversion, the crude mixture was filtered through a pad of Celite (washed with EtOH) and was purified by CC (DCM/MeOH=30:1, R*f* = 0.21) to afford compound **1a** as a white crystalline solid (1.027 g, 60% yield). The NMR data are in accordance with the literature data.[6,7]

^{1}H NMR (500 MHz, $CDCl_3$) δ 7.43 (br s, 1H), 3.63 (br s, 1H), 3.13 (ddq, *J* = 10.2, 7.7, 6.6 Hz, 1H), 2.50 (ddd, *J* = 17.3, 7.9, 4.1 Hz, 1H), 2.45 (ddd, *J* = 17.3, 9.2, 7.7 Hz, 1H), 2.07 (dp, *J* = 13.7, 4.1 Hz, 1H), 1.58 (dtd, *J* = 13.7, 9.2, 7.9 Hz, 1H), 1.15 (d, *J* = 6.6 Hz, 3H).

^{13}C{1H} NMR (500 MHz, $CDCl_3$) δ 172.1, 50.6, 31.0, 28.8, 19.1.

IR (neat, cm^{-1}): $\tilde{v}$ = 3223, 2185, 2963, 2889, 1635, 1398, 1232, 976, 891, 814, 761.

HRMS (ESI): *m*/*z* calcd for $C_5H_{11}N_2O$: 115.0866 [*M*+H]$^+$; found: 115.0861.

mp.: 69–71 °C.

Compound 1b: 6-cyclohexyltetrahydropyridazin-3(2*H*)-one

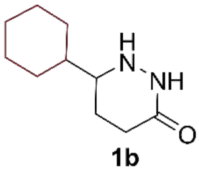

1b

Prepared according to the general procedure from **PYR-b** (0.5 mmol) and Adams' catalyst (10 mg). The reaction mixture was purged with argon and subsequently hydrogenated under 5 bar H_2 at 25 °C for 16 h. Some of the staring material was left unreacted and was not hydrogenated even when the reaction mixture was reacted for a prolonged time. After the reaction reached 80% conversion, the crude mixture was filtered through a pad of Celite (washed with EtOH) and was purified by CC (DCM/MeOH=30:1, R*f* = 0.21) to afford compound

1b as a white crystalline solid (63 mg, 70% yield). The NMR data are in accordance with the literature.[3]

^{1}H NMR (500 MHz, $CDCl_3$) δ 7.16 (br s, 1H), 3.61 (br s, 1H), 2.72–2.67 (m, 1H), 2.49 (ddd, *J* = 17.4, 7.9, 4.8 Hz, 1H), 2.41 (ddd, *J* = 17.4 Hz, 9.0, 7.6 Hz, 1H), 2.05 (ddt, *J* = 14.3, 7.6, 4.8 Hz, 1H), 1.92 (ddt, *J* = 12.8, 3.7, 1.9 Hz, 1H), 1.77–1.71 (m, 2H), 1.66 (tdd, *J* = 9.0, 7.9, 5.2 Hz, 3H), 1.30 (dddt, *J* = 14.3, 10.4, 6.7, 3.3 Hz, 1H), 1.25–1.12 (m, 3H), 1.06 (td, *J* = 12.1, 3.3 Hz, 1H), 0.97 (qd, *J* = 12.1, 3.7 Hz, 1H).

^{13}C{1H} NMR (500 MHz, $CDCl_3$) δ 173.2, 59.9, 40.7, 29.87, 29.58, 29.09, 26.48, 26.46, 26.29, 26.18.

IR (neat, cm^{-1}): $\tilde{v}$= 3258, 2916, 2849, 1657, 1146, 1401, 798, 784.

HRMS (ESI): *m*/*z* calcd for $C_{10}H_{19}N_2O$: 183.1492 [*M*+H]$^+$; found: 183.1491.

mp.: 110.8–111.5 °C.

Compounds 1d/1d': octahydrocinnolin-3(2*H*)-one

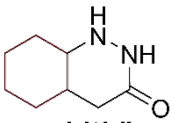

1d/1d'

Prepared according to the general procedure from **PYR-*a*** (2.0 mmol) and Adams' catalyst (33 mg). The reaction mixture was purged with argon and subsequently hydrogenated under 5 bar H_2 at 25 °C for 22 h. The crude mixture was filtered through a pad of Celite (washed with MeOH) and was purified by CC (DCM/MeOH=30:1, R*f* = 0.12) to afford diastereoisomers **1d/1d'** as a racemic mixture (234 mg, 75% yield, *d.r.* = 1.3:1).

^{13}C{1H} NMR (500 MHz, $CDCl_3$) δ 170.93, 170.83, 58.6, 53.3, 40.7, 36.6, 34.00, 33.70, 32.3, 30.0, 28.4, 26.8, 25.20, 25.18, 22.83, 22.24.

IR (neat, cm^{-1}): $\tilde{v}$= 3207, 3036, 2922, 2858, 1637, 1393, 1155, 980, 824.

HRMS (ESI): *m*/*z* calcd for $C_8H_{15}N_2O$: 155.1179 [*M*+H]$^+$; found: 155.1179.

mp.: 126–128 °C.

Corresponding ^{1}H NMR resonances for each diastereoizomer:

1d (major):

^{1}H NMR (500 MHz, $CDCl_3$) δ 7.06 (br s, 1H), 4.23 (d, *J* = 8.4 Hz, 1H), 3.14–3.10 (m, 1H), 2.58 (dd, *J* = 18.6, 8.4 Hz, 1H), 2.25 (dd, *J* = 18.6, 5.3 Hz, 1H), 2.09 (dd, *J* = 18.4, 11.5 Hz, 1H), 1.66–1.56 (m, 3H), 1.52–1.42 (m, 3H), 1.41–1.32 (m, 1H), 1.28–1.18 (m, 1H).

1d' (minor):

^{1}H NMR (500 MHz, $CDCl_3$) δ 7.22 (br s, 1H), 3.51 (d, *J* = 12.3 Hz, 1H), 2.60 (dd, *J* = 18.4, 6.5 Hz, 1H), 2.52 (br td, *J* = 11.7, 4.0 Hz, 1H), 2.14–2.08 (m, 1H), 1.98–1.94 (m, 1H), 1.89–1.83 (m, 1H), 1.82–1.77 (m, 1H), 1.76–1.71 (m, 2H), 1.52–1.42 (m, 1H), 1.41–1.32 (m, 1H), 1.05–0.95 (m, 2H).

4. Optimization studies for the synthesis of 2a

4.1 Optimization of the reaction conditions

Table S1: ^1H NMR yields of **2a** and *E*-**2a-OH**.

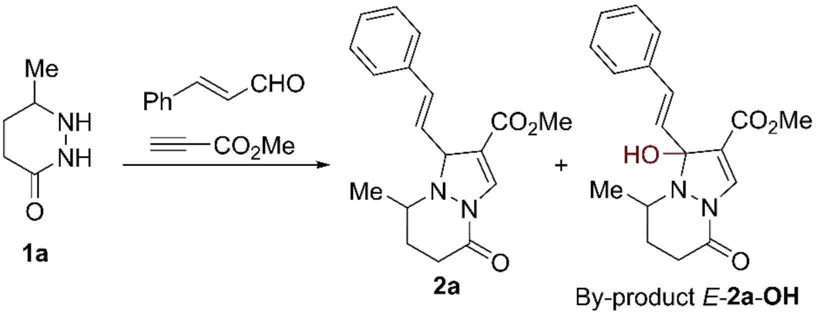

Entry	Deviation from standard reaction conditions[a]	Yield **2a** (*E*-**2a-OH**) [%][b]
1	None	81 (n.d.)
2	TFA instead of MS, 25 °C	20 (n.d.)
3	TFA instead of MS, 50 °C	41 (n.d.)
4	25 °C instead of 50 °C	59 (n.d.)
5	Et_3N instead of MS, 25 °C	n.d. (n.d.)
6	No Cu(0), 50 °C	75 (n.d.)
7	$Zn(OTf)_2$ instead of Cu(0),[c] 50 °C	22 (n.d.)
8	$Zn(OTf)_2$ instead of Cu(0),[c] 25 °C	21 (n.d.)
9	MeOH instead of DCM	78 (n.d.)
10	ACN instead of DCM	51 (n.d.)
11	THF instead of DCM	49 (n.d.)
12	Air atmosphere (16 h)	73 (10)
13	Air atmosphere (72 h)	n.d. (80)[d]
14	Purged with oxygen	43 (n.d.)
15	Purged with oxygen, no Cu(0)	65 (10)

[a] **1a** (0.2 mmol), cinnamaldehyde (0.3 mmol), methyl propiolate (0.22 mmol), DCM (1.0 mL), copper(0) (0.14 mmol), molecular sieves (MS, 4 Å, 30 mg), under Ar, 50 °C for 16 h. [b]NMR yields of **2a** (*E*-**2a-OH** in brackets). [c]$Zn(OTf)_2$ (0.1 eq.). [d]By-product *E*-**2a-OH** was isolated in 73% yield. n.d. = not detected.

Standard protocol: a vial was charged with **1** (0.2 mmol), cinnamaldehyde (0.3 mmol), CH_2Cl_2 (1.0 mL), methyl propiolate (0.22 mmol), copper(0) (0.14 mmol), molecular sieves (4 Å; 30 mg) and was sealed off with a screw cap with a septum. The reaction mixture was purged with argon and was reacted in the dark at 50 °C (sand bath) for 16 h. NMR yields were determined with 1,3,5-trimethoxybenzene as an internal standard.

Purging the reaction mixture with oxygen resulted in a complex mixture of products, indicating that the reaction is oxygen-sensitive (entry 14). In contrast, performing the reaction under oxygen in absence of copper did not lead to additional side products. These observations suggest that, under oxygen, Cu(0) is oxidized to copper-oxo species capable of generating radical intermediates, which in turn promote side-product formation.[8]

On the other hand, under milder oxidative conditions (ambient air), the by-product *E*-**2a-OH** was obtained as the main product due to oxidation of **2a** at the allylic position (entries 12 and 13). For details on the isolation and characterization of *E*-**2a-OH** see Section 9.2.

5. Synthesis of pyrazolo[1,2-*a*]pyridazinones 2

5.1 General procedure for the synthesis of compounds **2**

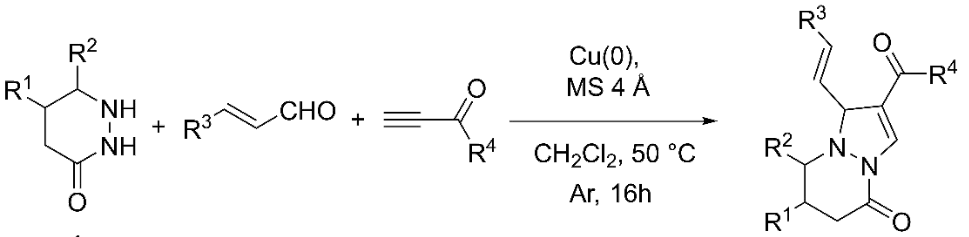

1 **2**

Scheme S4: Synthesis of pyrazolo[1,2-*a*]pyridazinones **2**.

A vial was charged with **1** (1.0 mmol), 3-(hetero)aryl-enal (1.5 mmol), CH_2Cl_2 (5.0 mL), terminal ynone (1.1 mmol), copper(0) (0.7 mmol), molecular sieves (4 Å; 100 mg) and was sealed off with a screw cap with a septum. The reaction mixture was purged with argon and was reacted in the dark at 50 °C (sand bath) for 16 h. Products **2** were obtained by preparative reversed-phase column chromatography (RP-CC) – initial isocratic hold at 80:20 H_2O/MeCN for 10 min, followed by gradient from 80:20 H_2O/ MeCN to 30:70 H_2O/ MeCN over 30 min (total runtime: 40 min).

5.2 Characterization data of compounds **2**

Compounds 2a/2a': Methyl 8-methyl-5-oxo-1-styryl-5,6,7,8-tetrahydro-1*H*-pyrazolo[1,2-*a*]pyridazine-2-carboxylate.

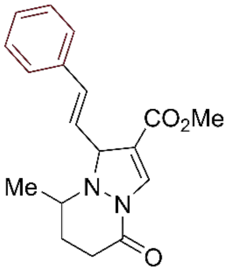

2a/2a'

Prepared according to the general procedure from **1a** (5.2 mmol), cinnamaldehyde (10.4 mmol) and methyl propiolate (6.2 mmol) in CH_2Cl_2 (22 mL). The product was isolated as a mixture of diastereoisomers **2a**/**2a'** by CC (PE/EA = 1:1) (1.121 g, 69% yield, *d.r.* = 5:1), and the diastereoisomers were further separated by preparative RP-CC (general purification procedure).

2a, major diastereoisomer, isolated as a yellow oil (942 mg, 58%).

^{1}H NMR (600 MHz, $CDCl_3$) δ 7.74 (s, 1H), 7.40 (d, *J* = 9.6 Hz, 2H), 7.31 (t, *J* = 7.7 Hz, 2H), 7.24 (t, *J* = 7.3 Hz, 1H), 6.68 (d, *J* = 16.2 Hz, 1H), 6.31 (d, *J* = 22.1 Hz, 1H), 5.03 (d, *J* = 6.4 Hz, 1H), 3.73 (s, 3H), 3.05 (dt, *J* = 8.2, 6.2 Hz, 1H), 2.65 (dt, *J* = 16.2, 8.2 Hz, 1H), 2.48 (ddd, *J* = 16.7, 7.7, 5.6 Hz, 1H), 1.98 (ddd, *J* = 14.0, 7.3, 5.8 Hz, 1H), 1.84 (dtd, *J* = 14.0, 8.4, 5.6 Hz, 1H), 1.20 (d, *J* = 6.2 Hz, 3H).

^{13}C{1H} NMR (600 MHz, $CDCl_3$) δ 166.2, 164.3, 136.7, 131.58, 131.30, 128.65, 128.37, 127.85, 126.7, 112.7, 70.3, 57.7, 51.7, 29.59, 29.10, 19.5.

IR (neat, cm^{-1}): $\tilde{v}$= 2949, 1709, 1681, 1616, 1405, 1214, 1124, 748

HRMS (ESI): *m*/*z* calcd for $C_{18}H_{21}N_2O_3$: 313.1547 [*M*+H]$^+$; found: 313.1551.

2a', minor diastereoisomer, isolated as a yellow oil (179 mg, 11%).

^{1}H NMR (600 MHz, $CDCl_3$) δ 7.83 (s, 1H), 7.41–7.39 (m, 2H), 7.33–7.31 (m, 2H), 7.27–7.25 (m, 1H), 6.68 (d, *J* = 15.7 Hz, 1H), 6.22 (dd, *J* = 8.3, 15.7 Hz, 1H), 5.02 (d, *J* = 8.3, 1H), 3.72 (s, 3H), 3.30 (dqd, *J* = 12.5, 6.3, 4.1 Hz, 1H), 2.56–2.53 (m, 2H), 2.09–2.05 (m, 1H), 1.76 (ddd, *J* = 16.2, 13.8, 8.1 Hz, 1H), 1.17 (d, *J* = 6.2 Hz, 3H).

^{13}C{1H} NMR (600 MHz, $CDCl_3$) δ 164.42, 163.96, 136.2, 133.7, 132.6, 128.74, 128.28, 126.9, 124.2, 114.5, 67.8, 51.69, 51.02, 28.66, 28.56, 15.8.

IR (neat, cm^{-1}): $\tilde{v}$ = 2949, 1707, 1663, 1609, 1403, 1249, 1123, 749.

HRMS (ESI): *m*/*z* calcd for $C_{18}H_{21}N_2O_3$: 313.1547 [*M*+H]$^+$; found: 313.1553.

Compounds 2b/2b': Methyl 1-(4-fluorostyryl)-8-methyl-5-oxo-5,6,7,8-tetrahydro-1*H*-pyrazolo[1,2-*a*]pyridazine-2-carboxylate.

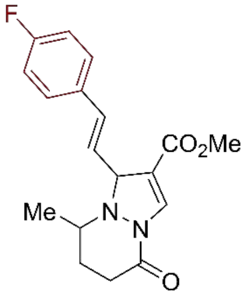

2b/2b'

Prepared according to the modified general procedure from **1a** (1.0 mmol), p-fluorocinnamaldehyde (1.5 mmol) and methyl propiolate (1.2 mmol). The reaction was stirred under argon in the dark at 25 °C for 16 h. The product was isolated as a mixture of diastereoisomers **2b**/**2b'** by CC (PE/EA = 1:1) (149 mg, 45% yield, *d.r.* = 5:1), and the diastereoisomers were further separated by preparative RP-CC (general purification procedure).

2b, major diastereoisomer, isolated as a yellow oil (123 mg, 37%).

^{1}H NMR (600 MHz, $CDCl_3$) δ 7.73 (d, *J* = 1.4 Hz, 1H), 7.36–7.34 (m, 2H), 7.01–6.98 (m, 2H), 6.63 (d, *J* = 15.8 Hz, 1H), 6.22 (dd, *J* = 15.8, 6.3 Hz, 1H), 5.01 (dd, *J* = 6.3, 1.4 Hz, 1H), 3.73 (s, 3H), 3.04 (dp, *J* = 8.0, 6.2 Hz, 1H), 2.64 (dt, *J* = 16.5, 8.0 Hz, 1H), 2.48 (ddd, *J* = 16.5, 7.5, 5.5 Hz, 1H), 1.98 (dtd, *J* = 13.7, 7.5, 6.2 Hz, 1H), 1.83 (dtd, *J* = 13.7, 8.0, 5.5 Hz, 1H), 1.20 (d, *J* = 6.2 Hz, 3H).

^{13}C{1H} NMR (600 MHz, $CDCl_3$) δ 166.2, 164.31, 163.36, 161.7, 132.93, 131.36, 130.41, 128.32, 128.15, 115.65, 115.50, 112.5, 70.2, 57.7, 51.7, 29.61, 29.12, 19.5.

$\tilde{v}$ = 2950, 1709, 1680, 1617, 1508, 1406, 1217, 1126, 760.

HRMS (ESI): *m*/*z* calcd for $C_{18}H_{20}FN_2O_3$: 331.1452 [*M*+H]$^+$; found: 331.1456.

2b', isolated as a yellow oil (23 mg, 7%).

^{1}H NMR (600 MHz, $CDCl_3$) δ 7.82 (s, 1H), 7.36 (br dd, *J* = 8.7, 6.3 Hz, 2H), 7.01 (t, *J* = 8.7 Hz, 2H), 6.63 (d, *J* = 15.6 Hz, 1H), 6.14 (dd, *J* = 15.6, 8.0 Hz, 1H), 5.00 (d, *J* = 8.0 Hz, 1H), 3.72 (s, 3H), 3.29 (dqd, *J* = 13.0, 6.3, 4.3 Hz, 1H), 2.54 (br t, *J* = 7.0 Hz, 2H), 2.09 (dddd, *J* = 13.0, 10.7, 6.3, 4.3 Hz, 1H), 1.75 (br sextet, *J* = 7.0 Hz, 1H), 1.17 (d, *J* = 6.3 Hz, 3H).

^{13}C{1H} NMR (600 MHz, $CDCl_3$) δ 164.48, 164.01, 163.57, 161.9, 132.63, 132.40, 128.51, 128.45, 124.1, 115.76, 115.62, 114.3, 67.8, 51.73, 51.12, 28.59, 28.52, 15.6.

IR (neat, cm^{-1}): $\tilde{v}$ = 2950, 1709, 1664, 1610, 1508, 1404, 1223, 1124, 761.

HRMS (ESI): *m*/*z* calcd for $C_{18}H_{20}FN_2O_3$: 331.1452 [*M*+H]$^+$; found: 331.1453.

Compounds 2c/2c': Methyl 1-(4-(dimethylamino)styryl)-8-methyl-5-oxo-5,6,7,8-tetrahydro-1*H*-pyrazolo[1,2-*a*]pyridazine-2-carboxylate.

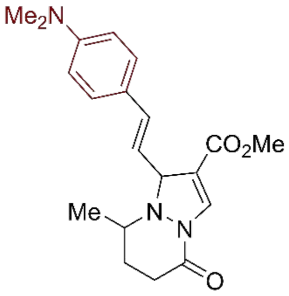

2c/2c'

Prepared according to the general procedure from **1a** (1.0 mmol), *p*-dimethylaminocinnamaldehyde (1.5 mmol) and methyl propiolate (1.2 mmol). The reaction was stirred under argon in the dark at 50 °C (sand bath) for 72 h. The product was isolated as a mixture of diastereoisomers **2c**/**2c'** by CC (PE/EA = 1:1) (249 mg, 70% yield, *d.r.* = 2:1), and the diastereoisomers were further separated by preparative RP-CC (general purification procedure).

2c, major diastereoisomer, isolated as a yellow oil (164 mg, 46%).

^{1}H NMR (600 MHz, $CDCl_3$) δ 7.72 (d, *J* = 1.4 Hz, 1H), 7.28 (br d, *J* = 8.8 Hz, 2H), 6.67 (d, *J* = 8.8 Hz, 2H), 6.56 (d, *J* = 15.9 Hz, 1H), 6.05 (dd, *J* = 15.9, 6.6 Hz, 1H), 4.99 (d, *J* = 6.6 Hz, 1H), 3.71 (s, 3H), 3.03 (ddq, *J* = 12.3, 7.6, 5.9 Hz, 1H), 2.95 (s, 6H), 2.63 (ddd, *J* = 16.3, 8.6, 7.5 Hz, 1H), 2.46 (ddd, *J* = 16.3, 7.6, 5.7 Hz, 1H), 1.96 (dtd, *J* = 14.0, 7.5, 5.9 Hz, 1H), 1.82 (dtd, *J* = 14.0, 8.6, 5.7 Hz, 1H), 1.20 (d, *J* = 5.9 Hz, 3H).

^{13}C{1H} NMR (600 MHz, $CDCl_3$) δ 166.1, 164.4, 150.4, 131.59, 131.11, 127.7, 125.2, 124.0, 113.24, 112.50, 70.8, 57.8, 51.6, 40.7, 29.71, 29.13, 19.5.

IR (neat, cm^{-1}): $\tilde{v}$= 2974, 1710, 1658, 1607, 1522, 1328, 1127, 806.

HRMS (ESI): *m*/*z* calcd for $C_{20}H_{26}N_3O_3$: 356.1969 [*M*+H]$^+$; found: 356.1967.

2c', minor diastereoisomer, isolated as a yellow oil (82 mg, 23%).

^{1}H NMR (600 MHz, $CDCl_3$) δ 7.81 (br s, 1H), 7.29–7.28 (m, 2H), 6.67–6.65 (m, 2H), 6.57 (d, *J* = 15.8 Hz, 1H), 5.95 (dd, *J* = 15.8, 8.4 Hz, 1H), 4.97 (d, *J* = 8.4 Hz, 1H), 3.71 (s, 3H), 3.27 (dqd, *J* = 12.3, 6.2, 3.5 Hz, 1H), 2.96 (s, 6H), 2.53–2.51 (m, 2H), 2.04–1.99 (m, 1H), 1.75 (dq, *J* = 13.7, 8.7 Hz, 1H), 1.16 (d, *J* = 6.2 Hz, 3H).

^{13}C{1H} NMR (600 MHz, $CDCl_3$) δ 164.32, 164.06, 150.6, 134.0, 132.3, 127.9, 124.5, 119.0, 115.3, 112.4, 68.2, 51.7, 50.7, 40.6, 28.92, 28.73, 16.4.

IR (neat, cm^{-1}): $\tilde{v}$= 2949, 1709, 1662, 1606, 1521, 1404, 1123, 726.

HRMS (ESI): *m*/*z* calcd for $C_{20}H_{26}N_3O_3$: 356.1969 [*M*+H]$^+$; found: 356.1961.

Compounds 2d/2d': Methyl 1-(2-methoxystyryl)-8-methyl-5-oxo-5,6,7,8-tetrahydro-1*H*-pyrazolo[1,2-*a*]pyridazine-2-carboxylate.

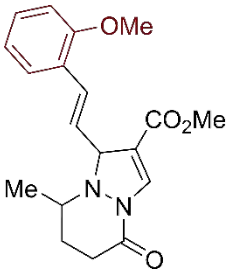

2d/2d'

Prepared according to the general procedure from **1a** (1.0 mmol), *o*-methoxycinnamaldehyde (1.5 mmol) and methyl propiolate (1.2 mmol). The product was isolated as a mixture of diastereoisomers **2d**/**2d'** by CC (PE/EA = 1:1) (219 mg, 64% yield, *d.r.* = 5:1), and the diastereoisomers were further separated by preparative RP-CC (general purification procedure).

2d, major diastereoisomer, isolated as a yellow oil (175 mg, 51%).

^{1}H NMR (600 MHz, $CDCl_3$) δ 7.74 (d, *J* = 1.4 Hz, 1H), 7.44–7.42 (m, 1H), 7.23–7.20 (m, 1H), 6.99 (d, *J* = 15.9 Hz, 1H), 6.92–6.89 (m, 1H), 6.86–6.85 (m, 1H), 6.30 (dd, *J* = 15.9, 6.6 Hz, 1H), 5.02 (dd, *J* = 6.6, 1.4 Hz, 1H), 3.84 (s, 3H), 3.72 (s, 3H), 3.04 (dp, *J* = 8.2, 6.2 Hz, 1H), 2.64 (ddd, *J* = 16.7, 8.4, 7.6 Hz, 1H), 2.47 (ddd, *J* = 16.7, 7.5, 5.7 Hz, 1H), 1.97 (dtd, *J* = 13.6, 7.5, 5.9 Hz, 1H), 1.83 (dtd, *J* = 13.6, 8.4, 5.7 Hz, 1H), 1.20 (d, *J* = 6.2 Hz, 3H).

^{13}C{1H} NMR (600 MHz, $CDCl_3$) δ 166.2, 164.4, 157.0, 131.3, 128.90, 128.78, 127.20, 126.70, 125.79, 120.7, 113.0, 111.0, 71.0, 57.8, 55.6, 51.7, 29.69, 29.15, 19.6.

IR (neat, cm^{-1}): $\tilde{v}$ = 2948, 1709, 1672, 1616, 1405, 1241, 1215, 749.

HRMS (ESI): *m*/*z* calcd for $C_{19}H_{23}N_2O_4$: 343.1652 [*M*+H]$^+$; found: 343.1657.

2d', minor diastereoisomer, isolated as a yellow oil (34 mg, 10%).

^{1}H NMR (600 MHz, $CDCl_3$) δ 7.82 (br s, 1H), 7.43–7.42 (m, 1H), 7.25–7.22 (m, 1H), 6.99 (d, *J* = 15.9 Hz, 1H), 6.92–6.90 (m, 1H), 6.88–6.86 (m, 1H), 6.22 (dd, *J* = 15.9, 8.5 Hz, 1H), 5.01 (d, *J* = 8.5 Hz, 1H), 3.85 (s, 3H), 3.72 (s, 3H), 3.34–3.27 (m, 1H), 2.53 (br t, *J* = 7.0 Hz, 2H), 2.05 (dq, *J* = 16.0, 5.1 Hz, 1H), 1.75 (br sextet, *J* = 7.6 Hz, 1H), 1.17 (d, *J* = 6.1 Hz, 3H).

^{13}C{1H} NMR (600 MHz, $CDCl_3$) δ 164.41, 164.04 157.3, 132.6, 129.33, 128.88, 127.41, 125.26, 124.57, 120.7, 114.9, 111.2, 68.5, 55.7, 51.66, 50.95, 28.76, 28.68, 15.9.

IR (neat, cm^{-1}): $\tilde{v}$ = 2948, 1709, 1666, 1611, 1405, 1243, 1170, 752.

HRMS (ESI): *m*/*z* calcd for $C_{19}H_{23}N_2O_4$: 343.1652 [*M*+H]$^+$; found: 343.1663.

Compounds 2e/2e': Methyl 1-(2-(anthracen-9-yl)vinyl)-8-methyl-5-oxo-5,6,7,8-tetrahydro-1*H*-pyrazolo[1,2-*a*]pyridazine-2-carboxylate.

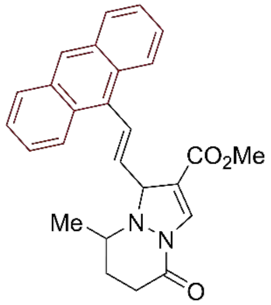

2e/2e'

Prepared according to the general procedure from **1a** (1.0 mmol), 3-(anthracene-9-yl)acrylaldehyde (1.5 mmol) and methyl propiolate (1.2 mmol). The reaction mixture was performed under argon in the dark at 50 °C (sand bath) for 48 h. The product was isolated as a mixture of diastereoisomers **2e**/**2e'** by preparative RP-CC (general purification procedure) (161 mg, 39% yield, *d.r.* = 3:1).

^{13}C{1H} NMR (600 MHz, $CDCl_3$) δ 166.1 (2C), 164.41, 164.30, 136.9, 133.10, 133.08, 132.23, 131.85, 131.50, 131.47, 130.49, 129.67 (2C), 128.84, 128.75, 128.28, 126.82, 126.57 (2C), 125.97, 125.78, 125.74, 125.58, 125.29, 125.24, 114.2, 112.5, 71.0, 68.4, 57.9, 51.82, 51.80, 51.34, 29.68, 29.17, 28.74, 28.71, 19.9, 16.1.

IR (neat, cm^{-1}): $\tilde{v}$ = 2949, 1708, 1665, 1617, 1405, 1248, 732.

HRMS (ESI): *m*/*z* calcd for $C_{26}H_{25}N_2O_3$: 413.1860 [*M*+H]$^+$; found: 413.1857.

Corresponding ^1H NMR resonances for each diastereoizomer:

2e (major):

^1H NMR (600 MHz, $CDCl_3$) δ 8.38 (s, 1H), 8.26–8.24 (m, 2H), 8.01–7.97 (m, 2H), 7.87–7.86 (m, 1H), 7.49–7.45 (m, 5H), 6.14 (dd, *J* = 6.6 Hz, 16.1 Hz, 1H), 5.33–5.31 (m, 1H), 3.81 (s, 3H), 3.24–3.17 (m, 1H), 2.73–2.67 (m, 1H), 2.57–2.50 (m, 1H), 2.10–2.03 (m, 1H), 1.96–1.90 (m, 1H), 1.47 (d, *J* = 6.3 Hz, 3H).

2e' (minor):

^1H NMR (600 MHz, $CDCl_3$) δ 8.40 (s, 1H), 8.22–8.19 (m, 2H), 8.01–7.97 (m, 2H), 7.91–7.90 (m, 1H), 7.51–7.45 (m, 5H), 6.10 (dd, *J* = 16.1, 8.36 Hz, 1H), 5.36–5.34 (m, 1H), 3.85 (s, 3H), 3.54–3.48 (m, 1H), 2.64–2.50 (m, 2H), 1.96–1.83 (m, 2H), 1.41 (d, *J* = 6.2 Hz, 3H).

Compounds 2f/2f': Methyl 1-(2-(furan-2-yl)vinyl)-8-methyl-5-oxo-5,6,7,8-tetrahydro-1*H*-pyrazolo[1,2-*a*]pyridazine-2-carboxylate.

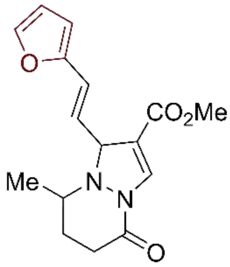

2f/2f'

Prepared according to the general procedure from **1a** (1.0 mmol), 3-(furan-2-yl)acrylaldehyde (1.5 mmol) and methyl propiolate (1.2 mmol). The product was isolated as a mixture of diastereoisomers **2f**/**2f'** by CC (PE/EA = 1:1) (131 mg, 42% yield, *d.r.* = 4:1), and the diastereoisomers were further separated by preparative RP-CC (general purification procedure).

2f, major diastereoisomer, isolated as a yellow oil (103 mg, 33%).

^1H NMR (600 MHz, $CDCl_3$) δ 7.70 (d, *J* = 1.3 Hz, 1H), 7.32 (d, *J* = 1.3 Hz, 1H), 6.51 (d, *J* = 15.8 Hz, 1H), 6.35 (dd, *J* = 3.3, 1.9 Hz, 1H), 6.28 (dd, *J* = 15.8, 6.0 Hz, 1H), 6.24 (d, *J* = 3.3 Hz, 1H), 4.97 (dd, *J* = 6.0, 1.9 Hz, 1H), 3.72 (s 3H), 3.01 (dp, *J* = 8.4, 6.6 Hz, 1H), 2.63 (dt, *J* = 16.5, 8.0 Hz, 1H), 2.46 (ddd, *J* = 16.5, 6.6, 5.4 Hz, 1H), 1.96 (dtd, *J* = 13.7, 8.0, 6.1 Hz, 1H), 1.82 (dtd, *J* = 13.7, 8.4, 5.4 Hz, 1H), 1.15 (d, *J* = 6.1 Hz, 3H).

$^{13}C\{1H\}$ NMR (600 MHz, $CDCl_3$) δ 166.2, 164.3, 152.5, 142.2, 131.2, 127.0, 119.8, 112.57, 111.4, 108.6, 69.6, 57.6, 51.7, 29.54, 29.11, 19.4.

IR (neat, cm^{-1}): $\tilde{v}$= 2950, 1709, 1677, 1617, 1406, 1215, 731.

HRMS (ESI): *m*/*z* calcd for $C_{16}H_{19}N_2O_4$: 303.1339 $[M+H]^+$; found: 303.1337.

2f', minor diastereoisomer, isolated as a yellow oil (25 mg, 8%).

^1H NMR (600 MHz, $CDCl_3$) δ 7.81 (br s, 1H), 6.35 (d, *J* = 1.8 Hz, 1H), 6.49 (d, *J* = 15.8 Hz, 1H), 6.37 (dd, *J* = 3.2, 1.8 Hz, 1H), 6.29 (d, *J* = 3.2 Hz, 1H), 6.14 (dd, *J* = 15.8, 8.2 Hz, 1H), 4.97 (d, *J* = 8.2 Hz, 1H), 3.72 (s 3H), 3.29 (dqd, J = 8.3, 6.2, 3.8 Hz, 1H), 2.53 (dd, *J* = 8.3, 5.9 Hz, 2H), 2.07 (dtd, *J* = 13.6, 5.9, 3.8 Hz, 1H), 1.74 (dq, *J* = 13.6, 8.3 Hz, 1H), 1.15 (d, *J* = 6.2 Hz, 3H).

$^{13}C\{1H\}$ NMR (600 MHz, $CDCl_3$) δ 164.43, 163.99, 152.0, 142.5, 132.6, 122.62, 121.75, 114.3, 111.6, 109.4, 67.4, 51.71, 51.08, 28.75, 28.60, 16.0.

IR (neat, cm^{-1}): $\tilde{v}$= 2950, 1708, 1662, 1610, 1403, 1124, 728.

HRMS (ESI): *m*/*z* calcd for $C_{16}H_{19}N_2O_4$: 303.1339 $[M+H]^+$; found: 303.1323.

Compounds 2g/2g': 8-methyl-2-(pyrrolidine-1-carbonyl)-1-styryl-7,8-dihydro-1*H*-pyrazolo[1,2-*a*]pyridazin-5(6*H*)-one.

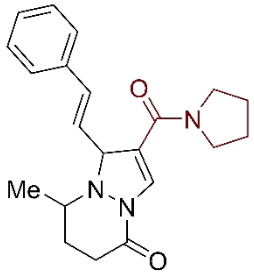

2g/2g'

Prepared according to the general procedure from **1a** (1.0 mmol), cinnamaldehyde (1.5 mmol) and 1-(pyrrolidin-1-yl)prop-2-yn-1-one (1.5 mmol). The reaction was carried out under argon in the dark at 50 °C (sand bath) for 48 h. The crude reaction mixture was separated by CC (DCM/MeOH = 10:1), affording two pure diastereoisomers, **2g** and **2g'**.

2g, major diastereoisomer, isolated as a yellow oil (126 mg, 36%, R_f = 0.55).

^{1}H NMR (600 MHz, $CDCl_3$) δ 7.40–7.38 (m, 2H), 7.31–7.28 (m, 3H), 7.23–7.20 (m, 1H), 6.70 (d, *J* = 15.8 Hz, 1H), 6.33 (dd, *J* = 15.8, 6.1 Hz, 1H), 5.25 (d, *J* = 6.1 Hz, 1H), 3.66–3.63 (m, 1H), 3.52–3.46 (m, 3H), 3.00 (dp, *J* = 8.8, 5.9 Hz, 1H), 2.64 (ddd, *J* = 15.7, 8.8, 6.8 Hz, 1H), 2.48 (ddd, *J* = 16.8, 7.7, 5.9 Hz, 1H), 2.00–1.80 (m, 4H), 1.96 (br sextet, *J* = 6.8 Hz, 1H), 1.83 (dtd, *J* = 14.5, 8.6, 6.0 Hz, 1H), 1.18 (d, *J* = 6.0 Hz, 3H).

^{13}C{1H} NMR (600 MHz, $CDCl_3$) δ 165.7, 162.5, 136.9, 131.4, 128.81, 128.57, 127.67, 126.72, 124.5, 117.5, 72.5, 57.7, 48.4, 46.6, 29.65, 28.99, 26.4, 24.1, 19.3.

IR (neat, cm^{-1}): $\tilde{v}$= 2973, 1619, 1450, 1382, 1353, 725.

HRMS (ESI): *m*/*z* calcd for $C_{21}H_{26}N_3O_2$: 352.2020 [*M*+H]$^+$; found: 352.2010.

2g', minor diastereoisomer, isolated as a yellow oil (32 mg, 9%, R_f = 0.53).

^{1}H NMR (600 MHz, $CDCl_3$) δ 7.43 (br s, 1H), 7.40–7.39 (m, 2H), 7.31–7.29 (m, 2H), 7.24–7.22 (m, 1H), 6.70 (d, *J* = 15.9 Hz, 1H), 6.25 (dd, *J* = 15.9, 7.9 Hz, 1H), 5.20 (d, *J* = 7.9 Hz, 1H), 3.64–3.61 (m, 1H), 3.52–3.48 (m, 3H), 3.31 (pd, *J*= 6.3, 4.0 Hz, 1H), 2.53 (dd, *J* = 8.0, 6.4 Hz, 2H), 2.10 (dtd, *J* = 13.5, 6.4, 4.0 Hz, 1H), 2.03–1.80 (m, 4H), 1.75 (dq, *J* = 13.5, 8.0 Hz, 1H), 1.15 (d, *J* = 6.3 Hz, 3H).

^{13}C{1H} NMR (600 MHz, $CDCl_3$) δ 164.3, 162.1, 136.5, 133.4, 128.66, 128.06, 126.91, 126.46, 124.9, 119.3, 69.7, 51.0, 48.3, 46.9, 28.41, 28.28, 26.6, 24.1, 15.1.

IR (neat, cm^{-1}): $\tilde{v}$= 2970, 1655, 1613, 1450, 1313, 1377, 726.

HRMS (ESI): *m*/*z* calcd for $C_{21}H_{26}N_3O_2$: 352.2020 [*M*+H]$^+$; found: 352.2021.

Compounds 2h/2h': Methyl 8-cyclohexyl-5-oxo-1-styryl-5,6,7,8-tetrahydro-1*H*-pyrazolo[1,2-*a*]pyridazine-2-carboxylate.

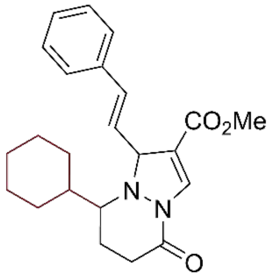

2h/2h'

Prepared according to the general procedure from **1b** (1.0 mmol), cinnamaldehyde (1.5 mmol) and methyl propiolate (1.2 mmol). The product was isolated as a mixture of diastereoisomers **2h**/**2h'** by CC (PE/EA = 5/1, R_f= 0.10) (244 mg, 64% yield, *d.r.* = 4:1).

^{13}C{1H} NMR (600 MHz, $CDCl_3$) δ 167.4, 164.42, 164.37, 163.93, 136.87, 136.23, 134.6, 132.8, 131.41, 131.37, 128.80, 128.68, 128.39, 127.83, 127.21, 126.88, 126.71, 123.0, 115.4, 112.4, 70.7, 66.93, 66.77, 59.5, 51.74, 51.68, 39.7, 38.4, 30.26, 30.22, 30.04, 29.92, 27.09, 26.84, 26.72, 26.68, 26.32, 25.65, 25.15, 23.97, 22.29, 21.41.

IR (neat, cm^{-1}): $\tilde{\nu}$ = 2923, 1711, 1684, 1616, 1413, 1245, 1212, 693.

HRMS (ESI): *m*/*z* calcd for $C_{23}H_{29}N_2O_3$: 381.2173 $[M+H]^+$; found: 381.2175.

Corresponding ^{1}H NMR resonances for each diastereoizomer:

2h (major):

^{1}H NMR (600 MHz, $CDCl_3$) δ 7.73 (s, 1H), 7.38–7.37 (m, 2H), 7.34–7.28 (m, 2H), 7.27–7.22 (m, 1H), 6.68 (d, *J* = 15.8 Hz, 1H), 6.22 (dd, *J* = 15.8, 7.0 Hz, 1H), 5.05 (d, *J* = 7.0 Hz, 1H), 3.72 (s, 3H), 2.92 (ddd, *J* = 8.5, 5.9, 3.0 Hz, 1H), 2.53 (ddd, *J* = 15.3, 11.3, 7.1 Hz, 1H), 2.41 (ddd, *J* = 15.3, 5.7, 4.0 Hz, 1H), 2.05 (dddd, *J* = 13.2, 7.1, 5.9, 4.0 Hz, 1H), 1.90–1.86 (m, 1H), 1.84–1.68 (m, 4H), 1.67–1.56 (m, 3H), 1.47 (d, *J* = 12.7 Hz, 1H), 1.25 (tt, *J* = 12.7, 3.5 Hz, 1H), 1.18–0.96 (m, 2H).

2h’ (major):

^{1}H NMR (600 MHz, $CDCl_3$) δ 7.84 (s, 1H), 7.38–7.37 (m, 2H), 7.34–7.28 (m, 2H), 7.27–7.22 (m, 1H), 6.69 (d, *J* = 15.8 Hz, 1H), 6.19 (dd, *J* = 15.8, 8.8 Hz, 1H), 5.01 (d, *J* = 8.8 Hz, 1H), 3.72 (s, 3H), 3.03 (dt, *J* = 10.4, 2.9 Hz, 1H), 2.56–2.51 (m, 1H), 2.48–2.43 (m, 1H), 1.90–1.86 (m, 1H), 1.84–0.88 (m, 12H).

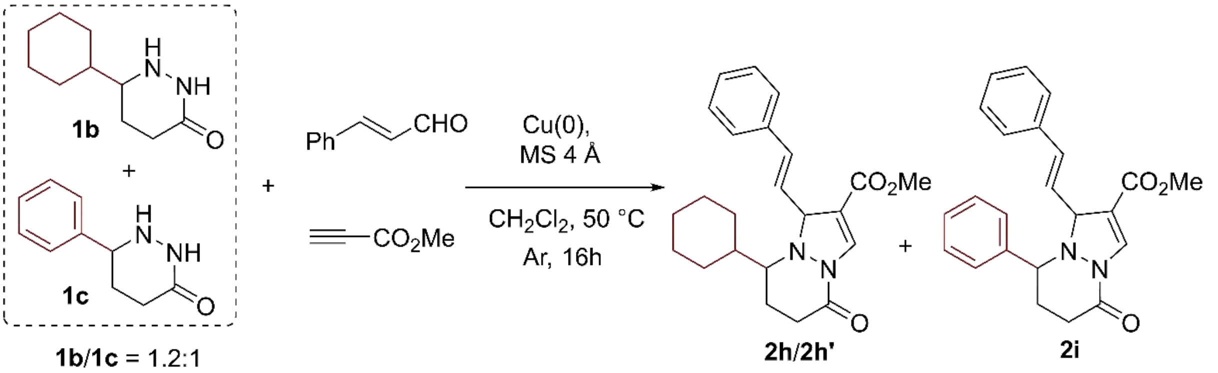

Scheme S5: Synthesis of compounds **2h**/**2h’** and **2i**.

Compound 2i: Methyl 5-oxo-8-phenyl-1-styryl-5,6,7,8-tetrahydro-1*H*-pyrazolo[1,2-*a*]pyridazine-2-carboxylate.

Prepared according to the general procedure from **1b**/**1c** (300 mg, **1b**/**1c** = 1.2:1), cinnamaldehyde (3.0 mmol) and methyl propiolate (2.4 mmol). The products were separated by preparative RP-CC (general purification procedure), affording pure **2i** (150 mg, 40% yield, calculated based on the starting concentration of **1c** in the reaction mixture) and a mixture of diastereoisomers **2h**/**2h’** (170 mg, *d.r.* = 4:1).

Compound 2i:

^{1}H NMR (600 MHz, $CDCl_3$) δ 7.80 (br s, 1H), 7.34–7.24 (m, 7H), 7.21–7.18 (m, 3H), 6.02 (d, *J* = 15.9 Hz, 1H), 5.89 (dd, *J* = 15.9, 6.8 Hz, 1H), 4.75 (d, *J* = 6.8 Hz, 1H), 3.90 (dd, *J* = 8.4, 7.2 Hz, 1H), 3.69 (s, 3H), 2.89 (ddd, *J* = 16.1, 13.1, 8.4 Hz, 1H), 2.58 (ddd, *J* = 16.1, 7.2, 4.1 Hz, 1H), 2.30 (dtd, *J* = 13.1, 8.0, 4.1 Hz, 1H), 2.21 (br sextet, *J* = 8.0 Hz, 1H).

^{13}C{1H} NMR (600 MHz, $CDCl_3$) δ 166.8, 164.3, 139.5, 136.8, 131.59 (2C), 131.38, 128.88, 128.72, 128.49, 127.67, 127.64, 126.71, 112.6, 70.0, 67.5, 51.7, 31.0, 29.8.

IR (neat, cm^{-1}): $\tilde{\nu}$ = 2943, 1705, 1679, 1620, 1414, 1324, 1225, 750.

HRMS (ESI): *m*/*z* calcd for $C_{23}H_{23}N_2O_3$: 375.1703 [*M*+H]$^+$; found: 375.1702.

Compounds 2j/2j'/2j'': Methyl 5-oxo-1-styryl-5,6,6a,7,8,9,10,10a-octahydro-1*H*-pyrazolo[1,2-*a*]cinnoline-2-carboxylate.

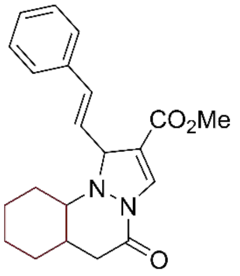

2j/2j'/2j''

Prepared according to the general procedure from **1d** (1.0 mmol), cinnamaldehyde (1.5 mmol) and methyl propiolate (1.2 mmol). The product was isolated as a mixture of diastereoisomers **2j**/**2j'**/**2j''** by preparative RP-CC (general purification procedure) (127 mg, 36% yield, *d.r.* = 2:2:1).

Due to similar concentrations of diastereoisomers in the racemic mixture **2j**/**2j'**/**2j''**, individual assignment of ^{1}H resonances was not possible.

^{1}H NMR (600 MHz, $CDCl_3$) δ 7.82, 7.79, and 7.77 (3d, 2:2:3, *J* = 1.1, 1.5, 1.7 Hz, 1H), 7.41–7.38 (m, 2H), 7.33–7.30 (m, 2H), 7.27–7.22 (m, 1H), 6.69, 6.68, and 6.67 (3d, 3:2:2, *J* = 16.0, 15.9, 15.7 Hz, 1H), 6.31, 6.25, and 6.23 (3dd, 3:2:2, *J* = 6.8, 15.9, 8.3 Hz, 1H), 5.16, 5.12, and 5.05 (3d, 3:2:2, *J* = 6.8, 7.8, 5.1 Hz, 1H), 3.71, 3.70, and 3.69 (3s, 2:3:2, 3H), 3.23–1.03 (m, 12H).

^{13}C{1H} NMR (600 MHz, $CDCl_3$) δ 164.23, 164.20, 164.17, 164.08, 163.86, 163.66, 136.82, 136.80, 136.28, 133.7, 132.40, 132.29, 132.24, 132.21, 131.91, 128.95, 128.74, 128.70, 128.69, 128.57, 128.25, 127.90, 127.87, 127.24, 126.90, 126.74, 124.50, 114.6, 113.40, 113.10, 71.7, 70.1, 66.72, 65.85, 61.1, 54.5, 51.68, 51.62, 51.60, 38.4, 36.61, 36.47, 35.37, 34.56, 33.77, 31.9, 29.73, 28.99, 28.61, 27.74, 25.38, 25.19, 24.55, 24.23, 23.30, 21.6, 19.7.

IR (neat, cm^{-1}): $\tilde{\nu}$ = 2931, 1708, 1662, 1415, 1404, 1217, 1129, 693.

HRMS (ESI): *m*/*z* calcd for $C_{21}H_{25}N_2O_3$: 353.1860 [*M*+H]$^+$; found: 353.1854.

Compounds 2k/2k': Methyl 8-methyl-1-(2-methylprop-1-en-1-yl)-5-oxo-5,6,7,8-tetrahydro-1H-pyrazolo[1,2-a]pyridazine-2-carboxylate.

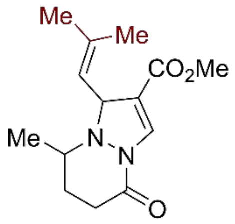

2k/2k'

Prepared according to the general procedure from **1a** (1.0 mmol), 3-methyl-2-butenal (1.2 mmol) and methyl propiolate (1.2 mmol) in CH_2Cl_2 (5 mL). Two diastereoisomers **2k**/**2k'** were formed (*d.r.* = 4:1, determined from the crude reaction mixture), of which the major diastereoisomer **2k** was isolated by preparative RP-CC (general purification procedure) as a yellow oil (158 mg, 60% yield).

^{1}H NMR (500 MHz, $CDCl_3$) δ 7.69 (br s, 1H), 5.09–5.05 (m, 1H), 5.07 (d, *J* = 6.8 Hz, 1H), 3.69 (s, 3H), 2.99–2.94 (m, 1H), 2.56 (ddd, *J* = 17.1, 8.7, 6.8 Hz, 1H), 2.42 (ddd, *J* = 16.9, 7.6, 6.2 Hz, 1H), 1.94–1.88 (m, 1H), 1.79–1.72 (m, 1H), 1.78 (s, 3H), 1.71 (s, 3H), 1.13 (d, *J* = 6.3 Hz, 3H).

^{13}C{1H} NMR (500 MHz, $CDCl_3$) δ 165.8, 164.4, 134.3, 131.5, 124.1, 113.2, 67.4, 57.6, 51.5, 29.69, 28.98, 26.1, 19.07, 18.30.

IR (neat, cm^{-1}): $\tilde{\nu}$ = 2977, 1712, 1618, 1406, 1245, 1217, 1118, 734.

HRMS (ESI): *m*/*z* calcd for $C_{14}H_{21}N_2O_3$: 265.1547 [*M*+H]$^+$; found: 265.1548.

Compounds 2l/2l': Methyl 1-(tert-butyl)-8-methyl-5-oxo-5,6,7,8-tetrahydro-1H-pyrazolo[1,2-a]pyridazine-2-carboxylate.

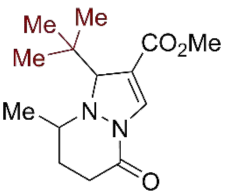

2l/2l'

Prepared according to the general procedure from **1a** (1.0 mmol), pivaldehyde (1.2 mmol) and methyl propiolate (1.2 mmol). The product was isolated as a mixture of diastereoisomers **2l/2l'** by CC (PE/EA = 5:2) (131 mg, 25% yield, *d.r.* = 2:1), and the diastereoisomers were further separated by preparative RP-CC (general purification procedure). Alongside, **5a/5a'** was isolated in 38% yield (for characterization of **5a/5a'**, see Section 10.3).

Note: **2l'** was isolated containing traces of **2l**.

2l, major diastereoisomer, isolated as a yellow oil (40 mg, 15%).

^{1}H NMR (600 MHz, $CDCl_3$) δ 7.80 (br s, 1H), 3.99 (d, *J* = 0.8 Hz, 1H), 3.73 (s, 3H), 3.23–3.18 (m, 1H), 2.58 (ddd, *J* = 15.4, 10.4, 8.6 Hz, 1H), 2.46–2.40 (m, 1H), 2.38 (dt, *J* = 7.6, 3.3 Hz, 1H), 1.48–1.43 (m, 1H), 0.92 (d, *J* = 6.5 Hz, 3H), 0.86 (s, 9H).

^{13}C{1H} NMR (600 MHz, $CDCl_3$) δ 166.7, 165.6, 134.4, 112.7, 75.0, 56.7, 51.6, 37.6, 28.5, 26.8, 25.7, 13.0.

IR (neat, cm^{-1}): $\tilde{\nu}$ = 2953, 1712, 1679, 1603, 1411, 1188, 1120, 757.

HRMS (ESI): *m*/*z* calcd for $C_{14}H_{23}N_2O_3$: 267.1703 [*M*+H]$^+$; found: 267.1711.

2l', minor diastereoisomer, isolated as a yellow oil (21 mg, 8%).

^{1}H NMR (600 MHz, $CDCl_3$) δ 7.75 (br s, 1H), 4.04 (s, 1H), 3.73 (s, 3H), 2.73–2.63 (m, 2H), 2.34 (ddd, *J* = 14.5, 7.0, 1.7 Hz, 1H), 1.97–1.90 (m, 1H), 1.81 (dddd, *J* = 13.4, 9.6, 7.8, 1.7 Hz, 1H), 1.18 (d, *J* = 6.3 Hz, 3H), 0.84 (s, 9H).

^{13}C{1H} NMR (600 MHz, $CDCl_3$) δ 168.7, 165.9, 132.9, 111.7, 73.3, 56.3, 51.7, 36.9, 30.0, 29.4, 26.0, 19.7.

IR (neat, cm^{-1}): $\tilde{\nu}$ = 2952, 1687, 1608, 1410, 1184, 1115, 749.

HRMS (ESI): *m*/*z* calcd for $C_{14}H_{23}N_2O_3$: 267.1703 [*M*+H]$^+$; found: 267.1706.

Compounds 2m/2m': Methyl 8-methyl-5-oxo-1-(pent-1-en-1-yl)-5,6,7,8-tetrahydro-1H-pyrazolo[1,2-a]pyridazine-2-carboxylate.

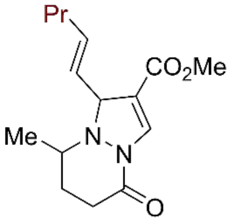

2m/2m'

Prepared according to the general procedure from **1a** (1.0 mmol), trans-2-hexen-1-al (1.2 mmol) and methyl propiolate (1.2 mmol). Two diastereoisomers **2m/2m'** were formed (*d.r.* = 10:1, determined from the crude reaction mixture). Upon preparative RP-CC purification (general procedure), the minor diastereoisomer was isolated as part of a **2m/2m'** mixture (14 mg, 5% yield, *d.r.* = 1:9). During purification, the remaining **2m** underwent oxidation to give *E*-**2m-OH**, which was isolated as a yellow oil (68 mg, 23% yield). For characterization of *E*-**2m-OH**, see Section 9.2.

2m/2m' (*d.r.* = 1:9).

IR (neat, cm^{-1}): $\tilde{v}$ = 2957, 1711, 1612, 1408, 1220, 1125, 777.

HRMS (ESI): *m*/*z* calcd for $C_{15}H_{23}N_2O_3$: 279.1703 [*M*+H]$^+$; found: 279.1712.

Corresponding ^{1}H and ^{13}C{1H} NMR resonances for each diastereoizomer:

2m:

^{1}H NMR (600 MHz, $CDCl_3$) δ 7.69 (d, *J* = 1.5 Hz, 1H), 2.57–2.70 (m, 1H), 5.49 (ddt, *J* = 15.3, 6.7, 1.5 Hz, 1H), 4.80 (d, *J* = 5.6 Hz, 1H), 3.71 (s, 3H), 2.98 (dp, *J* = 8.4, 6.2 Hz, 1H), 2.61–2.56 (m, 1H), 2.51–2.41 (m, 2H), 2.06–2.00 (m, 2H), 1.93 (dtd, *J* = 13.5, 7.5, 5.9 Hz, 1H), 1.81–1.70 (m, 2H), 1.16 (d, *J* = 6.3 Hz, 3H), 0.98–0.92 (m, 3H).

^{13}C{1H} NMR (600 MHz, $CDCl_3$) δ 166.1, 164.2, 133.4, 131.2, 129.2, 113.1, 70.6, 57.8, 51.6, 34.3, 29.67, 29.04, 22.3, 19.5, 13.7.

2m':

^{1}H NMR (600 MHz, $CDCl_3$) δ 7.76 (br s, 1H), 5.77 (dt, *J* = 15.3, 6.8 Hz, 1H), 5.43 (ddt, *J* = 15.3, 8.5, 1.5 Hz, 1H), 4.80 (d, *J* = 8.5 Hz, 1H), 3.71 (s, 3H), 3.25–3.20 (m, 1H), 2.51–2.48 (m, 2H), 2.05–2.00 (m, 3H), 1.74 (dtd, *J* = 13.6, 9.2, 7.9 Hz, 1H), 1.40 (dd, *J* = 7.3, 7.3 Hz, 2H), 1.13 (d, *J* = 6.3 Hz, 3H), 0.88 (t, *J* = 7.4 Hz, 3H).

^{13}C{1H} NMR (600 MHz, $CDCl_3$) δ 164.24, 164.07, 136.1, 132.4, 124.7, 115.1, 67.8, 51.6, 50.6, 34.4, 28.76, 28.66, 22.2, 16.0, 13.7.

Compounds 2n/2n': Methyl 1-(cyclohex-1-en-1-yl)-8-methyl-5-oxo-5,6,7,8-tetrahydro-1H-pyrazolo[1,2-a]pyridazine-2-carboxylate.

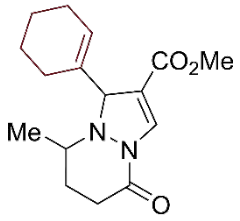

2n/2n'

Prepared according to the modified general procedure. An Ace pressure tube was charged with **1a** (2.0 mmol), 1-cyclohexene-1-carboxaldehyde (4.8 mmol), methyl propiolate (4.0 mmol), CH_2Cl_2 (10.0 mL), copper(0) (1.4 mmol), and molecular sieves (4 Å; 200 mg). The reaction mixture was purged with argon and was reacted in the dark at 50 °C (heating mantle) for 48 h. Two diastereoisomers **2n**/**2n'** were formed (*d.r.* = 2:1, determined from the crude reaction mixture), of which the major diastereoisomer **2n** was isolated by preparative RP-CC (general purification procedure) as a yellow oil (145 mg, 25% yield). Alongside, **5a**/**5a'** was isolated in 48% yield (for characterization of **5a**/**5a'**, see Section 10.3).

Compound **2n**:

^{1}H NMR (500 MHz, $CDCl_3$) δ 7.73 (br s, 1H), 5.70–5.68 (m, 1H), 4.76 (s, 1H), 3.70 (s, 3H), 2.96 (dt, *J* = 8.1, 6.2 Hz, 1H), 2.57 (dt, *J* = 16.3, 8.2 Hz, 1H), 2.41 (ddd, *J* = 16.5, 7.3, 5.4 Hz, 1H), 2.13–2.00 (m, 3H), 1.96–1.87 (m, 1H), 1.79–1.72 (m, 2H), 1-63–1-51 (m, 4H), 1.16 (d, *J* = 6.3 Hz, 3H).

^{13}C{1H} NMR (500 MHz, $CDCl_3$) δ 166.3, 164.4, 136.6, 131.6, 125.8, 112.3, 75.1, 57.9, 51.5, 29.82, 29.07, 25.4, 24.2, 22.71, 22.55, 19.4.

IR (neat, cm^{-1}): $\tilde{v}$ = 2929, 1712, 1683, 1616, 1406, 1243, 1119, 755.

HRMS (ESI): *m*/*z* calcd for $C_{16}H_{23}N_2O_3$: 291.1703 [*M*+H]$^+$; found: 291.1704.

6. Synthesis and characterization of 1,2-diazepine 3a

Compound 3a: Methyl 4-methyl-1-oxo-6-phenyl-1,2,3,4-tetrahydro-6*H*-pyridazino[1,2-*a*][1,2]diazepine-9-carboxylate

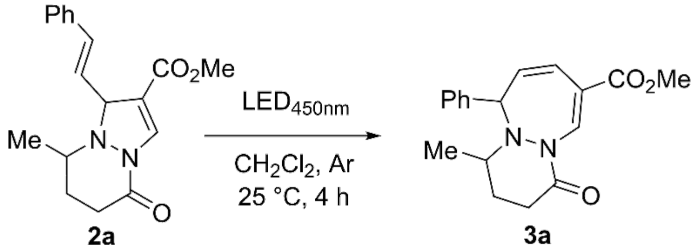

Scheme S6: Synthesis of 1,2-diazepine **3a**.

Both diastereoisomers **2a** and **2a'** afforded the same product **3a** under the reaction conditions, allowing the use of either isomer as the starting material. During optimizations studies, the reaction rate was found to be dependent on the concentration of **2a**. A more dilute solution (0.05 M) rapidly led to the formation of the tricyclic product **4a.** In contrast, the more concentrated 0.15 M solution progressed more slowly and was therefore adopted for the preparation of 1,2-diazepine **3a**.

Procedure: a vial was charged with **2a** (1.2 mmol) and CH_2Cl_2 (8.0 mL; 0.15 M), was sealed off with a screw cap equipped with a septum, and the resulting solution was purged with argon. The mixture was then irradiated with LED_{450nm} for 4 h at 25 °C. The crude reaction mixture was purified by CC (PE/EA = 5/3, R_f = 0.28), affording **3a** as a yellow oil (41 mg, 11% yield).

^{1}H NMR (600 MHz, $CDCl_3$) δ 7.82 (d, *J* = 1.4 Hz, 1H), 7.57–7.55 (m, 2H), 7.38–7.34 (m, 2H), 7.30–7.27 (m, 1H), 6.02 (d, *J* = 11.3 Hz, 1H), 5.63 (dd, *J* = 11.3, 10.0 Hz, 1H), 5.43 (d, *J* = 10.0 Hz, 1H), 3.77 (s, 3H), 2.81 (dp, *J* = 9.0, 6.3 Hz, 1H), 2.53 (ddd, *J* = 17.1, 8.6, 6.7 Hz, 1H), 2.40 (ddd, *J* = 17.1, 7.7, 6.3 Hz, 1H), 1.82 (dddd, *J* = 13.1, 7.7, 6.7, 5.4 Hz, 1H), 1.68 (dtd, *J* = 13.1, 8.6, 6.4 Hz, 1H), 0.71 (d, *J* = 6.4 Hz, 3H).

^{13}C{1H} NMR (600 MHz, $CDCl_3$) δ 165.7, 164.5, 136.6, 133.0, 130.78, 130.52, 129.06, 128.45, 127.49, 112.0, 65.9, 57.8, 51.7, 29.66, 28.98, 18.7.

IR (neat, cm^{-1}): $\tilde{v}$= 2949, 1709, 1672, 1615, 1405, 1216, 1123, 700.

HRMS (ESI): *m*/*z* calcd for $C_{18}H_{21}N_2O_3$: 313.1547 [*M*+H]$^+$; found: 313.1538.

7. Optimization studies for the synthesis of 4a

7.1 Optimization of the reaction conditions

The reaction was performed under standard reaction conditions (*vide infra*)

Table S2: ^{1}H NMR yields of **4a**.

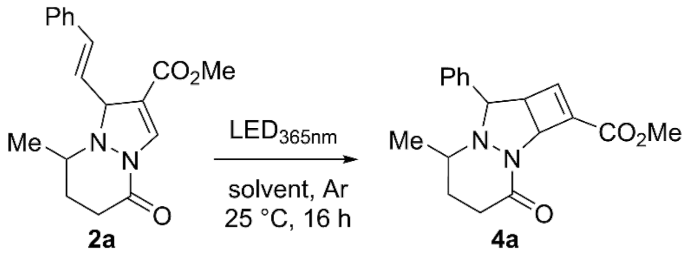

Entry	Solvent	NMR yield 4a [%]
**1**	DCM	85
**2**	THF	86
**3**	EtOAc	64
**4**	MeOH	50
**5**	MeCN	45
**6**	Toluene	39
**7**	CHCl3	n.d.
**8**	DCM + water[a]	82

[a]To 1.0 mL of DCM 50 µL of water was added. n.d. = not detected.

Standard reaction procedure: a vial was charged with **2a** (0.05 mmol) and solvent (1.0 mL), was sealed off with a screw cap equipped with a septum, and the resulting solution was purged with argon. The mixture was then irradiated with LED_{365nm} for 16 h at 25 °C. NMR yields were determined with 1,3,5-trimethoxybenzene as an internal standard.

Testing for water sensitivity (entry 8): before purging with argon 50 µL of water was added to the reaction mixture (0.05 mmol of **2a**, 1.0 mL of DCM). No major difference was observed compared to the standard reaction conditions (entry 1), which indicated that the presence of water does not influence the reaction performance.

In the presence of air an oxidized product *Z*-**2a-OH** was formed instead of **4a**, which was isolated and characterized (see Section 9.2).

7.2 Air sensitivity test of **4a**

A vial was charged with 20 mg of **4a**/**4a'**/**4a''** mixture and was stored in the dark under air at 25 °C for 6 months. After this period, a 1H NMR spectrum was recorded, revealing no changes in the product composition. This indicates that the compounds are stable under ambient conditions and are not susceptible to oxidation by atmospheric oxygen.

8. Synthesis and characterization of tricyclic products 4

8.1 General procedure for the synthesis of compounds **4**

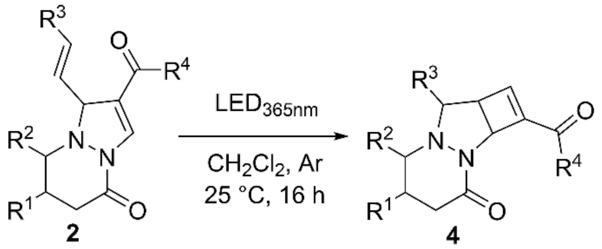

Scheme S7: Synthesis of tricyclic products **4**.

A vial was charged with **2** (0.2 mmol) and CH_2Cl_2 (4.0 mL; 0.05 M), was sealed off with a screw cap equipped with a septum, and the resulting solution was purged with argon. The mixture was then irradiated with LED_{365nm} for 16 h at 25 °C. Products **4** were obtained by radial chromatography (PE/EA=5:3) or preparative RP-CC (isocratic hold at 80:20 H_2O/ MeCN for 10 min, then gradient to 40:60 over 40 min (total runtime: 50 minutes)).

8.2 Characterization data of compounds **4**

Compounds 4a/4a'/4a'': Methyl 7-methyl-4-oxo-9-phenyl-2a,4,5,6,7,9a-hexahydro-9*H*-cyclobuta[3,4]pyrazolo[1,2-*a*]pyridazine-2-carboxylate.

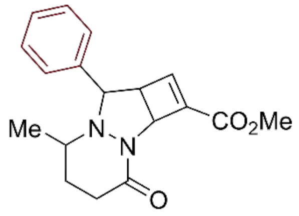

4a/4a'/4a''

Prepared according to the general procedure from **2a** (1.2 mmol). The product was isolated as a mixture of diastereoisomers **4a/4a'/4a''** by preparative RP-CC (general purification procedure) (251 mg, 67% yield, *d.r.* = 3:2:1). The major diastereoisomers **4a** and **4a'** were further isolated by CC (PE/EA = 1/1)).

4a, isolated as a colorless oil (109 mg, 29%).

^{1}H NMR (600 MHz, $CDCl_3$) δ 7.35–7.31 (m, 3H), 7.07–7.05 (m, 2H), 7.02–7.02 (m, 1H), 5.81 (dd, *J* = 3.8, 1.8 Hz, 1H), 4.24 (s, 1H), 3.77 (s, 3H), 3.53 (d, *J* = 3.8 Hz, 1H), 2.42–2.36 (m, 2H), 2.24 (ddd, *J* =17.1, 12.6, 6.1 Hz, 1H), 1.77–1.71 (m, 1H), 1.63 (ddt, *J* = 13.4, 6.1, 2.4 Hz, 1H), 1.09 (d, *J* = 6.1 Hz, 3H).

^{13}C{1H} NMR (600 MHz, $CDCl_3$) δ 164.3, 161.6, 148.0, 138.45, 138.35, 128.98, 128.27, 128.11, 64.4, 57.3, 51.96, 51.27, 51.00, 30.1, 29.0, 18.9.

IR (neat, cm^{-1}): $\tilde{v}$= 3043, 1715, 1632, 1435, 1187, 1128, 760.

HRMS (ESI): *m*/*z* calcd for $C_{18}H_{21}N_2O_3$: 313.1547 [*M*+H]$^+$; found: 313.1544.

4a', isolated as a colorless oil (82 mg, 22%).

^{1}H NMR (600 MHz, $CDCl_3$) δ 7.33–7.31 (m, 2H), 7.29–7.27 (m, 1H), 7.17–7.16 (m, 2H), 7.04–7.03 (m, 1H), 5.56 (br p, *J* = 1.9 Hz, 1H), 4.08 (t, *J* = 2.0 Hz, 1H), 3.78 (d, *J* = 1.5 Hz, 3H), 3.46 (dt, *J* = 3.9, 2.5 Hz, 1H), 3.39–3.36 (m, 1H), 2.41–2.34 (m, 2H), 1.53–1.48 (m, 1H), 1.11 (br dd, *J* = 6.7, 1.6 Hz, 3H), 1.09–1.04 (m, 1H).

$^{13}C\{1H\}$ NMR (600 MHz, $CDCl_3$) δ 167.5, 162.1, 149.0, 141.6, 140.3, 129.03, 128.03, 127.56, 64.9, 57.9, 54.7, 53.4, 52.0, 30.0, 27.0, 19.1.

IR (neat, cm^{-1}): $\tilde{v}$ = 3043, 1722, 1628, 1440, 1412, 1137, 766.

HRMS (ESI): *m*/*z* calcd for $C_{18}H_{21}N_2O_3$: 313.1547 [*M*+H]$^+$; found: 313.1547.

4a''.

Corresponding ^1H and $^{13}C\{1H\}$ NMR resonances were extracted from the spectra of the diastereoisomeric mixture **4a**/**4a'**/**4a''** (1.2:1).

^1H NMR (600 MHz, $CDCl_3$) δ 7.34–7.28 (m, 4H), 7.17–7.01 (m, 1H), 6.58 (br s, 1H), 5.69 (dd, *J* = 3.5, 1.4 Hz, 1H), 3.77 (s, 3H), 3.68 (br d, *J* = 7.1 Hz, 1H), 3.56 (br dd, *J* = 7.1, 3.5 Hz, 1H), 2.91 (br pd, *J* = 6.6, 3.1 Hz, 1H), 2.53 (dt, *J* = 18.0, 6.4 Hz, 1H), 2.46 (ddd, *J* = 18.0, 8.3, 6.4 Hz, 1H), 1.83–1.70 (m, 2H), 0.86 (d, *J* = 6.6 Hz, 3H).

$^{13}C\{1H\}$ NMR (600 MHz, $CDCl_3$) δ 165.0, 147.6, 139.7, 137.7, 128.91, 128.67, 128.09, 127.77, 127.19, 70.1, 56.1, 55.0, 50.0, 28.6, 26.4, 20.0.

Compounds 4b/4b'/4b'': Methyl 9-(4-fluorophenyl)-7-methyl-4-oxo-2a,4,5,6,7,9a-hexahydro-9*H*-cyclobuta[3,4]pyrazolo[1,2-*a*]pyridazine-2-carboxylate.

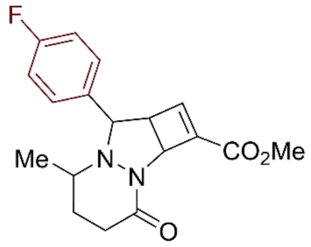

4b/4b'/4b''

Prepared according to the general procedure from **2b** (0.4 mmol). Three diastereoisomers **4b**/**4b'**/**4b''** were formed (*d.r.* = 3:3:1, determined from the crude reaction mixture). Diastereoisomers **4b** and **4b'** were isolated in pure form by preparative RP-CC (general purification procedure), while **4b''** could only be obtained in low mass and with traces of impurities.

Note: For all diastereoisomers **4b**/**4b'**/**4b''** two additional ^{13}C resonances are observed beyond the expected number.

4b, isolated as a colorless oil (38 mg, 29% yield).

^1H NMR (600 MHz, $CDCl_3$) δ 7.03–6.99 (m, 5H), 5.77 (dd, *J* = 3.7, 1.8 Hz, 1H), 4.23 (s, 1H), 3.76 (s, 3H), 3.49 (d, *J* = 3.7 Hz, 1H), 2.37 (dd, *J* = 17.7, 1.8 Hz, 1H), 2.34 (dqd, *J* = 13.0, 6.0, 2.7 Hz, 1H), 2.23 (ddd, *J* = 17.7, 12.7, 6.3 Hz, 1H), 1.74 (tdd, *J* = 12.7, 11.3, 5.1 Hz, 1H), 1.63 (ddt, *J* = 13.0, 6.3, 2.7 Hz, 1H), 1.07 (d, *J* = 6.0 Hz, 3H).

$^{13}C\{1H\}$ NMR (600 MHz, $CDCl_3$) δ 164.4, 163.4, 161.8, 161.5, 147.8, 138.5, 134.2, 129.75, 129.70, 116.04, 115.90, 63.6, 57.2, 52.01, 51.27, 50.98, 30.1, 29.0, 18.8.

IR (neat, cm^{-1}): $\tilde{v}$ = 2949, 1720, 1626, 1601, 1413, 1221, 764.

HRMS (ESI): *m*/*z* calcd for $C_{18}H_{20}FN_2O_3$: 331.1452 [*M*+H]$^+$; found: 331.1454.

4b', isolated as a colorless oil (31 mg, 24% yield).

^1H NMR (600 MHz, $CDCl_3$) δ 7.16–7.13 (m, 2H), 7.03–7.00 (m, 3H), 5.54 (dd, *J* = 4.0, 1.9 Hz, 1H), 4.08 (d, *J* = 2.4 Hz, 1H), 3.78 (s, 3H), 3.42 (br t, *J* = 3.0 Hz, 1H), 3.37 (dqd, *J* = 10.4, 6.7, 4.0 Hz, 1H), 2.43–2.33 (m, 2H), 1.57–1.52 (m, 1H), 1.14–1.09 (m, 1H), 1.09 (d, *J* = 6.7 Hz, 3H).

$^{13}C\{1H\}$ NMR (600 MHz, $CDCl_3$) δ 167.5, 163.2, 162.05, 161.59, 148.8, 140.5, 137.47, 137.45, 129.09, 129.04, 116.02, 115.88, 64.3, 57.9, 54.8, 53.4, 52.0, 30.0, 27.2, 19.2.

IR (neat, cm^{-1}): $\tilde{v}$= 2920, 1718, 1632, 1599, 1228, 762.

HRMS (ESI): *m*/*z* calcd for $C_{18}H_{20}FN_2O_3$: 331.1452 [*M*+H]$^+$; found: 331.1448.

4b'', isolated as a colorless oil (9 mg, 8% yield).

^1H NMR (600 MHz, $CDCl_3$) δ 7.31–7.29 (m, 2H), 7.05–7.02 (m, 2H), 6.56 (br s, 1H), 5.69 (dd, *J* = 3.8, 1.5 Hz, 1H), 3.78 (s, 3H), 3.68 (d, *J* = 7.0 Hz, 1H), 3.56 (ddd, *J* = 7.0, 3.8, 1.0 Hz, 1H), 2.92–2.87 (m, 1H), 2.53 (dt, *J* = 17.9, 6.4 Hz, 1H), 2.46 (ddd, *J* = 17.9, 8.2, 6.5 Hz, 1H), 1.83–1.78 (m, 1H), 1.78–1.73 (m, 1H), 0.86 (d, *J* = 6.5 Hz, 3H).

$^{13}C\{1H\}$ NMR (600 MHz, $CDCl_3$) δ 165.0, 163.4, 161.76, 161.48, 147.2, 140.2, 133.47, 133.45, 129.31, 129.25, 115.8, 115.6, 69.3, 56.1, 55.1, 52.0, 49.9, 28.6, 26.6, 20.1.

IR (neat, cm^{-1}): $\tilde{v}$= 2951, 1719, 1630, 1508, 1217, 728.

HRMS (ESI): *m*/*z* calcd for $C_{18}H_{20}FN_2O_3$: 331.1452 [*M*+H]$^+$; found: 331.1452.

Compounds 4c/4c'/4c'': Methyl 9-(4-(dimethylamino)phenyl)-7-methyl-4-oxo-2a,4,5,6,7,9a-hexahydro-9*H*-cyclobuta[3,4]pyrazolo[1,2-*a*]pyridazine-2-carboxylate.

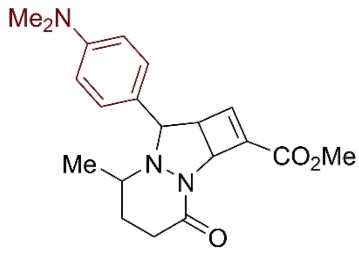

4c/4c'/4c''

Prepared according to the general procedure from **2c** (0.2 mmol). Three diastereoisomers **4c/4c'/4c''** were formed (*d.r.* = 2.5:2:1, determined from the crude reaction mixture). A mixture of the major diastereoisomers **4c/4c'** was isolated by preparative RP-CC (general purification procedure) (28 mg, 40% yield, *d.r.* = 3:1), and diastereoisomer **4c** was further isolated by radial chromatography (PE/EA=1:3, R_f = 0.10) (20mg, 28% yield).

4c, major diastereoisomer, isolated as a colorless oil.

^1H NMR (600 MHz, $CDCl_3$) δ 7.02–7.02 (m, 1H), 6.93–6.91 (m, 2H), 6.66–6.65 (m, 2H), 5.78 (dd, *J* = 3.8, 2.3 Hz, 1H), 4.15 (s, 1H), 3.77 (s, 3H), 3.50 (d, *J* = 3.8 Hz, 1H), 2.94 (s, 6H), 2.39–2.36 (m, 1H), 2.24 (ddd, *J* = 17.5, 12.0, 6.1 Hz, 1H), 1.73 (tdd, *J* = 13.0, 12.0, 5.1 Hz, 2H), 1.61 (ddt, *J* = 13.0, 6.1, 2.3 Hz, 1H), 1.09 (d, *J* = 6.1 Hz, 3H).

$^{13}C\{1H\}$ NMR (600 MHz, $CDCl_3$) δ 164.4, 161.7, 150.2, 148.5, 138.2, 129.0, 126.53, 125.56, 112.48, 112.36, 63.9, 57.3, 51.92, 51.14, 40.5, 30.2, 29.1, 18.9.

IR (neat, cm^{-1}): $\tilde{v}$= 2927, 1718, 1608, 1441, 1150, 799.

HRMS (ESI): *m*/*z* calcd for $C_{20}H_{26}N_3O_3$: 356.1969 [*M*+H]$^+$; found: 356.1964.

4c'.

Corresponding ^1H and $^{13}C\{1H\}$ NMR resonances were extracted from the spectra of the diastereoisomeric mixture **4c/4c'** (3:1).

^{1}H NMR (600 MHz, $CDCl_3$) δ 7.17–7.15 (m, 2H), 6.70–6.65 (m, 3H), 5.67 (dd, *J* = 3.8, 1.4 Hz, 1H), 3.78 (s, 3H), 3.57 (d, *J* = 6.9 Hz, 1H), 2.96 (s, 6H), 2.52–2.36 (m, 6H), 0.89 (d, *J* = 6.6 Hz, 3H).

^{13}C{1H} NMR (600 MHz, $CDCl_3$) δ 165.0, 163.9, 150.4, 148.46, 148.28, 139.4, 128.73, 124.81, 118.3, 69.8, 55.8, 54.2, 51.9, 50.2, 40.6, 28.5, 26.1, 20.0.

Compounds 4d/4d/4d'': Methyl 9-(2-methoxyphenyl)-7-methyl-4-oxo-2a,4,5,6,7,9a-hexahydro-9*H*-cyclobuta[3,4]pyrazolo[1,2-*a*]pyridazine-2-carboxylate.

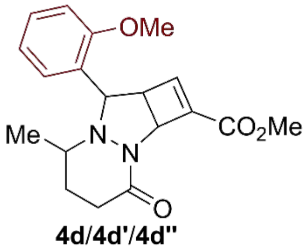

4d/4d'/4d"

Prepared according to the general procedure from **2d** (0.2 mmol). Three diastereoisomers **4d/4d'/4d''** were formed (*d.r.* = 6:4:1, determined from the crude reaction mixture), of which the two major diastereoisomers (**4d** and **4d'**) were isolated by RP-CC (general purification procedure).

4d, isolated as a colorless oil (23 mg, 34% yield).

^{1}H NMR (600 MHz, $CDCl_3$) δ 7.27–7.24 (m, 1H), 7.02 (br s, 1H), 6.92–6.88 (m, 3H), 5.76 (dd, *J* = 3.7, 1.6 Hz, 1H), 4.86 (br s, 1H), 3.81 (s, 3H), 3.76 (s, 3H), 3.46 (d, *J* = 3.7 Hz, 1H), 2.44 (dtt, *J* = 12.0, 6.0, 2.5 Hz, 1H), 2.38 (ddd, *J* = 17.7, 4.8, 2.5 Hz, 1H), 2.20 (ddd, *J* = 17.7, 12.5, 6.1 Hz, 1H), 1.72 (ddd, *J* = 24.4, 12.5, 4.8 Hz Hz, 1H), 1.64–1.61 (m, 1H), 1.08 (d, *J* = 6.1 Hz, 3H).

^{13}C{1H} NMR (600 MHz, $CDCl_3$) δ 164.4, 161.7, 157.1, 148.5, 138.3 (2C), 129.1, 126.8, 121.2, 110.7, 57.4 (2C), 55.5, 51.88, 51.49, 51.06, 30.08, 29.15, 19.0.

IR (neat, cm^{-1}): $\tilde{v}$= 2950, 1722, 1623, 1490, 1238, 725.

HRMS (ESI): *m*/*z* calcd for $C_{19}H_{23}N_2O_4$: 343.1652 [*M*+H]$^+$; found: 343.1653.

4d', isolated as a colorless oil (22 mg, 33% yield).

^{1}H NMR (600 MHz, $CDCl_3$) δ 7.32–7.30 (m, 1H), 7.25–7.23 (m, 1H), 7.12 (br s, 1H), 6.94–6.92 (m, 1H), 6.87–6.85 (m, 1H), 5.40 (dd, *J* = 3.8, 1.6 Hz, 1H), 4.55 (d, *J* = 2.41 Hz, 1H), 3.85 (s, 3H), 3.78 (s, 3H), 3.46–3.45 (m, 1H), 3.41–3.36 (m, 1H), 2.52 (ddd, *J* = 16.2, 7.5, 6.3 Hz, 7.5 Hz, 1H), 2.40 (ddd, *J* = 16.2, 7.5, 6.0 Hz, 1H), 1.78–1.72 (m, 1H), 1.50–1.44 (m, 1H), 1.02 (d, *J* = 6.6 Hz, 3H).

^{13}C{1H} NMR (600 MHz, $CDCl_3$) δ 168.5, 162.3, 156.0, 150.7, 141.7, 130.6, 128.61, 127.72, 120.9, 110.4, 59.02, 58.87, 56.68, 55.49, 53.9, 52.0, 30.4, 29.3, 19.9.

IR (neat, cm^{-1}): $\tilde{v}$= 2948, 1719, 1639, 1489, 1239, 729.

HRMS (ESI): *m*/*z* calcd for $C_{19}H_{23}N_2O_4$: 343.1652 [*M*+H]$^+$; found: 343.1650.

Compounds 4e/4e': Methyl 9-(anthracen-9-yl)-7-methyl-4-oxo-2a,4,5,6,7,9a-hexahydro-9*H*-cyclobuta[3,4]pyrazolo[1,2-*a*]pyridazine-2-carboxylate.

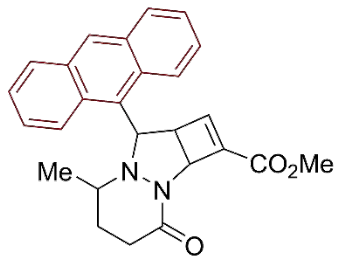

4e/4e'

Prepared according to the general procedure from **2e**/**2e'** (0.2 mmol, *d.r.* = 1.3:1). The product was isolated as a mixture of diastereoisomers **4e**/**4e'** by radial chromatography (PE/EA=1:1, R_f = 0.10) (74 mg, 90% yield, *d.r.* = 1.3:1).

$^{13}C\{1H\}$ NMR (600 MHz, $CDCl_3$) δ 167.0, 165.7, 162.35, 161.75, 150.68, 149.49, 143.8, 139.1, 132.15, 132.12, 131.52, 131.48, 131.17, 131.13, 130.20, 130.03, 129.99, 129.49, 129.25, 129.12, 128.87, 128.83, 127.87, 127.19, 127.03, 126.90, 126.59, 125.79, 125.40, 125.36, 125.06, 124.92, 122.52, 122.42, 67.3, 61.8, 59.5, 56.4, 55.2, 52.15, 51.99, 50.07, 49.72, 49.40, 41.0, 28.65, 28.49, 27.43, 26.1, 24.0, 18.5, 11.8.

IR (neat, cm^{-1}): $\tilde{v}$= 2948, 1721, 1633, 1435, 1274, 272.

HRMS (ESI): *m*/*z* calcd for $C_{26}H_{25}N_2O_3$: 413.1860 $[M+H]^+$; found: 413.1853.

Corresponding ^1H NMR resonances for each diastereoizomer:

4e, major diastereoisomer.

^1H NMR (600 MHz, $CDCl_3$) δ 9.46 (d, *J* = 8.3 Hz, 1H), 8.44 (s, 1H), 8.29 (d, *J* = 9.1 Hz, 1H), 8.06–8.02 (m, 1H), 7.97–7.96 (m, 1H), 7.58–7.55 (m, 1H), 7.52–7.47 (m, 1H), 7.47–7.42 (m, 2H), 6.41 (br s, 1H), 5.86 (dd, *J* = 3.8, 1.4 Hz, 1H), 5.21 (d, *J* = 6.9 Hz, 1H), 3.91 (dd, *J* = 6.9, 3.8 Hz, 1H), 3.84 (s, 3H), 2.97 (pd, *J* = 6.6, 3.0 Hz, 1H), 2.63 (dt, *J* = 18.2, 6.6 Hz, 1H), 2.56–2.50 (m, 1H), 1.84 (dtd, *J* = 13.6, 6.5, 3.0 Hz, 1H), 1.77 (td, *J* = 13.6, 7.7 Hz, 1H), 1.10 (d, *J* = 6.5 Hz, 3H).

4e', minor diastereoisomer.

^1H NMR (600 MHz, $CDCl_3$) δ 8.77 (d, *J* = 8.1 Hz, 1H), 8.47 (s, 1H), 8.29 (d, *J* = 9.1 Hz, 1H), 8.06–8.02 (m, 2H), 7.58–7.55 (m, 1H), 7.52–7.47 (m, 3H), 7.18 (s, 1H), 5.63 (br d, *J* = 4.3 Hz, 1H), 5.59 (d, *J* = 5.1 Hz, 1H), 4.09 (br t, *J* = 5.1 Hz, 1H), 3.86 (s, 3H), 2.83 (dtd, *J* = 10.7, 6.4, 4.3 Hz, 1H), 2.58–2.52 (m, 1H), 2.63 (dt, *J* = 18.2, 6.4 Hz, 1H), 1.91 (dddd, *J* = 13.3, 10.7, 6.5, 4.3 Hz, 1H), 1.61–1.56 (m, 1H), 0.80 (d, *J* = 6.5 Hz, 3H).

Compounds 4f/4f'/4f'': Methyl 9-(furan-2-yl)-7-methyl-4-oxo-2a,4,5,6,7,9a-hexahydro-9*H*-cyclobuta[3,4]pyrazolo[1,2-*a*]pyridazine-2-carboxylate.

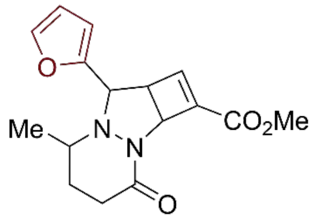

4f/4f'/4f''

Prepared according to the general procedure from **2f** (0.2 mmol). Product was isolated as a mixture of three diastereoisomers **4f/4f'/4f''** (28 mg, 46% yield, *d.r.* = 2:2:1) by CC (PE/EA = 1:1), and the diastereoisomers were further separated by preparative RP-CC (general purification procedure).

4f, major diastereoisomer, isolated as a colorless oil (11 mg, 18% yield).

^1H NMR (600 MHz, $CDCl_3$) δ 7.37–7.37 (m, 1H), 6.97–6.97 (m, 1H), 6.32 (dd, *J* = 3.2, 1.8 Hz, 1H), 6.01 (d, *J* = 3.2 Hz, 1H), 5.84 (dd, *J* = 3.5, 1.8 Hz, 1H), 4.20 (s, 1H), 3.76 (s, 3H), 3.55–

3.49 (m, 1H), 3.45 (d, *J* = 3.5 Hz, 1H), 2.38 (ddd, *J* = 17.6, 12.3, 7.8 Hz, 1H), 2.21 (ddd, *J* = 17.6, 5.8, 1.5 Hz, 1H), 1.35 (d, *J* = 7.0 Hz, 3H), 1.33–1.28 (m, 1H), 0.27 (ddd, *J* = 25.5, 12.3, 5.8 Hz, 1H).

$^{13}C\{^1H\}$ NMR (600 MHz, $CDCl_3$) δ 168.2, 161.7, 152.8, 147.8, 142.0, 138.1, 111.1, 108.45, 56.3, 54.1, 51.87, 51.43, 50.88, 30.5, 24.4, 17.9.

IR (neat, cm^{-1}): $\tilde{\nu}$ = 2947, 1709, 1656, 1617, 1426, 1226, 746.

HRMS (ESI): *m*/*z* calcd for $C_{16}H_{19}N_2O_4$: 303.1339 [*M*+H]$^+$; found: 303.1340.

4f', major diastereoisomer, isolated as a colorless oil (11 mg, 18% yield).

^1H NMR (600 MHz, $CDCl_3$) δ 7.37–7.37 (m, 1H), 6.98–6.97 (m, 1H), 6.33–6.33 (m, 1H), 6.14 (d, *J* = 3.4 Hz, 1H), 5.76–5.75 (br dd, 1H), 4.37 (s, 1H), 3.76 (s, 3H), 3.57 (d, *J* = 3.4 Hz, 1H), 2.39 (ddd, *J* = 17.6, 5.1, 1.8 Hz, 1H), 2.33–2.29 (m, 1H), 2.24 (ddd, *J* = 17.6, 12.4, 6.1 Hz, 1H), 1.72 (ddd, *J* = 24.5, 12.4, 5.1 Hz, 1H), 1.65–1.61 (m, 1H), 1.19 (d, *J* = 6.1 Hz, 3H).

$^{13}C\{^1H\}$ NMR (600 MHz, $CDCl_3$) δ 164.4, 161.5, 151.7, 147.4, 142.7, 138.8, 110.3, 108.9, 57.91, 57.01, 52.06, 51.97, 48.8, 30.1, 28.8, 19.2.

IR (neat, cm^{-1}): $\tilde{\nu}$ = 2948, 1717, 1621, 1442, 1154, 794.

HRMS (ESI): *m*/*z* calcd for $C_{16}H_{19}N_2O_4$: 303.1339 [*M*+H]$^+$; found: 303.1340.

4f'', minor diastereoisomer, isolated as a colorless oil (6 mg, 10% yield).

^1H NMR (600 MHz, $CDCl_3$) δ 7.05–7.03 (m, 3H), 7.01–7.01 (br s, 1H), 5.79 (dd, *J* = 3.5, 1.6 Hz, 1H), 4.24 (s, 1H), 3.78 (s, 3H), 3.50 (d, *J* = 3.5 Hz, 1H), 2.41 (ddd, *J* = 17.8, 5.0, 1.6 Hz, 1H), 2.38–2.33 (m, 1H), 2.25 (ddd, *J* = 17.8,12.7, 6.0 Hz, 1H), 1.75 (ddd, *J* = 25.0, 12.7, 5.0 Hz, 1H), 1.67–1.63 (m, 1H), 1.09 (d, *J* = 6.0 Hz, 3H).

$^{13}C\{^1H\}$ NMR (600 MHz, $CDCl_3$) δ 164.39, 161.52, 147.8, 138.5, 134.2, 129.75, 129.70, 115.9, 63.6, 57.2, 52.01, 51.28, 50.98, 30.13, 29.04, 18.8.

IR (neat, cm^{-1}): $\tilde{\nu}$ = 2951, 1717, 1624, 1441, 1223, 796.

HRMS (ESI): *m*/*z* calcd for $C_{16}H_{19}N_2O_4$: 303.1339 [*M*+H]$^+$; found: 303.1340.

Compounds 4g/4g'/4g'': 7-methyl-9-phenyl-2-(pyrrolidine-1-carbonyl)-2a,6,7,9a-tetrahydro-9*H*-cyclobuta[3,4]pyrazolo[1,2-*a*]pyridazin-4(5*H*)-one.

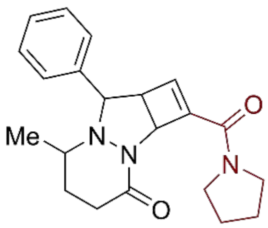

4g/4g'/4g''

Prepared according to the general procedure from **2g** (0.2 mmol). The product was isolated as a mixture of diastereoisomers **4g**/**4g'**/**4g**'' by radial chromatography (isocratic hold DCM, then gradient to DCM:MeOH = 10:1; Rf (DCM/MeOH=10:1) = 0.41) (34 mg, 49% yield, *d.r.* = 5:1.3:1).

Due to similar concentrations of diastereoisomers in the racemic mixture **4g**/**4g'**/**4g**'', individual assignment of ^1H resonances was not possible.

^{1}H NMR (600 MHz, $CDCl_3$) δ 7.33–7.03 (m, 5H), 6.90–6.90, 6.79–6.79, and 6.43–6.43 (br d, 5:20:4, *J* = 1.9, 2.8, 2.4 Hz, 1H), 5.86, 5.75, and 5.61 (3dd, 20:4:5, *J* = (3.8, 1.8 Hz), (3.7, 1.2 Hz), (3.8, 1.9 Hz), 1H), 4.22–1.11 (m, 15H), 1.09, 1.08, and 0.95 (3dd, 5:20:4, *J* = 5.5, 6.0, 6.7 Hz, 3H).

^{13}C{1H} NMR (600 MHz, $CDCl_3$) δ 167.4, 165.6, 164.6, 161.40, 161.10, 160.91, 145.1, 143.92, 143.19, 143.08, 142.18, 141.86, 138.7, 137.3, 128.96, 128.93, 128.69, 128.65, 128.46, 128.13, 128.09, 127.93, 127.90, 127.56, 69.51, 64.99, 64.47, 64.05, 59.94, 59.11, 57.7, 54.8, 53.18, 52.33, 51.19, 51.0, 50.0, 46.76, 46.70, 46.54, 46.36, 46.26, 30.10, 30.07, 28.77, 27.99, 27.07, 26.49, 26.42, 26.39, 24.26, 24.00, 23.97, 23.96, 19.60, 19.09, 18.91.

IR (neat, cm^{-1}): $\tilde{\nu}$ = 2970, 1618, 1600, 1434, 1412, 915, 726.

HRMS (ESI): *m*/*z* calcd for $C_{21}H_{26}N_3O_2$: 352.2020 $[M+H]^+$; found: 352.2018.

Compounds 4h/4h'/4h'': Methyl 7-cyclohexyl-4-oxo-9-phenyl-2a,4,5,6,7,9a-hexahydro-9*H*-cyclobuta[3,4]pyrazolo[1,2-*a*]pyridazine-2-carboxylate.

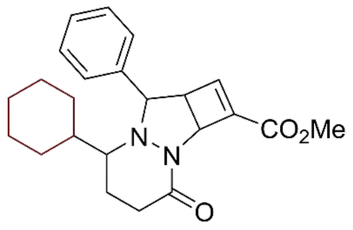

4h/4h'/4h''

Prepared according to the general procedure from **2h**/**2h'** (0.2 mmol, *d.r.* = 4:1). The product was isolated as a mixture of diastereoisomers **4h**/**4h'**/**4h**'' by preparative RP-CC (general purification procedure) (37 mg, 48% yield, *d.r.* = 10:5:1).

^{13}C{1H} NMR (600 MHz, $CDCl_3$) δ 168.16, 167.76, 165.1, 162.43, 161.56, 148.19, 147.98, 147.29, 140.68, 140.54, 139.06, 138.38, 138.21, 136.1, 128.96, 128.71, 128.53, 128.19, 128.08, 128.05, 127.86, 67.9, 64.95, 64.10, 61.57, 59.2, 58.1, 56.75, 56.60, 55.57, 53.8, 51.89, 51.80, 51.19, 51.16, 39.10, 38.34, 37.0, 30.96, 30.69, 30.03, 29.69, 29.59, 28.61, 27.51, 27.13, 26.97, 26.87, 26.69, 26.50, 26.46, 26.30, 26.18, 26.11, 20.9, 19.7, 16.2.

IR (neat, cm^{-1}): $\tilde{\nu}$ = 1926, 1720, 2+32, 2558, 2525, 908, 728, 700.

HRMS (ESI): *m*/*z* calcd for $C_{23}H_{29}N_2O_3$: 381.2173 $[M+H]^+$; found: 381.2171.

Corresponding ^{1}H NMR resonances for each diastereoizomer:

4h.

^{1}H NMR (600 MHz, $CDCl_3$) δ 7.34–7.28 (m, 3H), 7.18–7.17 (m, 1H), 7.01–6.99 (m, 2H), 5.82 (dd, *J* = 3.6, 1.7 Hz, 1H), 4.17 (s, 1H), 3.76 (s, 3H), 3.54 (d, *J* = 3.6 Hz, 1H), 2.27–2.24 (m, 1H), 2.02 (ddd, *J* = 17.4, 11.4, 5.4 Hz, 1H), 2.32–0.88 (m, 14H).

4h'.

^{1}H NMR (600 MHz, $CDCl_3$) δ 7.34–7.28 (m, 3H), 7.01–6.99 (m, 3H), 5.40 (dd, *J* = 3.8, 2.1 Hz, 1H), 4.00 (d, *J* = 3.0 Hz, 1H), 3.78 (s, 3H), 2.86–2.82 (m, 1H), 2.35–2.33 (m, 1H), 2.00–1.97 (m, 1H), 1.92–1.89 (m, 1H), 2.32–0.88 (m, 13H).

4h''.

^{1}H NMR (600 MHz, $CDCl_3$) δ 7.34–7.28 (m, 5H), 6.71–6.70 (m, 1H), 5.71 (dd, *J* = 3.6, 1.2 Hz, 1H), 3.77 (s, 3H), 2.49–0.69 (m, 15H).

Compounds 4i/4i'/4i'': Methyl 4-oxo-7,9-diphenyl-2a,4,5,6,7,9a-hexahydro-9*H*-cyclobuta[3,4]pyrazolo[1,2-*a*]pyridazine-2-carboxylate.

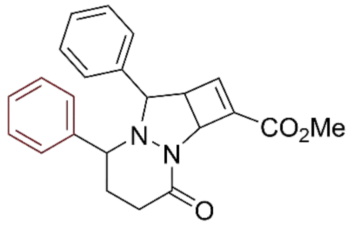

4i/4i'/4i''

Prepared according to the general procedure from **2i** (0.2 mmol). The product was isolated as a mixture of diastereoisomers **4i/4i'/4i''** by preparative RP-CC (general purification procedure) (65 mg, 87% yield, *d.r.* = 4:3:1), and the diastereoisomers were further separated by radial chromatography (PE/EA=1:1, then gradient to 100% EA).

4i, major diastereoisomer, isolated as a colorless oil (31 mg, 42% yield, R_f = 0.10).

^{1}H NMR (600 MHz, $CDCl_3$) δ 7.34–7.28 (m, 6H), 7.19–7.17 (m, 2H), 7.03 (dd, *J* = 1.7, 0.8 Hz, 1H), 6.79–6.78 (m, 2H), 5.89 (dd, *J* = 3.7, 1.7 Hz, 1H), 3.94 (s, 1H), 3.83 (s, 3H), 3.55 (d, *J* = 3.7 Hz, 1H), 3.31 (dd, *J* = 11.7, 2.7 Hz, 1H), 2.45 (ddd, *J* = 17.5, 5.0, 2.17 Hz, 1H), 2.35 (ddd, *J* = 17.5, 12.5, 5.8 Hz, 1H), 2.01 (ddd, *J* = 24.9, 11.7, 5.0 Hz, 1H), 1.80–1.76 (m, 1H).

^{13}C{1H} NMR (600 MHz, $CDCl_3$) δ 164.2, 161.7, 148.11, 140.75, 138.38, 138.00, 128.77, 128.75, 128.49, 128.40, 128.05, 127.44, 63.9, 60.4, 57.3, 52.0, 50.9, 31.21, 30.46.

IR (neat, cm^{-1}): $\tilde{v}$= 2954, 1714, 1631, 1450, 1280, 759, 702.

HRMS (ESI): *m*/*z* calcd for $C_{23}H_{23}N_2O_3$: 375.1703 [*M*+H]$^+$; found: 375.1699.

4i', isolated as a colorless oil (23 mg, 31% yield, R_f = 0.12).

^{1}H NMR (600 MHz, $CDCl_3$) δ 7.23–7.21 (m, 3H), 7.18–7.16 (m, 5H), 7.06 (dd, *J* = 1.9, 1.0 Hz, 1H), 6.84–6.82 (m, 2H), 5.60 (dd, *J* = 3.7, 1.9 Hz, 1H), 4.43 (dd, *J* = 9.0, 4.9 Hz, 1H), 3.90 (d, *J* = 2.3 Hz, 1H), 3.83 (s, 3H), 3.52 (br ddd, *J* = 3.7, 2.3, 1.0 Hz, 1H), 2.55 (dt, *J* = 16.6, 6.8, 6.6 Hz, 1H), 2.47 (ddd, *J* = 16.6., 7.3, 6.8 Hz, 1H), 1.98 (dtd, *J* = 14.0, 6.6, 4.9 Hz, 1H), 1.66 (ddt, *J* = 14.0, 9.0, 7.3 Hz, 1H).

^{13}C{1H} NMR (600 MHz, $CDCl_3$) δ 168.1, 162.3, 149.3, 141.00, 140.90, 139.68, 128.61, 128.49, 127.99, 127.92, 127.72, 127.55, 64.7, 63.1, 58.9, 53.8, 52.1, 30.39, 26.92.

IR (neat, cm^{-1}): $\tilde{v}$= 2949, 1720, 1644, 1434, 1275, 752, 698.

HRMS (ESI): *m*/*z* calcd for $C_{23}H_{23}N_2O_3$: 375.1703 [*M*+H]$^+$; found: 375.1698.

4i'', minor diastereoisomer, isolated as a colorless oil (7 mg, 10% yield, R_f = 0.08).

^{1}H NMR (600 MHz, $CDCl_3$) δ 7.21–7.14 (m, 10H), 6.68–6.67 (m, 1H), 5.81 (dd, *J* = 3.7, 1.0 Hz, 1H), 4.11 (dd, *J* = 4.9, 3.3 Hz, 1H), 3.86–3.85 (m, 1H), 3.86 (s, 3H), 3.64 (ddd, *J* = 6.6, 3.7, 1.0 Hz, 1H), 2.41–2.37 (m, 1H), 2.21 (ddd, *J* = 16.4, 9.3, 7.2 , 6.6 Hz, 1H), 2.14–2.10 (m, 2H).

^{13}C{1H} NMR (600 MHz, $CDCl_3$) δ 165.5, 161.6, 147.6, 140.5, 139.4, 135.9, 128.69, 128.39, 128.14, 127.97, 127.18, 126.87, 69.1, 57.7, 55.6, 52.0, 50.5, 27.7, 23.2.

IR (neat, cm^{-1}): $\tilde{v}$= 2950, 1722, 1642, 1434, 1271, 750, 698.

HRMS (ESI): *m*/*z* calcd for $C_{23}H_{23}N_2O_3$: 375.1703 [*M*+H]$^+$; found: 375.1699.

Compounds 4j/4j'/4j'': Methyl 6-oxo-10-phenyl-1,2,3,4,4a,5,6,7a,9a,11a-decahydro-10*H*-cyclobuta[3,4]pyrazolo[1,2-*a*]cinnoline-8-carboxylate.

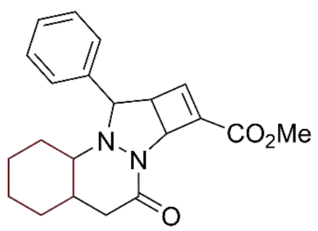

4j/4j'/4j''

Prepared according to the general procedure from **2j/2j'/2j''** (0.2 mmol, *d.r.* = 2:2:1). The crude reaction mixture was partially separated by preparative RP-CC (general purification procedure), affording the pure diastereoisomer **4j** (6 mg, 8%) and a mixture of diastereoisomers **4j/4j'/4j''** (46 mg, 65% yield, *d.r.* = 1:2:1).

4j, major diastereoisomer, isolated as a colorless oil.

^{1}H NMR (600 MHz, $CDCl_3$) δ 7.35–7.30 (m, 3H), 7.06–7.04 (m, 2H), 7.02 (br s, 1H), 5.79 (dd, *J* = 3.7, 1.8 Hz, 1H), 4.26 (s, 1H), 3.77 (s, 3H), 3.53 (d, *J* = 3.7 Hz, 1H), 2.37 (dd, *J* = 17.2, 4.2 Hz, 1H), 1.93–1.88 (m, 2H), 1.88 (dd, *J* = 17.2, 12.2 Hz, 1H), 1.78–1.72 (m, 1H), 1.67–1.58 (m, 3H), 1.29–1.16 (m, 2H), 1.02–0.94 (m, 1H), 0.82–0.75 (m, 1H).

^{13}C{1H} NMR (600 MHz, $CDCl_3$) δ 164.0, 161.6, 148.0, 138.43, 138.41, 128.99, 128.23, 128.07, 63.7, 59.5, 57.0, 51.97, 51.15, 37.4, 36.1, 31.5, 29.5, 25.2, 24.0.

IR (neat, cm^{-1}): $\tilde{v}$= 2929, 1719, 1622, 1438, 1228, 723.

HRMS (ESI): *m*/*z* calcd for $C_{21}H_{25}N_2O_3$: 353.1860 [*M*+H]$^+$; found: 353.1862.

4j/4j'/4j'' (*d.r.* = 1:2:1).

^{13}C{1H} NMR (600 MHz, $CDCl_3$) δ 167.0, 166.0, 163.3, 161.94, 161.63, 161.53, 148.70, 147.90, 147.24, 141.5, 139.24, 139.12, 138.40, 138.31, 136.8, 128.95, 128.90, 128.75, 128.27, 128.17, 128.07, 128.02, 127.89, 127.85, 68.5, 64.4, 63.15, 62.45, 56.97, 56.94, 55.64, 55.34, 53.09, 52.43, 51.88, 51.84, 50.84, 50.40, 37.30, 36.33, 35.2, 33.18, 32.32, 32.14, 29.00, 28.51, 28.28, 28.21, 27.51, 27.31, 25.73, 25.49, 24.99, 24.12, 21.0, 19.9.

IR (neat, cm^{-1}): $\tilde{v}$= 2931, 1721, 1625, 1436, 1274, 726.

HRMS (ESI): *m*/*z* calcd for $C_{21}H_{25}N_2O_3$: 353.1860 [*M*+H]$^+$; found: 353.1862.

Corresponding ^{1}H NMR resonances for each diastereoizomer:

4j' (major):

^{1}H NMR (600 MHz, $CDCl_3$) δ 7.35–7.28 (m, 4H), 7.05–7.04 (m, 1H), 6.60 (s, 1H), 5.71–5.70 (m, 1H), 3.76 (s, 3H), 3.70 (d, *J* = 6.5 Hz, 1H), 3.53 (dd, *J* = 12.9, 3.6 Hz, 1H), 2.82–2.78 (m, 1H), 2.58 (dd, *J* = 17.7, 10.6 Hz, 1H), 2.37–0.67 (m, 10H).

4j'' (minor):

^{1}H NMR (600 MHz, $CDCl_3$) δ 7.35–7.28 (m, 4H), 7.10–7.09 (m, 2H), 5.68–5.67 (m, 1H), 4.03–4.03 (m, 1H), 3.76 (s, 3H), 3.51 (d, *J* = 3.5 Hz, 1H), 3.38–3.37 (m, 1H), 2.88 (dt, *J* = 11.3, 3.5 Hz, 1H), 2.26 (dd, *J* = 17.7, 5.6 Hz, 1H), 2.37–0.67 (m, 9H).

8.3 Time-course monitoring of the reaction of **2a** to **3a** and **4a**

*8.3.1 Irradiation of **2a** with 450 nm light*

An NMR tube was charged with **2a** (0.02 mmol), 1,3,5-trimethoxybenzene (internal standard for NMR, 0.03 mmol, 5.1 mg) and CD_2Cl_2 (0.6 mL) under an argon atmosphere. The resulting solution was irradiated with a 450 nm LED (10% intensity) and reaction progress was monitored by recording ^{1}H NMR at various time intervals. The ^{1}H NMR yields of **2a**, **3a** and **4a** are depicted in the following graph:

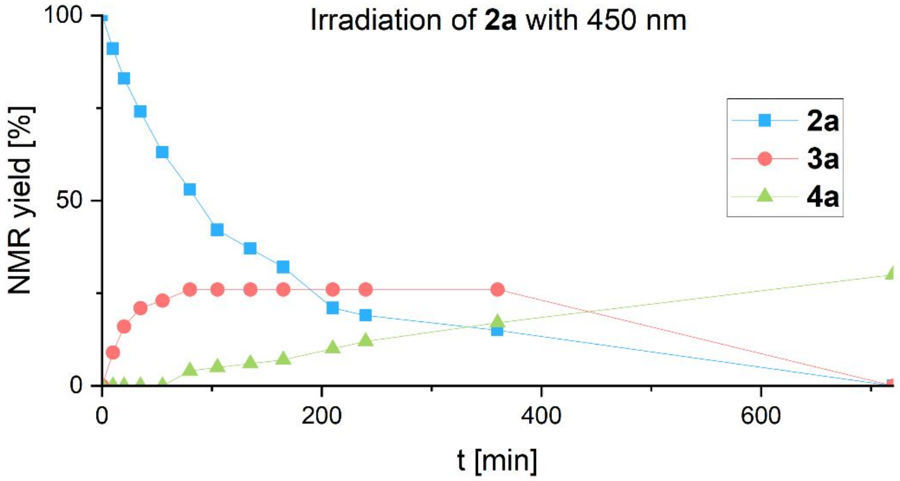

Figure S1: ^{1}H NMR yields of **2a**, **3a** and **4a** during the time-course monitoring (450 nm).

The experiment showed that the ring expansion from **2a** to **3a** proceeds at a slower rate than the subsequent 4π-electrocyclization of **3a** to **4a**. As a result, **3a** is rapidly consumed once formed, which prevents its accumulation in the reaction mixture and hinders its isolation in high yield under the investigated conditions.

*8.3.2 Irradiation of **2a** with 365 nm light*

An NMR tube was charged with **2a** (0.02 mmol), 1,3,5-trimethoxybenzene (internal standard for NMR, 0.03 mmol, 5.3 mg) and CD_2Cl_2 (0.6 mL) under an argon atmosphere. The resulting solution was irradiated with a 365 nm LED (50% intensity) and reaction progress was monitored by recording ^{1}H NMR at various time intervals. The ^{1}H NMR yields of **2a**, **3a** and **4a** are depicted in the following graph:

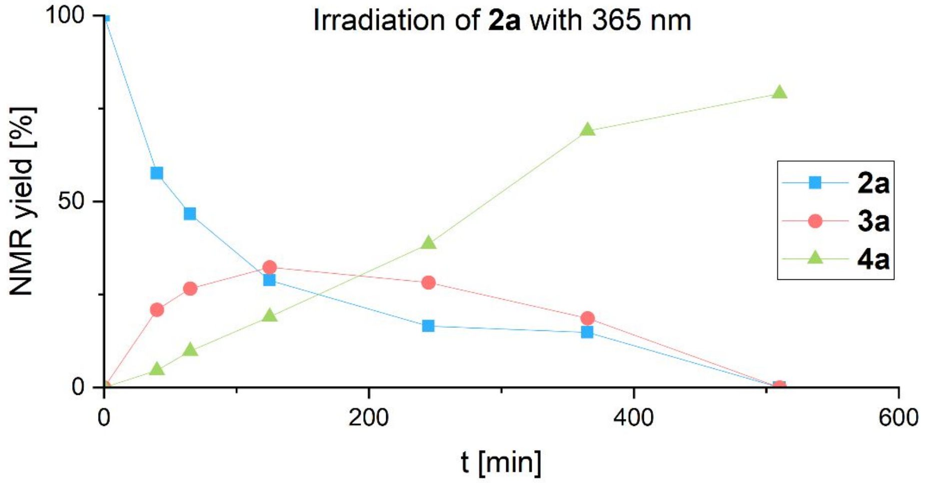

Figure S2: ^{1}H NMR yields of **2a**, **3a** and **4a** during the time-course monitoring (365 nm).

Irradiating **2a** with 365 nm led to the formation of **4a** in a higher NMR yield (79%). Similar to irradiation with 450 nm, **3a** is rapidly consumed for the formation of **4a**, its NMR yield not exceeding 32 %, which hinders its isolation in a higher yield.

8.3.3 On-off experiment (365 nm)

An NMR tube was charged with **2a** (0.02 mmol), 1,3,5-trimethoxybenzene (internal standard for NMR, 0.03 mmol, 5.3 mg) and CD_2Cl_2 (0.6 mL) under an argon atmosphere. The resulting solution was alternately irradiated with a 365 nm LED (50% intensity) and kept in the dark for consecutive time intervals. At each time point, a ^{1}H NMR spectrum was recorded. The ^{1}H NMR yields of **2a**, **3a** and **4a** are depicted in the following graph:

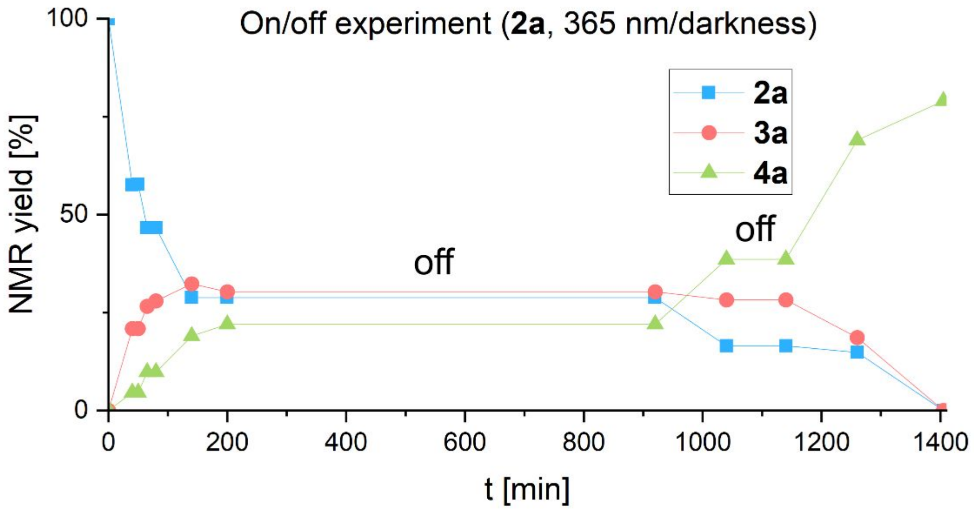

Figure S3: ^{1}H NMR yields of **2a**, **3a** and **4a** during the on-off experiment (365 nm).

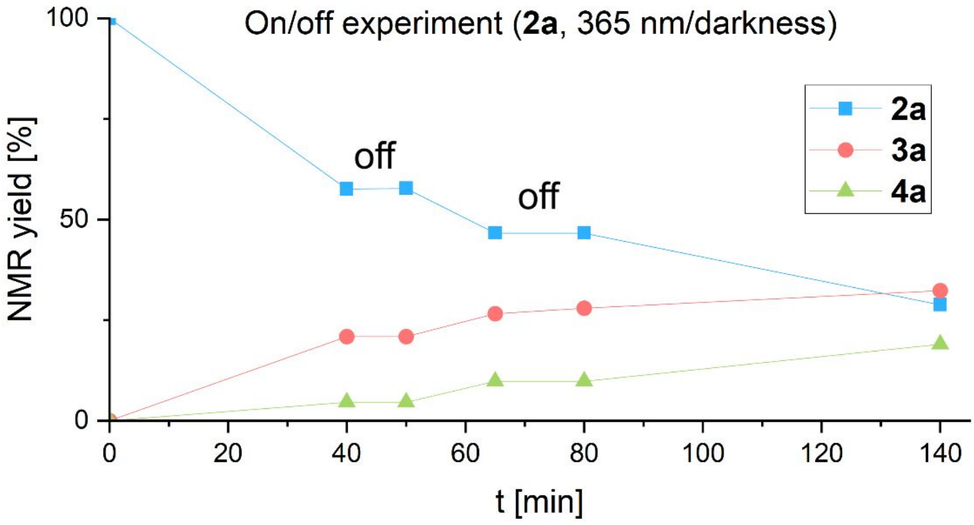

Figure S4: Zoomed-in graph for the on-off experiment (365 nm) for the 0–140 minute period.

The experiment revealed that no conversion occurred during the dark intervals, even when the reaction mixture was kept in the dark for up to 12 hours. These results indicate that continuous irradiation with light is essential for the transformation of **2a** to **3a** and **4a**.

9. Synthesis of products *E*-2-OH and *Z*-2-OH

9.1 General procedures

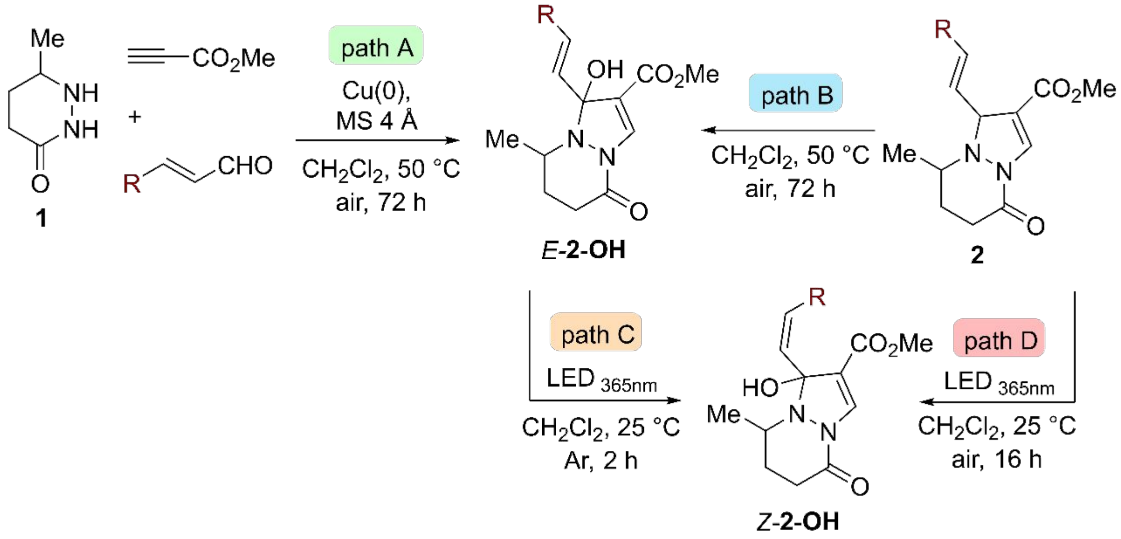

Scheme S8: Formation of products *E*-**2-OH** and *Z*-**2-OH** under different reaction conditions

Path A: A vial was charged with **1** (1.0 mmol), 3-(hetero)aryl-enal (1.5 mmol), CH_2Cl_2 (5.0 mL), methyl propiolate (1.1 mmol), copper(0) (0.7 mmol), molecular sieves (4 Å; 100 mg) and was sealed off with a screw cap. The reaction mixture was reacted in the dark at 50 °C (sand bath) for 72 h (under air). Products *E*-**2-OH** were obtained by CC (PE/EA = 5:1, then gradient to PE/EA = 1:1).

Path B: A vial was charged with **2** (1.0 mmol) and CH_2Cl_2 (5.0 mL), was sealed off with a screw cap, and was reacted in the dark at 50 °C (sand bath) for 72 h (under air). Products *E*-**2-OH** were obtained by CC (PE/EA = 5:1, then gradient to PE/EA = 1:1).

Path C: A vial was charged with *E*-**2-OH** (1.0 mmol) and CH_2Cl_2 (6.0 mL), was sealed off with a screw cap equipped with a septum, and the resulting solution was purged with argon. The mixture was then irradiated with LED_{365nm} for 2 h at 25 °C. Products *Z*-**2-OH** were obtained by CC (PE/EA = 5:3).

Path D: A vial was charged with **2** (1.0 mmol) and CH_2Cl_2 (6.0 mL), was sealed off with a screw cap, and the resulting solution was irradiated with LED_{365nm} for 16 h at 25 °C (under air). Products *Z*-**2-OH** were obtained by CC (PE/EA = 5:3).

9.2 Characterization data of products *E*-2-OH and *Z*-2-OH

Compound *E*-2a-OH: **Methyl (*E*)-1-hydroxy-8-methyl-5-oxo-1-styryl-5,6,7,8-tetrahydro-1*H*-pyrazolo[1,2-*a*]pyridazine-2-carboxylate.**

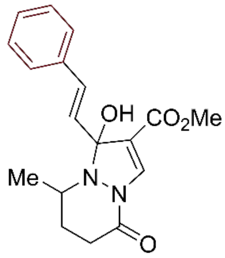

E-**2a-OH**

Prepared according to the general procedure "Path A" from **1a** (1.0 mmol), cinnamaldehyde (1.5 mmol) and methyl propiolate (1.1 mmol). The crude reaction mixture was purified by CC (general purification procedure; R_f = 0.20 (in PE/EA = 1:1)). Product *E*-**2a-OH** was isolated as a yellow oil (240 mg, 73% yield).

Product *E*-**2a**-**OH** was also prepared according to the general procedure "Path B" from **2a** (0.5 mmol). The crude reaction mixture was purified by CC (general purification procedure). Product *E*-**2a**-**OH** was isolated as a yellow oil (140 mg, 85% yield).

^{1}H NMR (600 MHz, $CDCl_3$) δ 8.00 (s, 1H), 7.52–7.51 (m, 2H), 7.36–7.34 (m, 2H), 7.31–7.28 (m, 2H), 7.24 (d, *J* = 16.7 Hz, 1H), 7.16 (d, *J* = 16.7 Hz, 1H), 4.72 (dqd, *J* = 13.4, 6.7, 4.2 Hz, 1H), 3.82 (s, 3H), 2.34–2.28 (m, 1H), 2.20–2.15 (m, 1H), 2.09–2.00 (m, 2H), 1.54 (d, *J* = 6.7 Hz, 3H).

^{13}C{1H} NMR (600 MHz, $CDCl_3$) δ 178.5, 164.2, 143.4, 142.3, 138.3, 136.3, 129.12, 128.98, 127.3, 114.0, 111.6, 54.0, 51.6, 31.3, 30.2, 21.3.

IR (neat, cm^{-1}): $\tilde{v}$ = 2948, 1705, 1535, 1209, 730.

HRMS (ESI): *m*/*z* calcd for $C_{18}H_{21}N_2O_4$: 329.1496 [*M*+H]$^+$; found: 329.1503.

Compound *Z*-2a-OH: Methyl (*Z*)-1-hydroxy-8-methyl-5-oxo-1-styryl-5,6,7,8-tetrahydro-1*H*-pyrazolo[1,2-*a*]pyridazine-2-carboxylate.

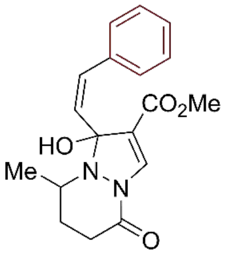

***Z*-2a-OH**

Prepared according to the general procedure "Path C" from *E*-**2a**-**OH** (0.3 mmol). The crude reaction mixture was purified by CC (PE/EA = 5/3; Rf = 0.17), affording *Z*-**2a**-**OH** as a yellow oil (94 mg, 95% yield).

Product *Z*-**2a**-**OH** was also prepared according to the general procedure "Path D" from **2a** (0.5 mmol). The crude reaction mixture was purified by CC (PE/EA = 5/3; Rf = 0.17), affording *Z*-**2a**-**OH** as a yellow oil (81 mg, 49% yield).

^{1}H NMR (600 MHz, $CDCl_3$) δ 8.00 (s, 1H), 7.22–7.20 (m, 3H), 6.99–6.97 (m, 2H), 6.92 (d, *J* = 12.3 Hz, 1H), 6.51 (d, *J* = 12.3 Hz, 1H), 4.16 (dqd, *J* = 9.8, 6.8, 4.3 Hz, 1H), 3.79 (s, 3H), 2.05–1.93 (m, 2H), 1.89–1.83 (m, 2H), 0.96 (d, *J* = 6.6 Hz, 3H).

^{13}C{1H} NMR (600 MHz, $CDCl_3$) δ 177.5, 163.8, 142.41, 141.88, 136.76, 135.83, 128.77, 128.72, 128.60, 117.0, 112.7, 54.2, 51.4, 31.3, 30.2, 19.7.

IR (neat, cm^{-1}): $\tilde{v}$ = 2949, 1707, 1537, 1220, 732.

HRMS (ESI): *m*/*z* calcd for $C_{18}H_{21}N_2O_4$: 329.1496 [*M*+H]$^+$; found: 329.1501.

Compound *E*-2b-OH: Methyl (*E*)-1-(4-fluorostyryl)-1-hydroxy-8-methyl-5-oxo-5,6,7,8-tetrahydro-1*H*-pyrazolo[1,2-*a*]pyridazine-2-carboxylate.

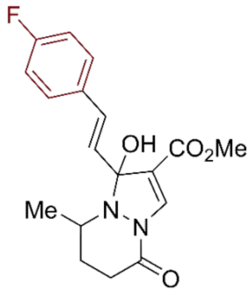

***E*-2b-OH**

Prepared according to the general procedure "Path A" from **1a** (0.5 mmol), p-fluorocinnamaldehyde (1.5 mmol) and methyl propiolate (1.1 mmol). The crude reaction mixture was purified by CC (PE/EA = 5/1; R_f = 0.05), affording *E*-**2b**-**OH** as a yellow oil (146 mg, 84% yield).

Product *E*-**2b**-**OH** was also prepared according to the general procedure "Path B" from **2b** (0.5 mmol). The crude reaction mixture was purified by CC (PE/EA = 5/1; R_f = 0.05), affording *E*-**2b**-**OH** as a yellow oil (156 mg, 90% yield).

^{1}H NMR (600 MHz, $CDCl_3$) δ 7.98 (s, 1H), 7.51–7.49 (m, 2H), 7.24 (d, *J* = 16.6 Hz, 1H), 7.08 (d, *J* = 16.6 Hz, 1H), 7.05–7.03 (m, 2H), 4.71 (br p, *J* = 4.5 Hz, 1H), 3.82 (s, 3H), 2.36–2.29 (m, 1H), 2.26–2.19 (m, 1H), 2.10–2.03 (m, 2H), 1.54 (d, *J* = 6.6 Hz, 3H).

^{13}C{1H} NMR (600 MHz, $CDCl_3$) δ 164.1, 162.4, 143.2, 142.2, 137.0, 132.5, 128.91, 128.86, 116.00, 115.86, 113.6, 111.4, 53.8, 51.5, 31.2, 21.2.

IR (neat, cm^{-1}): $\tilde{\nu}$ = 2950, 1706, 1508, 1220, 818, 777.

HRMS (ESI): *m*/*z* calcd for $C_{18}H_{20}FN_2O_4$: 347.1402 [*M*+H]$^+$; found: 347.1399.

Compound *Z*-2b-OH: Methyl (*Z*)-1-hydroxy-8-methyl-5-oxo-1-styryl-5,6,7,8-tetrahydro-1*H*-pyrazolo[1,2-*a*]pyridazine-2-carboxylate.

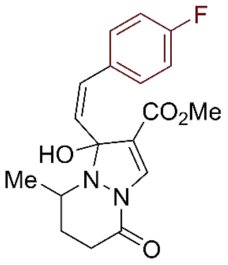

Z-**2b-OH**

Prepared according to the general procedure "Path C" from *E*-**2b-OH** (0.3 mmol). The crude reaction mixture was purified by CC (DCM/MeOH = 10:1; R_f = 0.35), affording *Z*-**2b-OH** as a yellow oil (99 mg, 95% yield).

Product *Z*-**2b-OH** was also prepared according to the general procedure "Path D" from **2b** (0.5 mmol). The crude reaction mixture was purified by CC (DCM/MeOH = 10:1; R_f = 0.35), affording *Z*-**2b-OH** as a yellow oil (161 mg, 93% yield).

^{1}H NMR (600 MHz, $CDCl_3$) δ 8.01 (s, 1H), 6.98–6.96 (m, 2H), 6.91 (t, *J* = 8.7 Hz, 2H), 6.87 (d, *J* = 11.9 Hz, 1H), 6.48 (d, *J* = 11.9 Hz, 1H), 4.21–4.16 (m, 1H), 3.78 (s, 3H), 2.06–1.98 (m, 2H), 1.92–1.87 (m, 2H), 1.02 (d, *J* = 6.6 Hz, 3H).

^{13}C{1H} NMR (600 MHz, $CDCl_3$) δ 177.7, 163.69, 163.47, 161.8, 142.13, 141.96, 135.6, 132.0, 130.6, 116.75, 115.91, 115.76, 112.7, 54.2, 51.5, 31.3, 30.2, 19.9.

IR (neat, cm^{-1}): $\tilde{\nu}$ = 2950, 1707, 1508, 1220, 839, 776.

HRMS (ESI): *m*/*z* calcd for $C_{18}H_{20}FN_2O_4$: 347.1402 [*M*+H]$^+$; found: 347.1400.

Compound *E*-2m-OH: Methyl (*E*)-1-hydroxy-8-methyl-5-oxo-1-(pent-1-en-1-yl)-5,6,7,8-tetrahydro-1H-pyrazolo[1,2-a]pyridazine-2-carboxylate.

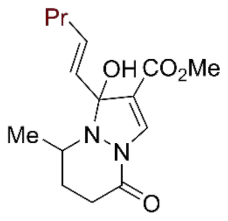

E-**2m-OH**

Prepared and isolated during the preparation of **2m/2m'** (see Section 5.2 for full procedure). *E*-**2m-OH** resulted from oxidation of **2m** during preparative RP-CC purification and was obtained as a yellow oil (68 mg, 23% yield).

^{1}H NMR (600 MHz, $CDCl_3$) δ 7.92 (s, 1H), 6.43 (dt, *J* = 16.2, 1.5 Hz, 1H), 6.26 (dt, *J* = 16.3, 6.9 Hz, 1H), 4.62–4.56 (m, 1H), 3.79 (s, 3H), 2.35–2.30 (m, 1H), 2.25 (qd, *J* = 7.0, 1.6 Hz, 2H), 2.20–2.15 (m, 1H), 2.09–2.03 (m, 2H), 1.52 (tt, *J* = 7.4, 7.4 Hz, 2H), 1.48 (d, *J* = 6.6 Hz, 3H), 0.96 (t, *J* = 7.4 Hz, 3H).

^{13}C{1H} NMR (600 MHz, $CDCl_3$) δ 178.1, 164.1, 143.7, 141.9, 116.1, 110.9 (2C), 53.6, 51.3, 35.7, 31.09, 30.14, 22.05, 21.24, 13.8.

IR (neat, cm^{-1}): $\tilde{\nu}$ = 2957, 1708, 1536, 1410, 1220, 1185, 777.

HRMS (ESI): *m*/*z* calcd for $C_{15}H_{23}N_2O_4$: 295.1652 [*M*+H]$^+$; found: 295.1656.

10. Synthesis and characterization of pyrazolo[1,2-*a*]pyridazinones 5

10.1 Reaction of **1a** with β-substituted (R ≠ H) aldehydes, non-conjugated aldehydes and less activated terminal ynones

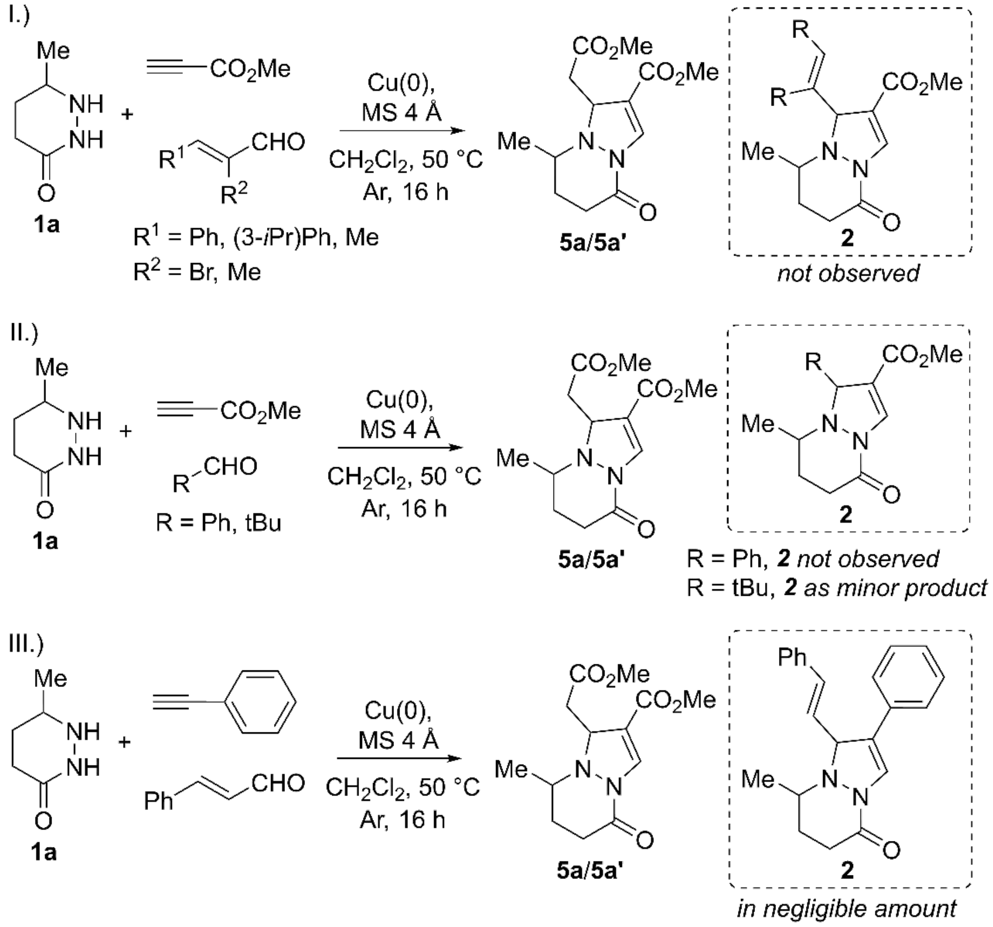

Scheme S9: The formation of products **5a**/**5a'**.

Reaction procedures were based on the general procedure for the synthesis of compounds **2** (see Section 5.1). A screw-cap vial equipped with a septum was charged with **1a** (1.0 mmol), terminal ynone (1.1 mmol), and aldehyde (1.5 mmol). Then, CH_2Cl_2 (5.0 mL), copper(0) (0.7 mmol), and molecular sieves (4 Å; 100 mg) were added. The reaction mixture was purged with argon and stirred in the dark at 50 °C (sand bath) for 16 h.

Instead of the expected compounds **2**, two diastereoisomers **5a**/**5a'** were obtained in all cases (*d.r.* = 1.3:1).

Reagents used:

I.) β-substituted (R ≠ H) aldehydes: 2-bromo-3-phenylacrylaldehyde, 3-(4-isopropylphenyl)-2-methylacrylaldehyde, and 2-methylbut-2-enal.
II.) Non-conjugated aldehydes: benzaldehyde and pivaldehyde.
III.) Less activated terminal ynone: phenylacetylene.

These results indicate that β-substituted (R ≠ H) and non-conjugated aldehydes are less reactive under these conditions compared to their β-unsubstituted (R = H) conjugated analogues, which afforded products **2** in moderate to high yields.

Similarly, when phenylacetylene – a less activated terminal alkyne – was used, the desired product **2** was formed only in trace amounts, giving **5a**/**5a’** as a main product. This observation can be attributed to the mechanistic nature of copper-catalyzed transformations, which are generally initiated through the formation of copper acetylide intermediates. Electron-deficient alkynes readily participate in this step due to their higher acidity, whereas simple alkynes such as phenylacetylene are significantly less reactive under identical conditions.[9,10]

The reaction procedure was then adapted for the synthesis of products **5** (see Section 10.2).

For details on the isolation and characterization of **5a**/**5a’,** see Section 10.3.

10.2 General procedure for the synthesis of compounds 5

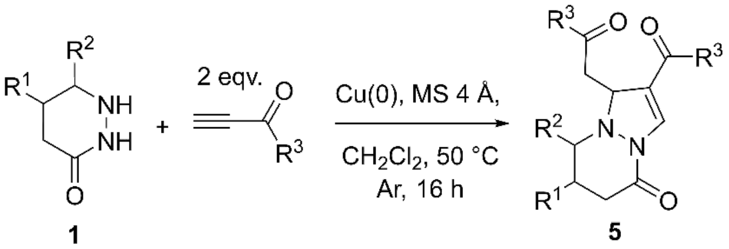

Scheme S10: Synthesis of pyrazolo[1,2-*a*]pyridazinones **5**.

A vial was charged with **1** (1.0 mmol), terminal ynone (2.5 mmol), CH_2Cl_2 (5.0 mL), copper(0) (0.7 mmol), molecular sieves (4 Å; 100 mg) and was sealed off with a screw cap with a septum. The reaction mixture was purged with argon and was reacted in the dark at 50 °C (sand bath) for 16 h. Products **5** were obtained by CC (PE/EA=5:2) or preparative RP-CC (isocratic hold at 80:20 H_2O/ MeCN for 15 min, then gradient to 50:50 over 20 min (total runtime: 35 minutes)).

10.3 Characterization data of compounds 5

Compounds 5a/5a’: Methyl 1-(2-methoxy-2-oxoethyl)-8-methyl-5-oxo-5,6,7,8-tetrahydro-1*H*-pyrazolo[1,2-*a*]pyridazine-2-carboxylate.

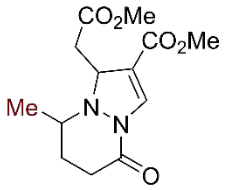

5a/5a'

Prepared according to the general procedure from **1a** (2.0 mmol) and methyl propiolate (5.0 mmol). The product was isolated as a mixture of diastereoisomers **5a**/**5a’** by CC (PE/EA=1:1) (512 mg, 91%, *d.r.* = 1.3:1), and the diastereoisomers were further separated by CC (PE/EA=5:3, R_f = 0.29 and 0.25).

5a, isolated as a yellow oil (293 mg, 52%, R_f = 0.29).

^{1}H NMR (600 MHz, $CDCl_3$) δ 7.72 (br s, 1H), 4.77 (ddd, J = 6.7, 4.6, 1.3 Hz, 1H), 3.74 (s, 3H), 3.68 (s, 3H), 2.89 (dp, J = 8.1, 6.3 Hz, 1H), 2.69 (dd, J = 4.6 Hz, 15.2 Hz, 1H), 2.63–2.56 (m, 2H), 2.38 (ddd, J = 3.6 Hz, 7.1 Hz, 15.2 Hz, 1H), 1.93 (ddt, J = 14.1, 9.3, 7.1 Hz, 1H), 1.77 (dddd, J = 13.4, 9.3, 8.1, 3.6 Hz, 1H), 1.21 (d, J = 6.3 Hz, 3H).

^{13}C{1H} NMR (600 MHz, $CDCl_3$) δ 170.9, 167.6, 164.3, 132.3, 112.0, 64.4, 57.1, 51.77, 51.74, 41.2, 29.76, 29.12, 19.1.

IR (neat, cm^{-1}): $\tilde{\nu}$= 2952, 1736, 1680, 1614, 1406, 1121, 759.

HRMS (ESI): *m*/*z* calcd for $C_{13}H_{19}N_2O_5$: 283.1288 [*M*+H]$^+$; found: 283.1284.

5a', isolated as a yellow oil (214 mg, 38%, R_f = 0.29).

^{1}H NMR (600 MHz, $CDCl_3$) δ 7.77 (br s, 1H), 4.83 (ddd, *J* = 9.4, 3.0, 1.3 Hz, 1H), 3.72 (s, 3H), 3.69 (s, 3H), 3.53 (dtdd, *J* = 13.0, 6.5, 2.8, 1.3 Hz, 1H), 2.96 (dd, *J* = 16.3, 3.0 Hz, 1H), 2.53 (dd, *J* = 16.3, 9.4 Hz, 1H), 2.48 (ddd, *J* = 16.5, 9.5, 6.9 Hz, 1H), 2.40 (ddd, *J* = 16.5, 7.3, 5.3 Hz, 1H), 2.28 (dddd, *J* = 13.4, 9.5, 7.3, 5.3 Hz, 1H), 1.54 (dddd, *J* = 13.4, 8.0, 6.9, 2.8 Hz, 1H), 1.02 (d, *J* = 6.5 Hz, 3H).

^{13}C{1H} NMR (600 MHz, $CDCl_3$) δ 171.7, 166.1, 164.2, 133.8, 112.2, 63.1, 54.2, 51.77, 51.71, 40.2, 27.60, 27.30, 12.4.

IR (neat, cm^{-1}): $\tilde{\nu}$ = 2952, 1737, 1680, 1612, 1406, 1120, 758.

HRMS (ESI): *m*/*z* calcd for $C_{13}H_{19}N_2O_5$: 283.1288 [*M*+H]$^+$; found: 283.1286.

Compounds 5b/5b': Methyl 8-cyclohexyl-1-(2-methoxy-2-oxoethyl)-5-oxo-5,6,7,8-tetrahydro-1*H*-pyrazolo[1,2-*a*]pyridazine-2-carboxylate.

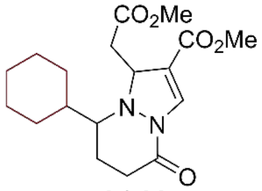

5b/5b'

Prepared according to the general procedure from **1b** (1.0 mmol) and methyl propiolate (2.5 mmol). Two diastereoisomers **5b**/**5b'** were formed (*d.r.* = 7:1, determined from the crude reaction mixture), which were separated by CC (PE/EA = 5:2, R_f = 0.19 and 0.17).

5b, isolated as a yellow oil (119 mg, 34%, R_f = 0.19).

^{1}H NMR (600 MHz, $CDCl_3$) δ 7.68 (s, 1H), 4.75 (dd, *J* = 10.0, 1.2 Hz, 1H), 3.72 (s, 3H), 3.64 (s, 3H), 2.82–2.79 (m, 1H), 2.66 (dd, *J* = 15.1, 6.0 Hz, 1H), 2.58 (dd, *J* = 15.1, 4.6 Hz, 1H), 2.43 (td, *J* = 14.0, 6.7 Hz, 1H), 2.30 (ddd, *J* = 14.0, 4.9, 2.8 Hz, 1H), 2.04 (dddd, *J* = 13.9, 6.7, 4.6, 2.8 Hz, 1H), 1.77–1.68 (m, 5H), 1.60 (tdd, *J* = 14.0, 10.0, 4.9 Hz, 1H), 1.42 (br d, *J* = 13.9 Hz, 1H), 1.35–1.20 (m, 2H), 1.15–1.00 (m, 3H).

^{13}C{1H} NMR (600 MHz, $CDCl_3$) δ 170.8, 168.3, 164.3, 132.3, 111.8, 65.39, 65.20, 51.78, 51.66, 40.7, 39.5, 30.35, 30.06, 26.75, 26.52, 25.60, 24.7, 22.5.

IR (neat, cm^{-1}): $\tilde{\nu}$ = 2927, 1686, 1619, 1415, 1248, 729.

HRMS (ESI): *m*/*z* calcd for $C_{18}H_{27}N_2O_5$: 351.1914 [*M*+H]$^+$; found: 351.1913.

5b', isolated as a yellow oil (18 mg, 5%, R_f = 0.17).

^{1}H NMR (600 MHz, $CDCl_3$) δ 7.72 (s, 1H), 5.05 (ddd, *J* = 7.5, 3.7, 1.0 Hz, 1H), 3.75 (s, 3H), 3.68 (s, 3H), 3.22 (td, *J* = 6.6, 3.6 Hz, 1H), 2.79 (dd, *J* = 15.3, 3.7 Hz, 1H), 2.57 (dd, *J* = 15.3, 7.6 Hz, 1H), 2.47–2.44 (m, 1H), 2.04 (dtd, *J* = 13.3, 6.6, 4.7 Hz, 1H), 1.86–1.80 (m, 2H), 1.78–1.67 (m, 3H), 1.56 (ddt, *J* = 25.5, 12.5, 3.6 Hz, 2H), 1.32–1.16 (m, 3H), 1.16–1.03 (m, 3H).

^{13}C{1H} NMR (600 MHz, $CDCl_3$) δ 171.6, 166.0, 164.2, 133.1, 113.6, 61.85, 60.89, 51.90, 51.79, 38.36, 38.18, 30.9, 29.4, 27.35, 26.76, 26.51, 26.33, 21.9.

IR (neat, cm^{-1}): $\tilde{\nu}$ = 2927, 1710, 1613, 1407, 1241, 1120, 730.

HRMS (ESI): *m*/*z* calcd for $C_{18}H_{27}N_2O_5$: 351.1914 [*M*+H]$^+$; found: 351.1916.

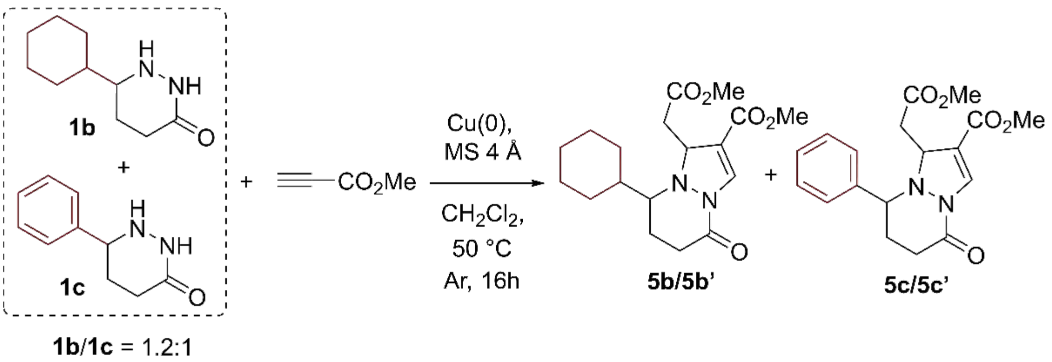

Scheme S11: Synthesis of compounds **5b/5b'** and **5c**.

Compounds 5c/5c': Methyl 1-(2-methoxy-2-oxoethyl)-5-oxo-8-phenyl-5,6,7,8-tetrahydro-1*H*-pyrazolo[1,2-*a*]pyridazine-2-carboxylate.

Prepared according to the general procedure from **1b/1c** (300 mg, **1b/1c** = 1.2:1) and methyl propiolate (5.0 mmol). The products were separated by preparative by CC (PE/EA = 5:2), affording a mixture of diastereoisomers **5b/5b'** (110 mg) and a pure product **5c** (169 mg, 60%, calculated based on the starting concentration of **1c** in the reaction mixture).

For full characterization of compounds **5b** and **5b'**, see above (previous procedure).

5c, isolated as a yellow oil.

^{1}H NMR (600 MHz, $CDCl_3$) δ 7.77 (s, 1H), 7.41–7.39 (m, 2H), 7.37–7.34 (m, 3H), 4.46 (br t, *J* = 4.9 Hz, 1H), 3.82 (t, *J* = 8.1 Hz, 1H), 3.72 (s, 3H), 3.50 (s, 3H), 2.85 (dt, *J* = 15.4, 9.3 Hz, 1H), 2.53 (dd, *J* = 15.4, 6.9, 3.8 Hz, 1H), 2.48 (dd, *J* = 14.8, 4.9 Hz, 1H), 2.27 (dtd, *J* = 12.3, 8.1, 3.8 Hz, 1H), 2.21–2.15 (m, 1H), 2.13 (dd, *J* = 14.8, 4.9 Hz, 1H).

^{13}C{1H} NMR (600 MHz, $CDCl_3$) δ 170.4, 167.2, 164.3, 139.7, 132.2, 129.18, 128.89, 128.68, 112.1, 67.1, 65.6, 51.77, 51.65, 39.9, 30.9, 29.8.

IR (neat, cm^{-1}): $\tilde{\nu}$ = 2952, 1736, 1708, 1562, 1437, 1245, 701.

HRMS (ESI): *m*/*z* calcd for $C_{18}H_{21}N_2O_5$: 345.1445 [*M*+H]$^+$; found: 345.1450.

Compounds 5d/5d'/5d": Methyl 1-(2-methoxy-2-oxoethyl)-5-oxo-5,6,6a,7,8,9,10,10a-octahydro-1*H*-pyrazolo[1,2-*a*]cinnoline-2-carboxylate.

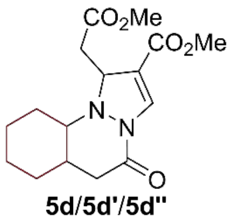

5d/5d'/5d"

Prepared according to the general procedure from **1d** (1.0 mmol) and methyl propiolate (2.5 mmol). Three diastereoisomers (**5d**/**5d'**/**5d"**) were formed (*d.r.* = 4:2:1, determined from the crude reaction mixture). The crude mixture was partially separated by preparative RP-CC (general purification procedure), affording pure major diastereoisomer **5d** (74 mg, 23% yield) and a mixture of diastereoisomers **5d'/5d"** (77 mg, 24% yield, *d.r.* = 2:1).

5d, isolated as a yellow oil.

^{1}H NMR (600 MHz, $CDCl_3$) δ 7.75 (br s, 1H), 4.95 (dd, *J* = 8.5, 3.3 Hz, 1H), 3.73 (s, 3H), 3.70 (s, 3H), 3.18 (dt, *J* = 11.4, 4.2 Hz, 1H), 2.99 (dd, *J* = 16.1, 3.3 Hz, 1H), 2.59 (dd, *J* = 16.1, 8.5 Hz, 1H), 2.54 (dd, *J* = 18.2, 9.0 Hz, 1H), 2.48 (tq, *J* = 8.1, 4.2 Hz, 1H), 2.36 (dd, *J* = 18.2, 8.1 Hz, 1H), 1.83–1.74 (m, 2H), 1.61–1.53 (m, 2H), 1.48–1.47 (m, 1H), 1.35–1.24 (m, 2H), 1.15 (qd, *J* = 12.1, 3.7 Hz, 1H).

$^{13}C\{1H\}$ NMR (600 MHz, $CDCl_3$) δ 171.8, 165.0, 164.3, 133.7, 112.3, 62.4, 59.6, 51.83, 51.73, 40.2, 32.51, 31.77, 29.7, 24.0, 20.02, 19.26.

IR (neat, cm^{-1}): $\tilde{\nu}$ = 2929, 1732, 1701, 1667, 1617, 1402, 1167, 752.

HRMS (ESI): *m*/*z* calcd for $C_{16}H_{23}N_2O_5$: 323.1601 [*M*+H]$^+$; found: 323.1601.

5d'/5d'' (*d.r.* = 2:1).

$^{13}C\{1H\}$ NMR (600 MHz, $CDCl_3$) δ 171.0, 165.7, 164.42, 164.39 (2C), 164.11, 132.60, 132.50, 112.55, 112.39, 65.87, 65.38, 64.63, 61.6, 51.78, 51.73, 51.71 (2C), 41.15, 40.17, 38.54, 36.59, 36.33, 35.0, 32.0, 28.90, 28.71, 26.63, 25.24, 24.54, 24.30, 20.00.

IR (neat, cm^{-1}): $\tilde{\nu}$ = 2932, 1731, 1714, 1662, 1625, 1403, 1209, 1123, 748

HRMS (ESI): *m*/*z* calcd for $C_{16}H_{23}N_2O_5$: 323.1601 [*M*+H]$^+$; found: 323.1600.

Corresponding ^1H NMR resonances for each diastereoizomer:

5d' (major):

^1H NMR (600 MHz, $CDCl_3$) δ 7.75 (d, *J* = 1.6 Hz, 1H), 4.95 (ddt, *J* = 4.8, 3.3, 1.6 Hz, 1H), 3.73 (s, 3H), 3.69 (s, 3H), 2.84 (dd, *J* = 15.5, 3.3 Hz, 1H), 2.72–2.64 (m, 2H), 2.55 (td, *J* = 10.5, 3.8 Hz, 1H), 2.10 (dd, *J* = 19.0, 10.6 Hz, 1H), 2.08–2.06 (m, 1H), 1.95–1.44 (m, 4H), 1.36–1.25 (m, 3H), 1.06 (qd, *J* = 12.6, 3.8 Hz, 1H).

5d'' (minor).

^1H NMR (600 MHz, $CDCl_3$) δ 7.73 (d, *J* = 2.0 Hz, 1H), 4.88 (td, *J* = 4.2, 2.0 Hz, 1H), 3.74 (s, 3H), 3.73 (s, 3H), 3.01 (dd, *J* = 4.8, 4.6 Hz, 1H), 2.88 (dd, *J* = 15.4, 4.4 Hz, 1H), 2.72–2.64 (m, 1H), 2.60 (dd, *J* = 17.4, 7.6 Hz, 1H), 2.24 (dd, *J* = 17.4, 4.8 Hz, 1H), 2.01 (tt, *J* = 11.8, 4.6 Hz, 1H), 1.95–1.64 (m, 6H), 1.52–1.44 (m, 2H).

Compounds 5e/5e': 2-*a*cetyl-8-methyl-1-(2-oxopropyl)-7,8-dihydro-1*H*-pyrazolo[1,2-*a*]pyridazin-5(6*H*)-one.

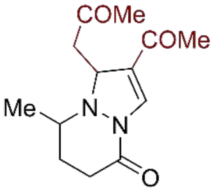

5e/5e'

Prepared according to the general procedure from **1a** (1.0 mmol) and but-3-yn-2-one (3.0 mmol). The reaction mixture was reacted at 50 °C (sand bath) for 48 h. The product was isolated as a mixture of diastereoisomers **5e**/**5e'** by preparative RP-CC (general purification procedure) (55 mg, 22% yield, *d.r.* = 5:1).

$^{13}C\{1H\}$ NMR (600 MHz, $CDCl_3$) δ 206.8, 205.7, 193.26, 193.19, 167.9, 166.8, 134.1, 132.5, 122.2, 63.9, 62.0, 57.5, 53.9, 49.0, 31.53, 30.90, 29.66, 29.16 (2C), 27.55, 27.22, 26.81, 26.78 (2C), 19.2, 12.3.

IR (neat, cm^{-1}): $\tilde{\nu}$ = 2979, 1718, 1660, 1540, 1225, 1164, 946

HRMS (ESI): *m*/*z* calcd for $C_{13}H_{19}N_2O_3$: 251.1390 [*M*+H]$^+$; found: 251.1389.

Corresponding ^{1}H NMR resonances for each diastereoizomer:

5e (major):

^{1}H NMR (600 MHz, $CDCl_3$) δ 7.71 (br s, 1H), 4.83 (dd, *J* = 6.4, 4.5 Hz, 1H), 2.87 (dt, *J* = 8.0, 6.4 Hz, 1H), 2.71 (qd, *J* = 16.0, 4.5 Hz, 2H), 2.58 (dt, *J* = 16.0, 8.9 Hz, 1H), 2.42 (ddd, *J* = 16.1, 8.0, 4.0 Hz, 1H), 2.29 (s, 3H), 2.15 (s, 3H), 1.97–1.91 (m, 1H), 1.77 (dtd, *J* = 13.4, 8.9, 4.0 Hz, 1H), 1.23 (d, *J* = 6.1 Hz, 3H).

5e' (minor):

^{1}H NMR (600 MHz, $CDCl_3$) δ 7.78 (br s, 1H), 4.90 (dd, *J* = 9.4, 2.3 Hz, 1H), 3.67 (qd, *J* = 6.6, 2.6 Hz, 1H), 3.10 (dd, *J* = 17.9, 2.6 Hz, 1H), 2.65 (dd, *J* = 17.9 Hz, 9.4 Hz, 2H), 2.50–2.44 (m, 1H), 2.27 (s, 3H), 2.14 (s, 3H), 1.56–1.51 (m, 1H), 1.27–1.24 (m, 1H), 1.01 (d, *J* = 6.6 Hz, 3H).

Compounds 5f/5f': 8-methyl-1-(2-oxo-2-(pyrrolidin-1-yl)ethyl)-2-(pyrrolidine-1-carbonyl)-7,8-dihydro-1*H*-pyrazolo[1,2-*a*]pyridazin-5(6*H*)-one.

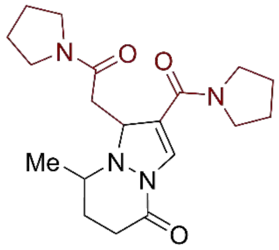

5f/5f'

Prepared according to the general procedure from **1a** (1.0 mmol) and 1-(pyrrolidin-1-yl)prop-2-yn-1-one (3.0 mmol). The reaction mixture was reacted at 50 °C (sand bath) for 96 h. The product was isolated as a mixture of diastereoisomers **5f**/**5f'** by preparative RP-CC (isocratic hold H2O: MeCN = 80:20 for 20 minutes) (184 mg, 51% yield, *d.r.* = 10:1).

^{13}C{1H} NMR (600 MHz, $CDCl_3$) δ 169.26, 168.59, 167.83, 166.0, 162.79, 162.70, 127.6, 125.7, 117.71, 117.53, 66.9, 65.5, 56.9, 53.6, 48.4, 47.13, 47.00, 46.73, 45.76, 45.66, 41.40, 41.17, 29.96, 29.15, 27.44, 27.42, 26.58, 26.33, 26.20, 24.59, 24.56, 24.14, 19.1, 12.3.

IR (neat, cm^{-1}): $\tilde{v}$= 2971, 1617, 1448, 1380, 1223, 726

HRMS (ESI): *m*/*z* calcd for $C_{19}H_{29}N_4O_3$: 361.2234 [*M*+H]$^+$; found: 361.2233.

Corresponding ^{1}H NMR resonances for each diastereoizomer:

5f (major):

^{1}H NMR (600 MHz, $CDCl_3$) δ 7.31 (br s, 1H), 4.97 (dd, *J* = 8.3, 3.0 Hz, 1H), 3.71–3.64 (m, 1H), 3.54–3.39 (m, 8H), 2.82 (h, *J* = 6.2 Hz, 1H), 2.74 (dd, *J* = 14.3, 3.6 Hz, 1H), 2.57 (dt, *J* = 15.4, 9.4 Hz, 1H), 2.50 (dd, *J* = 14.3, 7.8 Hz, 1H), 2.35 (ddd, *J* = 15.4, 7.3, 3.0 Hz, 1H), 1.94–1.89 (m, 5H), 1.88–1.81 (m, 3H), 1.78–1.73 (m, 1H), 1.21 (d, *J* = 6.2 Hz, 3H).

5f' (minor):

^{1}H NMR (600 MHz, $CDCl_3$) δ 7.41 (br s, 1H), 5.09 (dt, *J* = 9.5, 2.1 Hz, 1H), 3.80 (qt, *J* = 6.6, 3.1 Hz, 1H), 2.97 (dd, *J* = 15.8, 2.1 Hz, 1H), 2.46 (dd, *J* = 6.4, 3.1 Hz, 1H), 2.41 (dd, *J* = 8.0, 5.7 Hz, 1H), 2.31–2.24 (m, 2H), 2.02–1.81 (m, 16H), 1.56–1.51 (m, 1H), 1.02 (d, *J* = 6.6 Hz, 3H).

11. Synthesis and characterization of intermediate Int

When **1b** was reacted with a smaller excess of methyl propiolate (1.5 equiv.), the intermediate **Int** was formed in addition to product **5c**/**5c'**. Compound **Int** was isolated and characterized as described below.

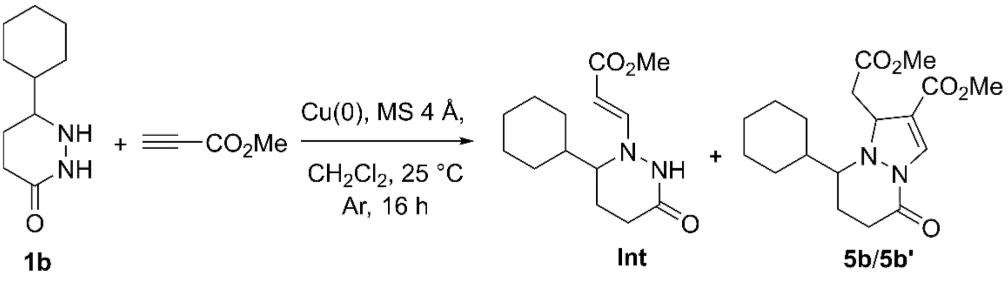

Scheme S12: Formation of intermediate **Int**.

A vial was charged with **1b** (1.0 mmol), methyl propiolate (1.5 mmol), CH_2Cl_2 (5.0 mL), copper(0) (0.7 mmol), molecular sieves (4 Å; 100 mg) and was sealed off with a screw cap with a septum. The reaction mixture was purged with argon and was reacted in the dark at 50 °C (sand bath) for 16 h. Compounds **Int** and **5b**/**5b'** were formed as major diastereoisomers [**Int**/(**5b**/**5b'**) = 2:1]. The crude reaction mixture was purified by CC (PE/EA = 5:2), affording products **Int** (30 mg, 15% yield, R_f = 0.13) and **5b**/**5b'** (84 mg, 24% yield, R_f = 0.18; for characterization see Section 10.3).

Compound Int: Methyl (3-(6-cyclohexyl-3-oxotetrahydropyridazin-1(2*H*)-yl)acrylate, isolated as a yellow oil.

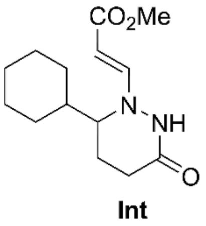

^{1}H NMR (600 MHz, $CDCl_3$) δ 8.59 (br s, 1H), 7.36 (d, *J* = 13.1 Hz, 1H), 4.95 (d, *J* = 13.1 Hz, 1H), 3.64 (s, 3H), 3.27 (br dd, *J* = 8.5, 7.5 Hz, 1H), 2.33–2.23 (m, 3H), 1.85–1.64 (m, 6H), 1.47 (br td, *J* = 11.6, 8.5 Hz, 1H), 1.23–1.12 (m, 3H), 0.93 (pd, *J* = 12.1, 3.5 Hz, 2H).

^{13}C{1H} NMR (600 MHz, $CDCl_3$) δ 174.7, 169.3, 150.4, 88.1, 66.8, 51.0, 40.9, 30.10, 29.31, 29.28, 26.41, 26.22, 25.92, 25.74.

IR (neat, cm^{-1}): $\tilde{\nu}$= 2926, 1683, 1605, 1355, 1146, 727

HRMS (ESI): *m*/*z* calcd for $C_{14}H_{23}N_2O_3$: 267.1703 [*M*+H]$^+$; found: 267.1704.

12. Synthesis and characterization of aldehydes 6

12.1 General procedure for the synthesis of compounds **6**

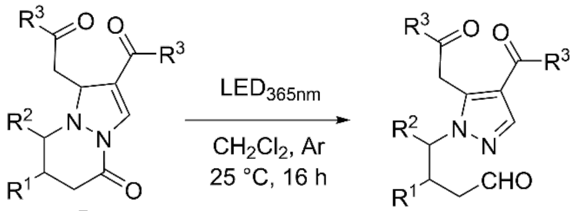

Scheme S13: Synthesis of aldehydes **6**.

Both diastereoisomers **5** and **5'** afforded the same product **6** under the reaction conditions, allowing the use of either isomer as the starting material.

General procedure: a vial was charged with **5** (0.1 mmol) and CH_2Cl_2 (2.0 mL; 0.05 M), was sealed off with a screw cap equipped with a septum, and the resulting solution was purged with argon. The mixture was then irradiated with LED_{365nm} for 16 h at 25 °C. Products **6** were obtained by preparative RP-CC (isocratic hold at 80:20 H_2O/ MeCN for 10 min, then gradient to 70:30 over 30 min (total runtime: 40 minutes)).).

12.2 Characterization data of compounds **6**

Compound 6a: Methyl 5-(2-methoxy-2-oxoethyl)-1-(5-oxopentan-2-yl)-1*H*-pyrazole-4-carboxylate.

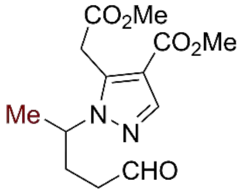

6a

Prepared according to the general procedure from **5a** (0.5 mmol). The crude reaction mixture was purified by preparative RP-CC (general purification procedure), affording **6a** as a colorless oil (134 mg, 95% yield).

Alternatively, compound **6a** was also obtained by irradiating **5a** with LED_{450nm} for 48 h at 25 °C, followed by preparative RP-CC (general procedure), giving **6a** as a colorless oil (130 mg, 93% yield).

^1H NMR (600 MHz, $CDCl_3$) δ 9.65 (s, 1H), 7.93 (s, 1H), 4.35 (ddd, *J* = 11.1, 8.8, 5.5 Hz, 1H), 4.12 (d, *J* = 17.3 Hz, 1H), 4.05 (d, *J* = 17.3 Hz, 1H), 3.81 (s, 3H), 3.72 (s, 3H), 2.39–2.30 (m, 2H), 2.29–2.23 (m, 1H), 2.16–2.10 (m, 1H), 1.47 (d, *J* = 6.6 Hz, 3H).

$^{13}C\{1H\}$ NMR (600 MHz, $CDCl_3$) δ 201.7, 169.3, 164.0, 141.4, 139.2, 112.3, 53.8, 52.7, 51.4, 40.0, 29.9, 28.4, 21.6.

IR (neat, cm^{-1}): $\tilde{\nu}$= 2953, 1740, 1708, 1560, 1231, 772.

HRMS (ESI): *m*/*z* calcd for $C_{13}H_{19}N_2O_5$: 283.1288 [*M*+H]$^+$; found: 283.1288.

Compound 6b: Methyl 1-(1-cyclohexyl-4-oxobutyl)-5-(2-methoxy-2-oxoethyl)-1*H*-pyrazole-4-carboxylate.

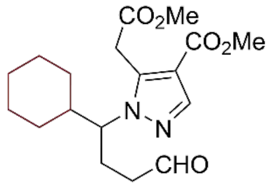

6b

Prepared according to the general procedure from **5b** (0.1 mmol). The reaction proceeded quantitatively, affording **6b** (colorless oil) without the need for further purification (35 mg, >99% yield).

^{1}H NMR (600 MHz, $CDCl_3$) δ 9.61 (s, 1H), 7.94 (s, 1H), 4.27 (d, *J* = 17.2 Hz, 1H), 3.86 (ddd, *J* = 12.2, 8.6, 3.5 Hz, 1H), 3.80 (s, 3H), 3.77 (d, *J* = 17.2 Hz, 1H), 3.70 (s, 3H), 2.33–2.16 (m, 4H), 1.96 (dddd, *J* = 12.6, 7.2, 5.2, 4.2, 3.5 Hz, 1H), 1.84–1.74 (m, 2H), 1.65–1.59 (m, 2H), 1.28–1.20 (m, 1H), 1.14–0.99 (m, 4H), 0.77 (qd, *J* = 12.2, 4.0 Hz, 1H).

^{13}C{1H} NMR (600 MHz, $CDCl_3$) δ 202.0, 169.3, 164.0, 141.35, 140.78, 112.1, 63.2, 52.6, 51.3, 43.3, 39.8, 30.26, 30.25, 29.87, 26.26, 26.08, 25.96, 24.2.

IR (neat, cm^{-1}): $\tilde{v}$ = 2928, 1741, 1708, 1559, 1235 1165, 731.

HRMS (ESI): *m*/*z* calcd for $C_{18}H_{27}N_2O_5$: 351.1914 [*M*+H]$^+$; found: 351.1910.

Compound 6c: 4-(5-(2-oxo-2-(pyrrolidin-1-yl)ethyl)-4-(pyrrolidine-1-carbonyl)-1*H*-pyrazol-1-yl)pentanal.

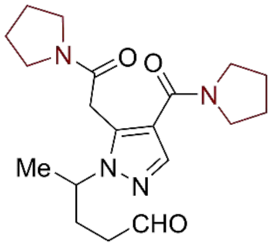

6c

Prepared according to the general procedure from **5f/5f’** (0.1 mmol, *d.r.* = 10:1). The crude reaction mixture was purified by preparative RP-CC (general purification procedure), affording **6c** as a colorless oil (32 mg, 90% yield).

^{1}H NMR (600 MHz, $CDCl_3$) δ 9.65 (s, 1H), 7.71 (s, 1H), 4.45–4.39 (m, 1H), 4.19 (d, *J* = 16.5 Hz, 1H), 4.03 (d, *J* = 16.5 Hz, 1H), 3.68–3.66 (m, 2H), 3.61–3.53 (m, 4H), 3.42 (t, *J* = 6.7 Hz, 2H), 2.42–2.30 (m, 3H), 2.14–2.08 (m, 1H), 1.97 and 1.85 (2p, 1:1, *J* = 6.7 Hz, 4H), 1.91 (br sextet, *J* = 6.1 Hz, 4H), 1.47 (d, *J* = 6.7 Hz, 3H).

^{13}C{1H} NMR (600 MHz, $CDCl_3$) δ 202.2, 166.7, 164.0, 139.6, 138.3, 115.3, 53.5, 49.0, 46.92, 46.52, 46.14, 40.2, 30.6, 28.4, 26.69, 26.25, 24.50, 24.33, 21.7.

IR (neat, cm^{-1}): $\tilde{v}$ = 2972, 1719, 1634, 1594, 1417, 728.

HRMS (ESI): *m*/*z* calcd for $C_{19}H_{29}N_4O_3$: 361.2234 [*M*+H]$^+$; found: 361.2231.

Compounds 6d/6d’: 4-(5-(2-oxo-2-(pyrrolidin-1-yl)ethyl)-4-(pyrrolidine-1-carbonyl)-1*H*-pyrazol-1-yl)pentanal.

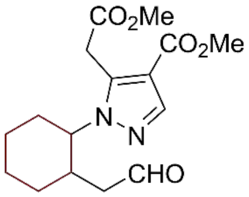

6d/6d’

Prepared according to the general procedure from **5d’/5d’’** (0.1 mmol). The product was isolated as a mixture of diastereoisomers **6d/6d’** by preparative RP-CC (general purification procedure) (29 mg, 90% yield, *d.r.* = 1:1).

Due to similar concentrations of both diastereoisomers in the racemic mixture **6d/6d’**, individual assignment of ^{1}H resonances was not possible.

^{1}H NMR (600 MHz, $CDCl_3$) δ 9.47–9.46 (m, 1H), 7.93 and 7.85 (2s, 1:1, 1H), 4.33 and 3.87 (p and ddd, 1:1, J_p = 4.3 Hz, J_{ddd} = 10.1, 7.4, 4.2 Hz, 1H), 4.21 (d, J = 17.2 Hz, 1:1, 1H), 4.03 (dd, J = 17.2, 3.2 Hz, 1:1, 1H), 3.81 and 3.80 (2s, 1:1, 3H), 3.73 and 3.72 (2s, 1:1, 3H), 2.73 (dtd, J = 16.1, 8.2, 4.2 Hz, 1H), 2.69–2.62 (m, 2H), 2.40 (A) and 2.37 (B) (2dd, 1:1, J_A = 8.1, 1.4 Hz, J_B = 8.5, 1.4 Hz, 1H), 2.21–2.13 (m, 1H), 2.14 and 2.13 (2dd, J = 16.7, 1.8 Hz, 1H), 2.05 and 2.04 (2dd, 1:1, J = 16.7, 2.4 Hz, 1H), 1.95–1.25 (m, 4H).

$^{13}C\{^{1}H\}$ NMR (600 MHz, $CDCl_3$) δ 201.48, 201.00, 169.33, 169.21, 164.04, 163.95, 141.37, 140.44, 139.39, 139.36, 112.62, 112.30, 61.9, 58.3, 52.73, 52.71, 51.37, 51.33, 47.0, 43.2, 37.6, 33.65, 33.44, 32.0, 30.27, 30.05, 29.61, 28.31, 25.55, 25.43, 23.9, 21.8.

IR (neat, cm^{-1}): $\tilde{\nu}$ = 2936, 140, 1708, 1561, 1252, 1197, 777.

HRMS (ESI): m/z calcd for $C_{16}H_{23}N_2O_5$: 323.1601 $[M+H]^+$; found: 323.1600.

Compound 6e: Methyl 5-(2-methylprop-1-en-1-yl)-1-(5-oxopentan-2-yl)-1H-pyrazole-4-carboxylate.

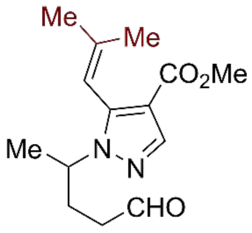

6e

Prepared according to the general procedure from **2k** (0.2 mmol). The crude reaction mixture was purified by preparative RP-CC (general purification procedure), affording **6e** as a colorless oil (40 mg, 75% yield).

^{1}H NMR (500 MHz, $CDCl_3$) δ 9.63 (br s, 1H), 7.94 (s, 1H), 5.92–1.25 (m, 1H), 4.36–4.30 (m, 1H), 3.78 (s, 3H), 2.25–2.15 (m, 3H), 2.11–2.04 (m, 1H), 1.96 (br d, J = 1.5 Hz, 3H), 1.59 (br d, J = 1.4 Hz, 3H), 1.46 (d, J = 6.7 Hz, 3H).

$^{13}C\{^{1}H\}$ NMR (500 MHz, $CDCl_3$) δ 201.1, 163.8, 145.2, 143.2, 141.7, 111.96, 111.19, 53.7, 51.2, 40.3, 28.7, 25.7, 21.0, 20.5.

IR (neat, cm^{-1}): $\tilde{\nu}$ = 2979, 1712, 1540, 1407, 1222, 773.

HRMS (ESI): m/z calcd for $C_{14}H_{21}N_2O_3$: 265.1547 $[M+H]^+$; found: 265.1548.

Compound 6f: Methyl 5-(cyclohex-1-en-1-yl)-1-(5-oxopentan-2-yl)-1H-pyrazole-4-carboxylate.

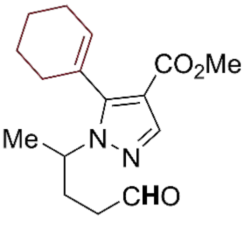

6f

Prepared according to the general procedure from **2n** (0.2 mmol). The reaction proceeded quantitatively, affording **6f** (colorless oil) without the need for further purification (58 mg, >99% yield).

^{1}H NMR (600 MHz, $CDCl_3$) δ 9.64 (s, 1H), 7.89 (s, 1H), 5.73–5.71 (m, 1H), 4.44–4.38 (m, 1H), 3.78 (s, 3H), 2.34–2.17 (m, 6H), 2.10–2.04 (m, 2H), 1.78 (dt, J = 5.9, 5.9 Hz, 2H), 1.72 (dt, J = 5.4, 5.4 Hz, 2H), 1.45 (d, J = 6.6 Hz, 3H).

$^{13}C\{^{1}H\}$ NMR (600 MHz, $CDCl_3$) δ 201.2, 163.7, 148.5, 141.5, 131.9, 128.2, 111.0, 53.5, 51.3, 40.6, 29.28, 28.48, 25.6, 22.67, 22.11, 21.74.

IR (neat, cm^{-1}): $\tilde{\nu}$ = 2934, 1713, 1539, 1406, 1222, 1196, 769.

HRMS (ESI): m/z calcd for $C_{16}H_{23}N_2O_3$: 291.1703 $[M+H]^+$; found: 291.1707.

13. Synthesis and characterization data of oxidized products 7

13.1 General procedure for the synthesis of compounds **7**

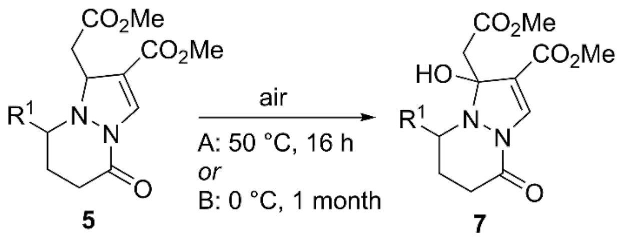

Scheme S14: Synthesis of aldehydes **7** by procedure A and B.

Procedure A: an ACE vial was charged with **5** (0.2 mmol) and CH_2Cl_2 (4.0 mL; 0.05 M), and was sealed off with a screw cap. The resulting solution was reacted at 50 °C (sand bath) under air for 72 h. Products **7** were isolated by CC (DCM/MeOH = 19:1) or preparative RP-CC (isocratic hold at 80:20 H_2O/ MeCN for 15 min, then gradient to 60:40 over 15 min (total runtime: 30 minutes)).

Procedure B: a vial was charged with **5** (0.2 mmol) and left solvent-free to oxidize in the dark at 0 °C over 1 month. Products **7** were obtained either without further purification or by isolation via preparative RP-CC using the same conditions as described in Procedure A.

13.2 Characterization data of compounds **7**

Compound 7a: Methyl 1-hydroxy-1-(2-methoxy-2-oxoethyl)-8-methyl-5-oxo-5,6,7,8-tetrahydro-1*H*-pyrazolo[1,2-*a*]pyridazine-2-carboxylate.

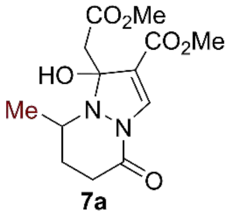

7a

Prepared according to the Procedure A from **5a**/**5a'** (1.3:1, 0.2 mmol). The crude reaction mixture was purified by CC (DCM:MeOH = 19:1), affording a single diastereoisomer **7a** as a colorless oil (35 mg, 58% yield, R_f = 0.20). Additionally, 20% of **5a**/**5a'** was recovered (1.3:1, R_f = 0.25).

^{1}H NMR (600 MHz, $CDCl_3$) δ 7.95 (s, 1H), 4.44–4.39 (m, 1H), 4.19 (d, *J* = 17.2 Hz, 1H), 4.07 (d, J = 17.2 Hz, 1H), 3.81 (s, 3H), 3.70 (s, 3H), 2.40–2.33 (m, 1H), 2.28–2.22 (m, 1H), 2.12 (td, *J* = 8.8, 6.6 Hz, 1H), 2.09–2.05 (m, 1H), 1.47 (d, *J* = 6.6 Hz, 3H).

^{13}C{1H} NMR (600 MHz, $CDCl_3$) δ 178.0, 169.2, 164.1, 141.4, 139.2, 112.2, 53.7, 52.7, 51.4, 30.95, 30.00, 29.96, 21.3.

IR (neat, cm^{-1}): $\tilde{v}$= 2954, 1740, 1707, 1561, 1231, 1168, 771.

HRMS (ESI): *m*/*z* calcd for $C_{13}H_{19}N_2O_6$: 299.1238 [*M*+H]$^+$; found: 299.1231.

Compound 7b: Methyl 8-cyclohexyl-1-hydroxy-1-(2-methoxy-2-oxoethyl)-5-oxo-5,6,7,8-tetrahydro-1*H*-pyrazolo[1,2-*a*]pyridazine-2-carboxylate.

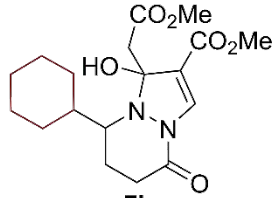

7b

Prepared according to the Procedure A from a single diastereoisomer **5b** (0.1 mmol). The crude reaction mixture was purified by preparative RP-CC (general purification procedure), affording a single diastereoisomer **7b** as a colorless oil (29 mg, 80% yield).

^{1}H NMR (600 MHz, DMSO-d$_6$) δ 7.89 (s, 1H), 4.16 (d, *J* = 17.4 Hz, 1H), 4.09 (d, *J* = 17.4 Hz, 1H), 4.18–4.07 (m, 1H), 3.70 (s, 3H), 3.60 (s, 3H), 2.08–2.02 (m, 1H), 1.88–1.70 (m, 2H), 1.78–1.69 (m, 2H), 1.58–1.51 (m, 3H), 1.41–1.36 (m, 1H), 1.23–1.16 (m, 1H), 1.09–0.93 (m, 4H), 0.81–0.74 (m, 1H).

^{13}C{1H} NMR (600 MHz, DMSO-d$_6$) δ 167.0, 163.3, 141.07, 140.19, 110.6, 62.8, 51.92, 50.91, 42.1, 40.06, 33.4, 29.97, 29.37, 29.11, 28.85, 25.92, 25.65, 25.52.

IR (neat, cm^{-1}): $\tilde{v}$= 2928, 1709, 1559, 1436, 1267, 1197, 997.

HRMS (ESI): *m*/*z* calcd for $C_{18}H_{27}N_2O_6$: 367.1864 [*M*+H]$^+$; found: 367.1872.

Compound 7c: Methyl 1-hydroxy-1-(2-methoxy-2-oxoethyl)-5-oxo-8-phenyl-5,6,7,8-tetrahydro-1*H*-pyrazolo[1,2-*a*]pyridazine-2-carboxylate.

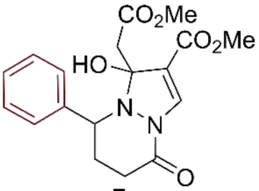

7c

Prepared according to the Procedure B from a single diastereoiosmer **5c** (0.2 mmol). The reaction proceeded quantitatively, affording a single diastereoisomer **7c** (colorless oil) without the need for further purification (72 mg, >99% yield).

^{1}H NMR (600 MHz, CDCl$_3$) δ 8.00 (s, 1H), 7.31 (br t, *J* = 7.2 Hz, 3H), 7.21 (br d, *J* = 7.2 Hz, 2H), 5.42 (dd, *J* = 9.9, 5.2 Hz, 1H), 4.24 (d, *J* = 17.2 Hz, 1H), 3.87 (d, *J* = 17.2 Hz, 1H), 3.80 (s, 3H), 3.55 (s, 3H), 2.88–2.82 (m, 1H), 2.50–2.33 (m, 3H).

^{13}C{1H} NMR (600 MHz, CDCl$_3$) δ 177.5, 168.9, 163.9, 141.09, 140.09, 139.65, 129.01, 128.34, 126.6, 113.1, 61.9, 52.6, 51.4, 30.74, 30.32, 30.21.

IR (neat, cm^{-1}): $\tilde{v}$= 2953, 1708, 1437, 1241, 1158, 700.

HRMS (ESI): *m*/*z* calcd for $C_{18}H_{20}N_2NaO_6$: 383.1214 [*M*+Na]$^+$; found: 383.1215.

14. Optical properties of compounds 2, 3 and 5

14.1 Optical properties of compounds **2**

Table S3: Absorption and emission maxima of pyrazolo[1,2-*a*]pyridazinones **2**.

Compound	**2a**	**2a'**	**2b**	**2b'**	**2c**	**2c'**
λ_{abs} [nm]	325	343	324	320	328	337
λ_{em}[nm]	522	524	525	522	528	524
Stokes shift $\lambda_{em} - \lambda_{abs}$ [nm]	197	181	201	202	200	196

Compound	**2d**	**2d'**	**2e/2e'**	**2f**	**2f'**
λ_{abs} [nm]	322	320	352	332	342
λ_{em}[nm]	521	525	473	520	521
Stokes shift $\lambda_{em} - \lambda_{abs}$ [nm]	199	205	121	188	179

Compound	**2g**	**2g'**	**2h/2h'**	**2i**	**2j/2j'/2j''**
λ_{abs} [nm]	307	311	337	326	307
λ_{em}[nm]	505	511	519	513	518
Stokes shift $\lambda_{em} - \lambda_{abs}$ [nm]	198	200	182	187	211

Compound	**2k**	**2l**	**2l'**	**2m/2m'**	**2n**
λ_{abs} [nm]	333	329	316	344	333
λ_{em}[nm]	532	514	497	525	526
Stokes shift $\lambda_{em} - \lambda_{abs}$ [nm]	199	185	181	181	193

Absorption maxima (λ_{abs}) are reported as local maxima above 300 nm. For diastereoisomeric mixtures the strongest absorption maximum above 300 nm is reported.

Compounds **2** exhibit a large Stokes shift, averaging at 190 nm.

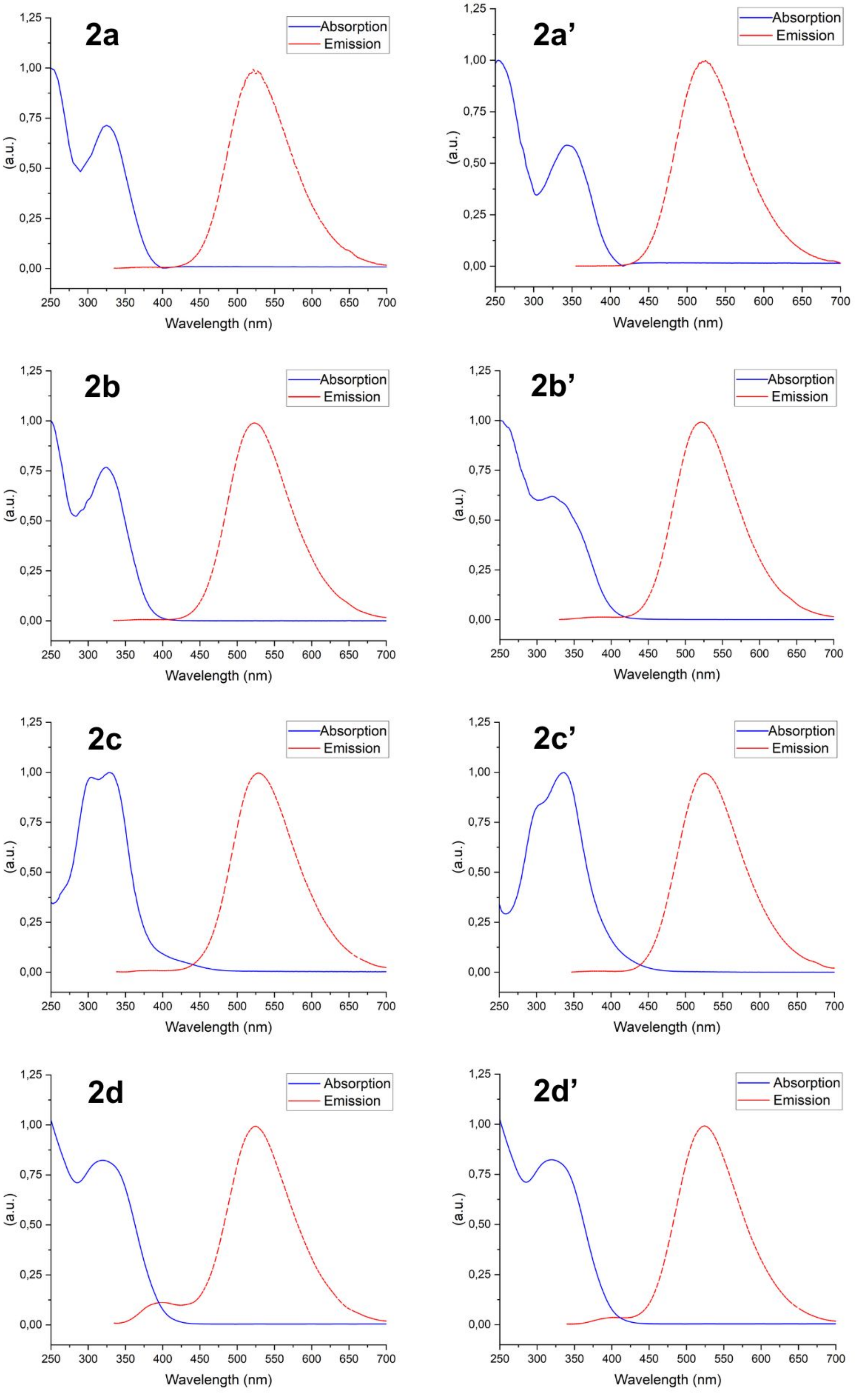

Figure S5. **2a–2d'**: Normalized absorption and fluorescence spectra of **2**.

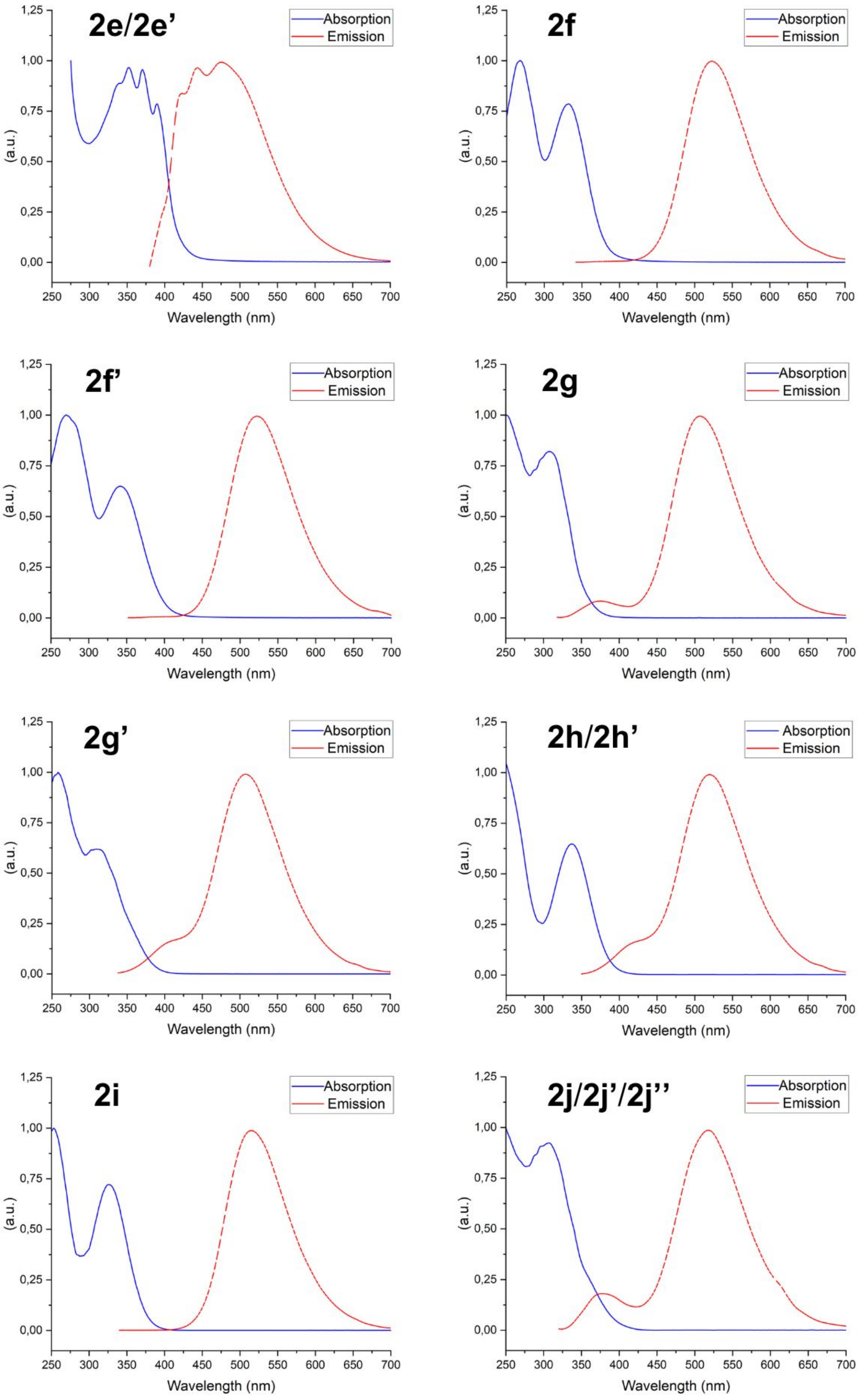

Figure S6. **2e/2e'–2j/2j'/2j''**: Normalized absorption and fluorescence spectra of **2**.

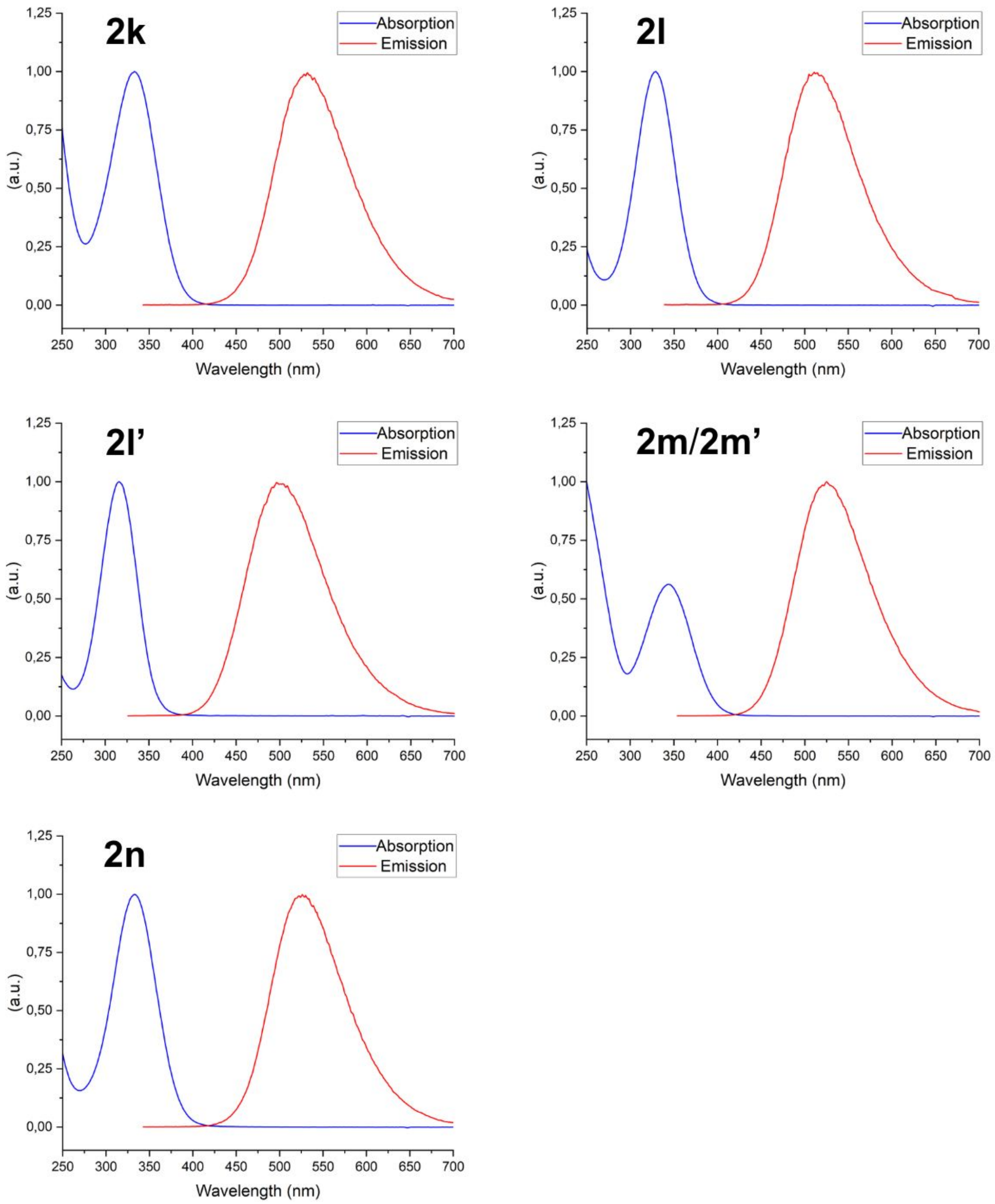

Figure S7. **2k–2n**: Normalized absorption and fluorescence spectra of **2**.

14.2 Optical properties of compound **3a**

Compound	**3a**
λ_{abs} [nm]	334
λ_{em}[nm]	525
Stokes shift $\lambda_{em} - \lambda_{abs}$ [nm]	191

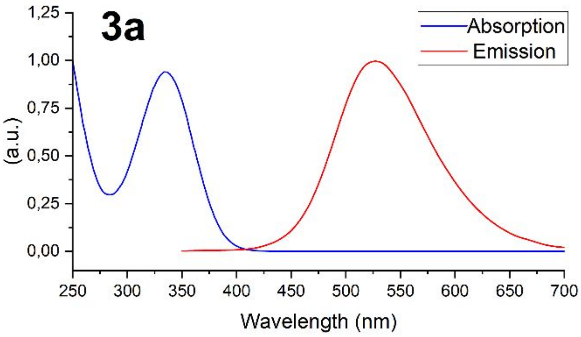

Figure S8. Left: absorption and emission maximum of 1,2-diazepine **3a**. Right: normalized absorption and fluorescence spectrum of **3a**.

14.3 Absorption spectra of **2a**, **3a** and **4a**

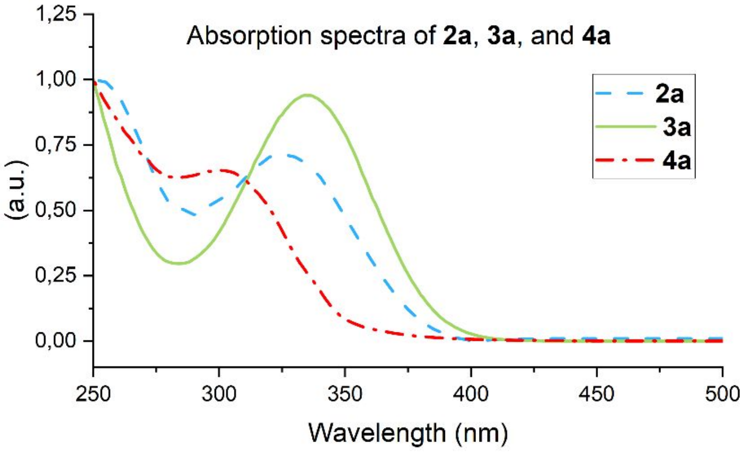

Figure S9. Comparison between normalized UV-VIS absorption spectrum of compounds **2a**, **3a**, and **4a**. All spectra are normalized to an absorbance of 1.0 (a.u.) at 250 nm.

14.4 Absorption spectra of *E*-**2a-OH** and *Z*-**2a-OH**

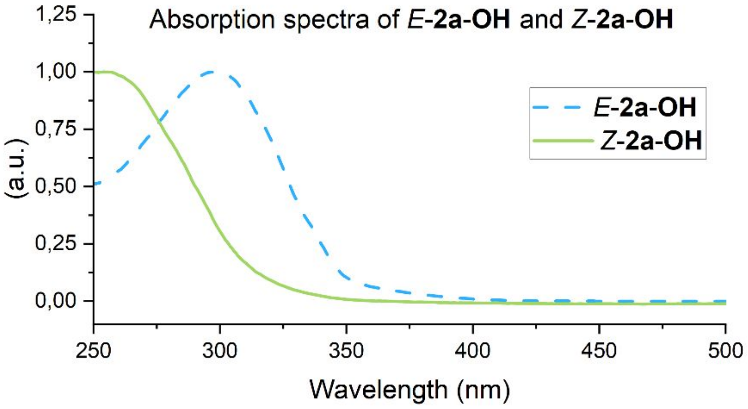

Figure S10. Comparison between normalized UV-VIS absorption spectrum of compounds *E*-**2a-OH** and *Z*-**2a-OH** (scaled to 1.0 (a.u.) at respective absorption maximum).

14.5 Optical properties of compounds **5**

Table S4: Absorption and emission maxima of pyrazolo[1,2-*a*]pyridazinones **5**.

Compound	**5a/5a'**	**5b**	**5b’**	**5d**	**5d’/5d’’**	**5f/5f'**
λ_{abs} [nm]	329	327	350	339	336	314
λ_{em}[nm]	519	527	522	521	506	527
Stokes shift $\lambda_{em} - \lambda_{abs}$ [nm]	190	200	172	182	170	213

Absorption maxima (λ_{abs}) are reported as local maxima above 300 nm. For diastereoisomeric mixtures, the strongest absorption maximum above 300 nm is reported.

Compounds **5** exhibit a large Stokes shift, averaging at <u>188 nm</u>.

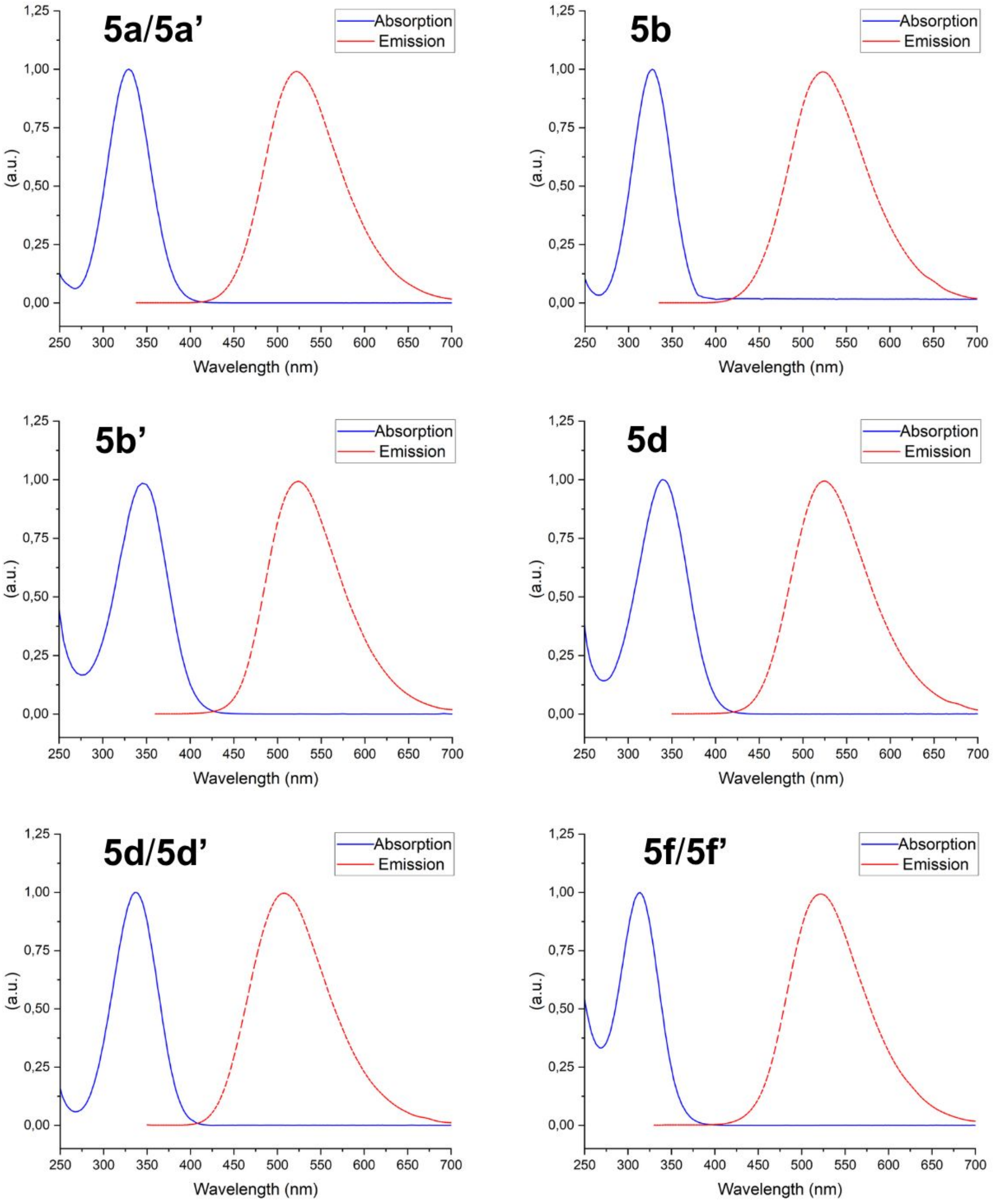

Figure S11. **5a**/**5a'**–**5f**/**5f'**: Normalized absorption and fluorescence spectra of **5**.

15. Investigating photoisomerization of *E*-2a-OH to *Z*-2a-OH

Structure optimization and TDDFT calculations were performed using the AMS2025 by SCM[11] or Gaussian 16 package, revision C.01.[12] The structure was optimized with the B3LYP(D4) functional and a triple-zeta polarized (TZ2P) Slater-type basis set or def2-TZVP basis set. The convergence criteria (maximum force threshold –4.5×10^{-4}; RMS force threshold –3×10^{-4}; maximum displacement threshold –1.8×10^{-3}; RMS displacement threshold –1.2×10^{-3}) were used for the ground and excited state optimizations. Solvation of the molecule was modelled using the implicit conductor-like screening model (COSMO)[13]. Vibrational analysis was performed to verify the presence of a local minimum. Excited state energies were obtained at the same level of theory, without a frozen core and including COSMO dichloromethane solvation. The Tamm-Dancoff approximation was applied for the calculation of excited state energies within the TDDFT framework.[14] To gain further insight into the nature of the excited states, an analysis of the natural transition orbitals (NTOs) was performed based on the TD-DFT results to provide a compact orbital representation for the electronic transition density matrix. For the excited states, the geometries of the lowest triplet excited state (T1) and phantom triplet geometry were optimized using SMD/(UDFT)/B3LYP-D3/def2-TZVP.

a) b)

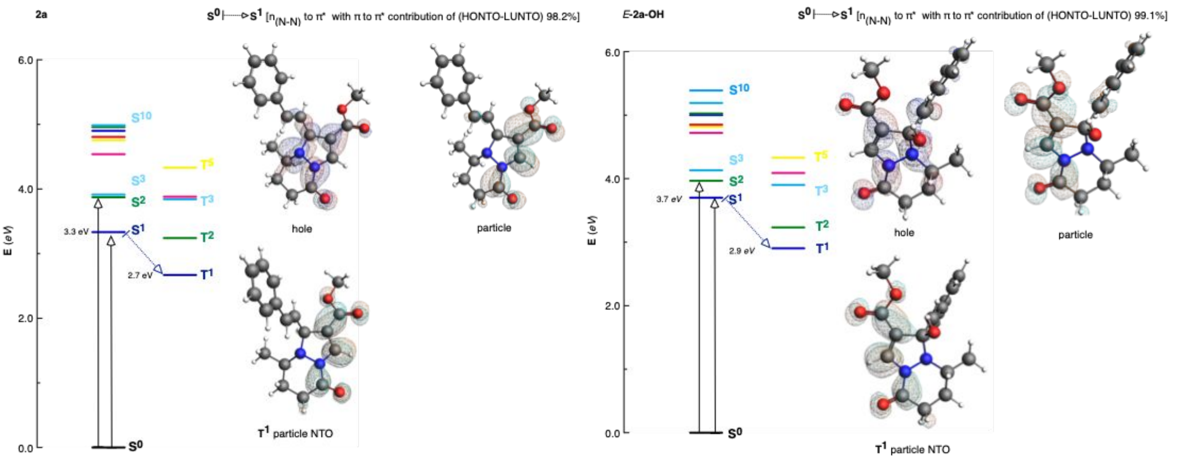

Figure S12. Energy levels (eV) of singlet and triplet states and the corresponding NTO pairs (hole and particle) for the S^0 to S^1 transitions and T^1 particle NTO for a) **2a** and b) for *E*-**2a-OH**, calculated at the B3LYP(D4)/TZ2P/COSMO(dichloromethane) level of theory.

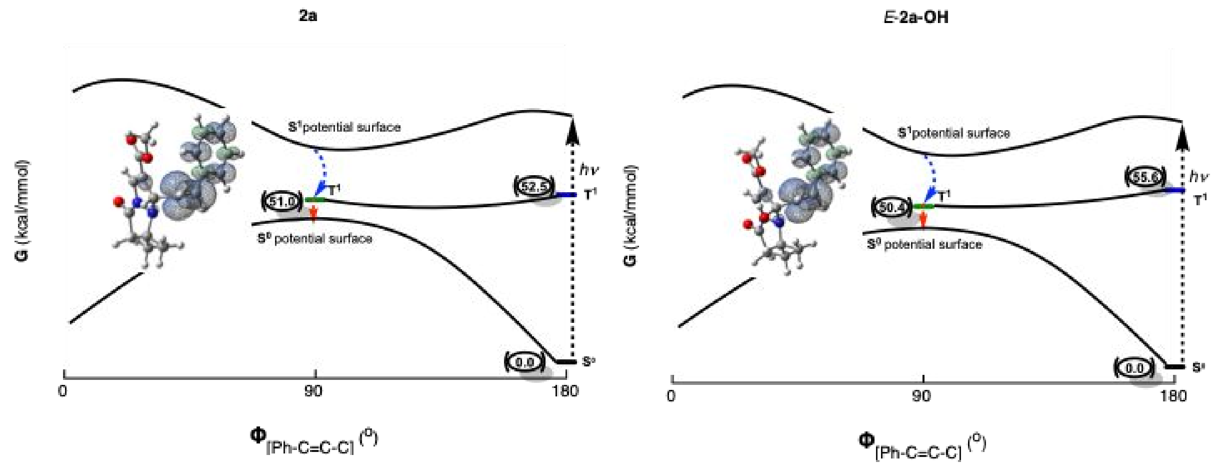

Figure S13. (U)DFT studies of *E*/*Z* isomerization of **2a** and *E*-**2a-OH**. The Gibbs free energies (in kcal/mol) at the optimized structures S^0, T^1, and "phantom" T^1 ($\phi_{[Ph\text{-}C=C\text{-}C]}$ = 90°). The structure given on diagram shows the corresponding spin density of "phantom" triplet state. All energies were calculated at the B3LYP(D3)/def2-TZVP(SMD-dichloromethane).

Irradiation of **2a** at 365 nm results in slow formation of tricyclic product **4a** (via **3a**), with product accumulation becoming evident only after 8 h and full conversion after 16 h. In contrast, *E*-**2a-OH** undergoes rapid *E* to *Z* photoisomerization, affording 85% *Z*-**2a-OH** within only 20 minutes. *Z*-**2a-OH** exhibits a blue-shifter absorption maximum at 254 nm and with no absorption at 365 nm compared to *E*-**2a-OH** (λ_{max} = 298 nm) and **2a** (λ_{max} = 325 nm). Due to the rapid accumulation of *Z*-**2a-OH** and its lack of excitation at 365 nm, no diazepine **3** or tricyclic products **4** are formed from *E*-**2a-OH**.

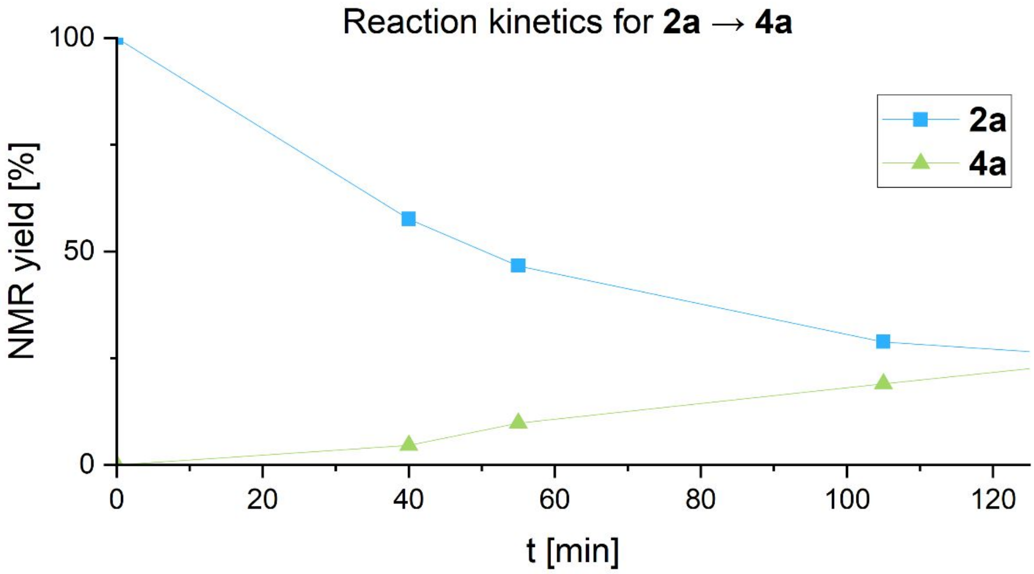

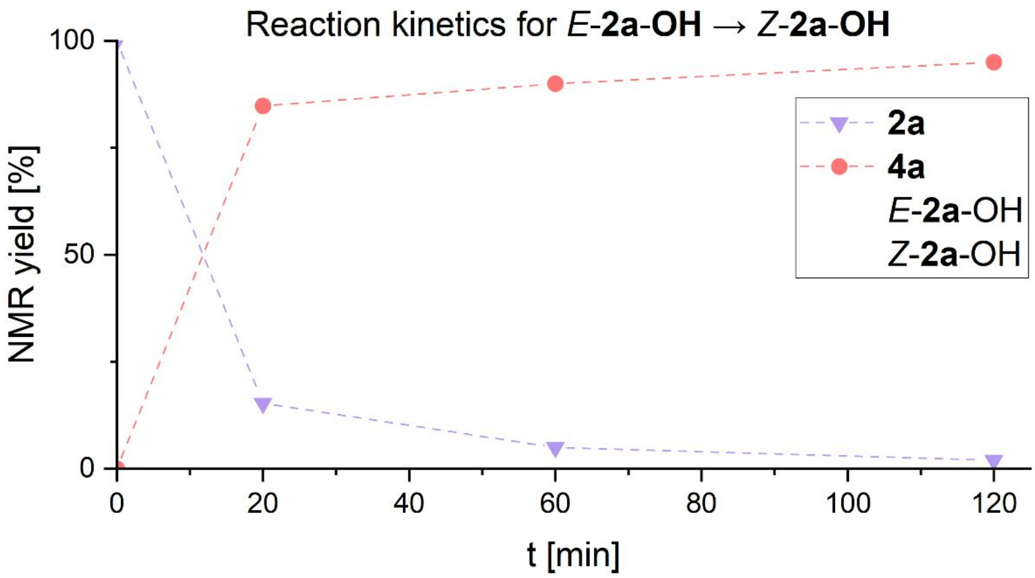

Figure S14. Comparison of reaction kinetics for transformation of **2a** to **4a** (via **3a**) and *E*-**2a-OH** to *Z*-**2a-OH.**

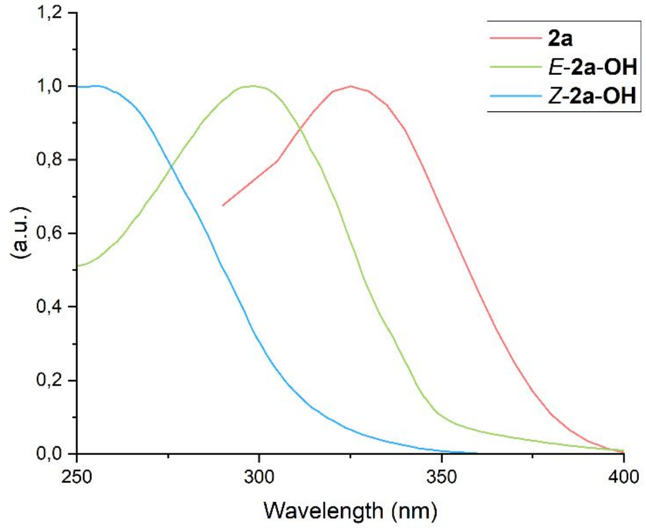

Figure S15. Comparison between normalized UV-VIS absorption spectrum of compounds **2a**, *E*-**2a-OH** and *Z*-**2a-OH** (scaled to 1.0 (a.u.) at respective absorption maximum). The spectrum of **2a** is truncated below 290 nm for clearer comparison of the longest-wavelength absorption maxima for all three compounds.

Coordinates of optimized structure of **2a**

SMD-B3LYP-3D/def2-TZVP at 298.15 K

EE = –1033.578692

EE+ZPE = –1033.226426

Enthalpy = –1033.204259

Free Energy = –1033.279543

0 1

C	-1.85027	-1.69069	1.37135
C	-2.44807	-2.90024	0.65710
C	-3.60814	-2.51773	-0.25955
C	-3.47773	-1.18646	-0.96610
N	-2.46455	-0.36356	-0.52256
N	-1.37845	-0.78229	0.32598
C	-2.30707	0.95431	-0.85399
C	-1.30370	1.51014	-0.16120
C	-0.68931	0.46225	0.75333
O	-4.26302	-0.80810	-1.81309
C	0.80904	0.35618	0.63318
C	1.44921	-0.57474	-0.07003
C	2.89739	-0.71014	-0.23920
C	3.38909	-1.78442	-0.98869
C	4.75227	-1.96521	-1.18130
C	5.65732	-1.07061	-0.62604
C	5.18482	0.00554	0.12126
C	3.82477	0.18398	0.31217
C	-0.70813	-2.10483	2.28669
C	-0.95627	2.92176	-0.23741
O	-0.04555	3.25883	0.71056
C	0.35529	4.63502	0.71631
H	-2.63158	-1.19356	1.97213
H	-2.78492	-3.63172	1.39341

H	-1.64742	-3.36705	0.07893
H	-4.53784	-2.43698	0.31146
H	-3.79011	-3.27463	-1.02270
H	-2.96963	1.40218	-1.57490
H	-0.93609	0.70383	1.80008
H	1.34632	1.13983	1.15068
H	0.84776	-1.32393	-0.57293
H	2.68658	-2.48355	-1.42628
H	5.10659	-2.80425	-1.76633
H	6.72075	-1.20622	-0.77378
H	5.88361	0.70943	0.55524
H	3.47902	1.02888	0.89264
H	0.07964	-2.60308	1.72219
H	-1.08839	-2.79536	3.04024
H	-0.26471	-1.25527	2.80391
H	-0.50231	5.28257	0.89506
H	0.80821	4.90372	-0.23730
H	1.07794	4.72663	1.52262
O	-1.41710	3.71863	-1.02191

Coordinates of optimized structure of **2a** (T^1)

SMD-(U)B3LYP-3D/def2-TZVP at 298.15 K

EE = –1033.490646

EE+ZPE –1033.141887

Enthalpy = –1033.119177

Free Energy = –1033.195808

0 3

C	-1.92384	-1.64246	1.41810
C	-2.40199	-2.88175	0.67013
C	-3.52612	-2.54605	-0.30315
C	-3.36107	-1.24296	-1.04000
N	-2.39519	-0.34656	-0.56188

N	-1.46382	-0.68655	0.40634
C	-2.21240	0.92681	-1.02237
C	-1.25284	1.56629	-0.17388
C	-0.73064	0.52861	0.77243
O	-4.06845	-0.88734	-1.97247
C	0.76745	0.37810	0.63150
C	1.37715	-0.62162	-0.00352
C	2.81800	-0.76122	-0.21727
C	3.31630	-1.98178	-0.68638
C	4.67579	-2.17181	-0.89257
C	5.56853	-1.13890	-0.63763
C	5.08748	0.08572	-0.18131
C	3.73077	0.27430	0.02363
C	-0.81050	-1.97614	2.39904
C	-0.89215	2.93887	-0.21638
O	0.00761	3.28525	0.76625
C	0.39696	4.65916	0.76806
H	-2.76811	-1.19775	1.96788
H	-2.73736	-3.63154	1.38788
H	-1.54616	-3.30182	0.13645
H	-4.48024	-2.47608	0.23124
H	-3.65792	-3.33128	-1.04908
H	-2.79219	1.30043	-1.84136
H	-0.97604	0.79889	1.80926
H	1.31846	1.20878	1.04866
H	0.76812	-1.42743	-0.39813
H	2.62340	-2.78961	-0.88963
H	5.03718	-3.12543	-1.25543
H	6.62886	-1.28094	-0.80038
H	5.77568	0.89991	0.00668

H	3.37374	1.23939	0.35744
H	0.03521	-2.43706	1.89068
H	-1.19718	-2.67851	3.13765
H	-0.44785	-1.09835	2.93162
H	-0.46612	5.30733	0.92180
H	0.87077	4.92937	-0.17614
H	1.10119	4.76600	1.58985
O	-1.34061	3.75189	-1.01486

Coordinates of optimized structure of **2a** (“phantom”-T^1)

SMD-(U)B3LYP-3D/def2-TZVP at 298.15 K

EE = –1033.493182

EE+ZPE –1033.144998

Enthalpy = –1033.122529

Free Energy = –1033.198304

0 3

C	-2.95680	-0.19371	1.19160
C	-3.81906	-1.23367	0.48006
C	-4.08441	-0.86401	-0.97735
C	-2.93608	-0.19986	-1.70431
N	-1.89831	0.24431	-0.91255
N	-1.69346	-0.12992	0.46223
C	-0.86976	1.04455	-1.32463
C	-0.08827	1.39775	-0.29417
C	-0.63193	0.77987	0.98760
O	-2.94744	0.01854	-2.89978
C	0.36024	0.00363	1.80405
C	0.80663	-1.32358	1.41098
C	1.91717	-1.60622	0.58922
C	2.18664	-2.93891	0.18057
C	3.25629	-3.23060	-0.63948
C	4.10506	-2.21406	-1.08352

C	3.86910	-0.89793	-0.68404
C	2.80294	-0.59421	0.13766
C	-2.71401	-0.58274	2.64208
C	1.01818	2.33404	-0.40547
O	1.50774	2.66410	0.81802
C	2.60504	3.58541	0.81381
H	-3.46205	0.78754	1.16044
H	-4.76583	-1.35053	1.00998
H	-3.29312	-2.18937	0.53709
H	-4.90977	-0.14915	-1.04769
H	-4.38502	-1.72938	-1.56807
H	-0.79885	1.32400	-2.36213
H	-1.06340	1.57386	1.61719
H	0.76642	0.49020	2.68422
H	0.20889	-2.17415	1.73119
H	1.52703	-3.72961	0.51693
H	3.43673	-4.25392	-0.94375
H	4.94043	-2.44588	-1.73068
H	4.52595	-0.10666	-1.02233
H	2.63728	0.42374	0.45836
H	-2.20063	-1.54330	2.69998
H	-3.67135	-0.67053	3.15705
H	-2.11533	0.15667	3.17210
H	2.30982	4.53090	0.36038
H	3.44594	3.17486	0.25549
H	2.87248	3.72767	1.85722
O	1.46158	2.78710	-1.43557

Coordinates of optimized structure of *E*-**2a-OH**

SMD-B3LYP-3D/def2-TZVP at 298.15 K

EE = –1108.839452

EE+ZPE = –1108.483335

Enthalpy = –1108.460010

Free Energy = –1108.536567

0 1

C	-1.73533	-1.79052	1.24285
C	-2.36576	-2.95097	0.47735
C	-3.61900	-2.52262	-0.27710
C	-3.51239	-1.19128	-0.97991
N	-2.47634	-0.37266	-0.56555
N	-1.35975	-0.78550	0.23997
C	-2.28351	0.91577	-0.96547
C	-1.23429	1.45834	-0.32612
C	-0.68203	0.44940	0.66343
O	-4.31030	-0.80526	-1.80923
C	0.82012	0.29739	0.61384
C	1.46525	-0.53817	-0.19589
C	2.91367	-0.71563	-0.30619
C	3.41222	-1.58680	-1.28096
C	4.77705	-1.79016	-1.43456
C	5.67575	-1.12611	-0.61032
C	5.19595	-0.25926	0.36852
C	3.83427	-0.05722	0.51987
O	-1.08778	0.79150	2.00501
C	-0.52710	-2.25838	2.04050
C	-0.82259	2.84658	-0.45249
O	0.01414	3.20221	0.56479
C	0.49498	4.55333	0.52556
H	-2.47346	-1.36253	1.93319
H	-2.61227	-3.75145	1.17632
H	-1.61985	-3.34469	-0.21747
H	-4.46179	-2.41741	0.41336
H	-3.92476	-3.25716	-1.02189

H	-2.95132	1.35351	-1.68760
H	1.35295	0.96945	1.27308
H	0.86645	-1.16454	-0.84805
H	2.71450	-2.10534	-1.92743
H	5.13776	-2.46740	-2.19805
H	6.74045	-1.28199	-0.72517
H	5.89008	0.25885	1.01796
H	3.48175	0.61430	1.29131
H	-0.82902	1.71079	2.14923
H	0.22656	-2.69970	1.38871
H	-0.07200	-1.44273	2.59599
H	-0.85559	-3.01609	2.75342
H	-0.33526	5.25587	0.58422
H	1.04701	4.73175	-0.39607
H	1.14745	4.65811	1.38807
O	-1.17363	3.61948	-1.31077

Coordinates of optimized structure of *E*-**2a-OH** (T^1)

SMD-(U)B3LYP-3D/def2-TZVP at 298.15 K

EE = –1108.746861

EE+ZPE –1108.393933

Enthalpy = –1108.370259

Free Energy = –1108.447927

0 3

C	-1.82172	-1.70833	1.36705
C	-2.28268	-2.96409	0.63544
C	-3.41992	-2.66592	-0.33043
C	-3.20789	-1.43139	-1.16173
N	-2.24645	-0.51318	-0.71478
N	-1.39768	-0.74243	0.34682
C	-2.00158	0.70259	-1.30748
C	-1.18350	1.44769	-0.41734

C	-0.71559	0.51988	0.66960
O	-3.85180	-1.14888	-2.16158
C	0.78166	0.39708	0.54406
C	1.43388	-0.62846	-0.01324
C	2.87409	-0.73089	-0.21969
C	3.41248	-1.95867	-0.62569
C	4.77686	-2.11550	-0.82131
C	5.63604	-1.04259	-0.61865
C	5.11621	0.18808	-0.22459
C	3.75442	0.34437	-0.03036
O	-1.11604	0.93436	1.97198
C	-0.68833	-2.02362	2.33392
C	-0.90890	2.84059	-0.47818
O	-0.21204	3.28215	0.64076
C	0.10161	4.67744	0.64638
H	-2.66076	-1.27641	1.92711
H	-2.59657	-3.70598	1.37046
H	-1.42714	-3.38174	0.09843
H	-4.35667	-2.52050	0.21918
H	-3.59571	-3.49509	-1.01695
H	-2.56626	1.00466	-2.16574
H	1.30130	1.28078	0.88849
H	0.85508	-1.48437	-0.34106
H	2.74560	-2.79723	-0.78658
H	5.16941	-3.07432	-1.13449
H	6.70066	-1.15929	-0.77312
H	5.77867	1.03127	-0.07656
H	3.36749	1.31289	0.25645
H	-0.93726	1.88623	2.00874
H	0.14929	-2.49095	1.81710

H	-0.33313	-1.13400	2.84511
H	-1.06401	-2.72376	3.08089
H	-0.80712	5.27803	0.61340
H	0.72197	4.93403	-0.21221
H	0.64268	4.85619	1.57244
O	-1.26029	3.60227	-1.36389

Coordinates of optimized structure of *E*-**2a-OH** (“phantom”-T^1)

SMD-(U)B3LYP-3D/def2-TZVP at 298.15 K

EE = –1108.754560

EE+ZPE –1108.402468

Enthalpy = –1108.378857

Free Energy = –1108.456184

0 3

C	2.88865	-0.41216	-1.13134
C	3.71529	-1.46091	-0.39153
C	4.08885	-1.00216	1.01331
C	2.97497	-0.32698	1.77606
N	1.91496	0.13041	1.01198
N	1.65974	-0.22503	-0.35510
C	0.88100	0.88557	1.47344
C	0.05745	1.23275	0.46965
C	0.62372	0.71192	-0.84350
O	3.00906	-0.12161	2.97165
C	-0.35278	-0.02942	-1.71713
C	-0.86573	-1.33914	-1.35237
C	-2.02719	-1.58875	-0.59242
C	-2.34486	-2.90984	-0.18174
C	-3.46569	-3.16966	0.57864
C	-4.31986	-2.13153	0.95732
C	-4.03722	-0.82640	0.55245
C	-2.91948	-0.55490	-0.20967

O	1.23664	1.78415	-1.57803
C	2.56842	-0.86819	-2.54694
C	-1.05264	2.15706	0.61381
O	-1.46018	2.62463	-0.60459
C	-2.56229	3.54210	-0.57627
H	3.44911	0.53081	-1.17788
H	4.62016	-1.67537	-0.96205
H	3.13147	-2.38349	-0.34604
H	4.89842	-0.26665	0.97105
H	4.45258	-1.82188	1.63244
H	0.83676	1.14383	2.51779
H	-0.65872	0.46577	-2.63205
H	-0.26981	-2.20591	-1.62990
H	-1.68147	-3.71715	-0.46718
H	-3.68239	-4.18469	0.88682
H	-5.19541	-2.33830	1.55815
H	-4.69850	-0.01882	0.83967
H	-2.71728	0.45393	-0.53796
H	0.57191	2.48045	-1.65901
H	2.00802	-1.80381	-2.53578
H	1.99718	-0.11878	-3.08919
H	3.50320	-1.03675	-3.08350
H	-2.30296	4.43362	-0.00700
H	-3.43422	3.07359	-0.12139
H	-2.76231	3.79337	-1.61417
O	-1.55957	2.51313	1.64955

16. References

(1) Stájer, G.; Szabó, A. E.; Túrós, G.; Sohár, P.; Sillanpää, R. Indoloquinolines, Indolobenzoxazines, and Quinazolophthalazines Prepared from Norbornane/Eneamino Acids and Hydrazides. *Eur. J. Org. Chem.* **2005**, *2005* (19), 4154–4161.

(2) Terán, C.; Raviña, E.; Santana, L.; García-Domínguez, N.; García-Mera, G.; Fontenla, J. A.; Orallo, F.; Calleja, J. M. Pyridazine Derivatives, VII. Synthesis and Hypotensive Activity of 3-Hydrazinocycloalkyl[1,2-c]pyridazines and Their Derivatives. *Arch. Pharm. (Weinheim, Ger.)* **1989**, *322* (6), 331–336.

(3) Wang, S.; Xie, C.; Zhu, Y.; Zi, G.; Zhang, Z.; Hou, G. Enantioselective Synthesis of Chiral Cyclic Hydrazines by Ni-Catalyzed Asymmetric Hydrogenation. *Org. Lett.* **2023**, *25* (20), 3644–3648.

(4) Reddy, R. S.; Saravanan, K.; Kumar, P. An Efficient Approach to γ-Alkylidene-γ-Butyrolactones: Application to the Syntheses of Pyridazinones and Diazocinones. *Tetrahedron* **1998**, *54* (23), 6553–6564.

(5) Lebrêne, A.; Martzel, T.; Gouriou, L.; Sanselme, M.; Levacher, V.; Oudeyer, S.; Afonso, C.; Loutelier-Bourhis, C.; Brière, J.-F. Catalytic Regio- and Stereoselective Synthesis of 1,6-Diazabicyclo[4.3.0]nonane-2,7-diones. *J. Org. Chem.* **2021**, *86* (13), 8600–8609.

(6) Taylor, E. C.; Hinkle, J. S. New Routes to 1,2-Diazetidin-3-ones. *J. Org. Chem.* **1987**, *52* (18), 4107–4110.

(7) Luo, Z.; Yang, J.; Yang, J.; Yao, Z.; He, Z.; Liu, K.; Xu, L.; Shi, Q. Borane-Catalyzed Selective Transformation of Levulinic Acid and Hydrazines into Hexahydropyridazines and Tetrahydropyridazin-3(2H)-ones Using Hydrosilane. *Adv. Synth. Catal.* **2024**, *366* (23), 4900–4906.

(8) McCann, S. D.; Stahl, S. S. Copper-Catalyzed Aerobic Oxidations of Organic Molecules: Pathways for Two-Electron Oxidation with a Four-Electron Oxidant and a One-Electron Redox-Active Catalyst. *Acc. Chem. Res.* **2015**, *48* (6), 1756–1766.

(9) Grošelj, U.; Požgan, F.; Štefane, B.; Svete, J. Copper-Catalyzed Azomethine Imine–Alkyne Cycloadditions (CuAIAC). *Synthesis* **2018**, *50* (23), 4501–4524.

(10) Nájera, C.; Sansano, J. M.; Yus, M. 1,3-Dipolar Cycloadditions of Azomethine Imines. *Org. Biomol. Chem.* **2015**, *13* (32), 8596–8636.

(11) AMS2025.103 r250525, Theoretical Chemistry, Vrije University, Amsterdam, The Netherlands 2025, https://www.scm.com.

(12) (a) Y. Zhao, D. G. Truhlar, *Theor. Chem. Acc.* **2008**, *120*, 215–241; (b) P. C. Hariharan, J. A. Pople, *Theor. Chim. Acta* **1973**, *213*, 213–222. (c) Gaussian 16, Revision C.01, M. J. Frisch, G. W. Trucks, H. B. Schlegel, G. E. Scuseria, M. A. Robb, J. R. Cheeseman, G. Scalmani, V. Barone, B. Mennucci, G. A. Petersson, H. Nakatsuji, M. Caricato, X. Li, H. P. Hratchian, A. F. Izmaylov, J. Bloino, G. Zheng, J. L. Sonnenberg, M. Hada, M. Ehara, K. Toyota, R. Fukuda, J. Hasegawa, M. Ishida, T. Nakajima, Y. Honda, O. Kitao, H. Nakai, T. Vreven, J. A. Montgomery, Jr., J. E. Peralta, F. Ogliaro, M. Bearpark, J. J. Heyd, E. Brothers, K. N. Kudin, V. N. Staroverov, R. Kobayashi, J. Normand, K. Raghavachari, A. Rendell, J. C. Burant, S. S. Iyengar, J. Tomasi, M. Cossi, N. Rega, J. M. Millam, M. Klene, J. E. Knox, J. B. Cross, V. Bakken, C. Adamo, J. Jaramillo, R. Gomperts, R. E. Stratmann, O. Yazyev, A. J. Austin, R. Cammi, C. Pomelli, J. W. Ochterski, R. L. Martin, K. Morokuma, V. G. Zakrzewski,

G. A. Voth, P. Salvador, J. J. Dannenberg, S. Dapprich, A. D. Daniels, O. Farkas, J. B. Foresman, J. V. Ortiz, J. Cioslowski, D. J. Fox, Gaussian Inc., Wallingford CT, **2009**.

(13) (a) A. Klamt, *J. Phys. Chem.* **1995**, *99*, 2224 – 2235. (b) A. Klamt, V. Jonas, *J. Phys. Chem.* **1996**, *105*, 9972 – 9981.

(14) S. Hirata, M. Head-Gordon, *Chem. Phys. Lett.* **1999**, *314*, 291 – 299.

17. NMR spectra

17.1 Compounds **PYR**

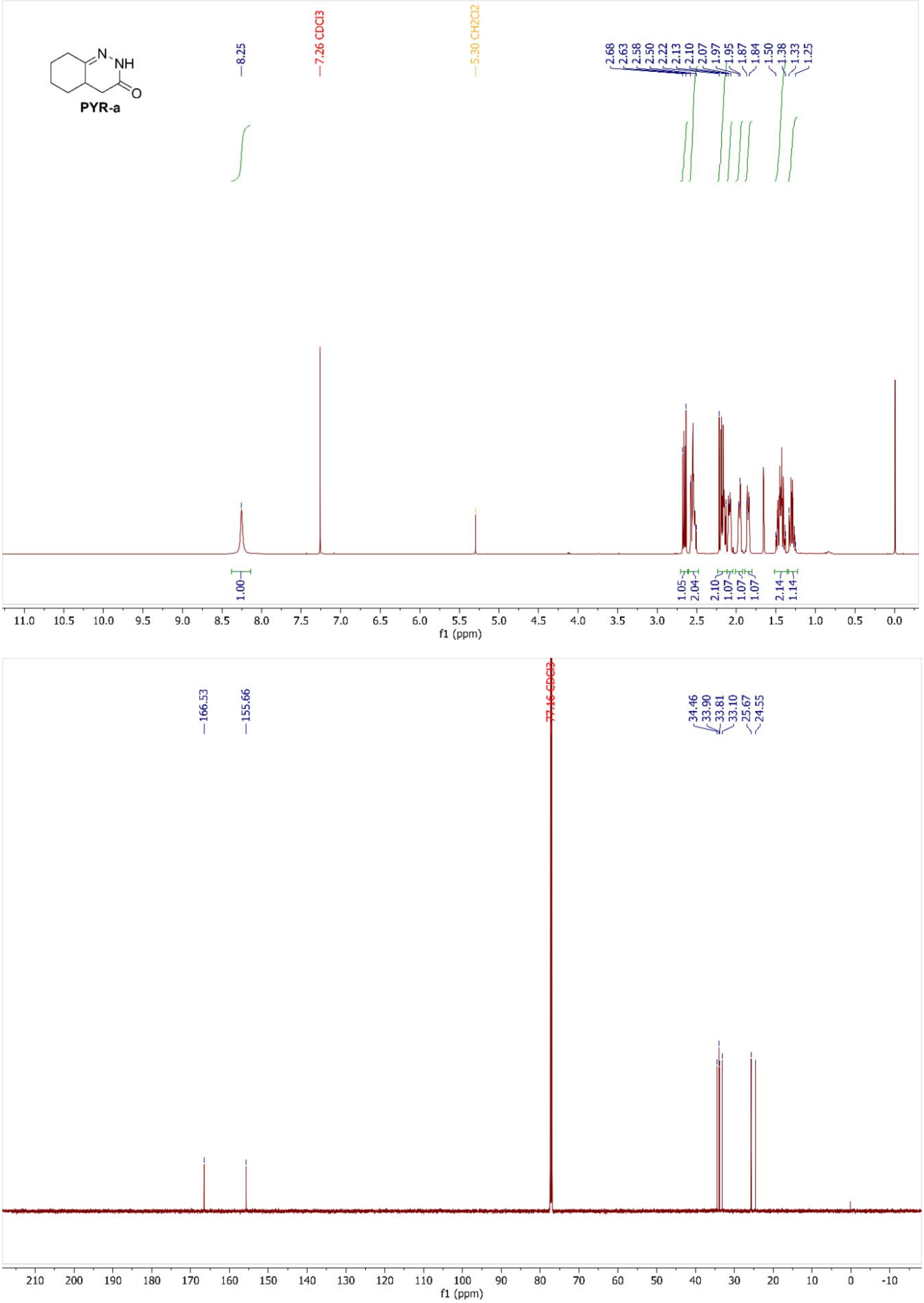

Figure S12. ^{1}H and $^{13}C\{1H\}$ NMR (500 MHz, $CDCl_3$) of compound **PYR-a**.

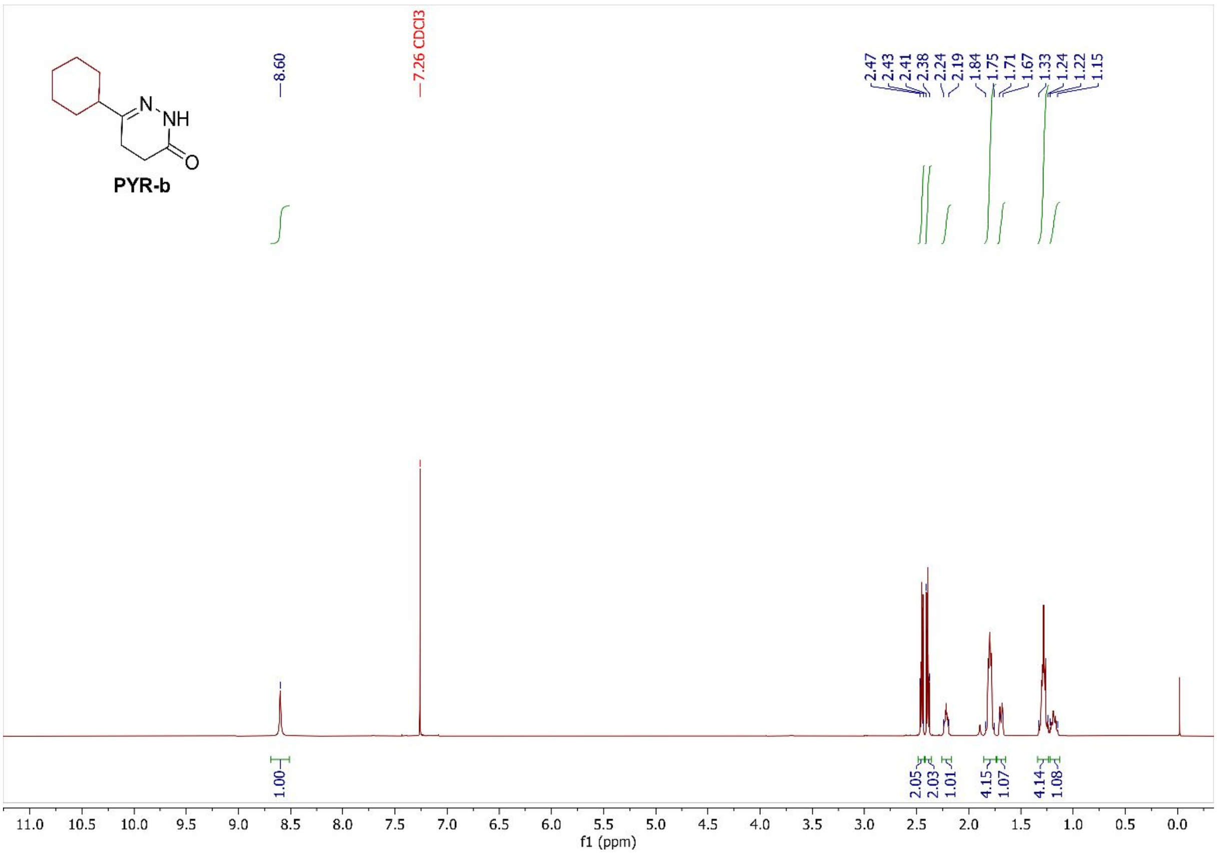

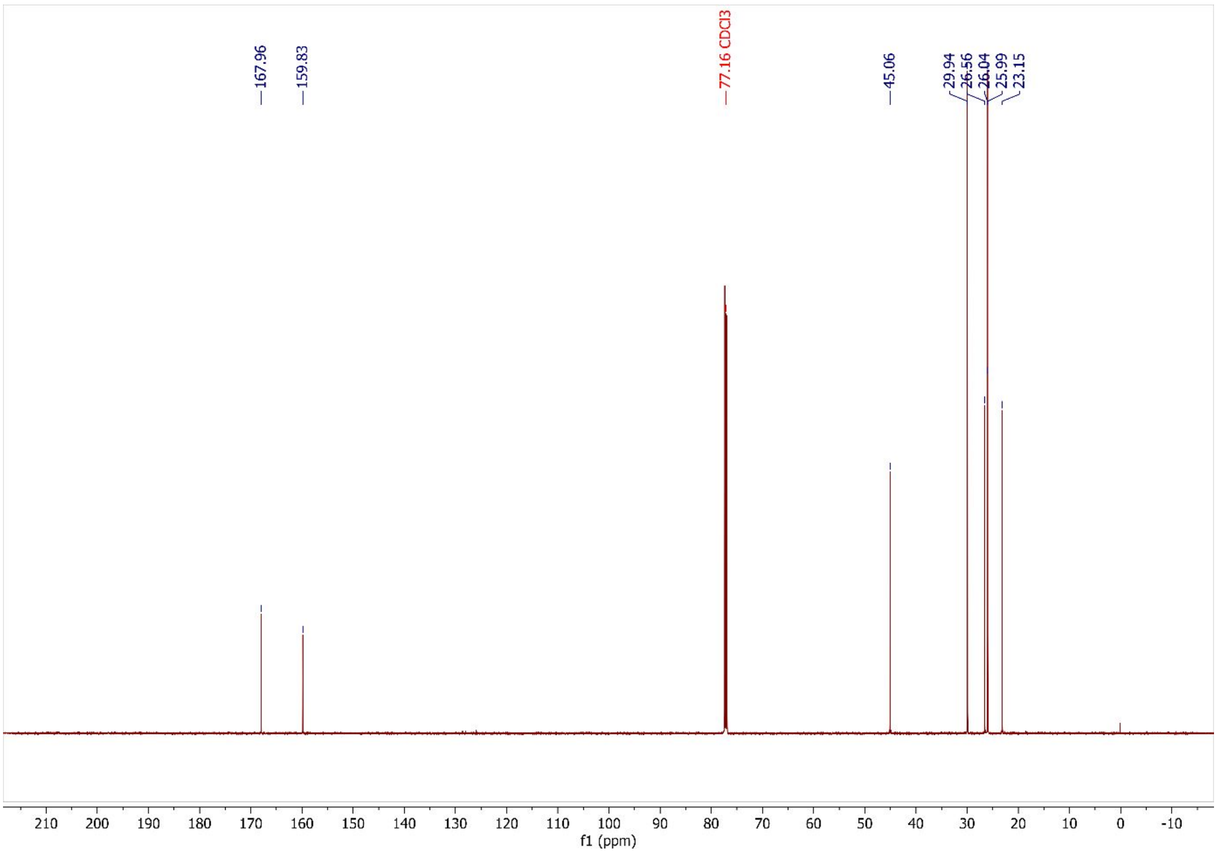

Figure S13. ^{1}H and ^{13}C{1H} NMR (600 MHz, $CDCl_3$) of compound **PYR-b**.

17.2 Tetrahydropyridazin-3(2*H*)-ones **1**

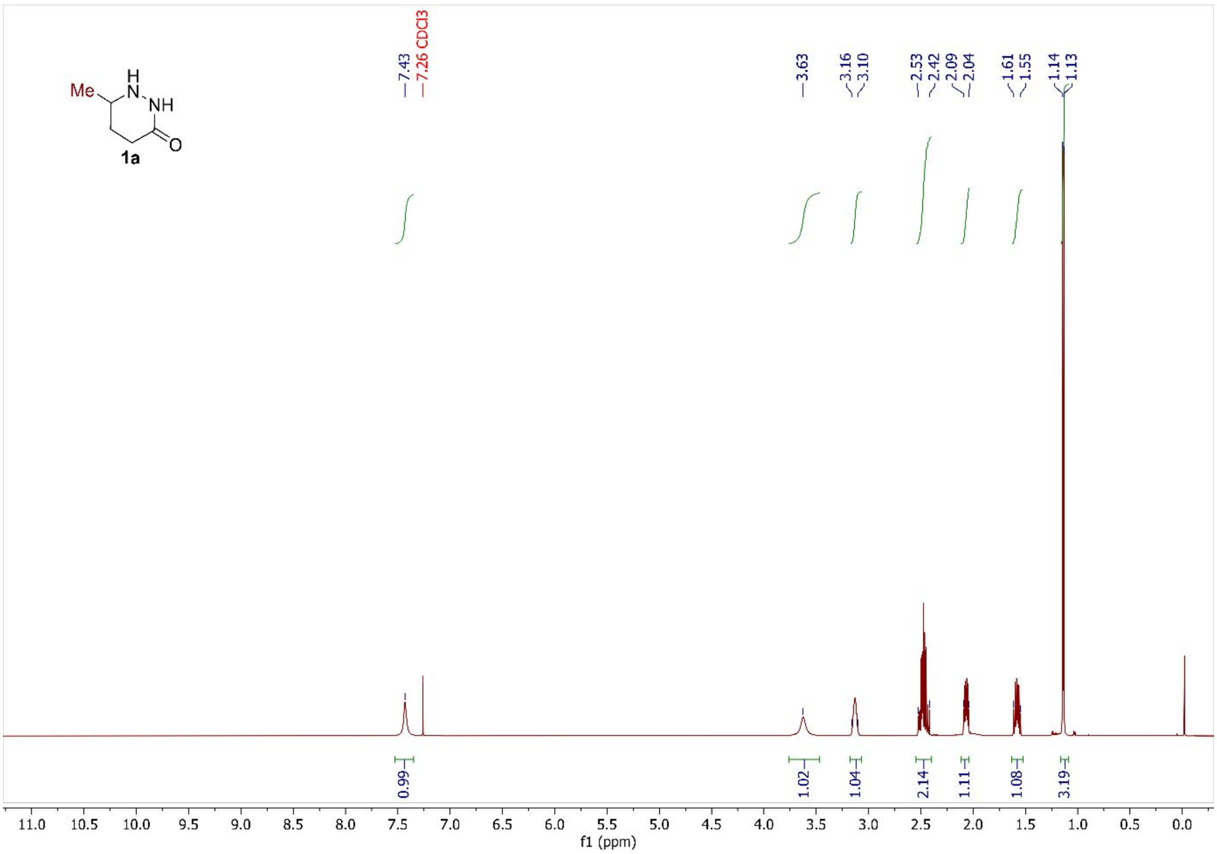

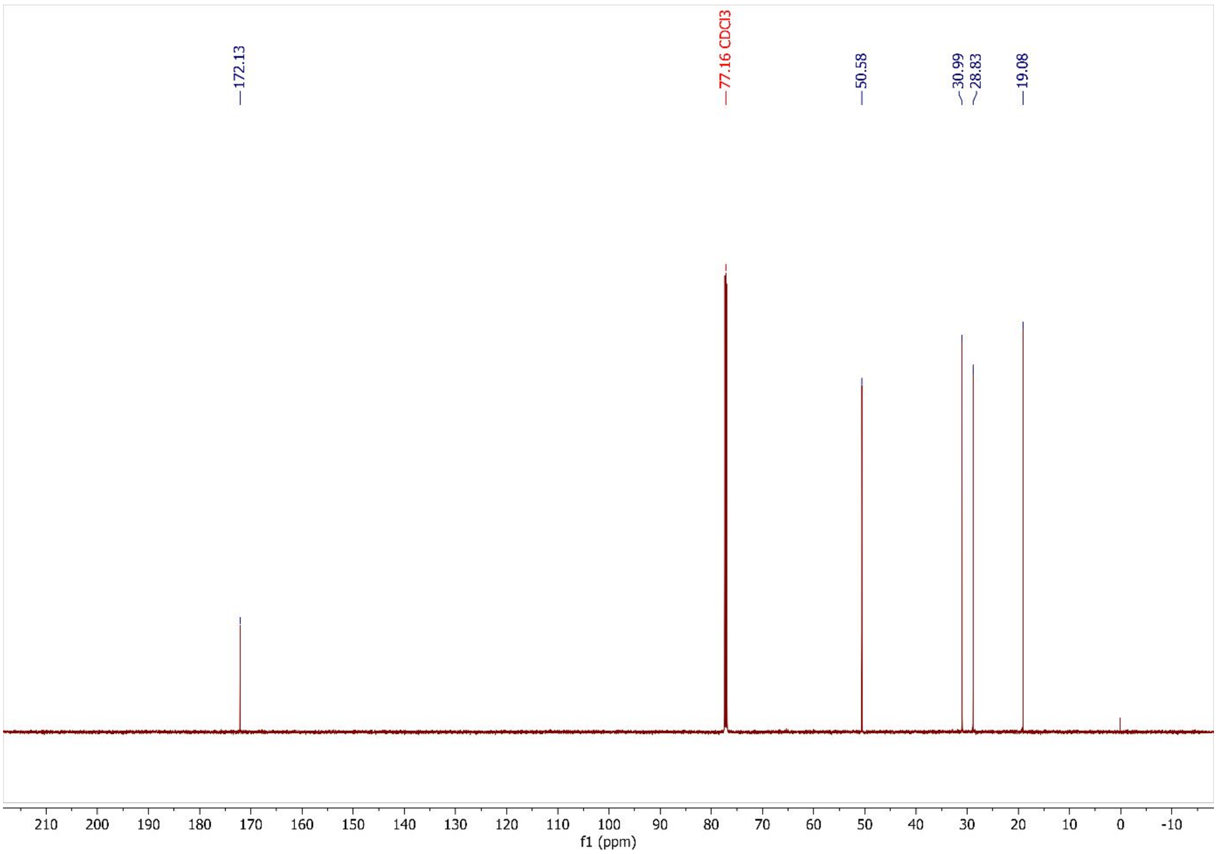

Figure S14. ^{1}H and $^{13}C\{1H\}$ NMR (500 MHz, $CDCl_3$) of compound **1a**.

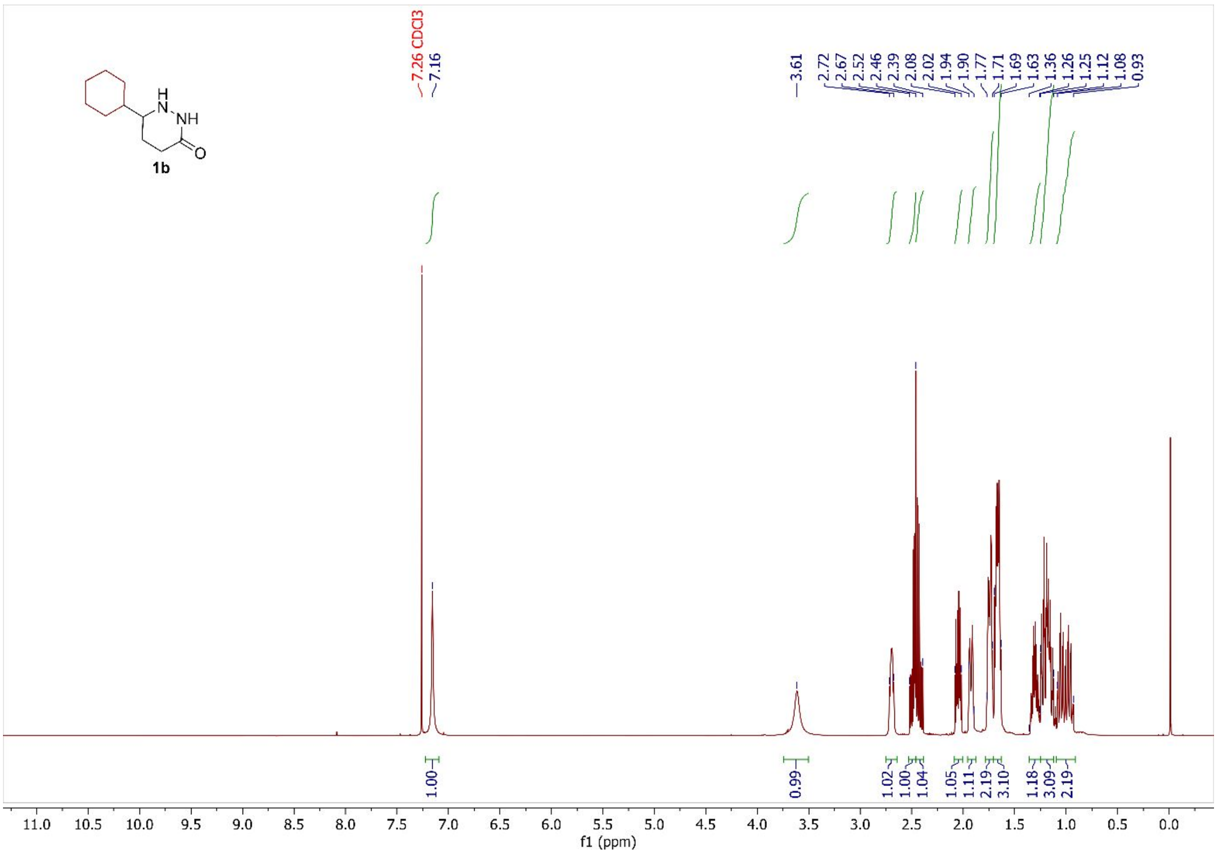

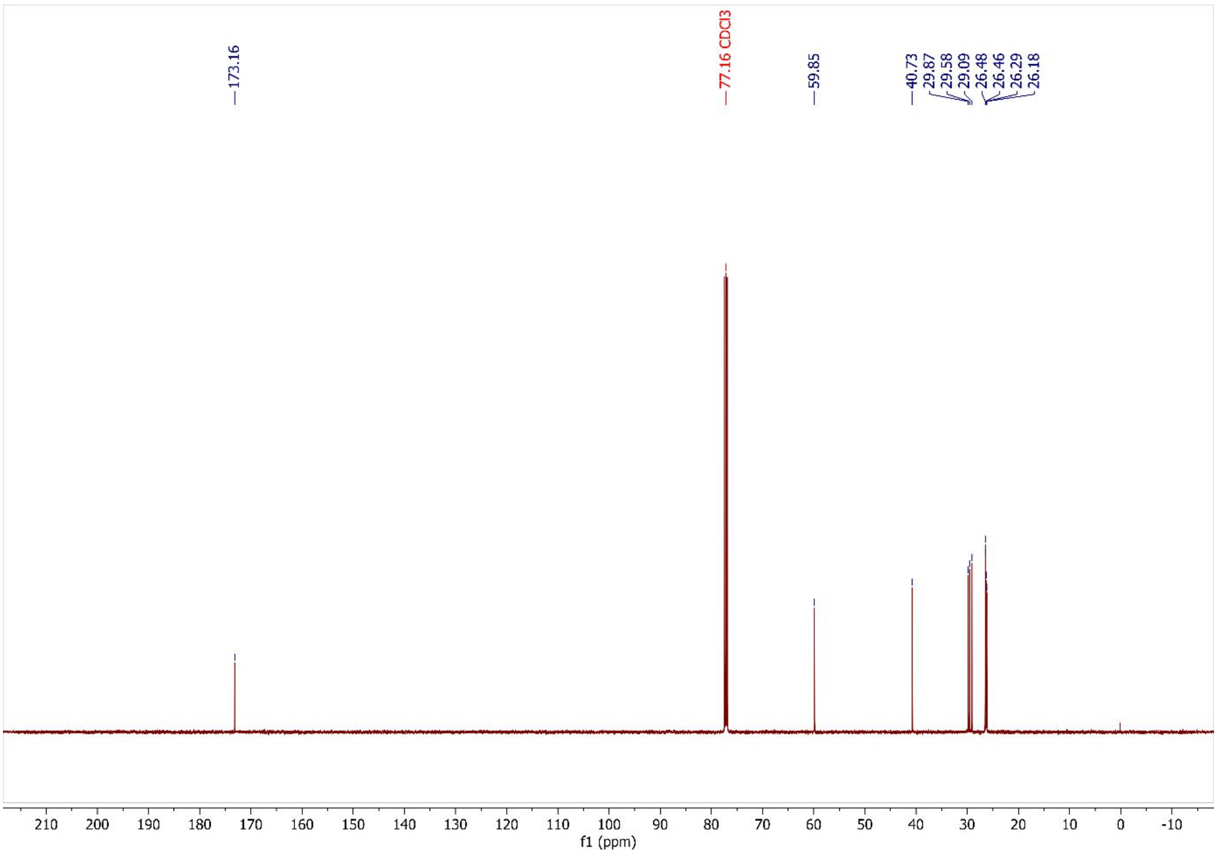

Figure S15. ^{1}H and $^{13}C\{1H\}$ NMR (500 MHz, $CDCl_3$) of compound **1b**.

^{1}H NMR spectrum of a mixture of **1b** and **1c**. Only signals attributed to **1c** are integrated to aid in peak assignment. Mixture **1b**/**1c** was used directly for the synthesis of **2i** and **5b** without further purification or full characterization.

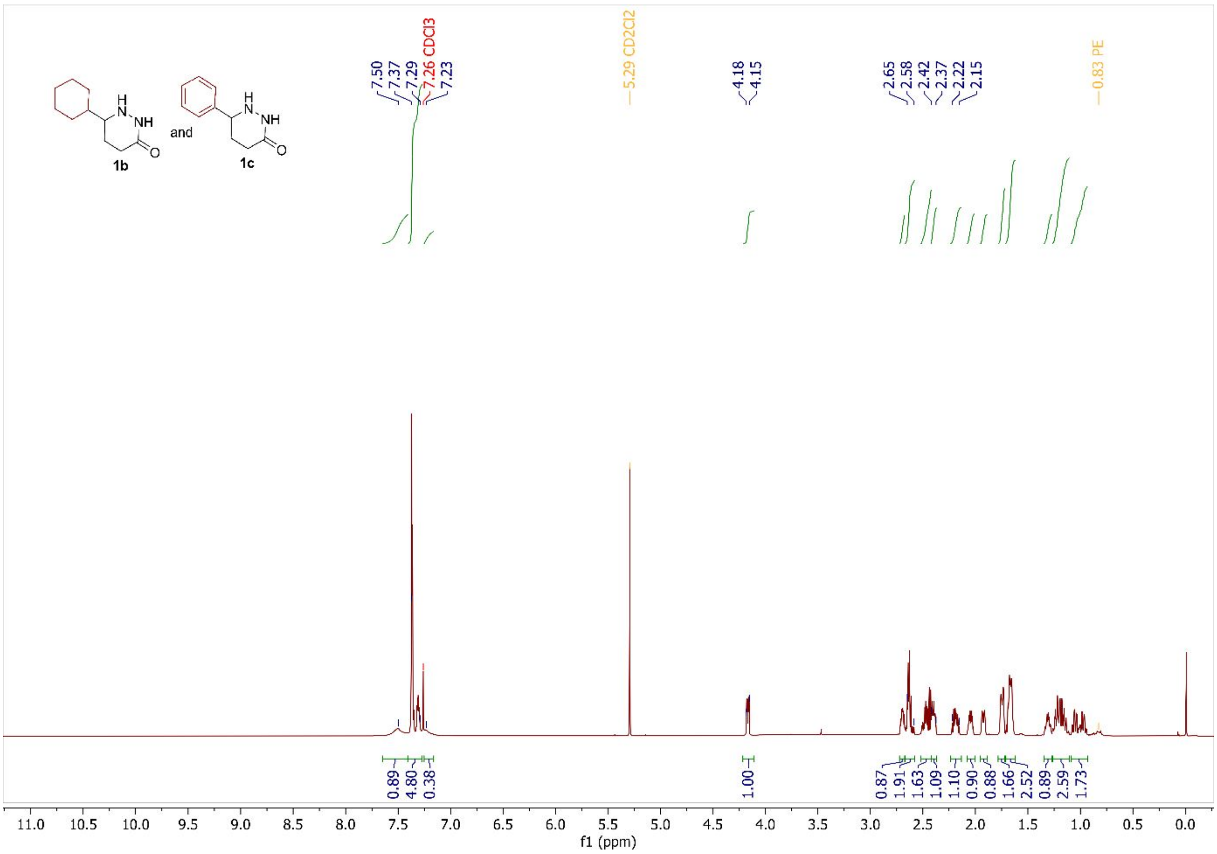

Figure S16. ^{1}H and $^{13}C\{1H\}$ NMR (600 MHz, $CDCl_3$) of mixture **1b**/**1c**.

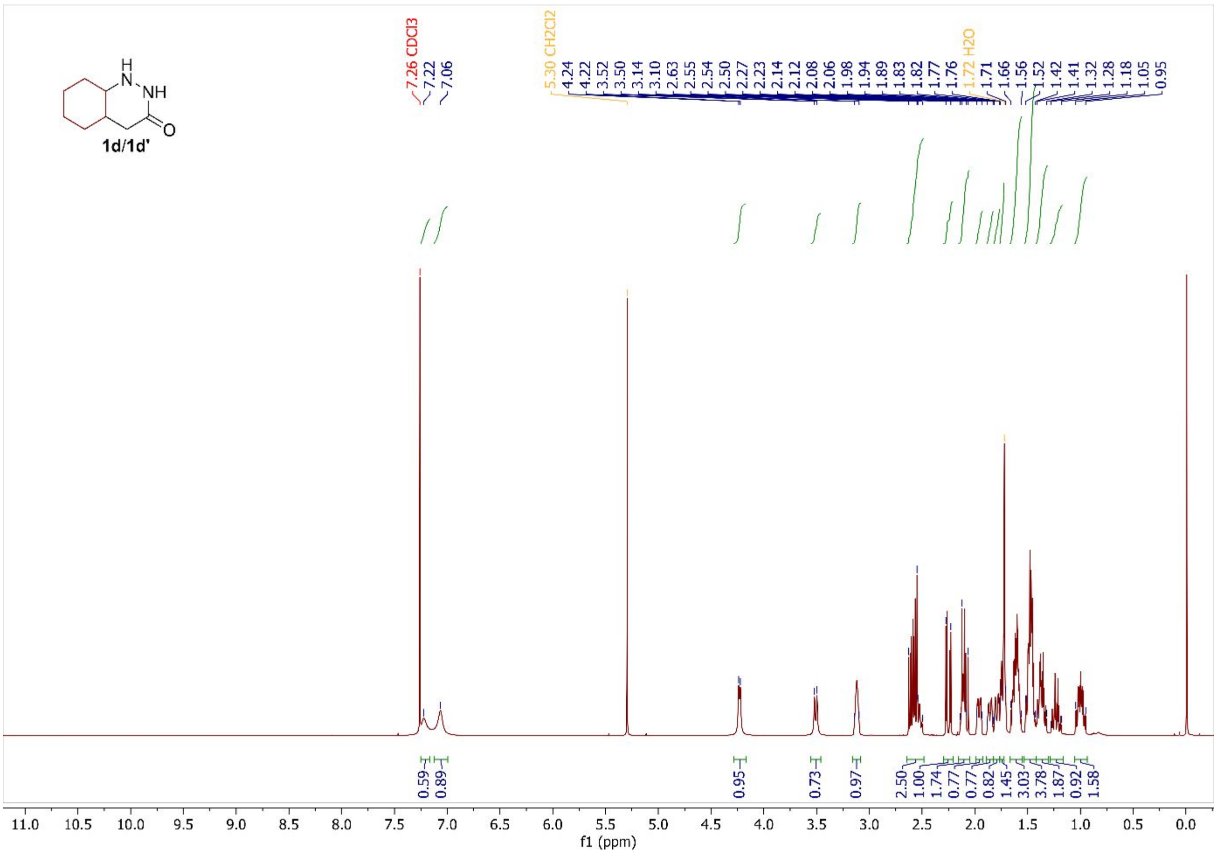

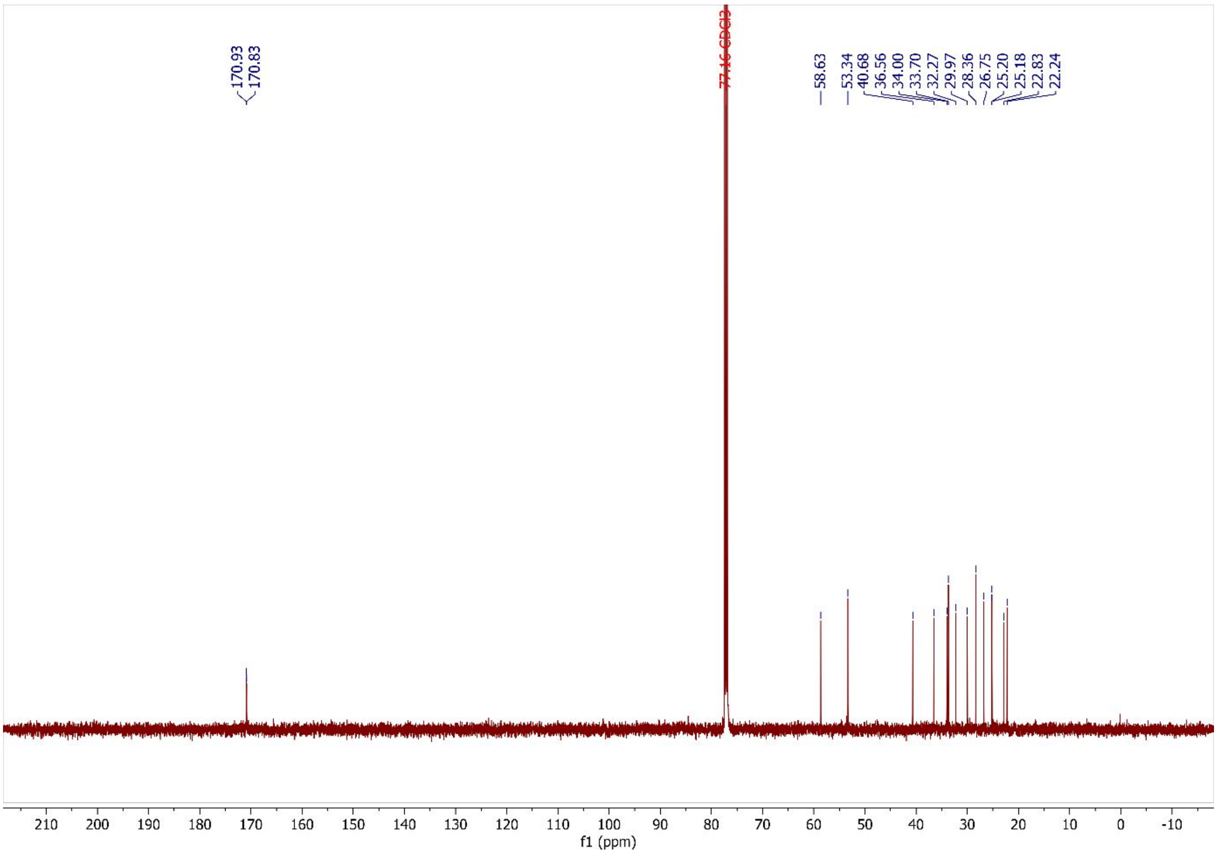

Figure S17. ^{1}H and ^{13}C{1H} NMR (500 MHz, $CDCl_3$) of compounds **1d**/**1d'**.

17.3 Pyrazolo[1,2-*a*]pyridazinones **2**

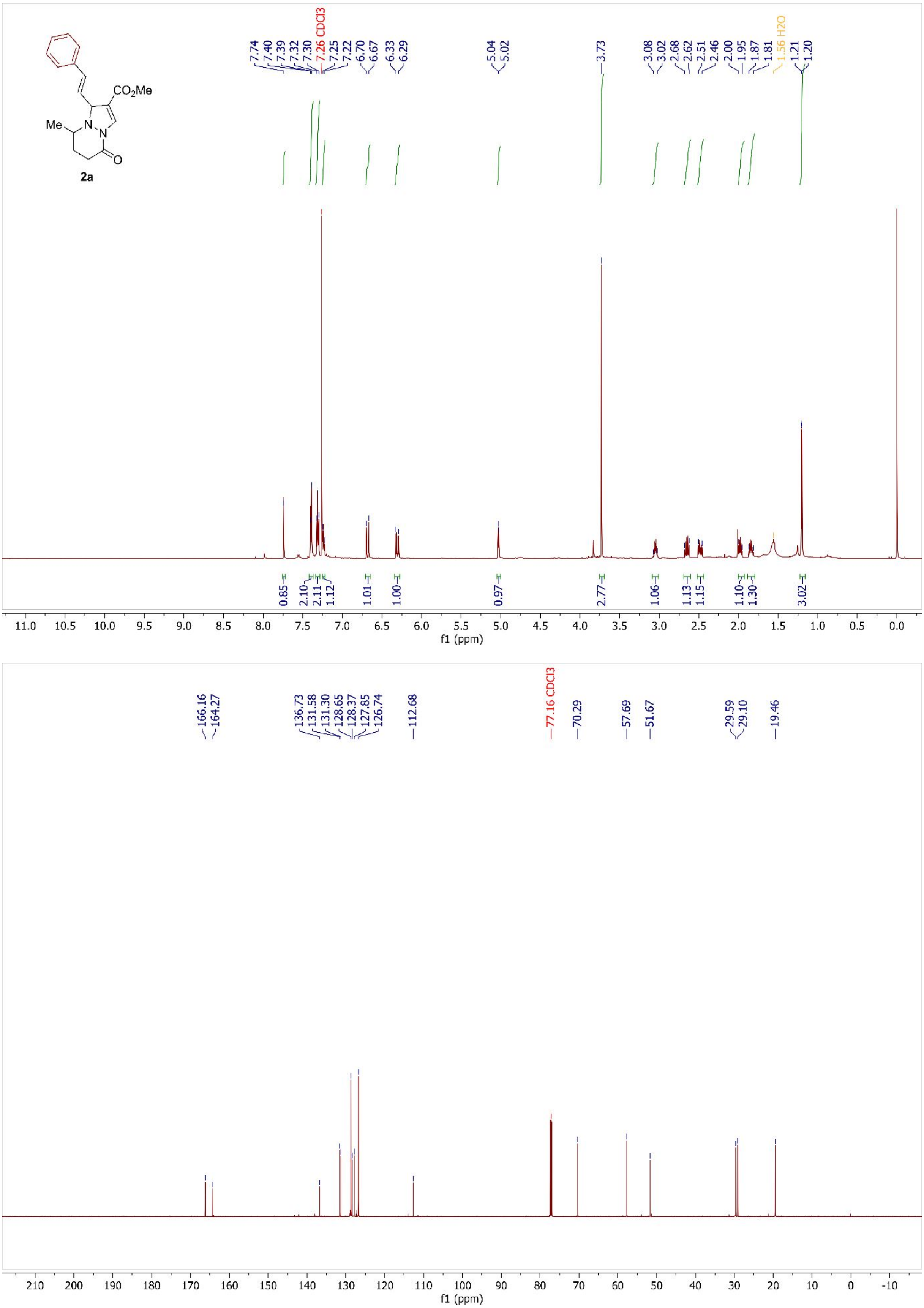

Figure S18. ^{1}H and ^{13}C{1H} NMR (600 MHz, $CDCl_3$) of compound **2a**.

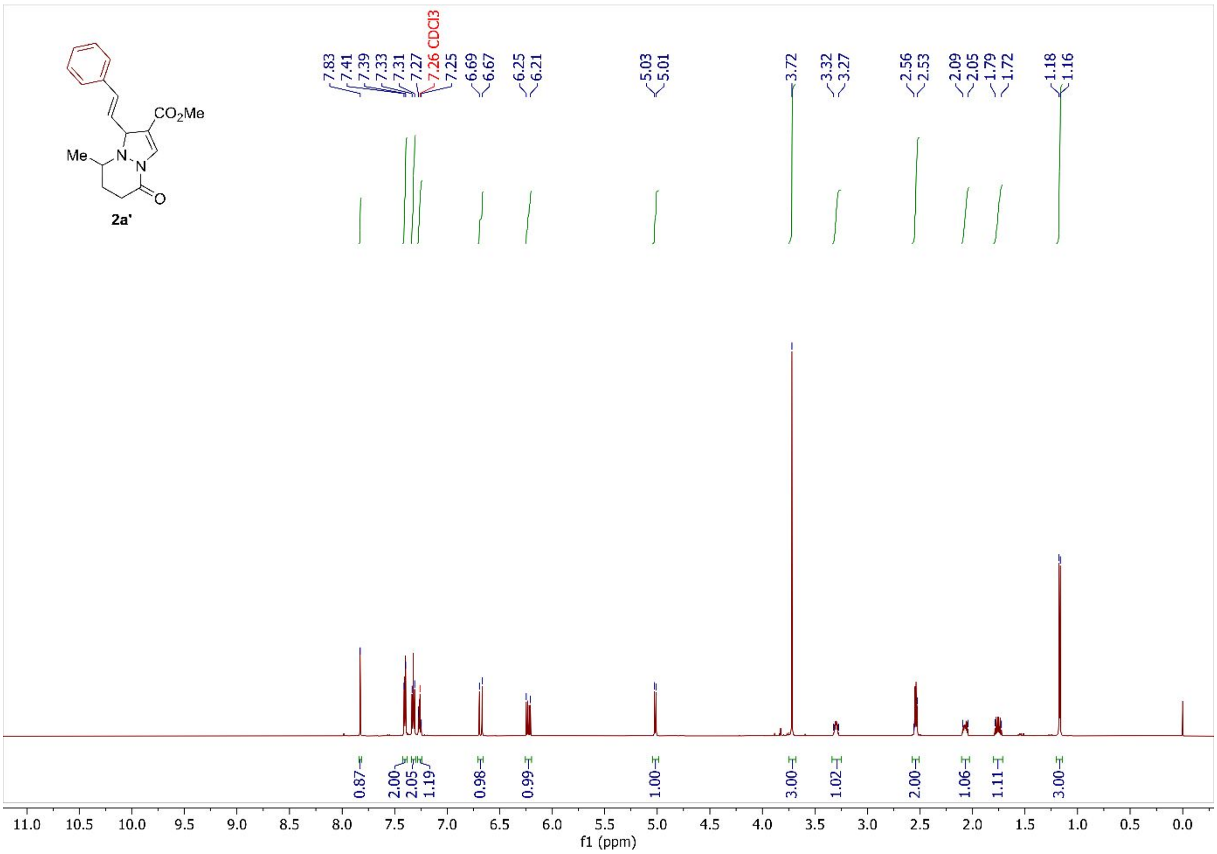

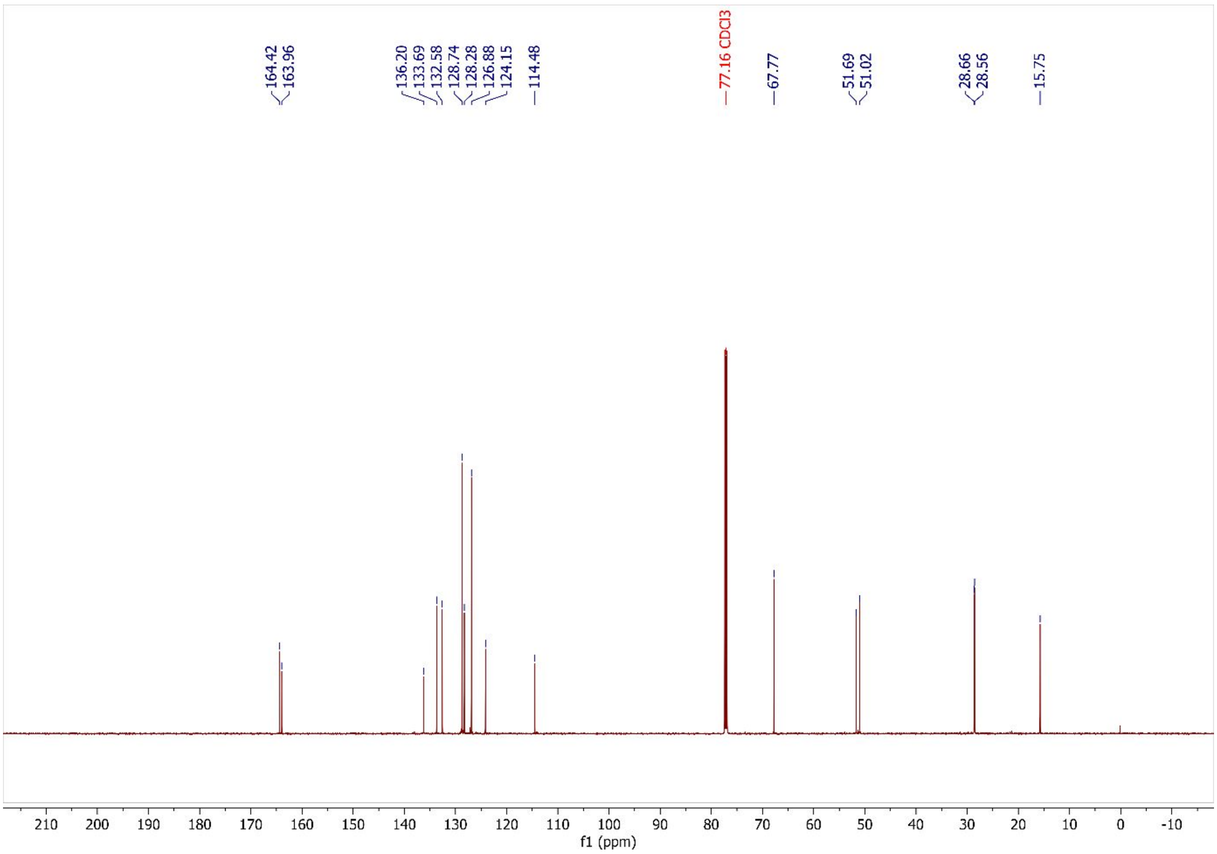

Figure S19. ^{1}H and $^{13}C\{1H\}$ NMR (600 MHz, $CDCl_3$) of compound **2a’**.

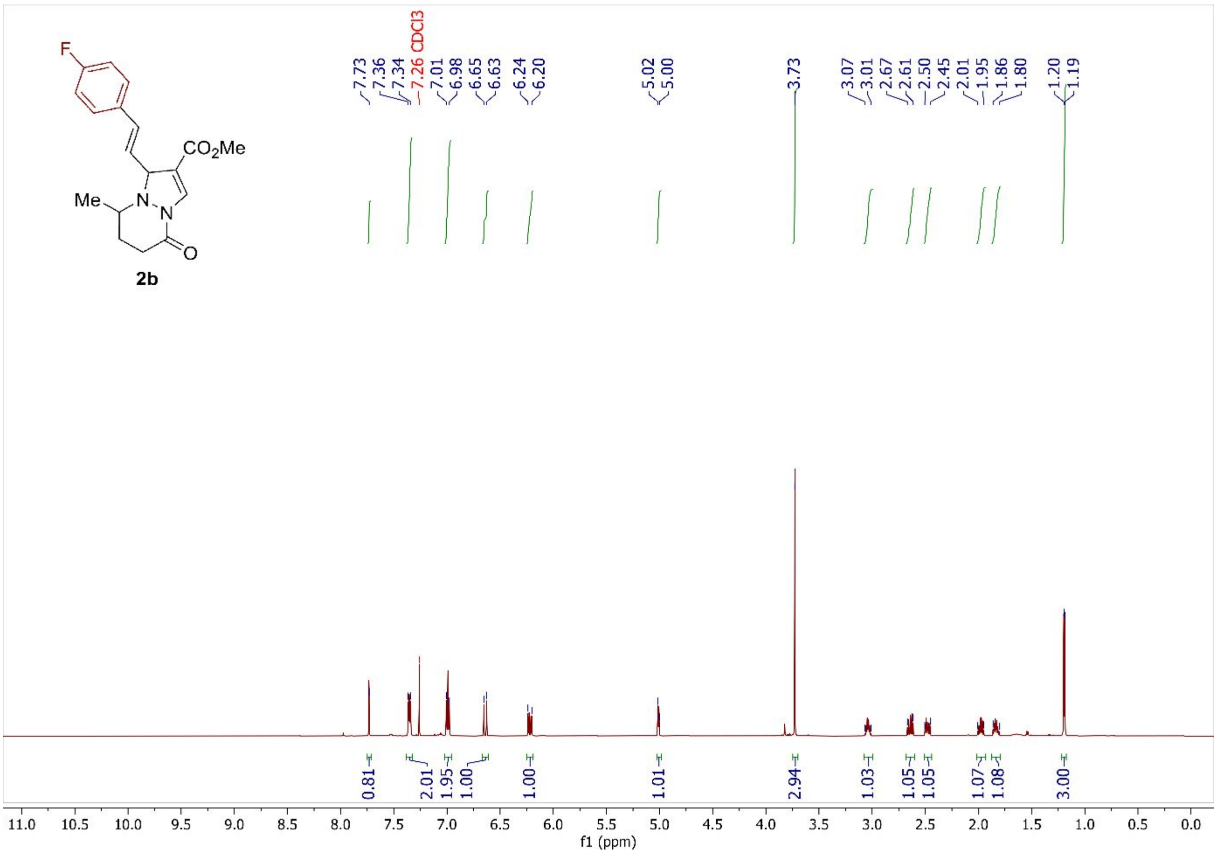

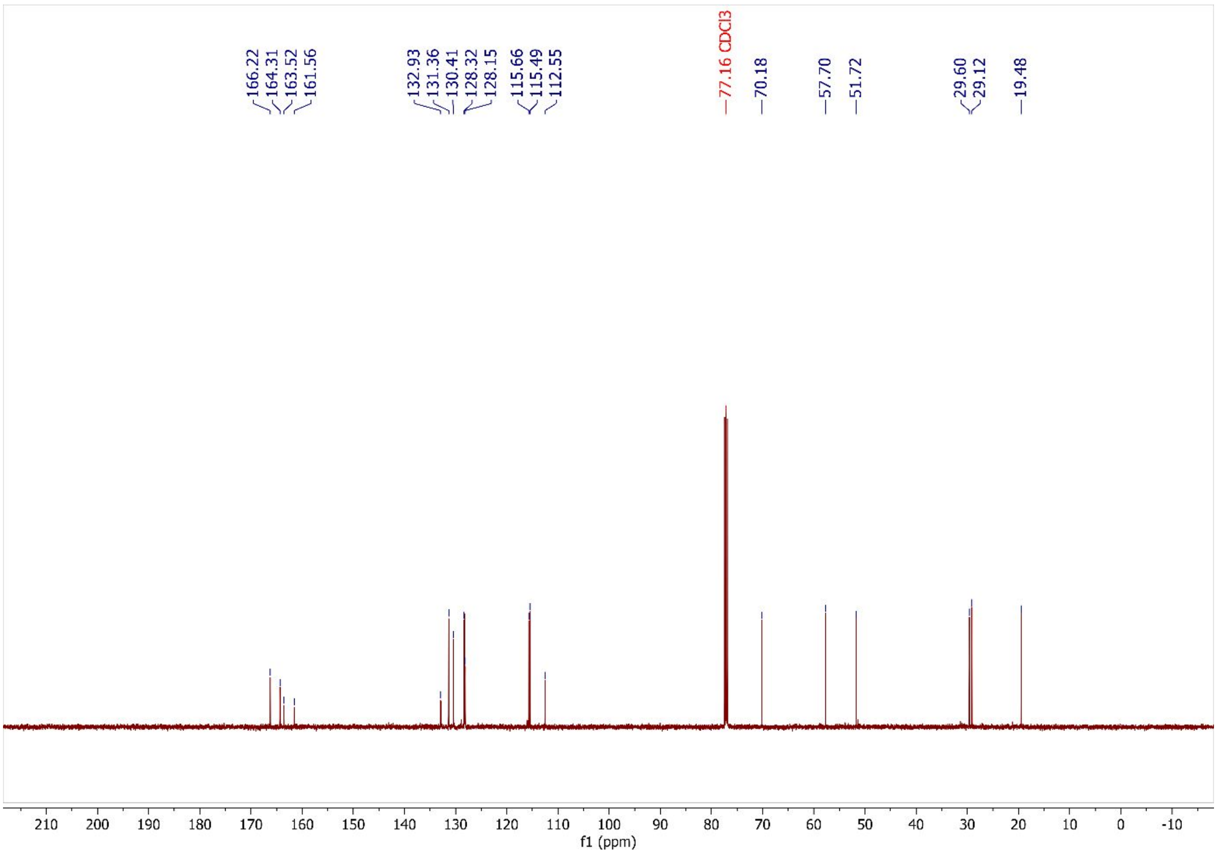

Figure S20. ^{1}H and $^{13}C\{1H\}$ NMR (600 MHz, $CDCl_3$) of compound **2b**.

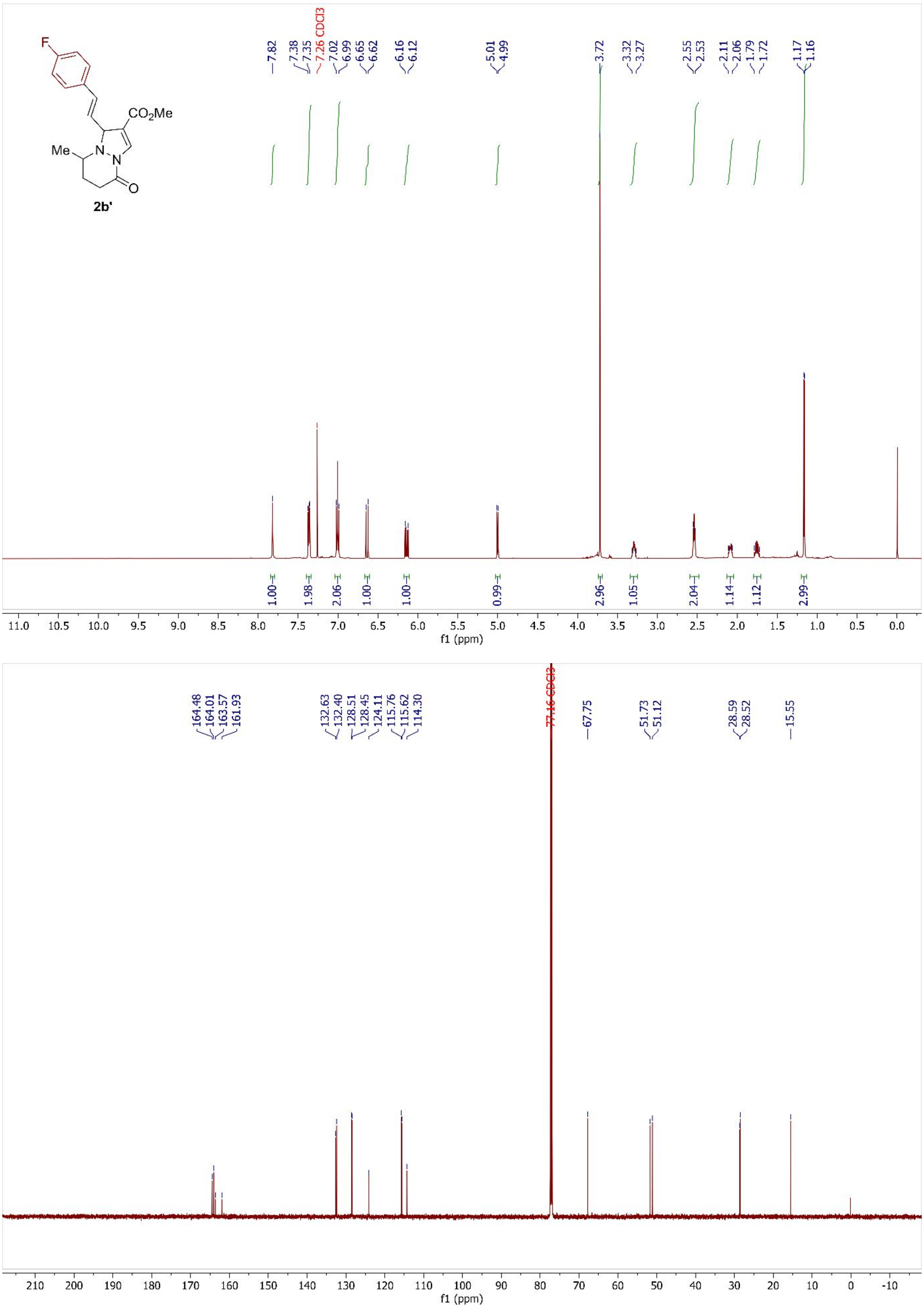

Figure S21. ^{1}H and ^{13}C{1H} NMR (600 MHz, $CDCl_3$) of compound **2b’**.

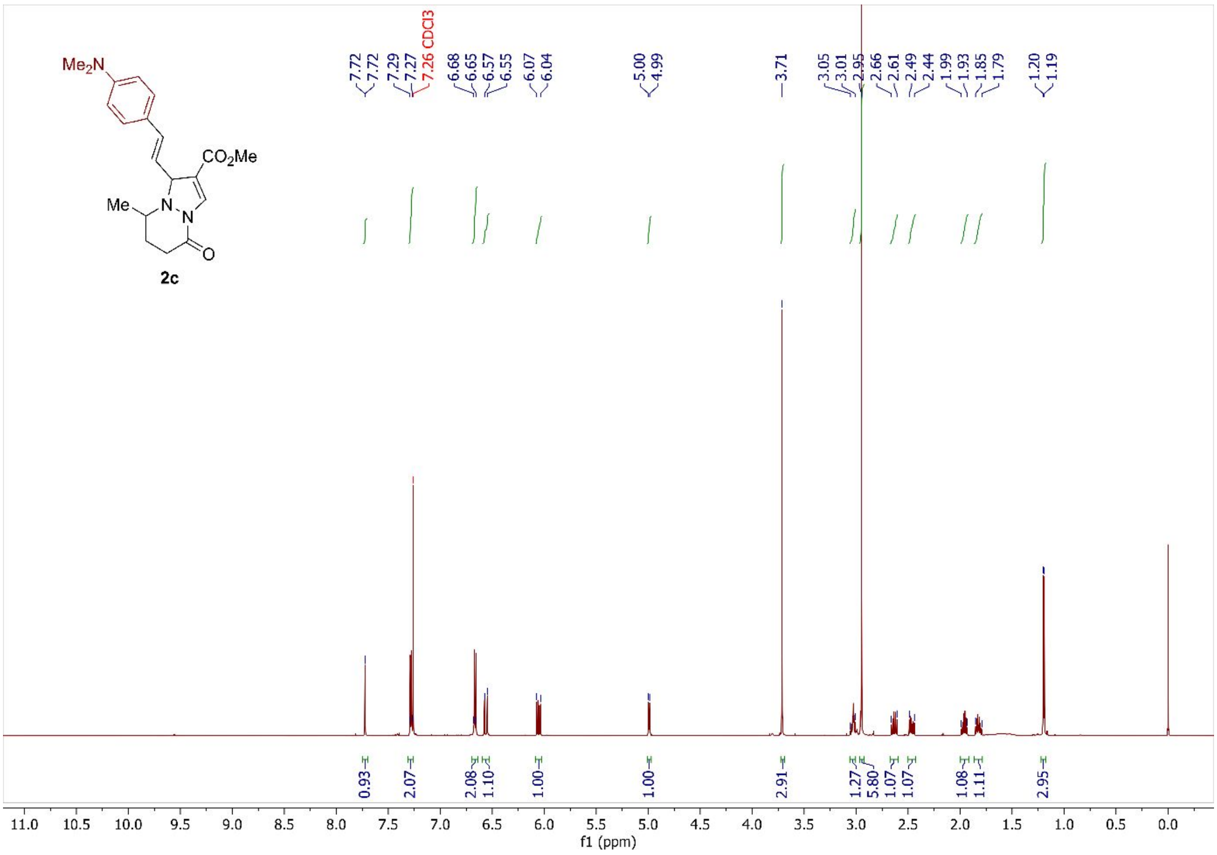

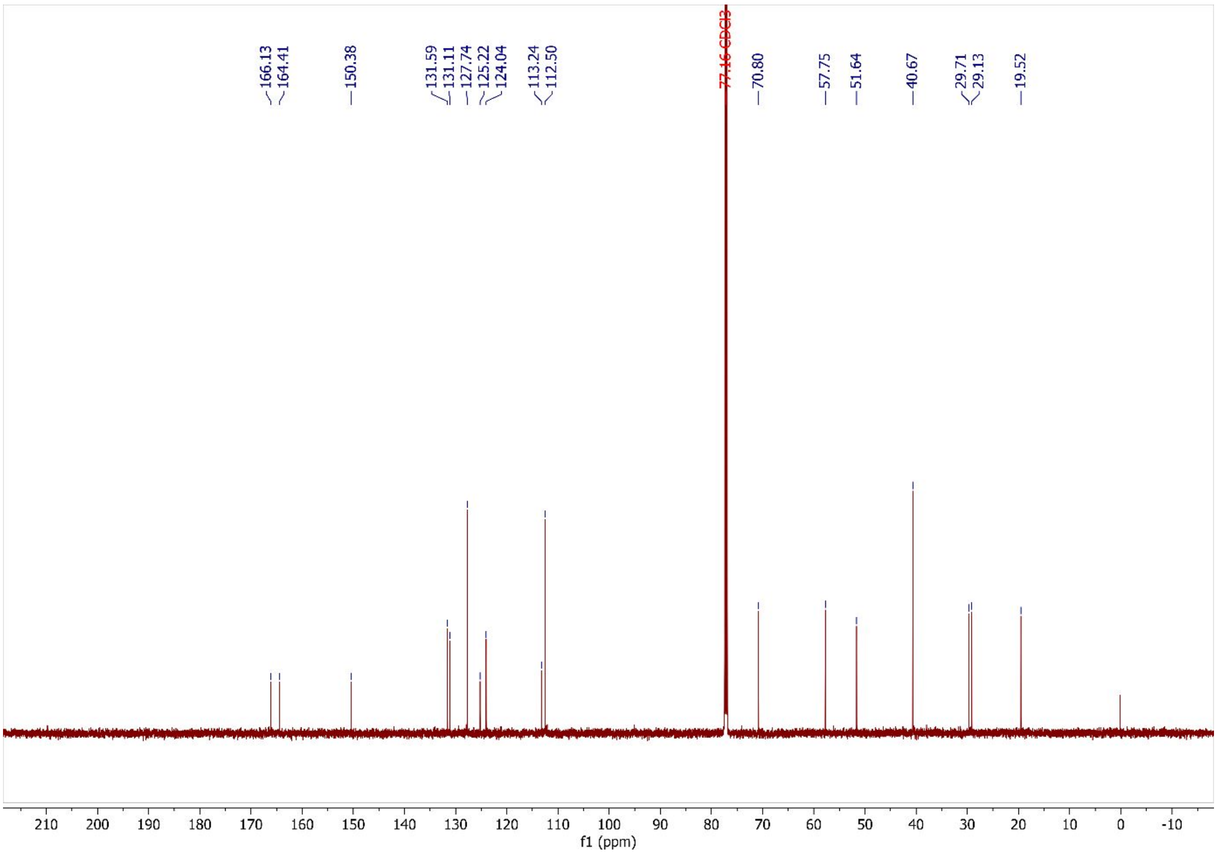

Figure S22. ^{1}H and ^{13}C{1H} NMR (600 MHz, $CDCl_3$) of compound **2c**.

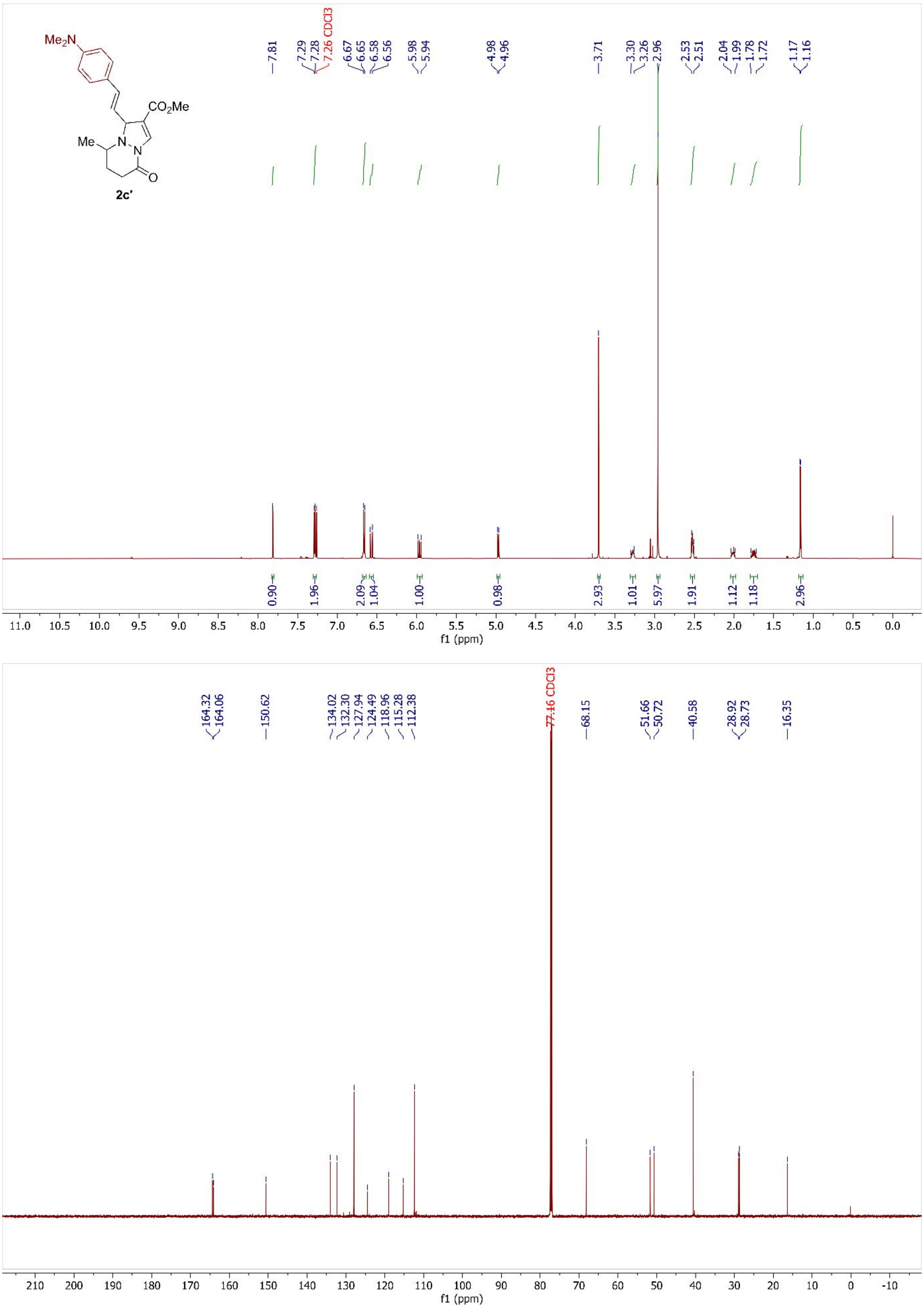

Figure S23. ^1H and $^{13}C\{1H\}$ NMR (600 MHz, $CDCl_3$) of compound **2c'**.

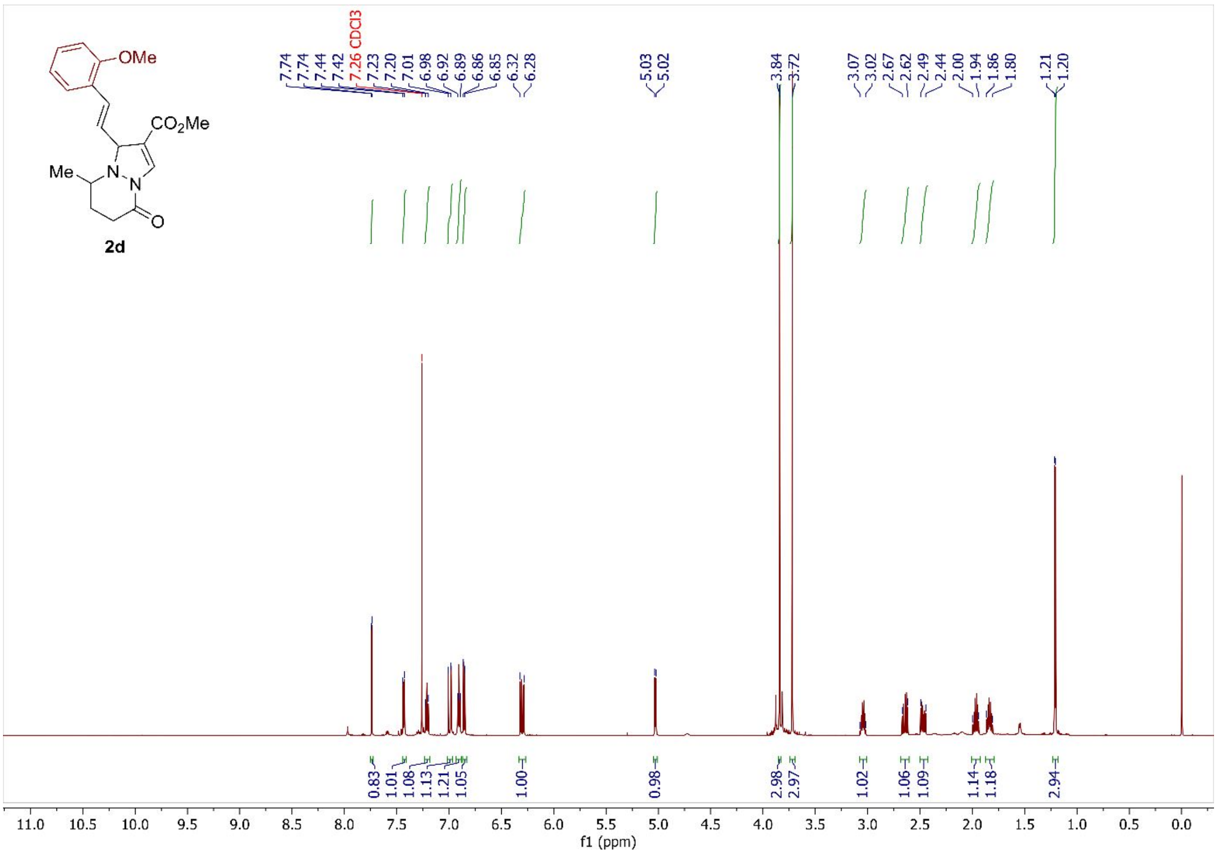

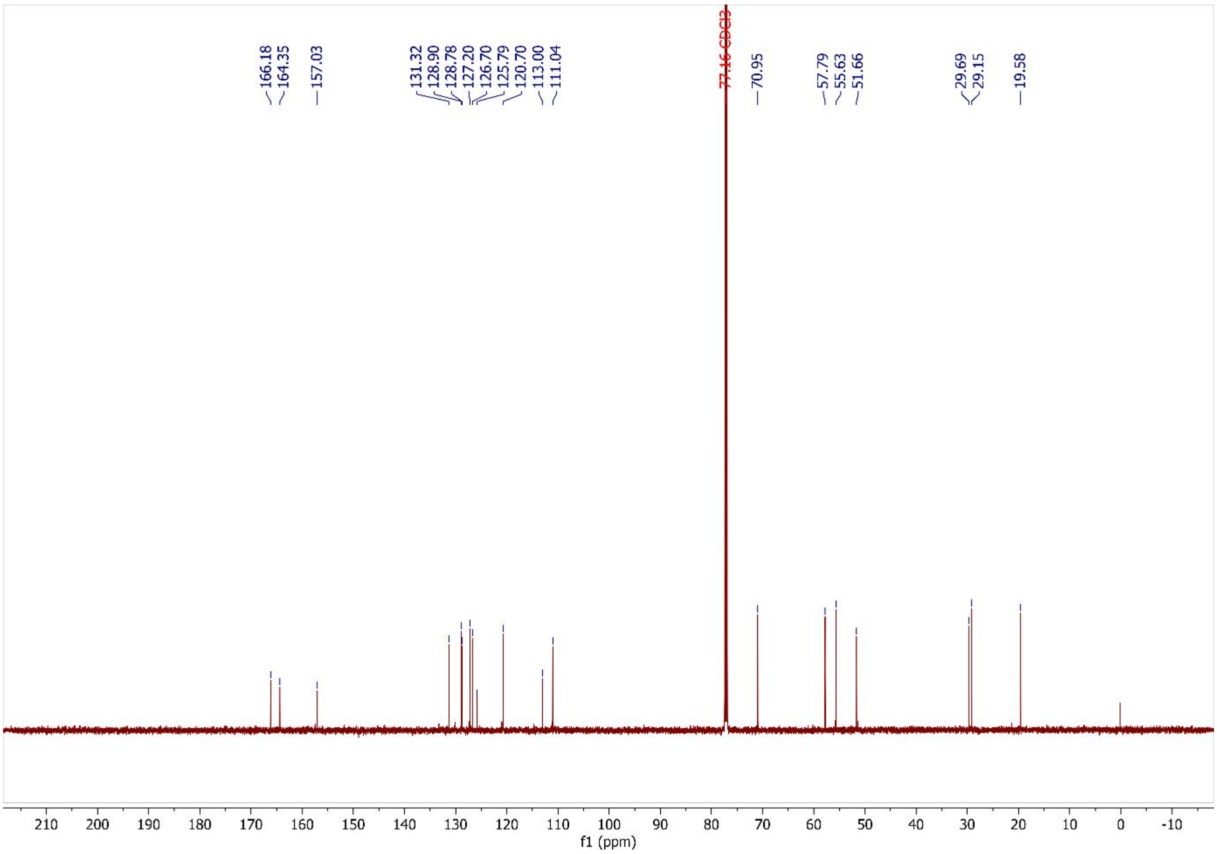

Figure S24. ^{1}H and $^{13}C\{1H\}$ NMR (600 MHz, $CDCl_3$) of compound **2d**.

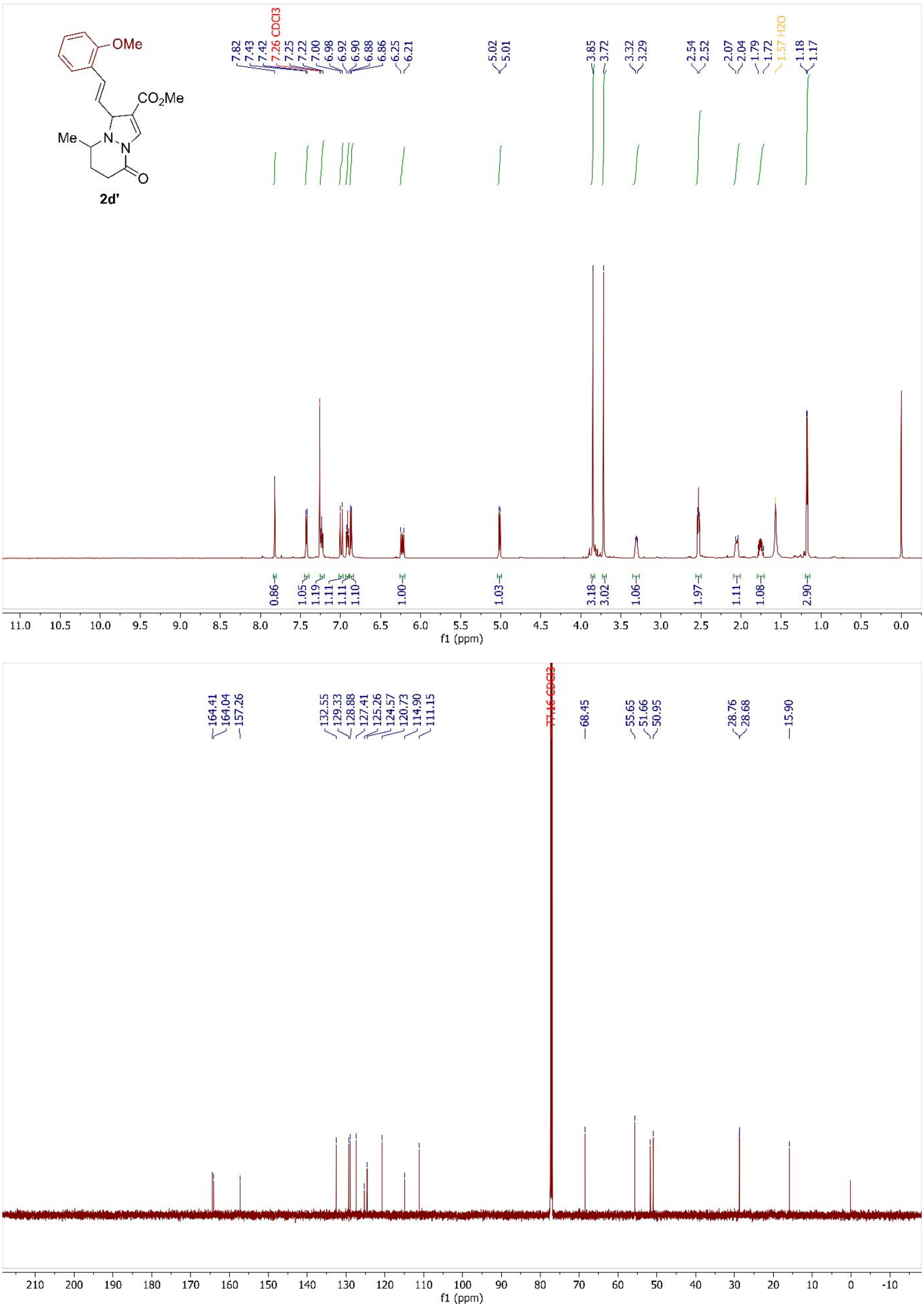

Figure S25. ^{1}H and $^{13}C\{1H\}$ NMR (600 MHz, $CDCl_3$) of compound **2d'**.

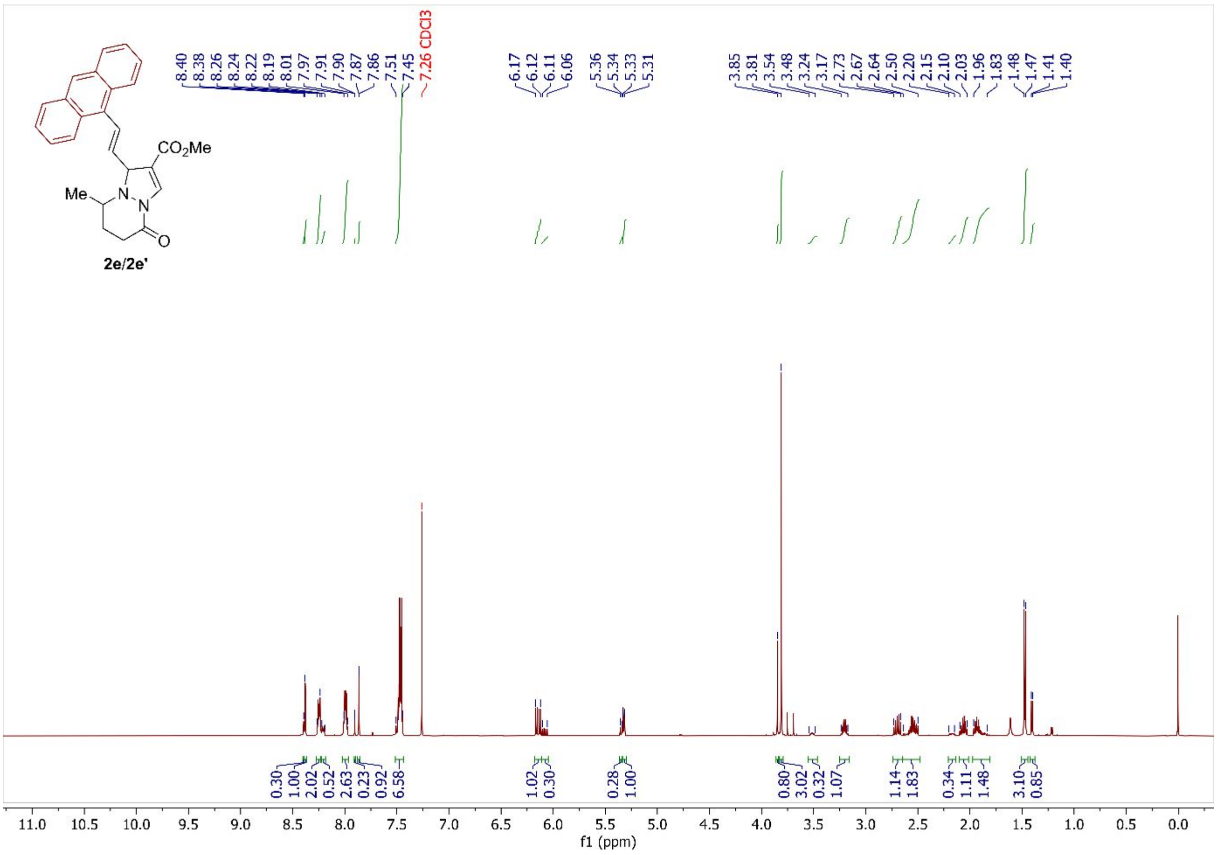

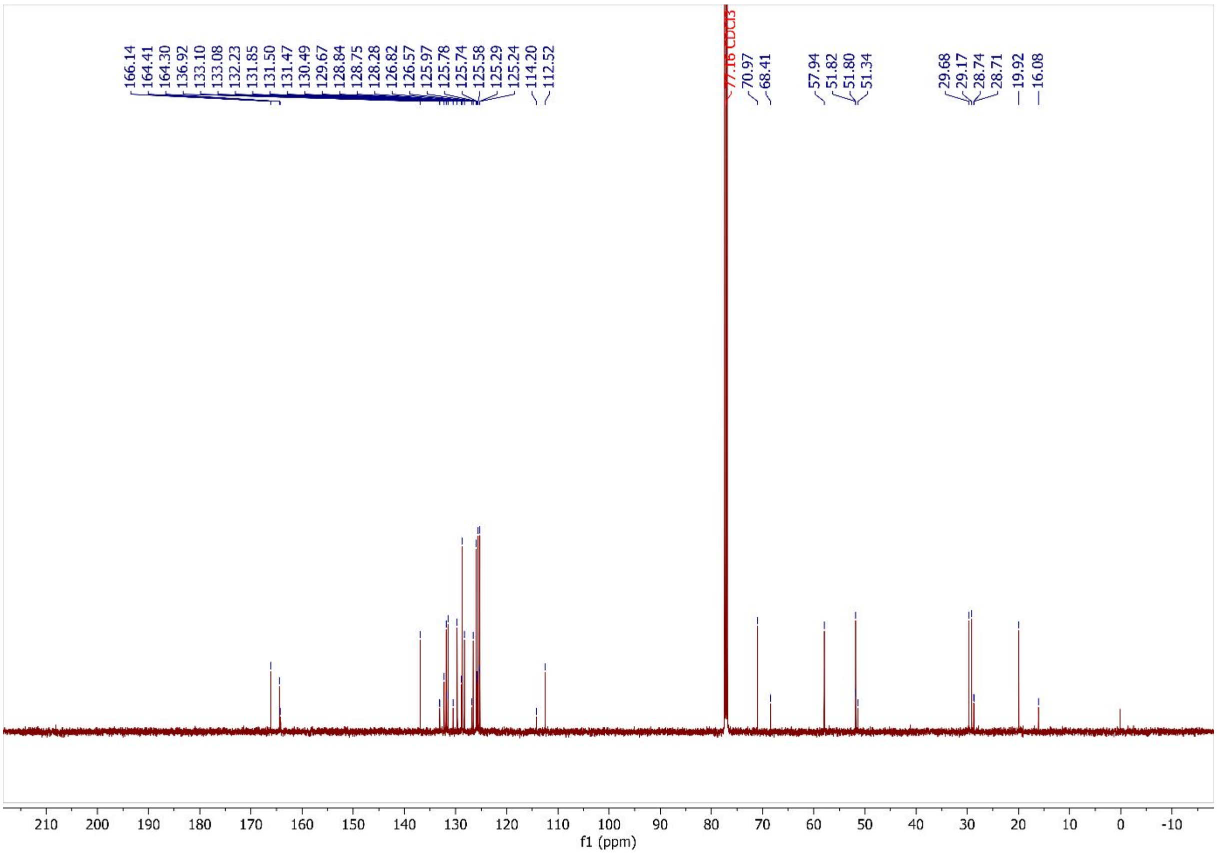

Figure S26. ^{1}H and $^{13}C\{1H\}$ NMR (600 MHz, $CDCl_3$) of compounds **2e**/**2e'**.

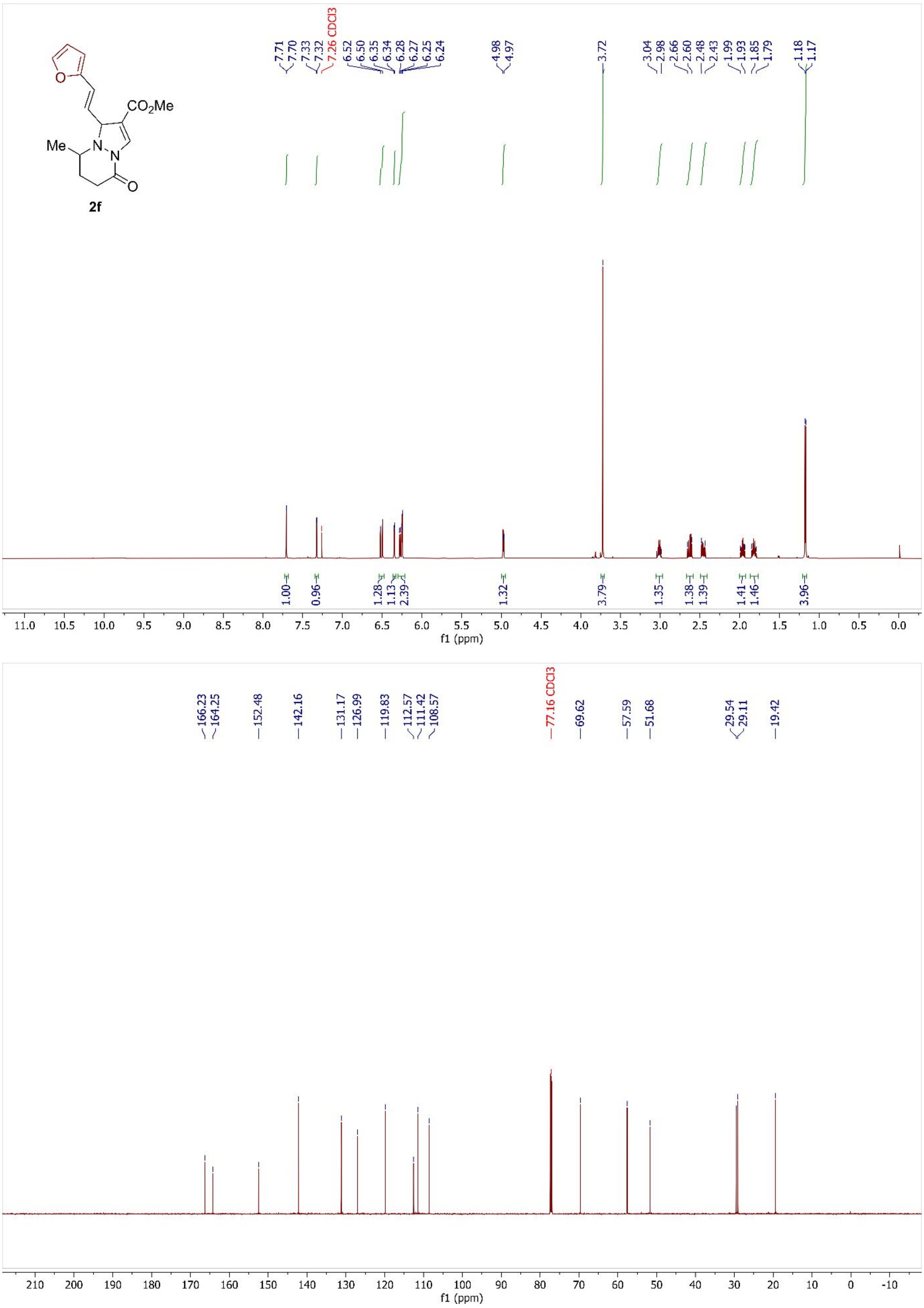

Figure S27. ^{1}H and $^{13}C\{1H\}$ NMR (600 MHz, $CDCl_3$) of compound **2f**.

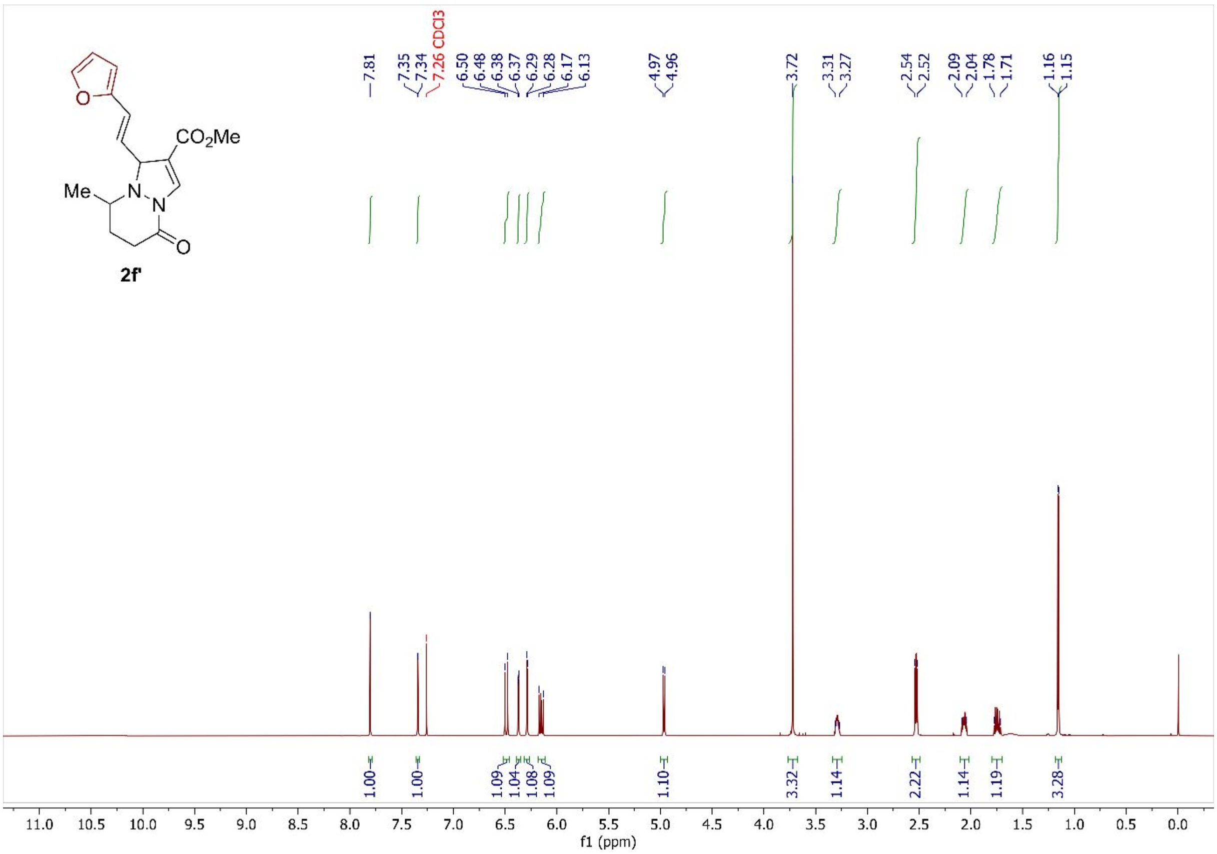

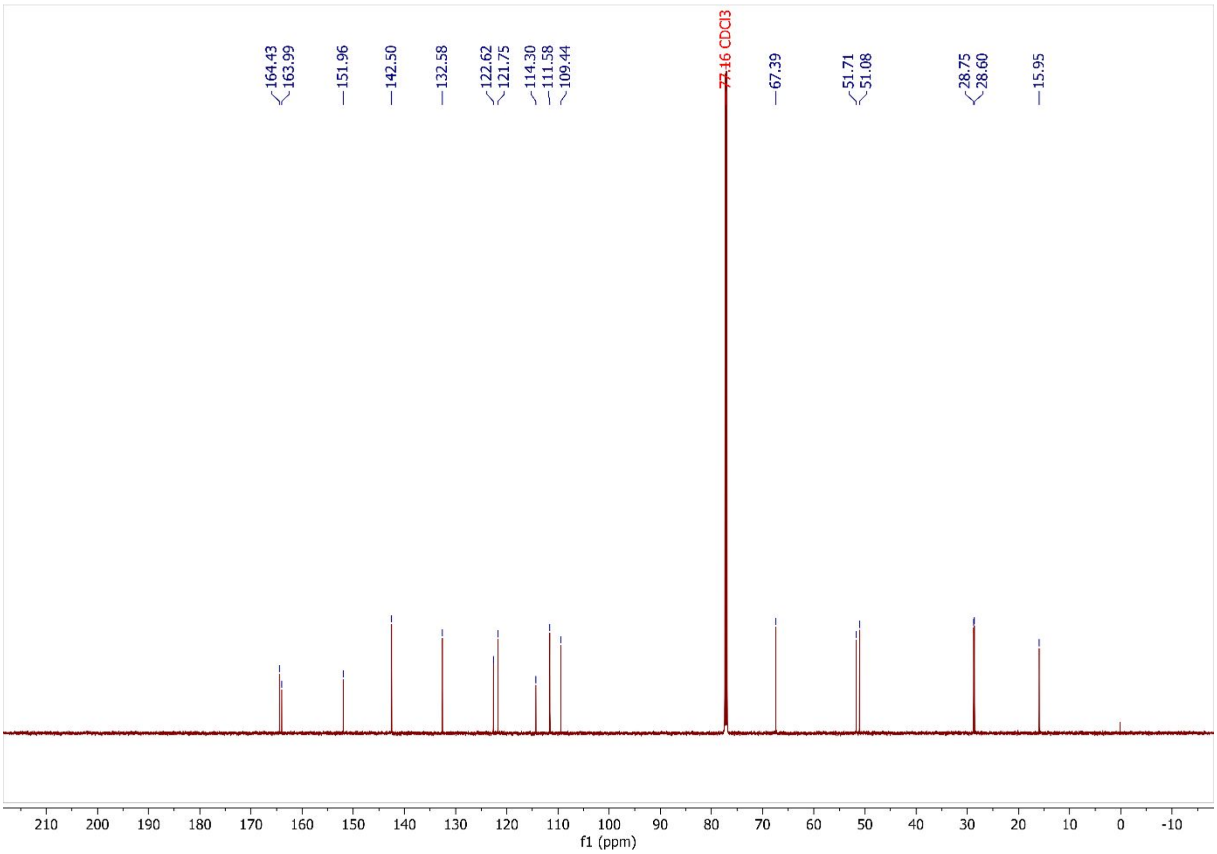

Figure S28. ^{1}H and $^{13}C\{1H\}$ NMR (600 MHz, $CDCl_3$) of compound **2f'**.

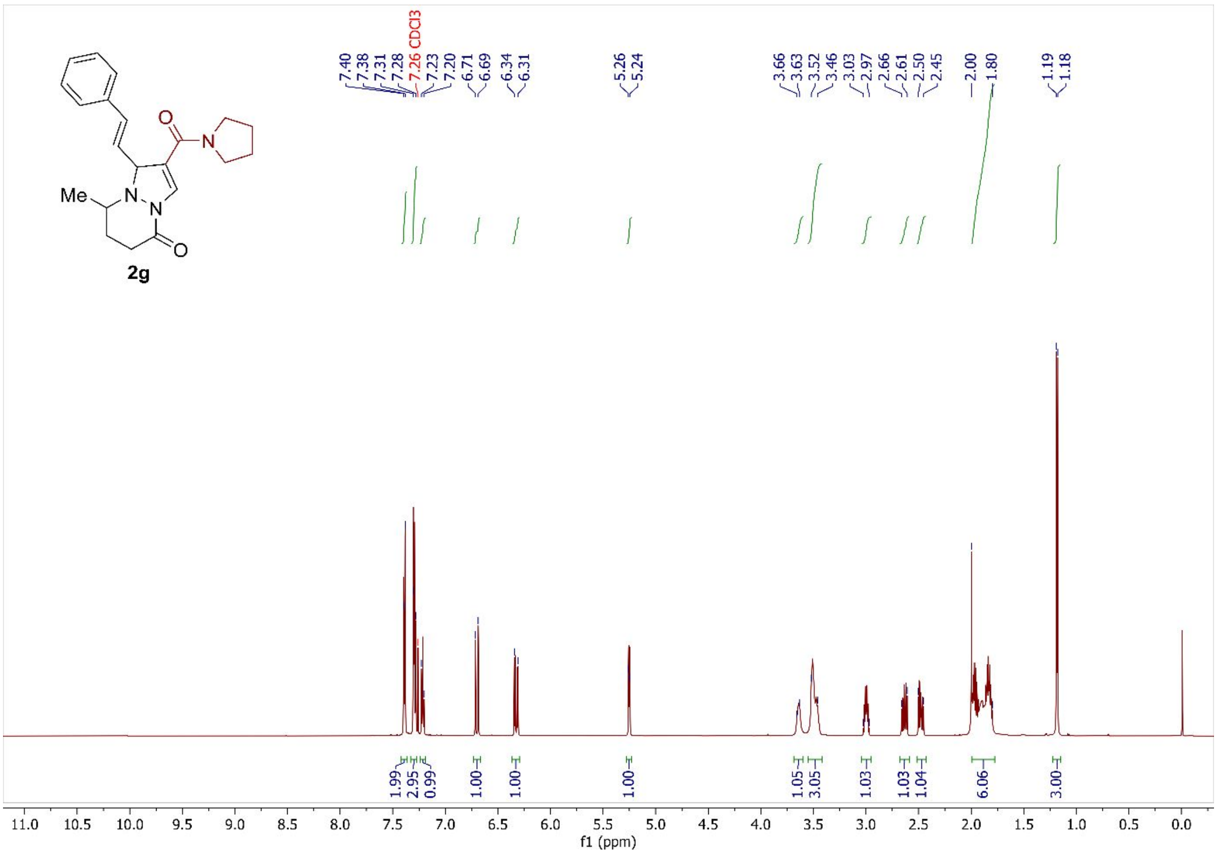

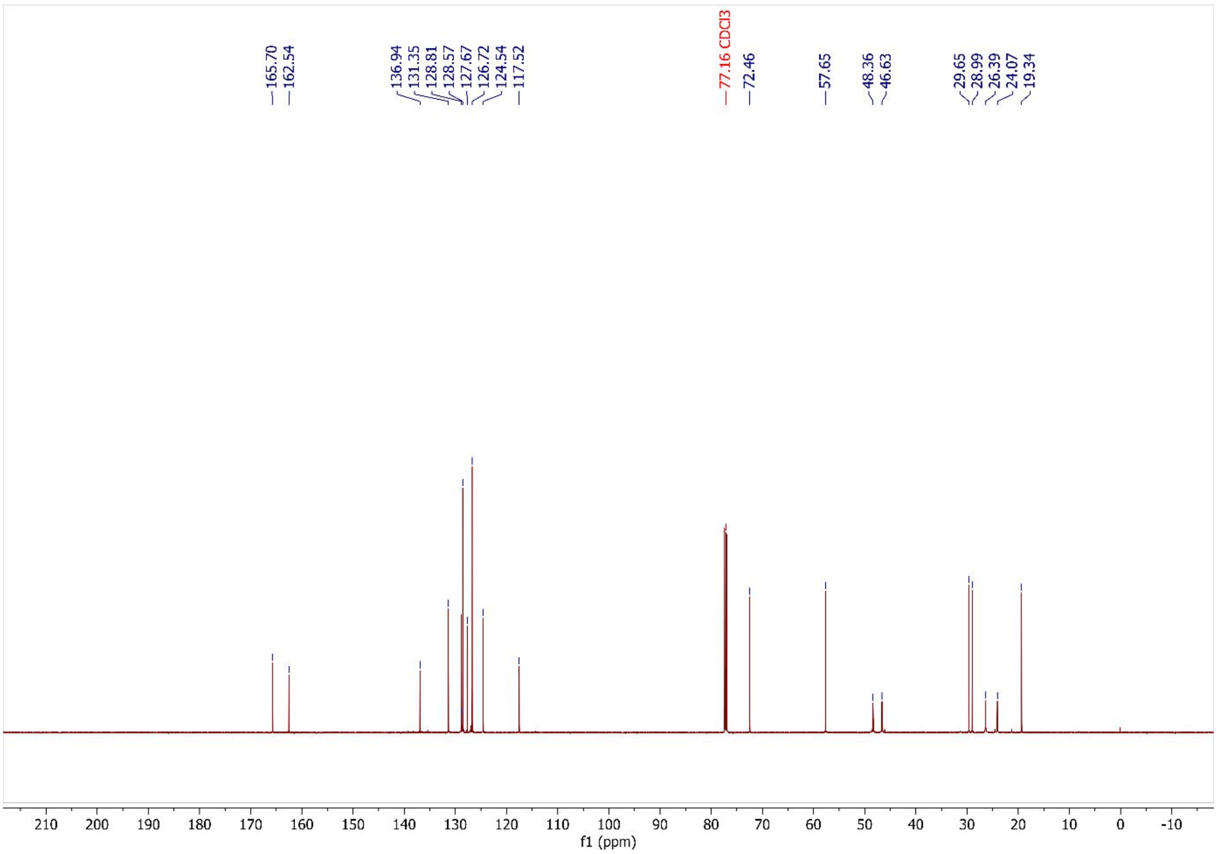

Figure S29. ^{1}H and $^{13}C\{1H\}$ NMR (600 MHz, $CDCl_3$) of compound **2g**.

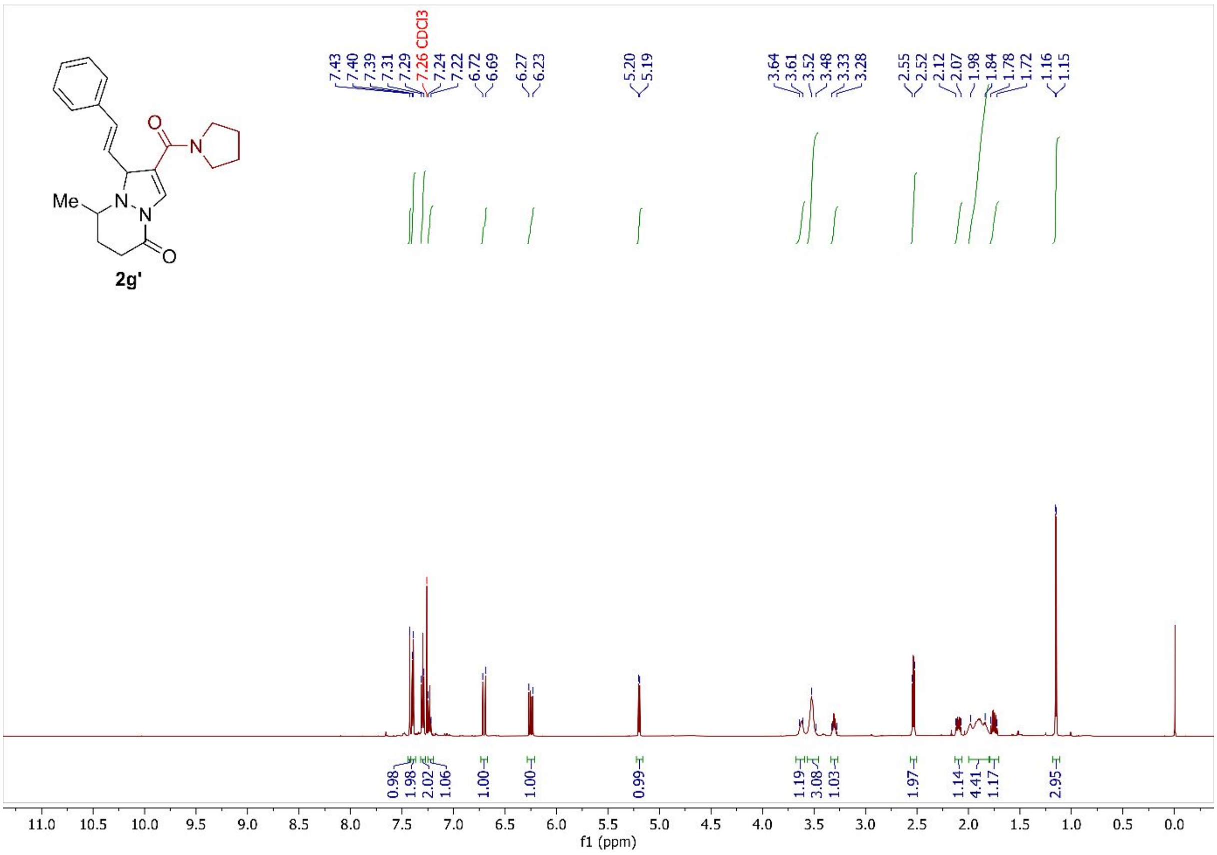

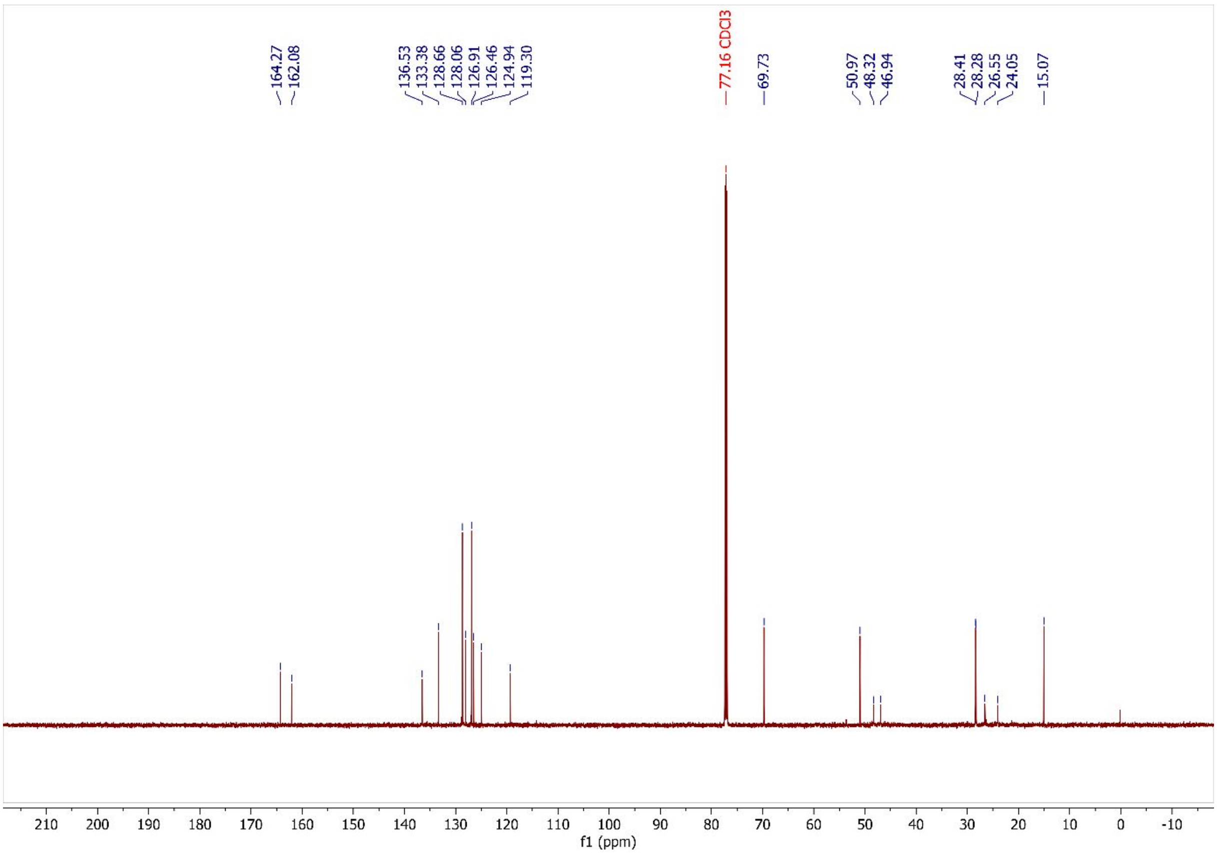

Figure S30. ^{1}H and $^{13}C\{1H\}$ NMR (600 MHz, $CDCl_3$) of compound **2g’**.

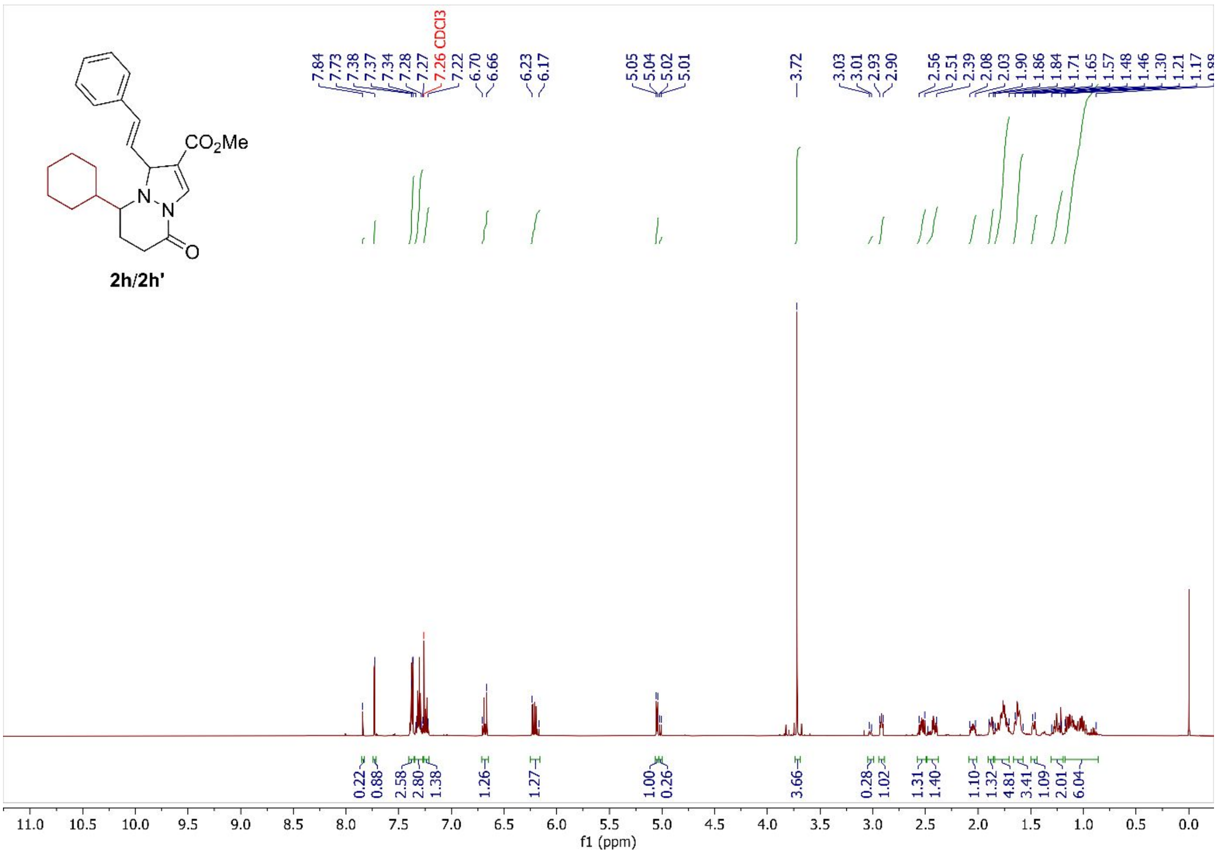

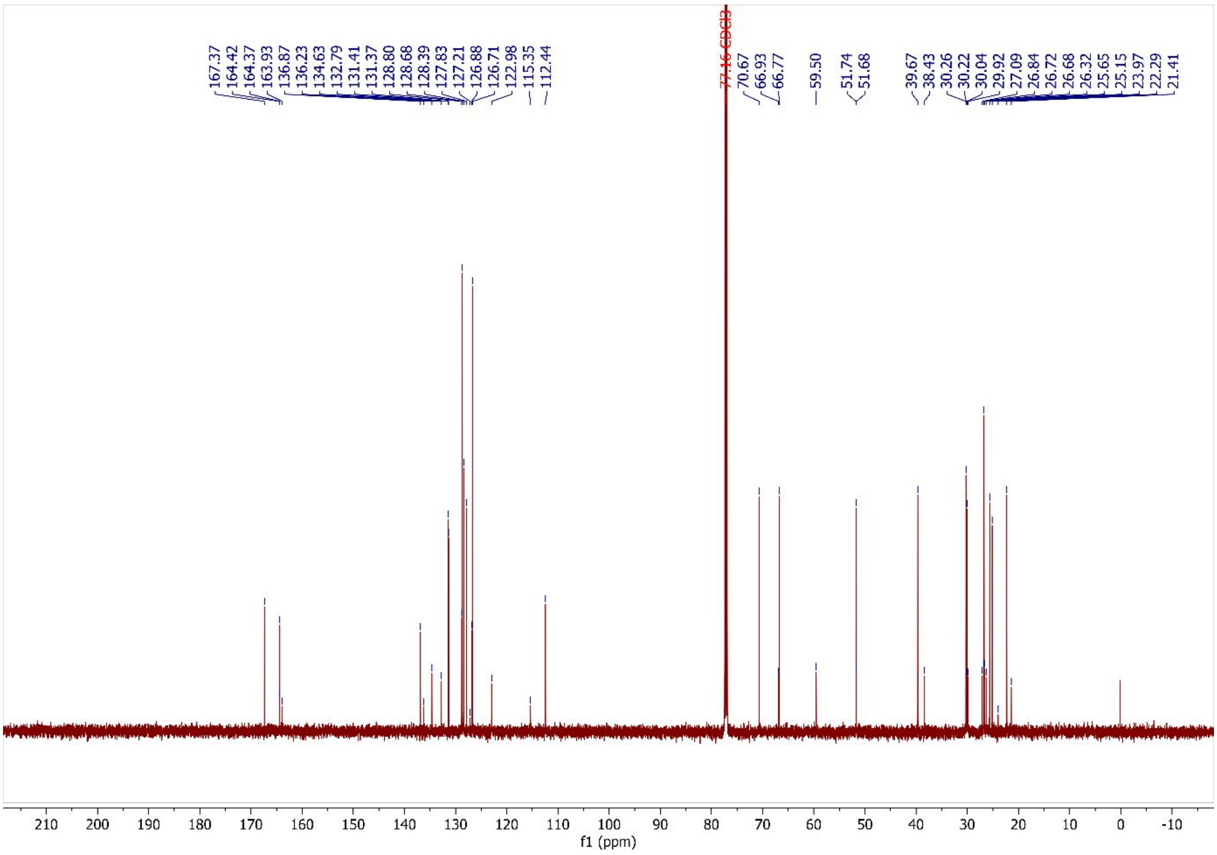

Figure S31. ^{1}H and $^{13}C\{1H\}$ NMR (600 MHz, $CDCl_3$) of compounds **2h**/**2h'**.

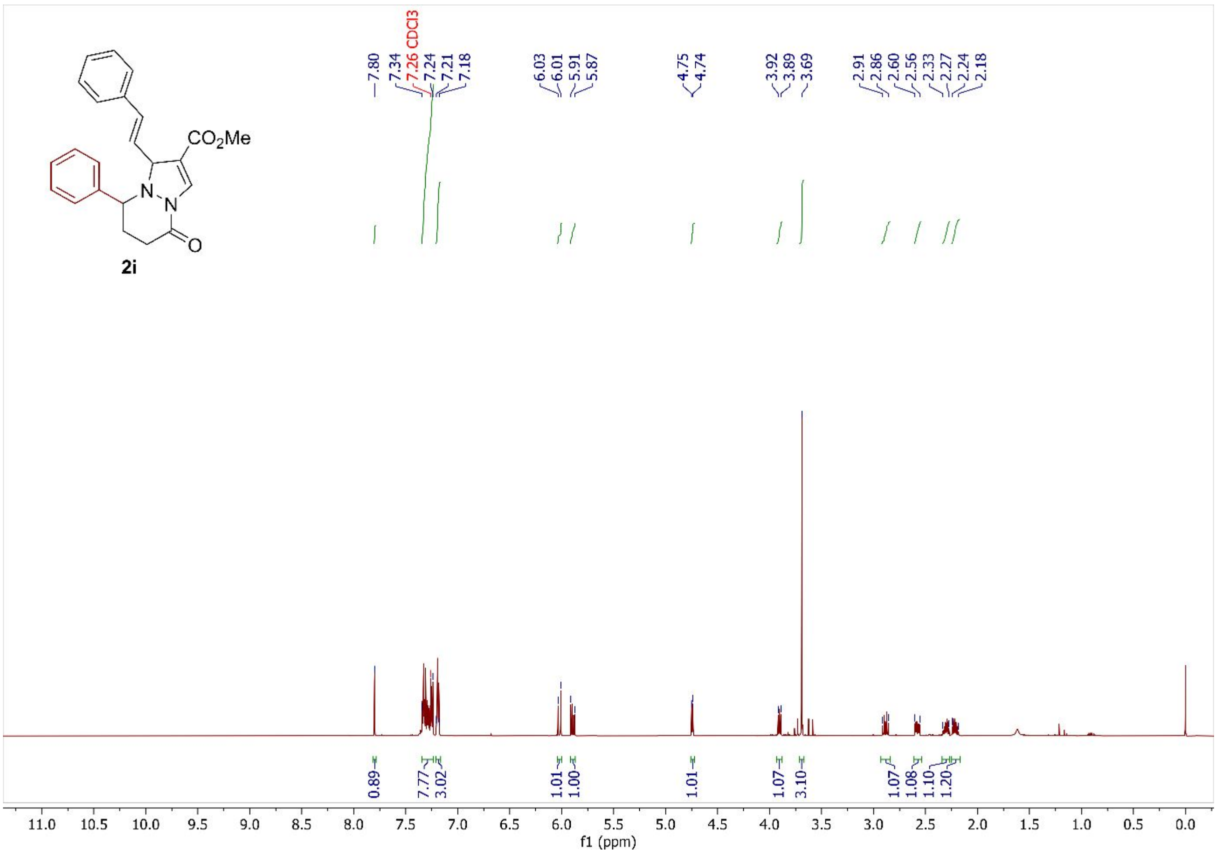

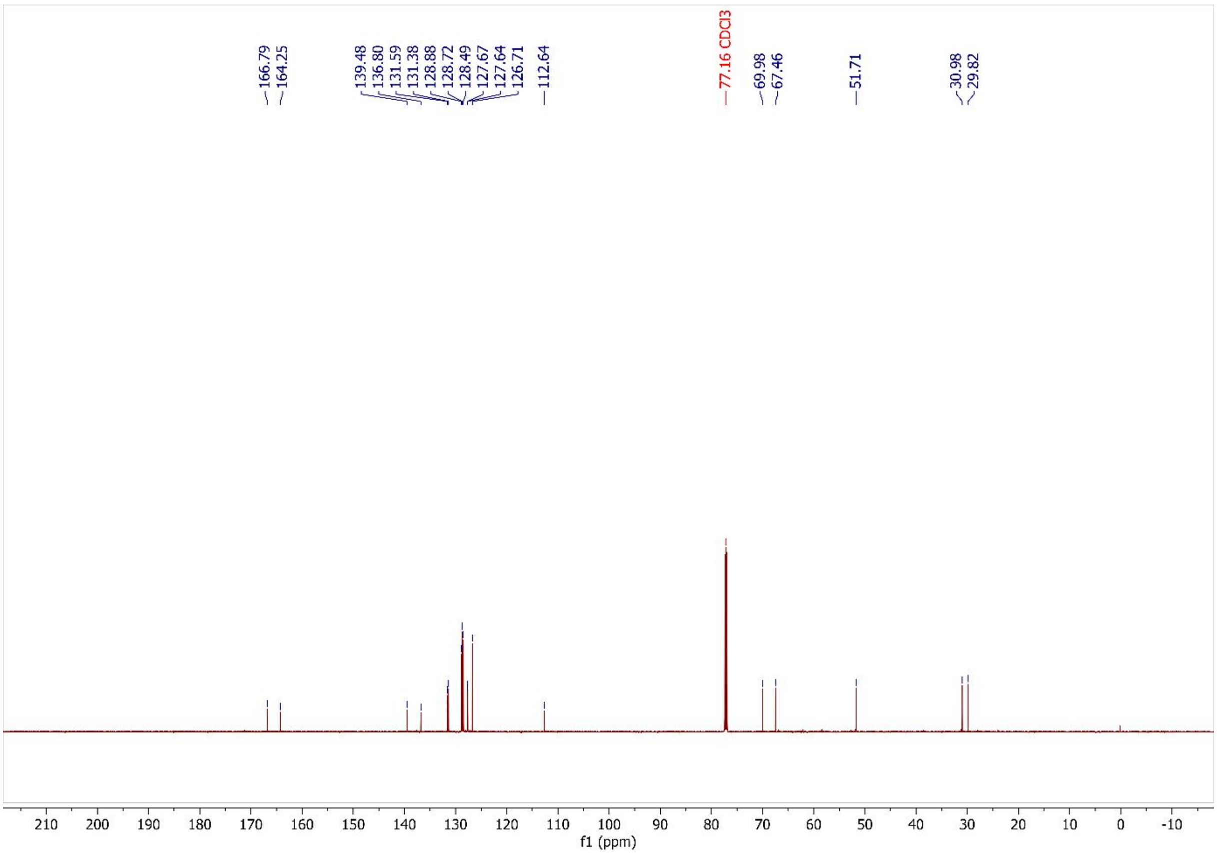

Figure S32. ^{1}H and $^{13}C\{1H\}$ NMR (600 MHz, $CDCl_3$) of compound **2i**.

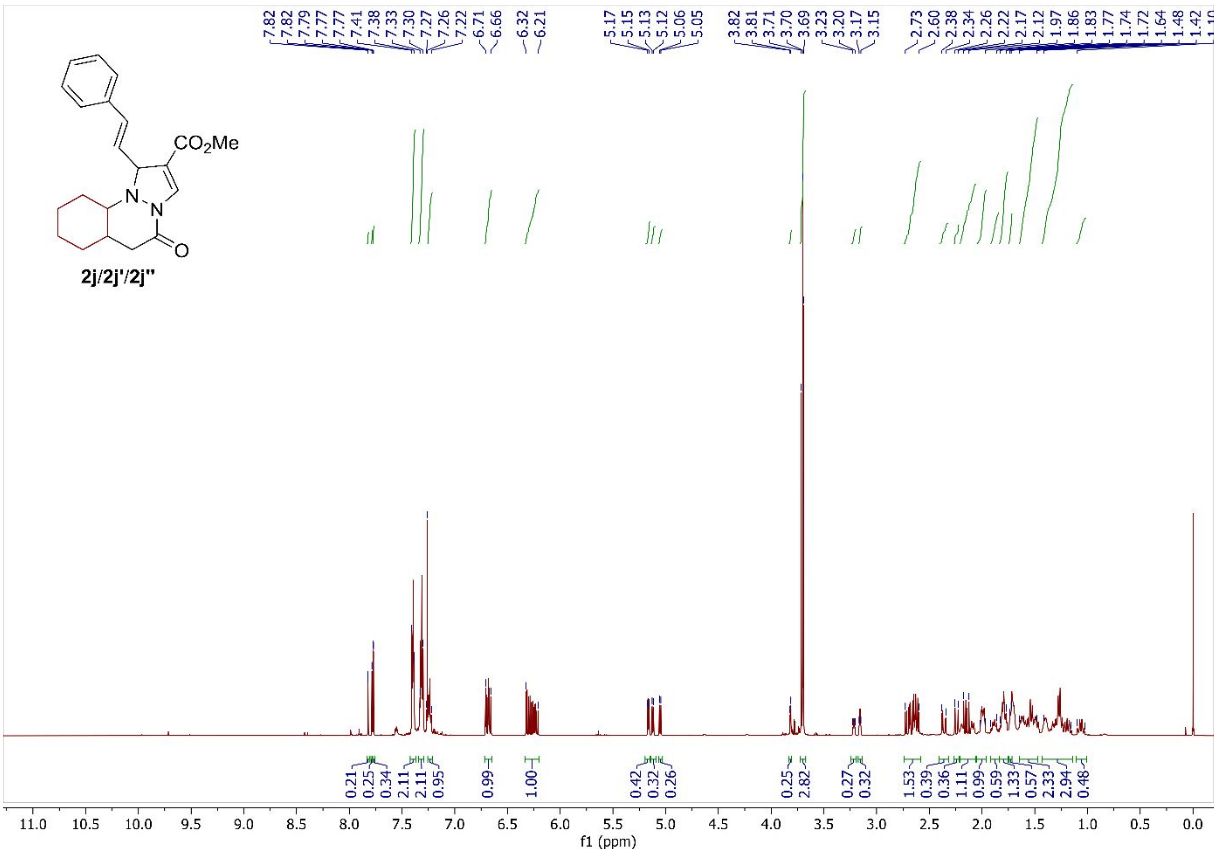

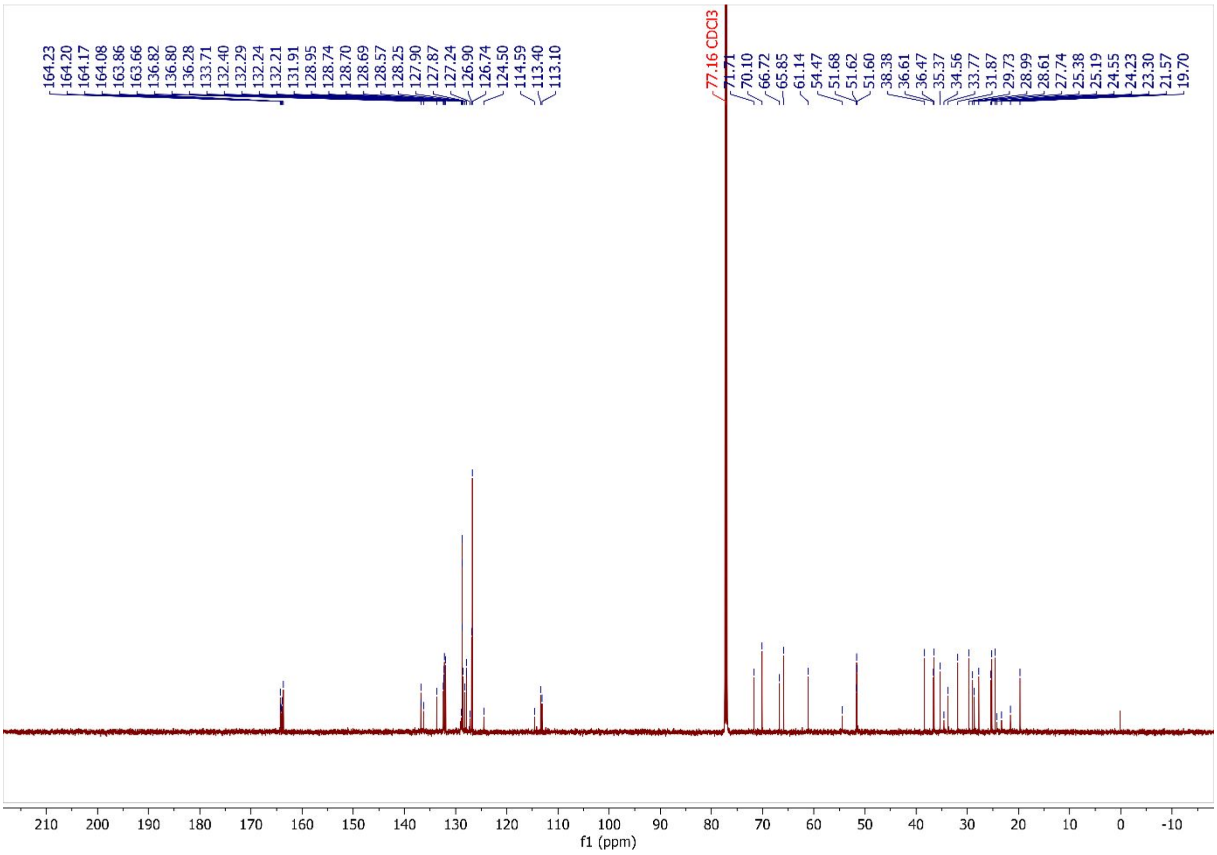

Figure S33. ^{1}H and ^{13}C{1H} NMR (600 MHz, $CDCl_3$) of compounds **2j**/**2j'**/**2j''**.

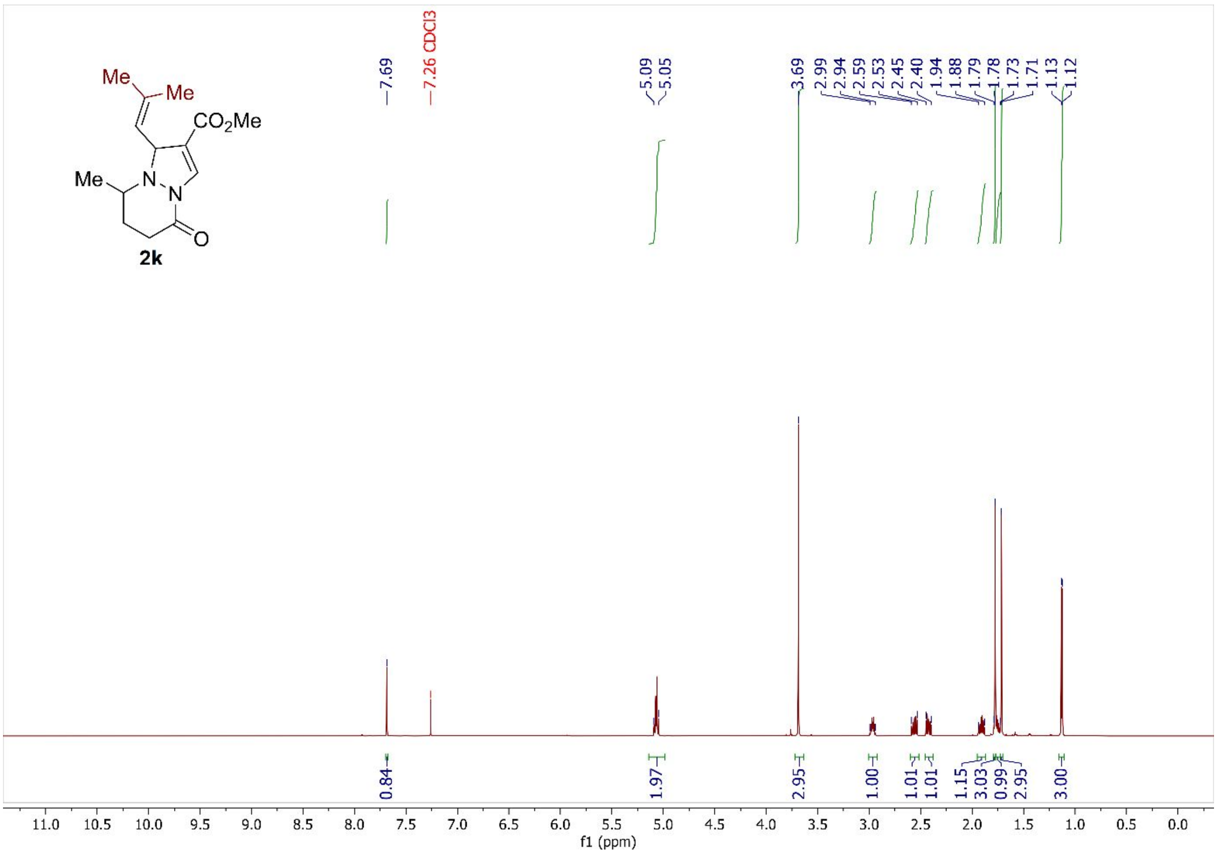

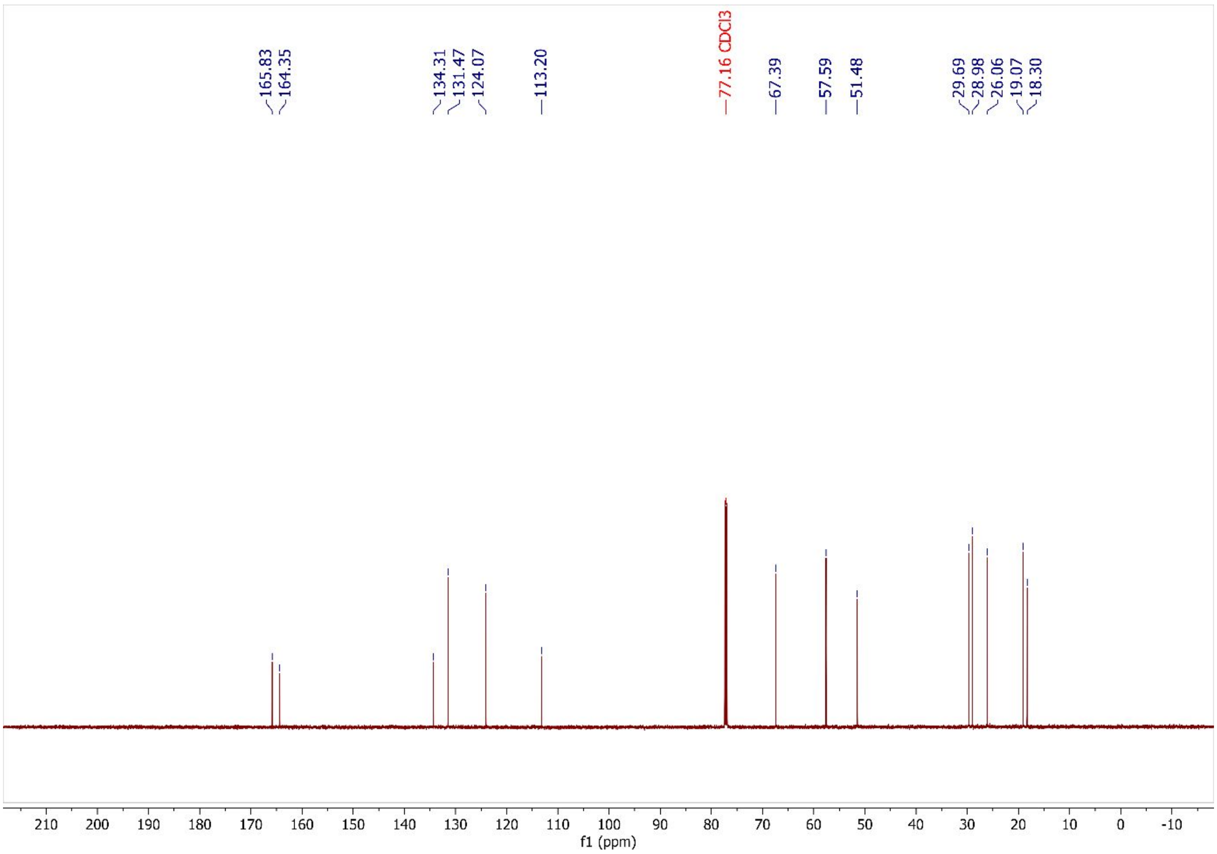

Figure S34. ^{1}H and $^{13}C\{1H\}$ NMR (500 MHz, $CDCl_3$) of compound **2k**.

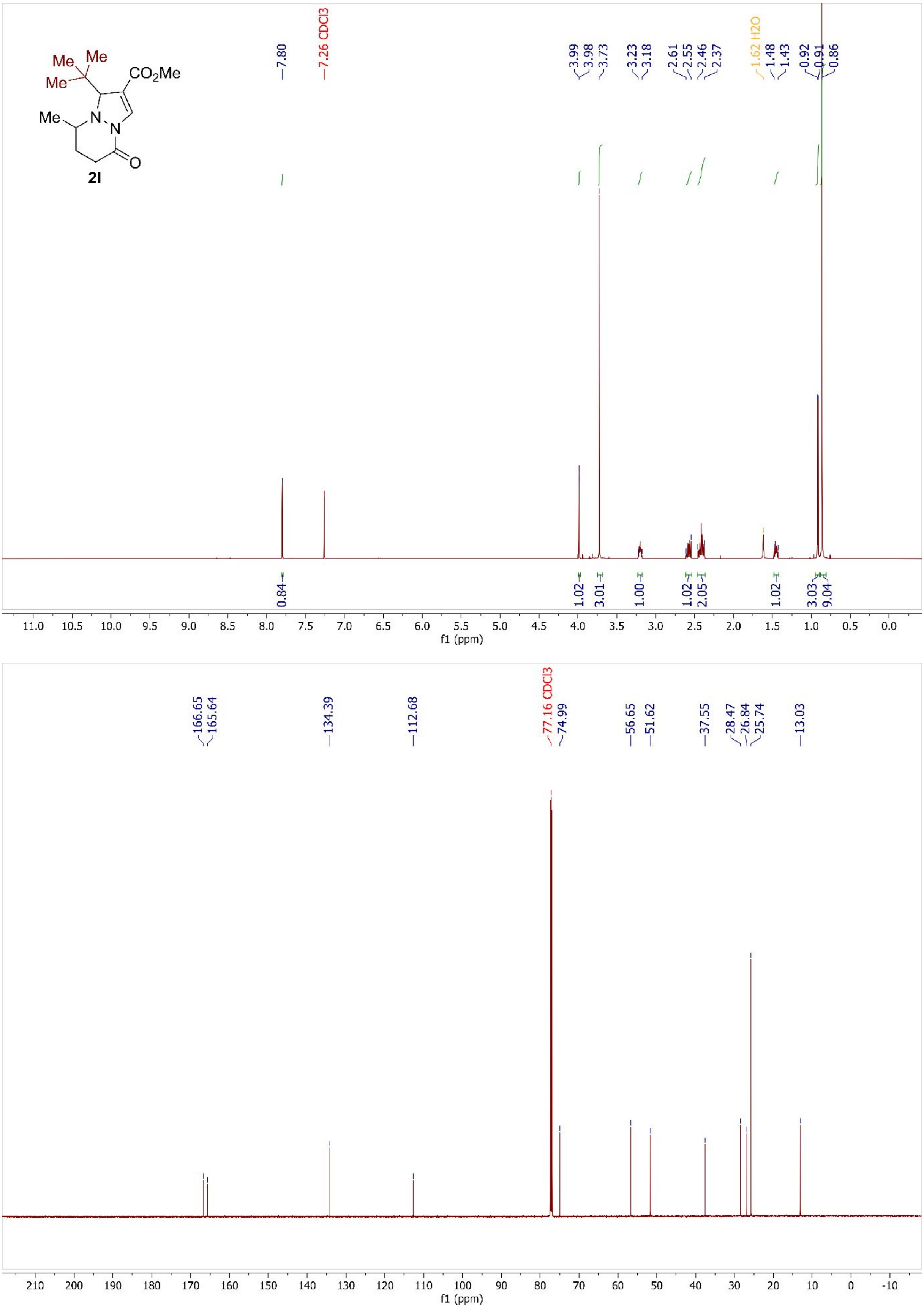

Figure S35. ^{1}H and $^{13}C\{1H\}$ NMR (600 MHz, $CDCl_3$) of compound **2l**.

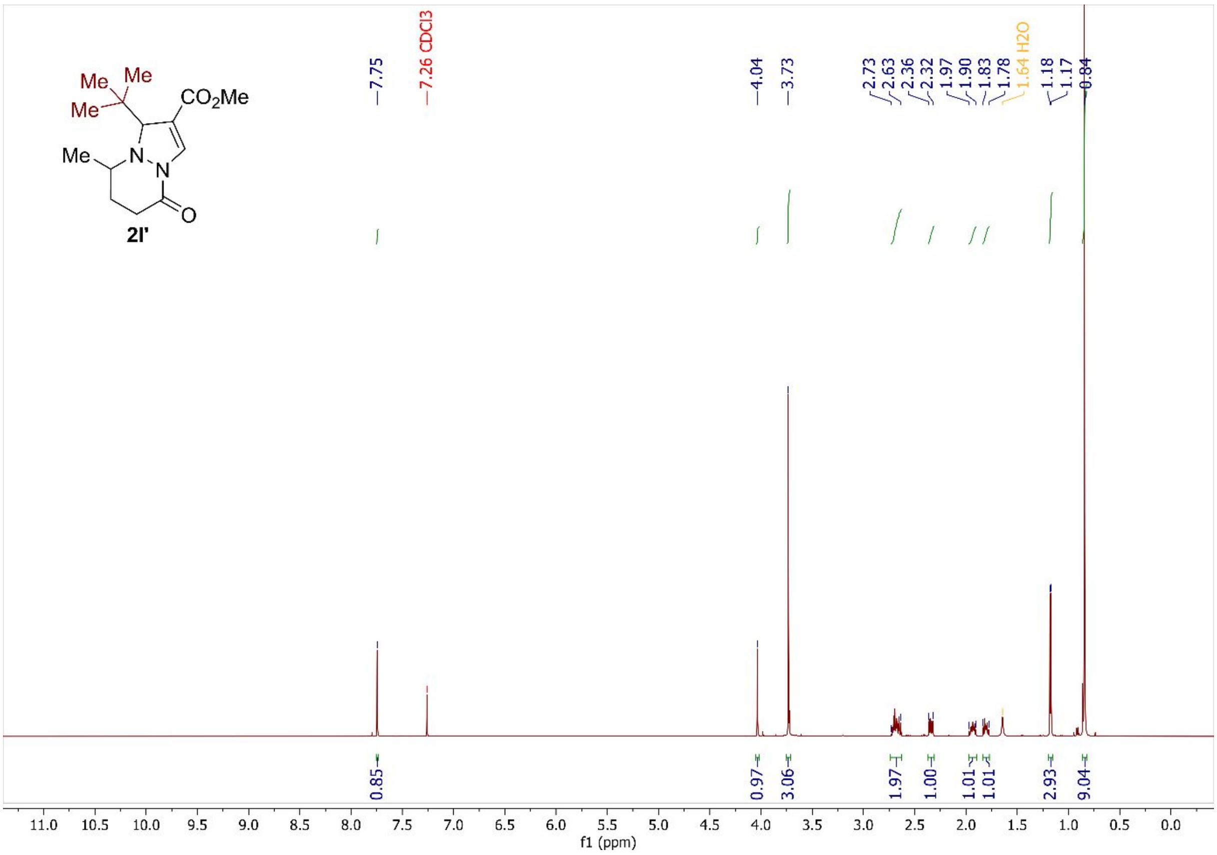

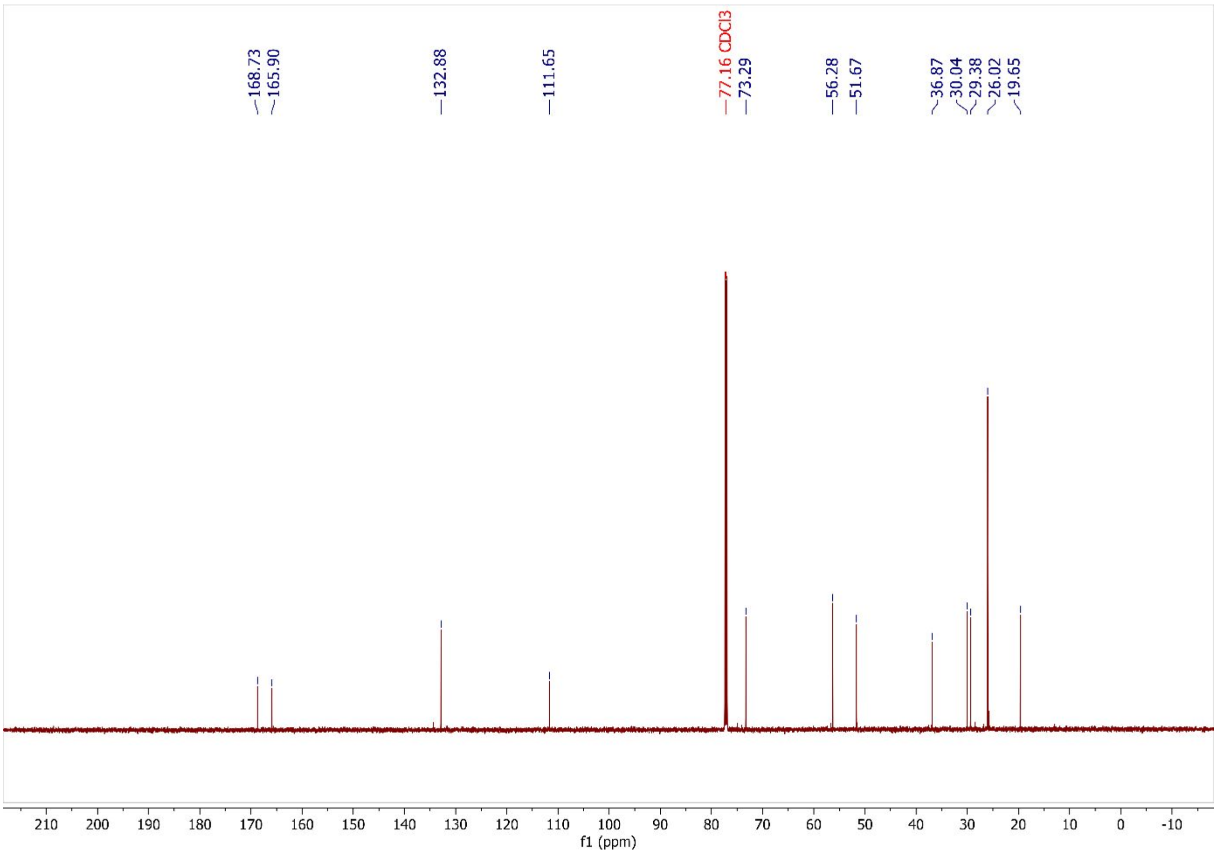

Figure S36. ^{1}H and $^{13}C\{1H\}$ NMR (600 MHz, $CDCl_3$) of compound **2l'**. The spectra show minor trace resonances corresponding to the major diastereoisomer **2l**.

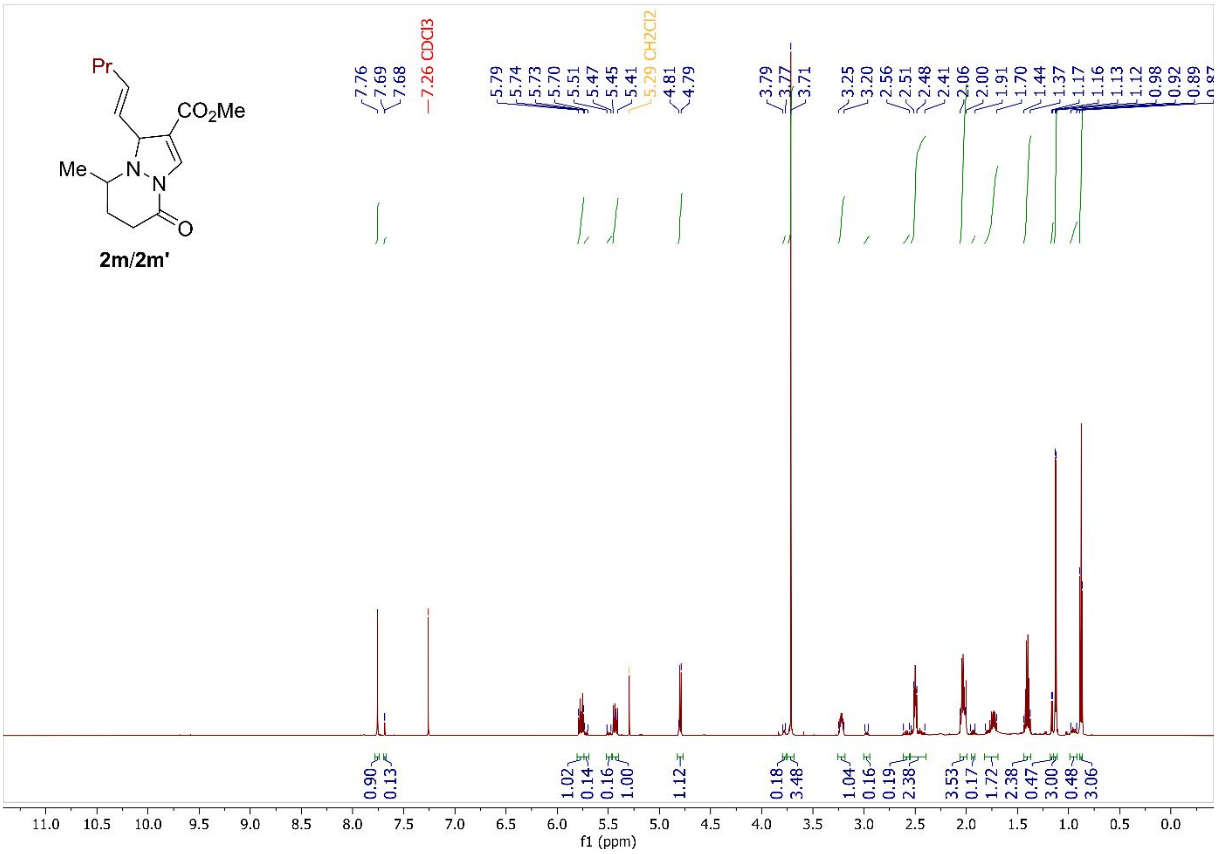

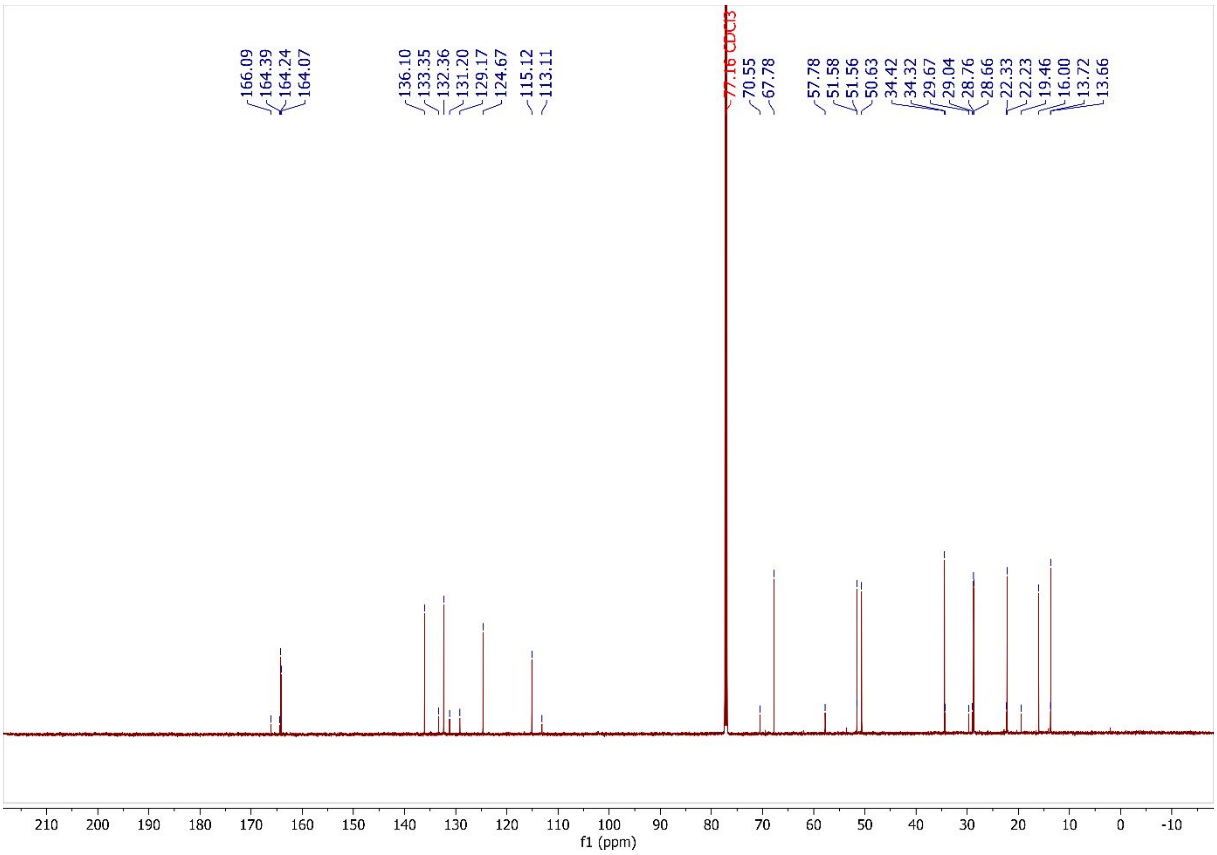

Figure S37. ^{1}H and $^{13}C\{1H\}$ NMR (600 MHz, $CDCl_3$) of compounds **2m**/**2m’**.

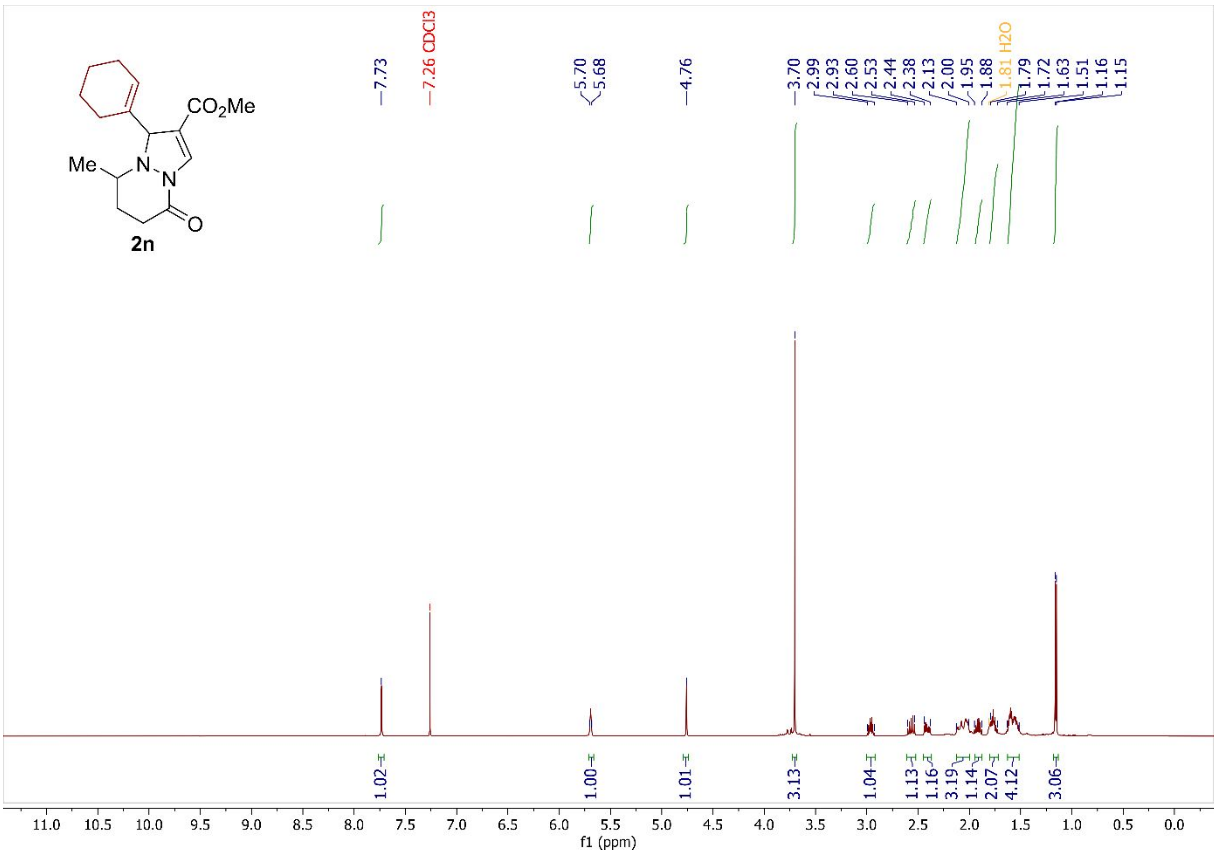

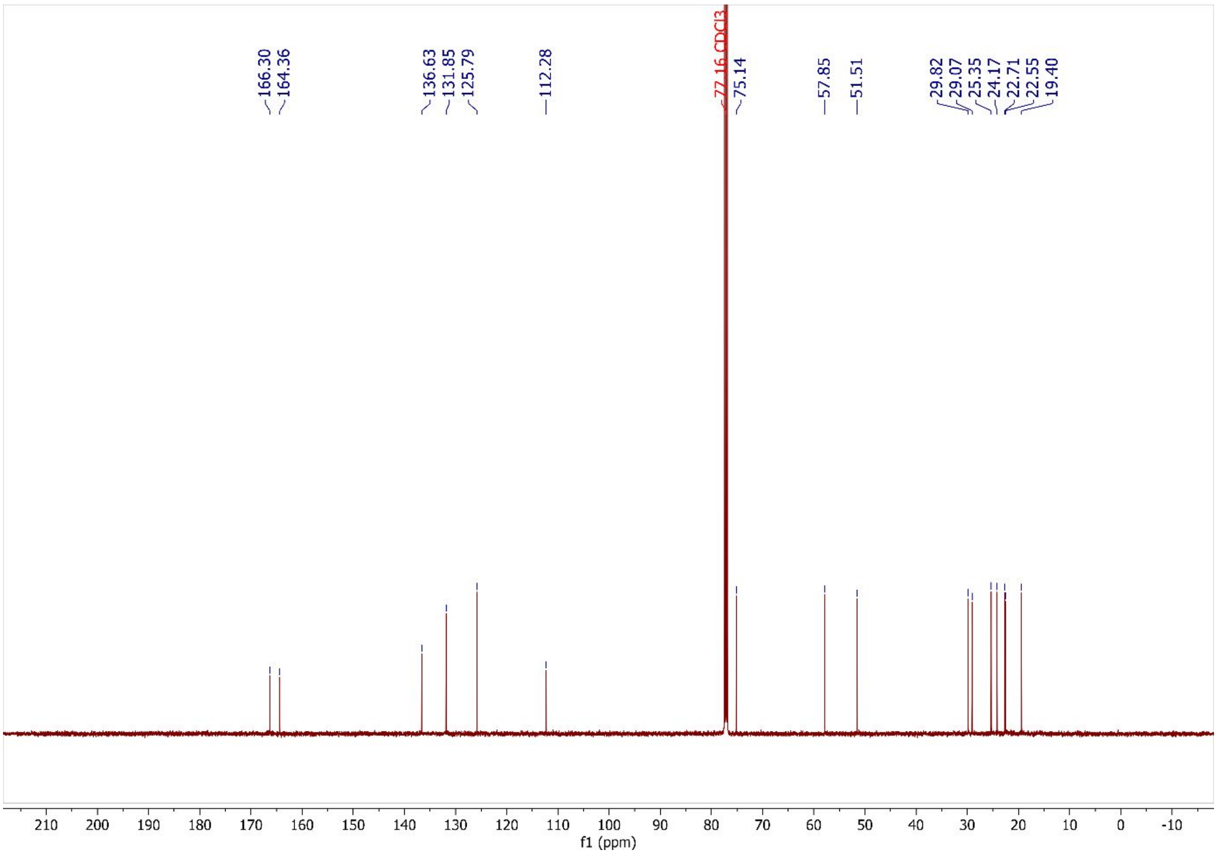

Figure S38. ^{1}H and $^{13}C\{1H\}$ NMR (500 MHz, $CDCl_3$) of compound **2n**.

16.4 1,2-diazepine **3a**

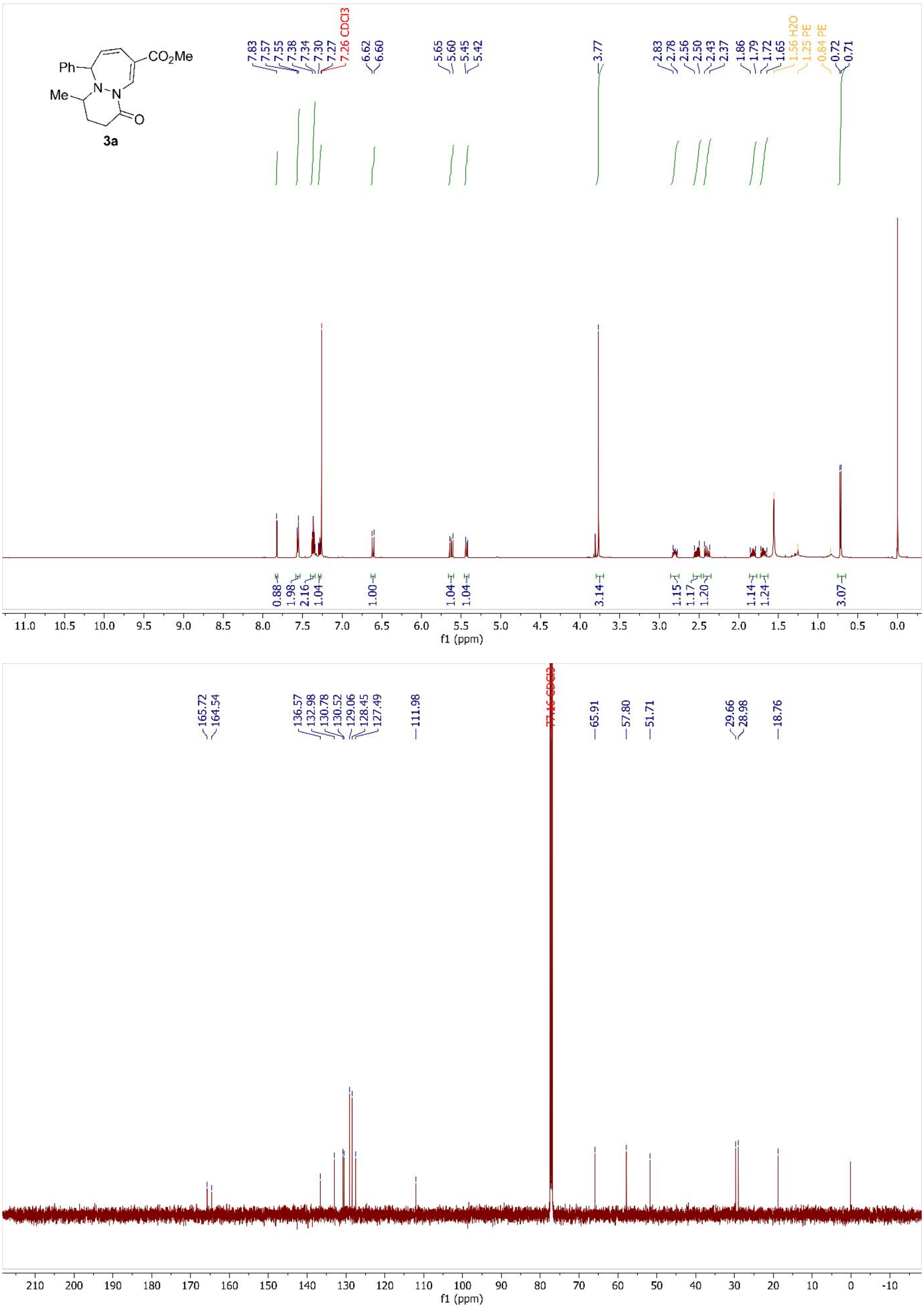

Figure S39. ^{1}H and $^{13}C\{1H\}$ NMR (600 MHz, $CDCl_3$) of compound **3a**.

17.5 6,5,4-tricyclic products **4**

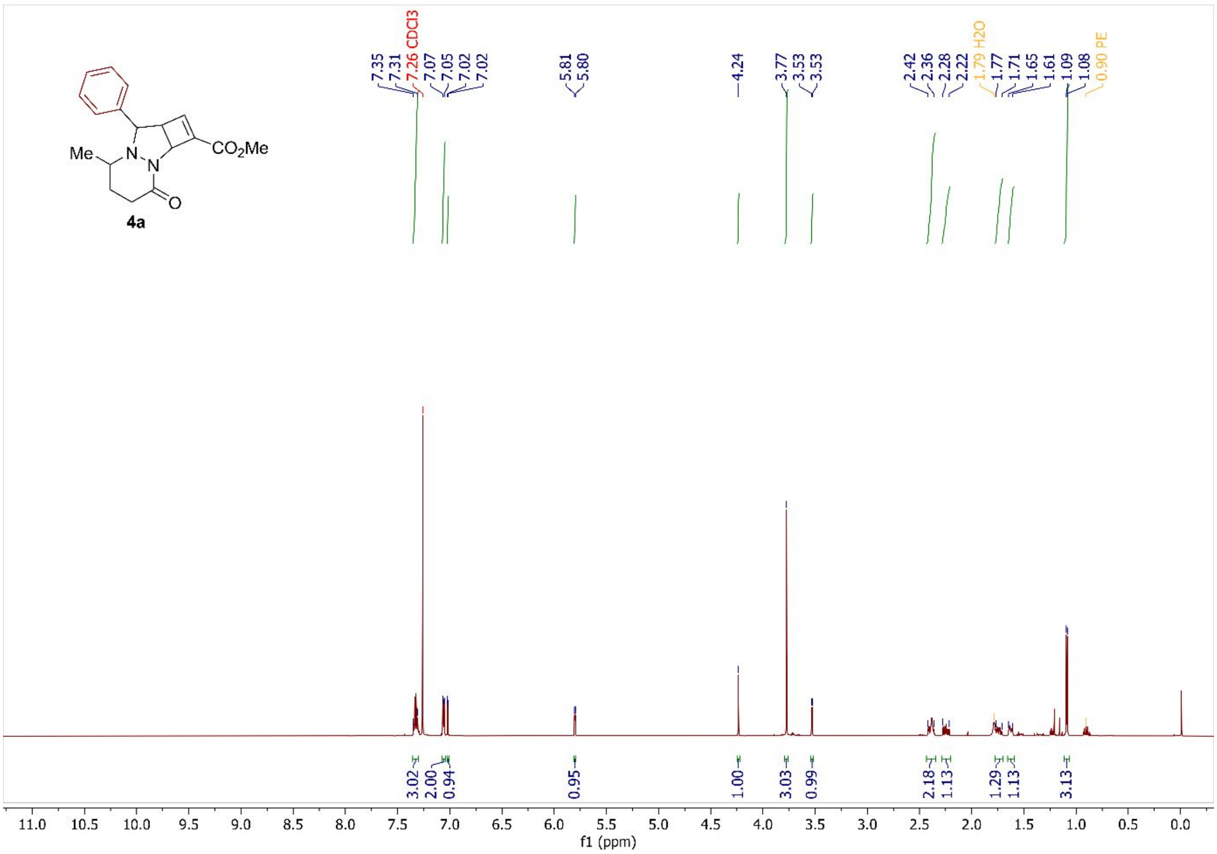

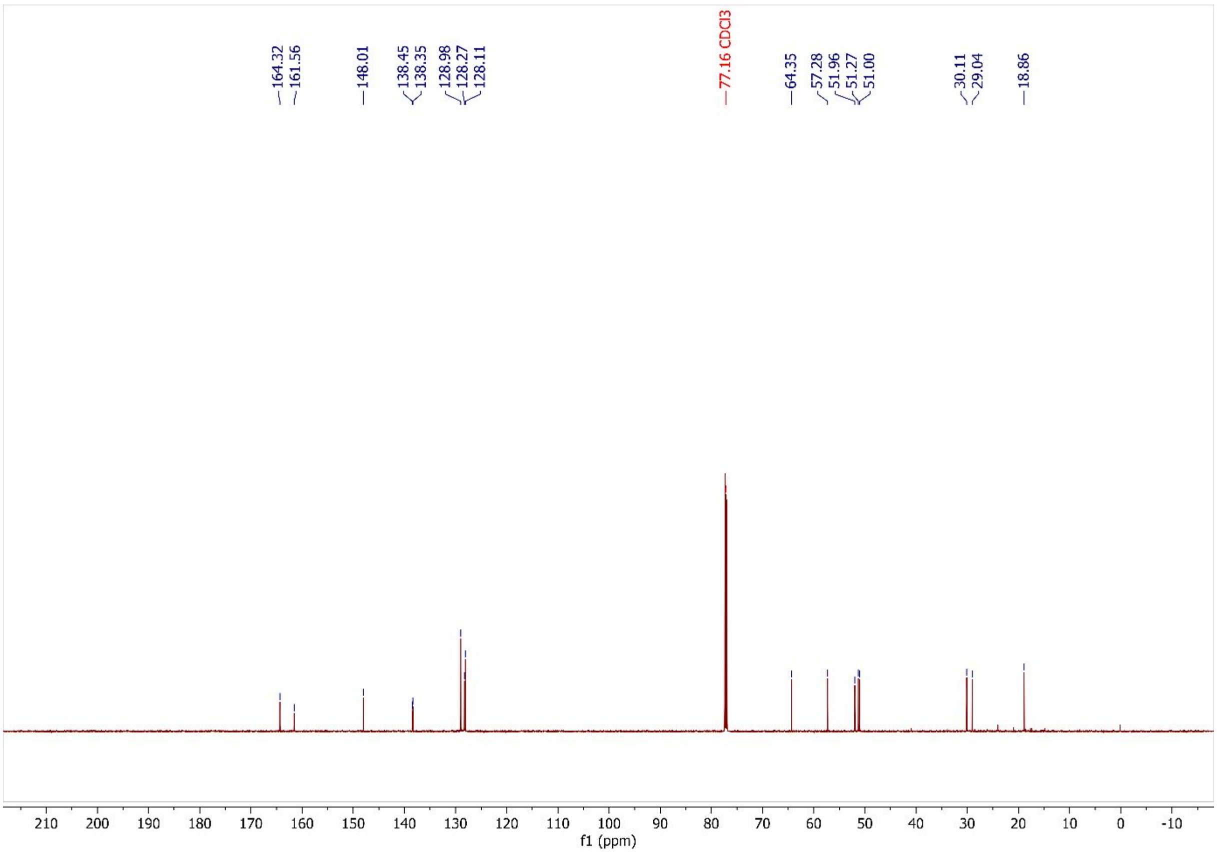

Figure S40. ^{1}H and ^{13}C{1H} NMR (600 MHz, $CDCl_3$) of compound **4a**.

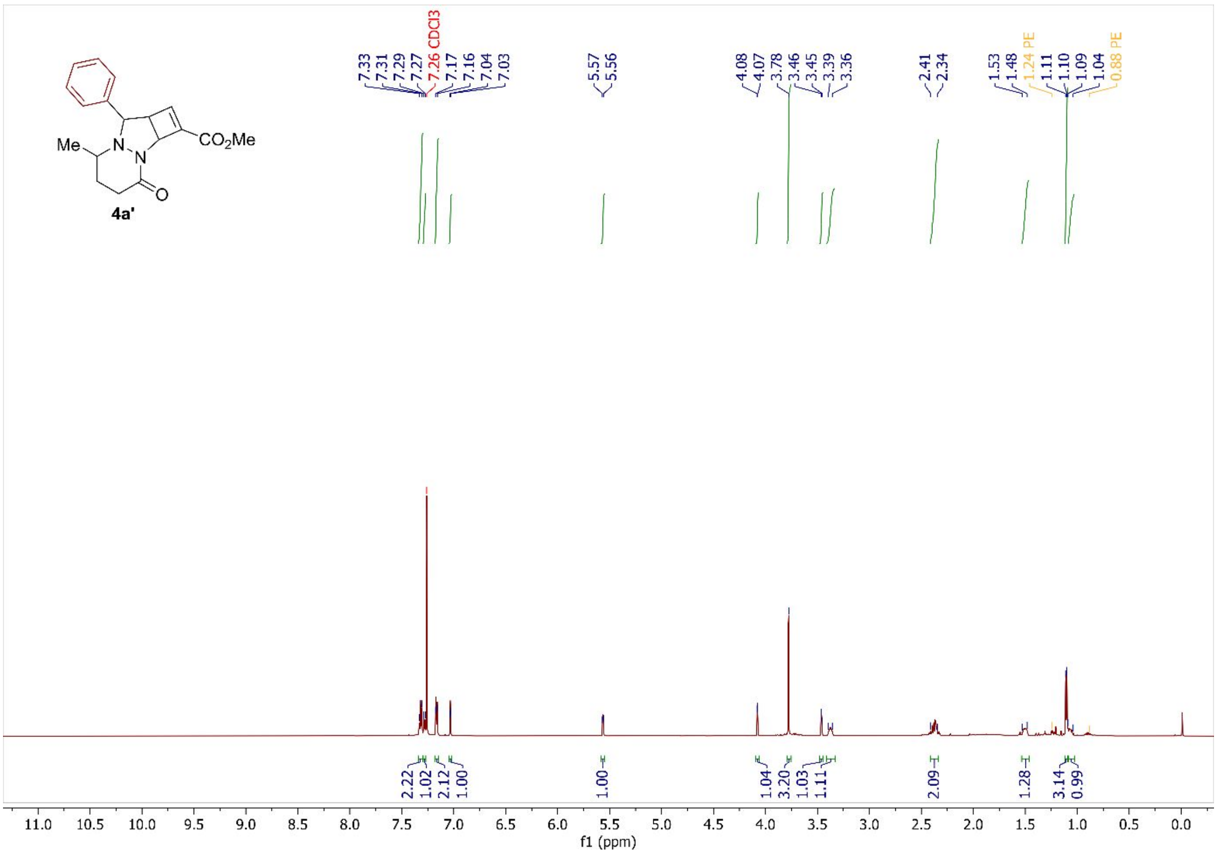

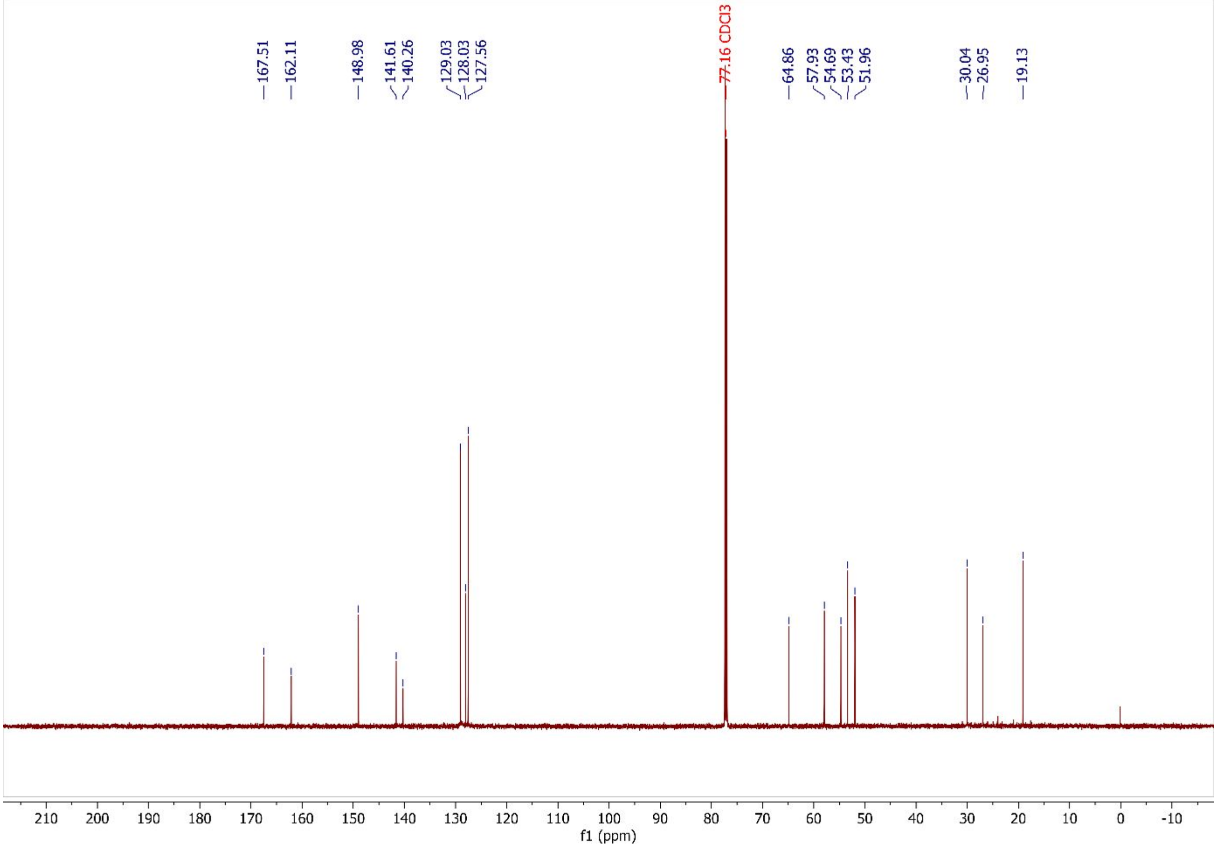

Figure S41. ^{1}H and $^{13}C\{1H\}$ NMR (600 MHz, $CDCl_3$) of compound **4a’**.

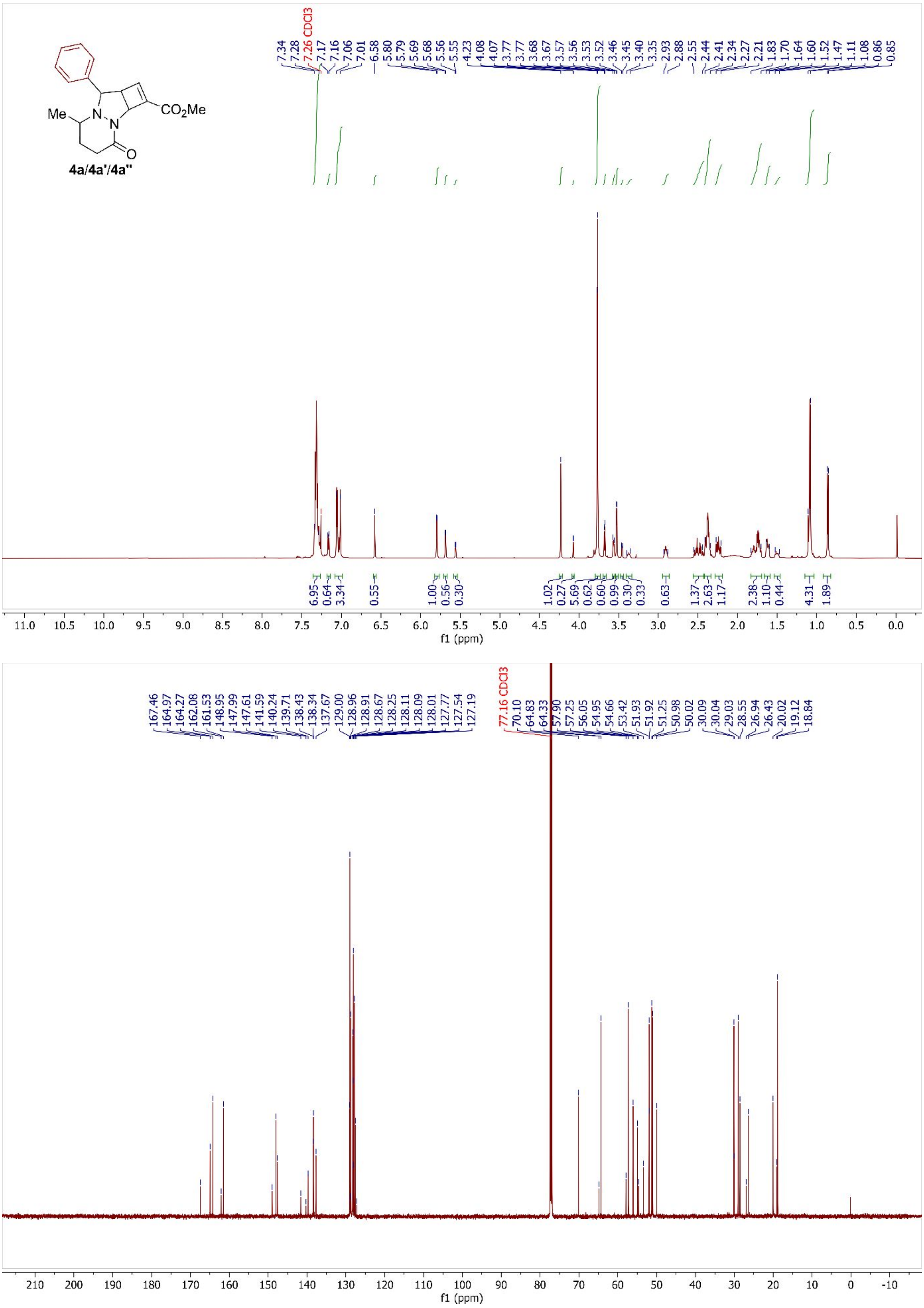

Figure S42. ^1H and $^{13}C\{^1H\}$ NMR (600 MHz, $CDCl_3$) of compounds **4a**/**4a'**/**4a''**.

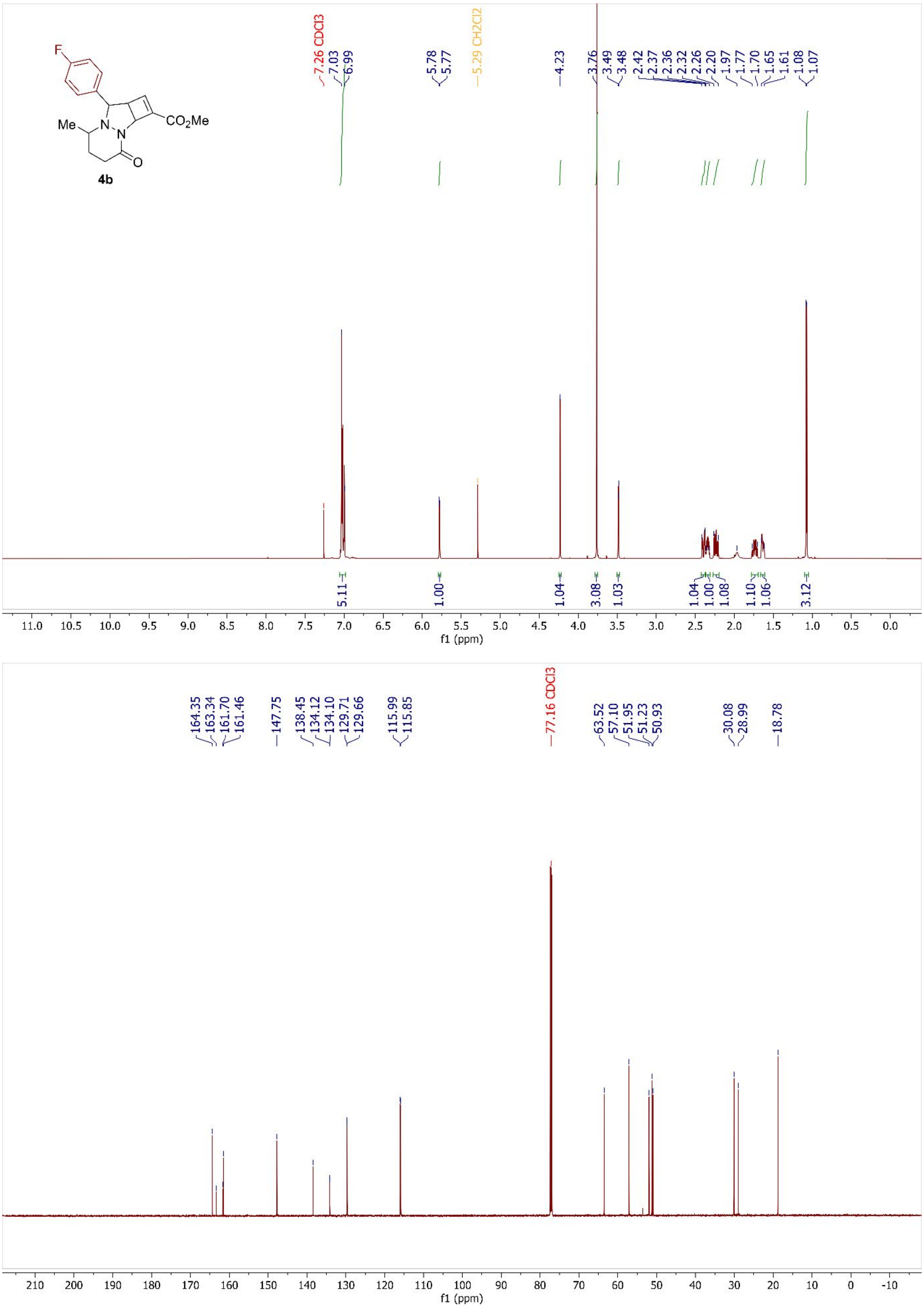

Figure S43. ^{1}H and $^{13}C\{1H\}$ NMR (600 MHz, $CDCl_3$) of compound **4b**.

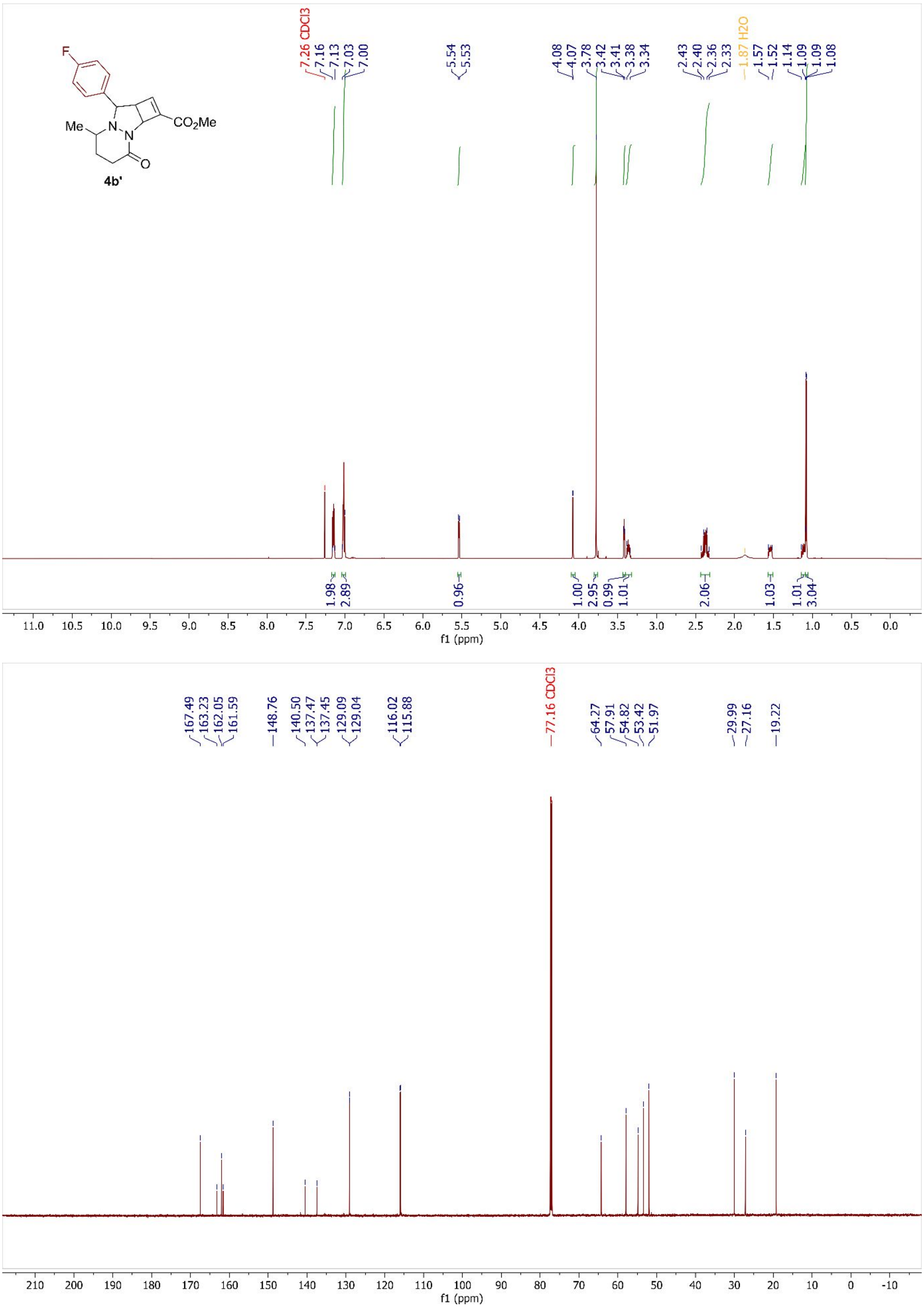

Figure S44. ^{1}H and $^{13}C\{1H\}$ NMR (600 MHz, $CDCl_3$) of compound **4b'**.

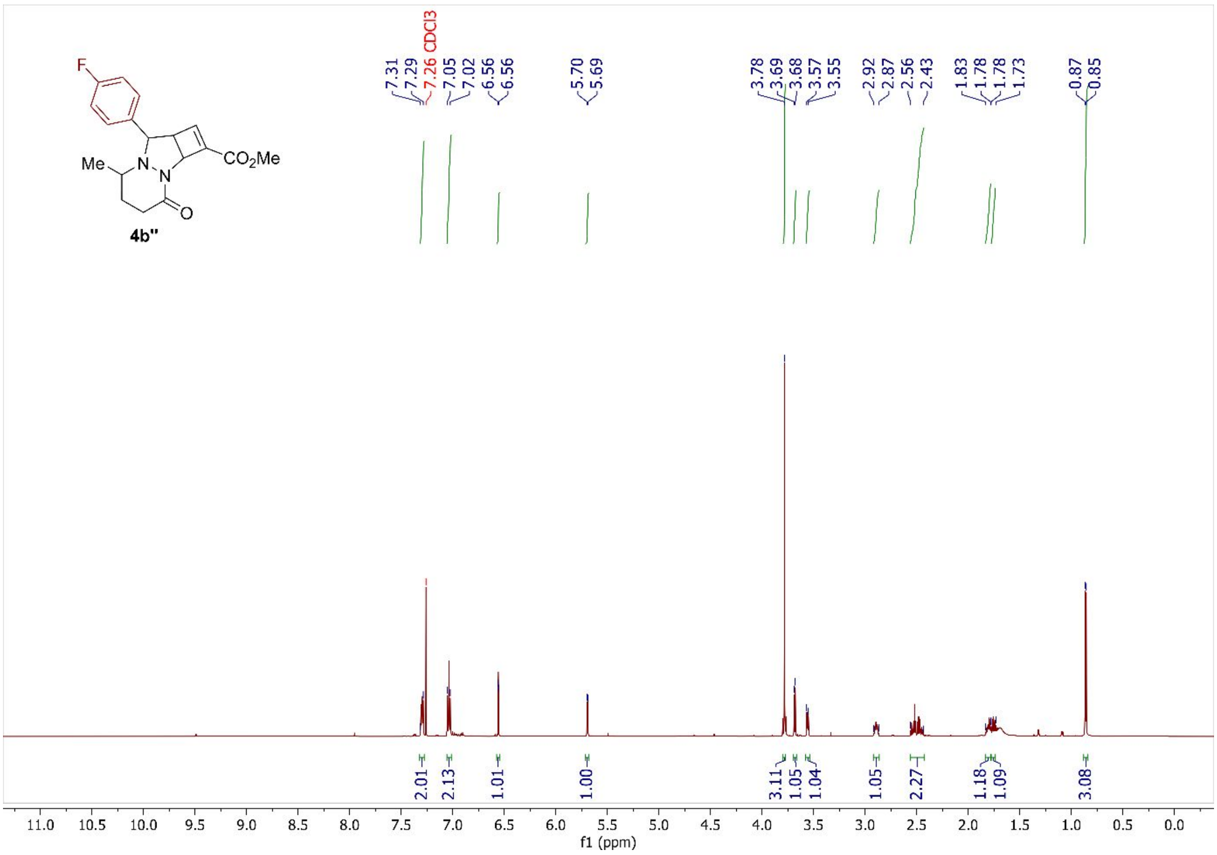

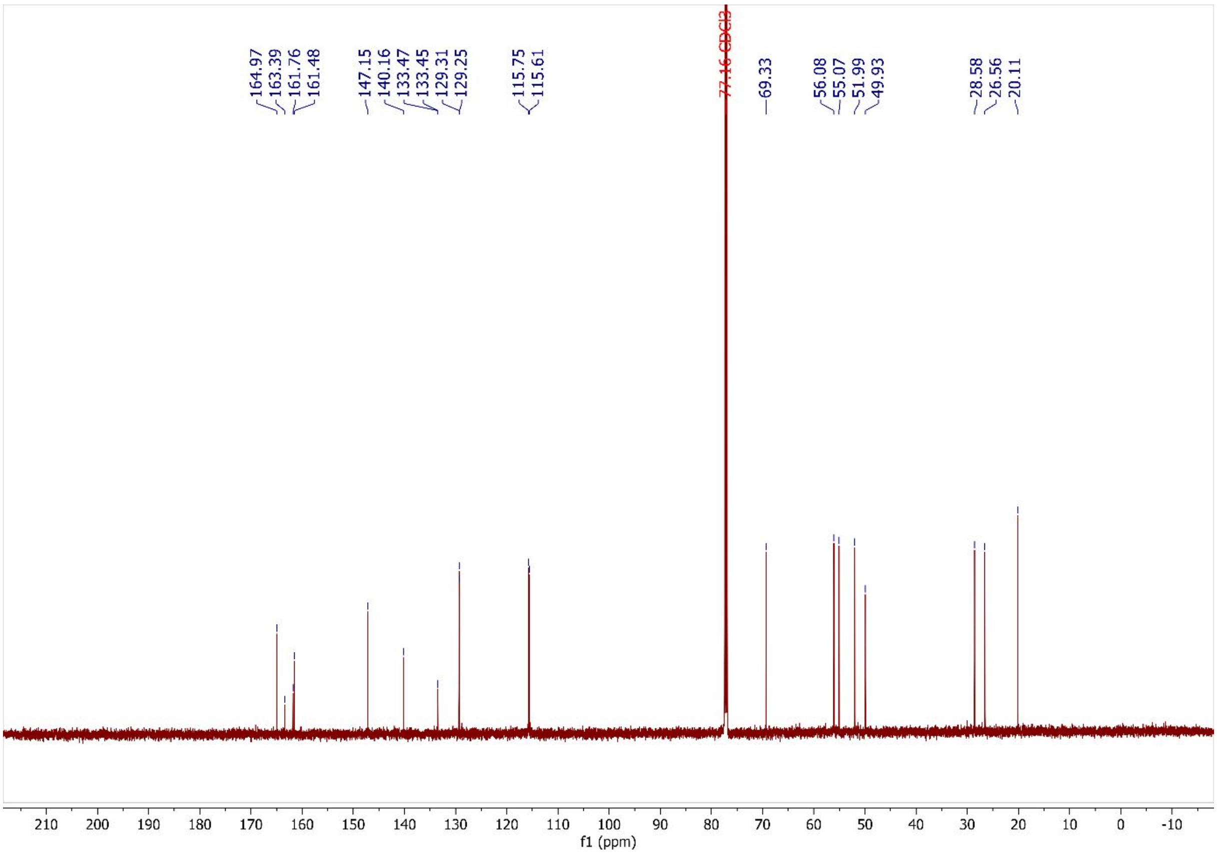

Figure S45. ^{1}H and $^{13}C\{1H\}$ NMR (600 MHz, $CDCl_3$) of compound **4b''**.

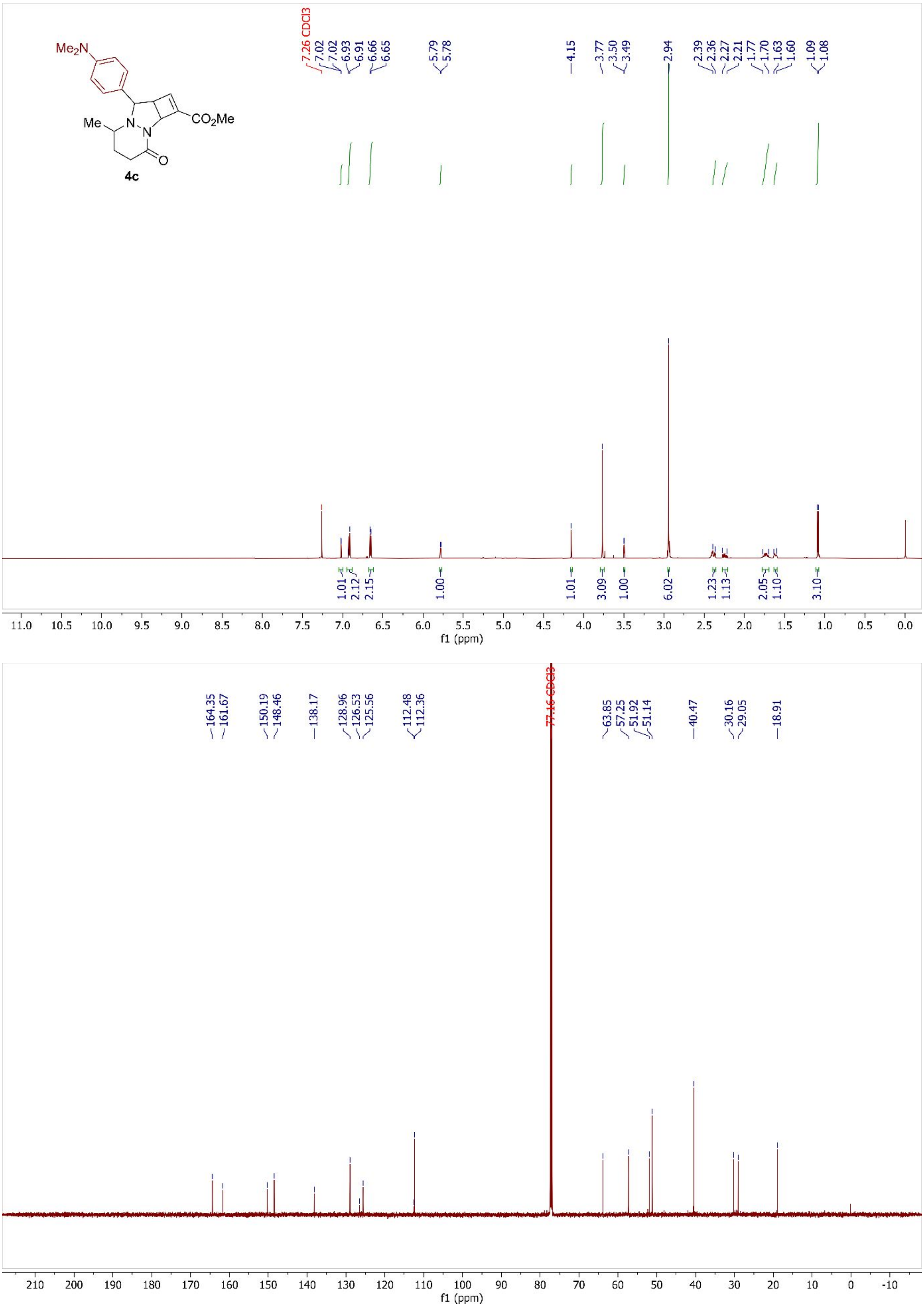

Figure S46. ^{1}H and ^{13}C{1H} NMR (600 MHz, $CDCl_3$) of compound **4c**.

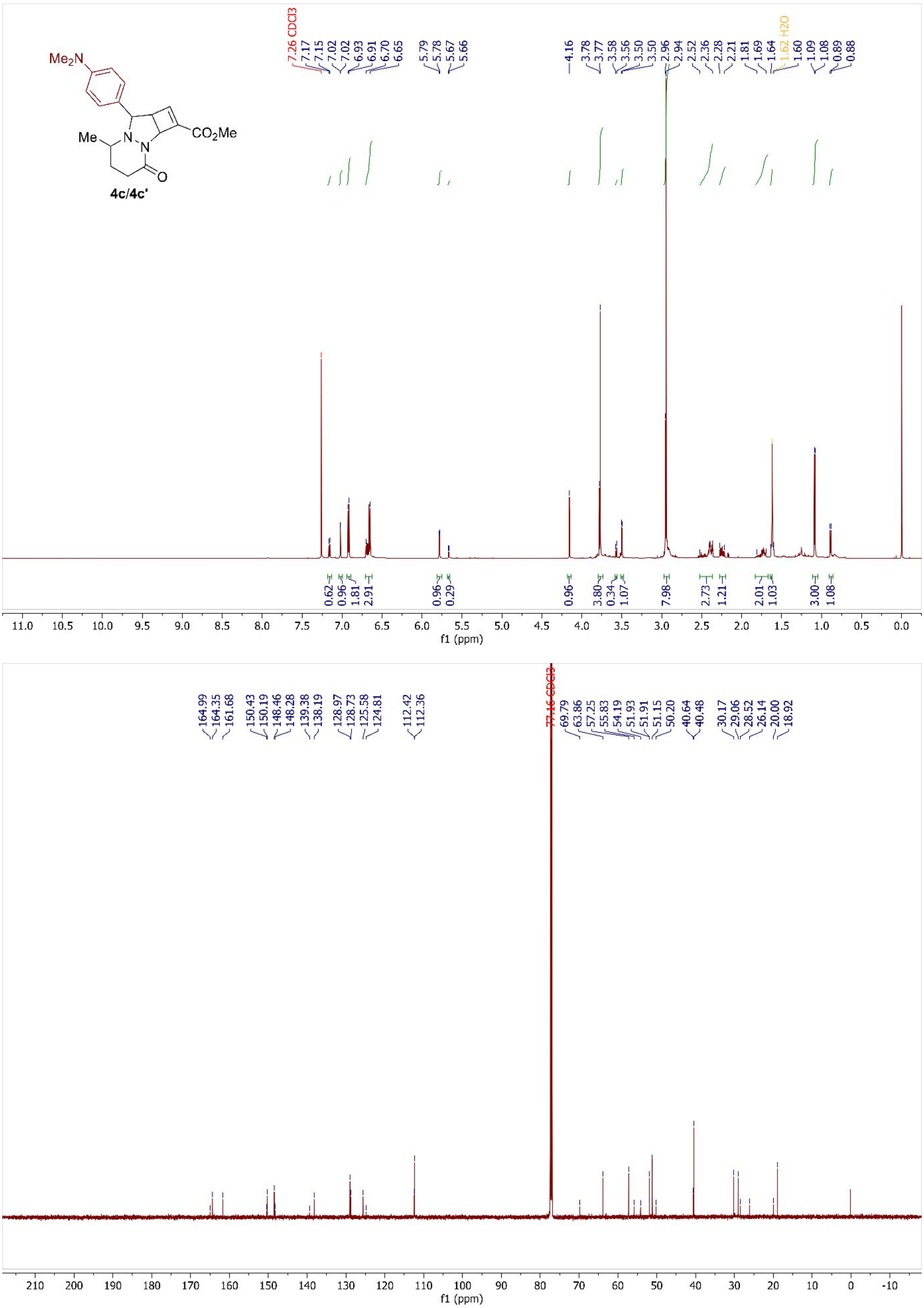

Figure S47. ^{1}H and $^{13}C\{1H\}$ NMR (600 MHz, $CDCl_3$) of compounds **4c**/**4c'**.

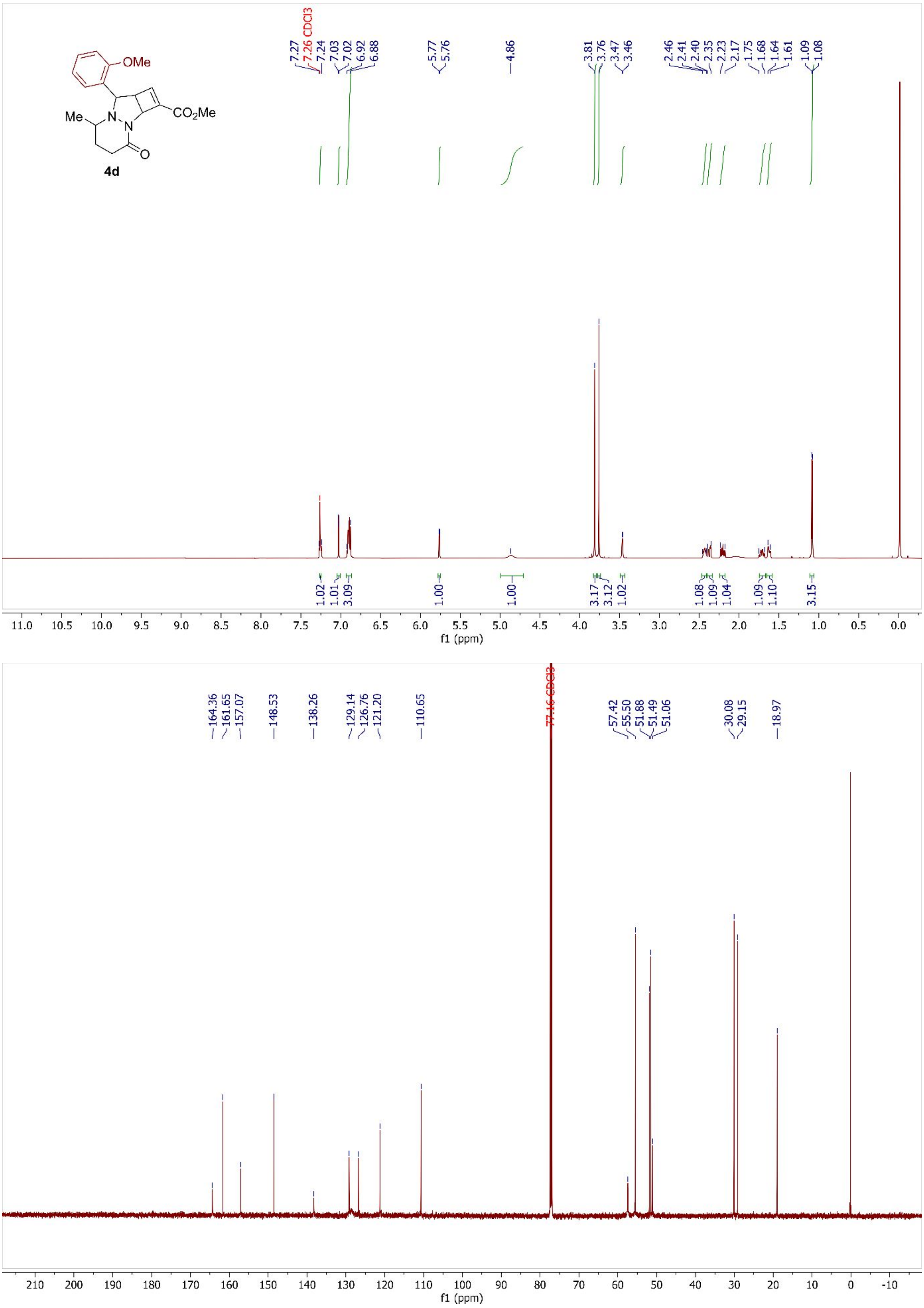

Figure S48. ^{1}H and ^{13}C{1H} NMR (600 MHz, $CDCl_3$) of compound **4d**.

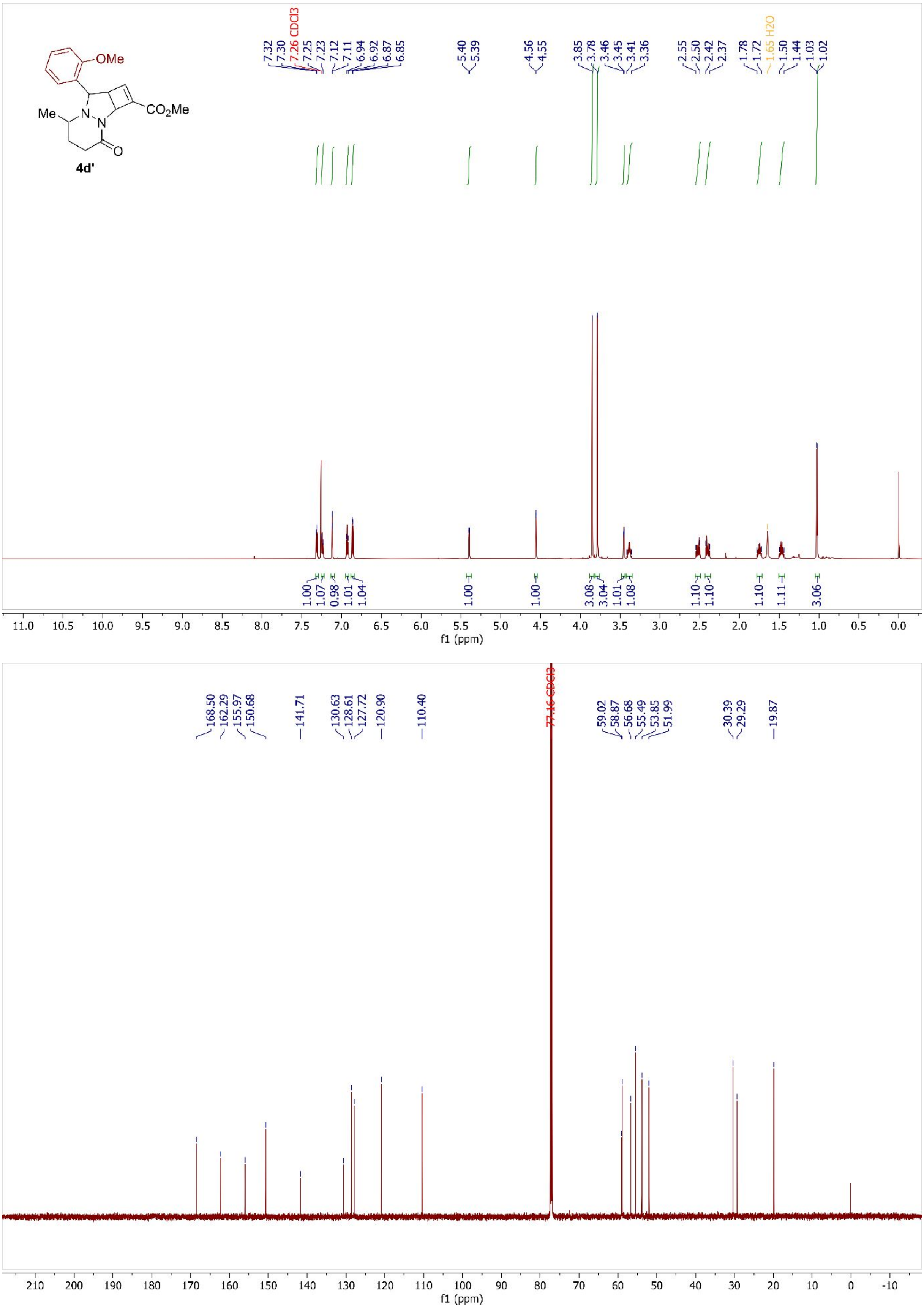

Figure S49. ^{1}H and $^{13}C\{1H\}$ NMR (600 MHz, $CDCl_3$) of compound **4d’**.

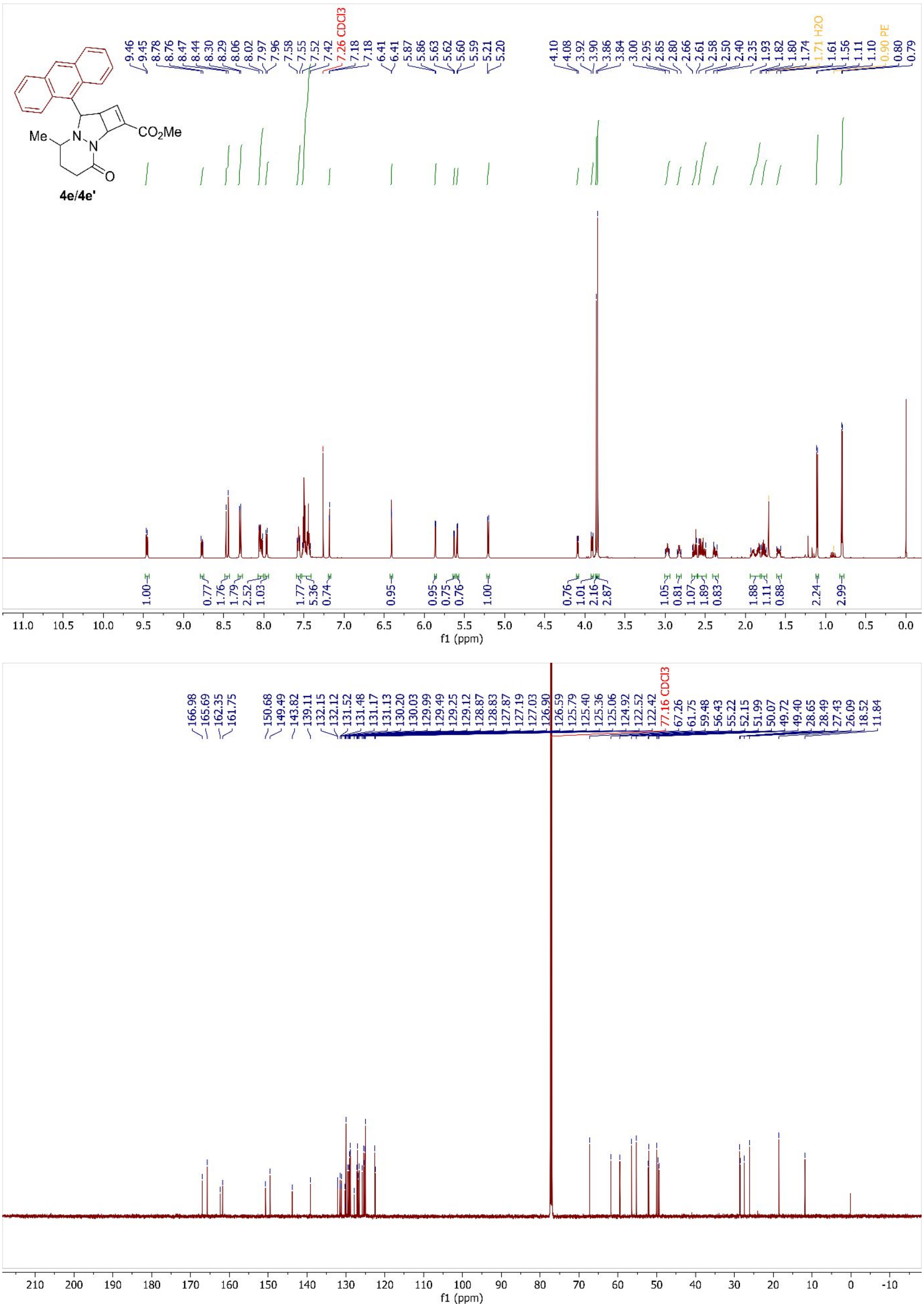

Figure S50. ^{1}H and $^{13}C\{1H\}$ NMR (600 MHz, $CDCl_3$) of compounds **4e**/**4e'**.

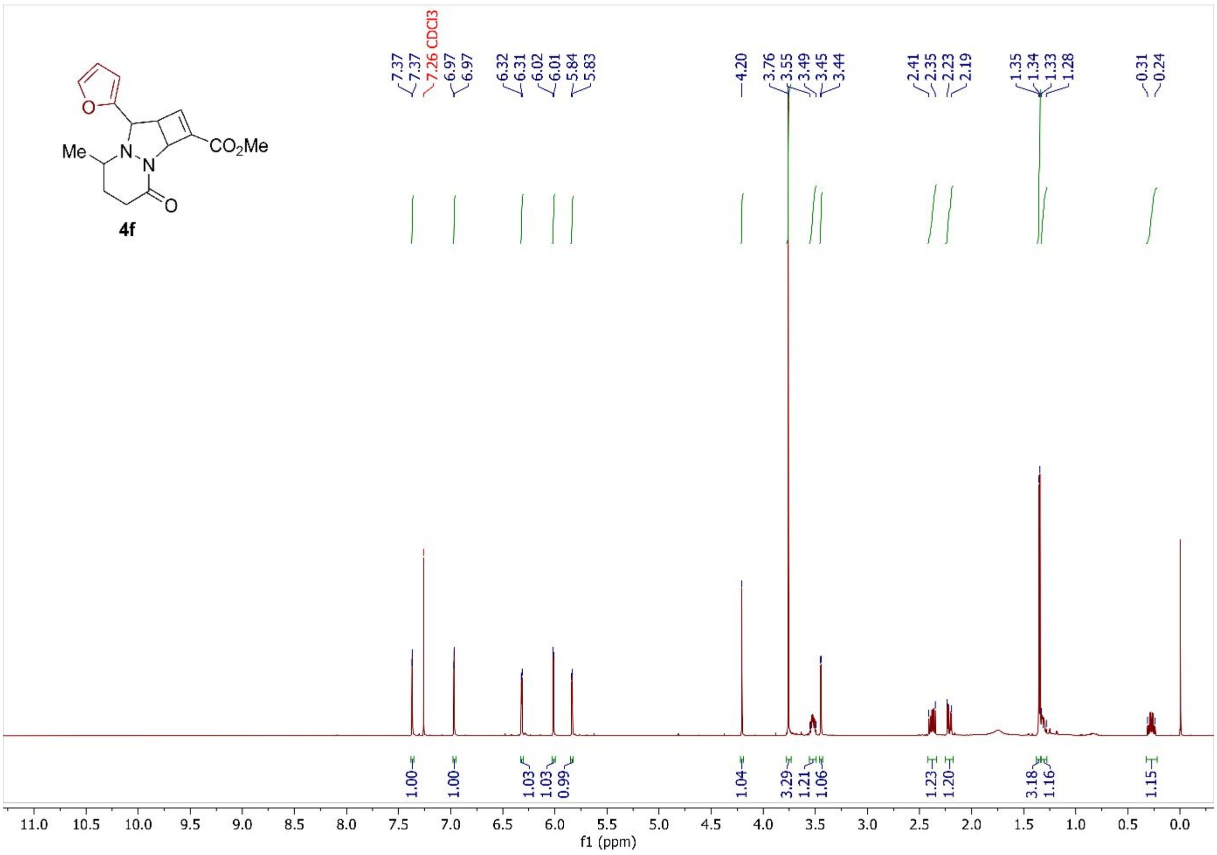

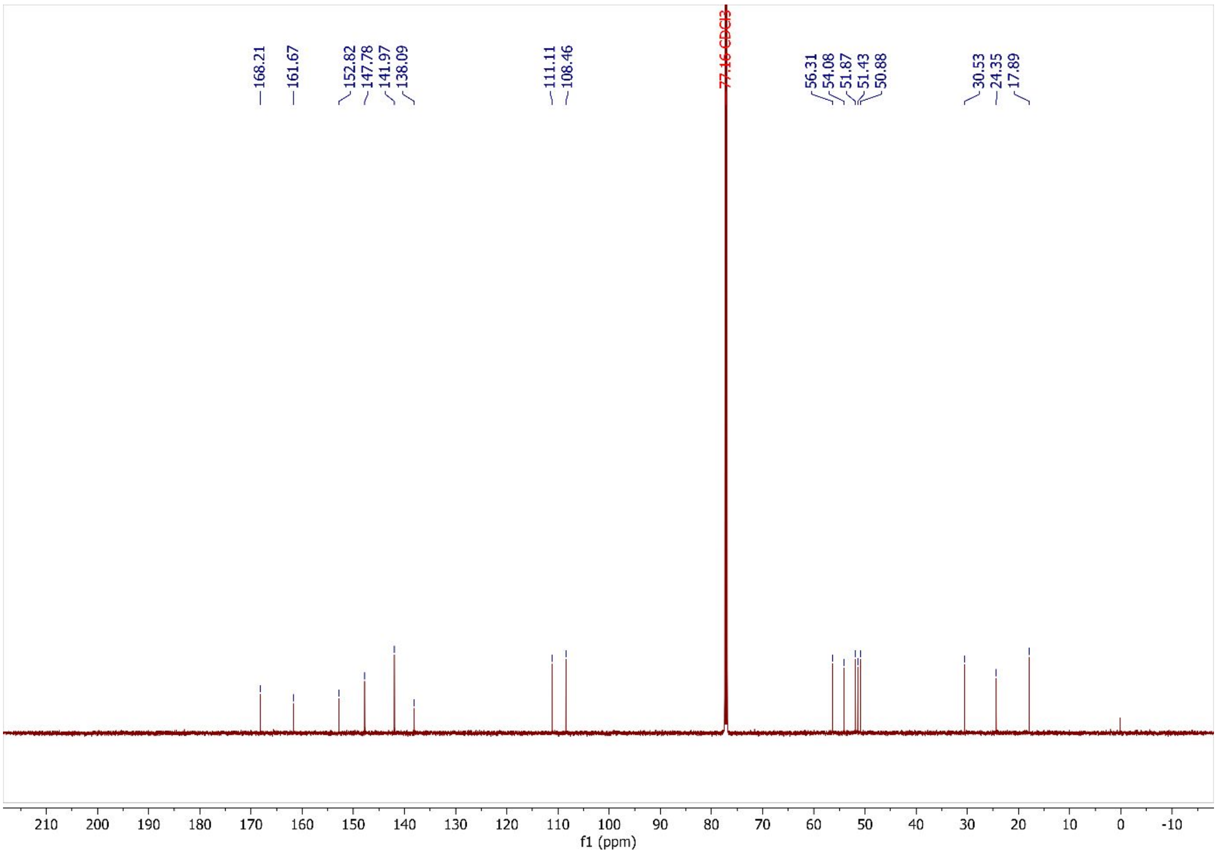

Figure S51. ^{1}H and $^{13}C\{1H\}$ NMR (600 MHz, $CDCl_3$) of compound **4f**.

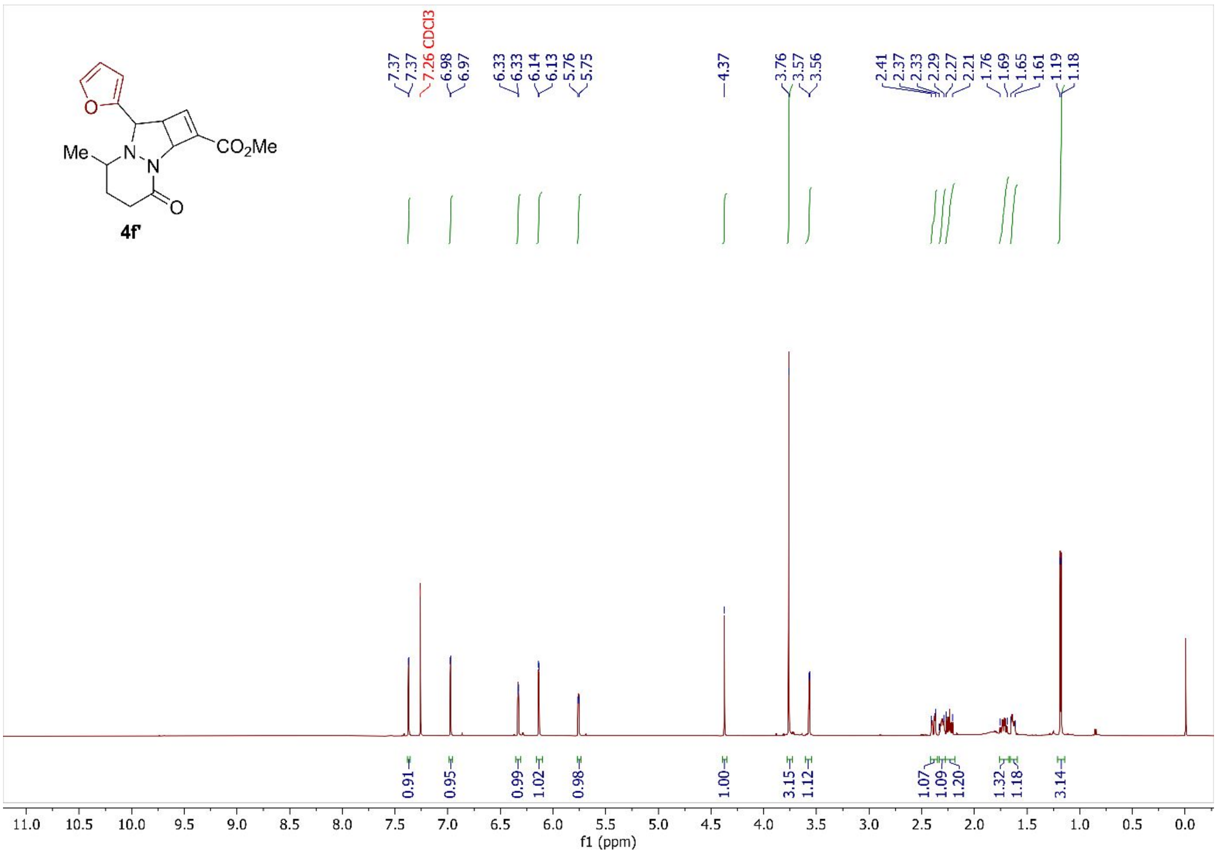

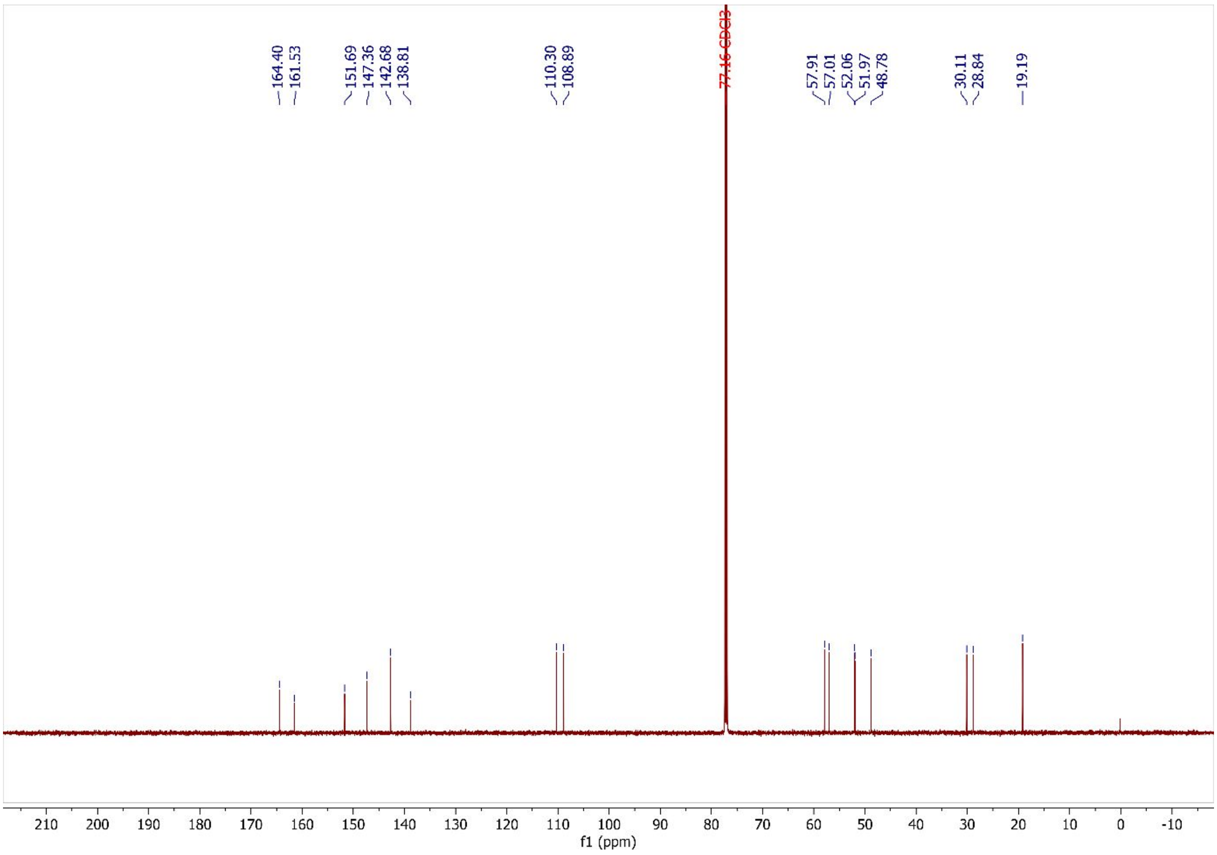

Figure S52. ^{1}H and $^{13}C\{1H\}$ NMR (600 MHz, $CDCl_3$) of compound **4f'**.

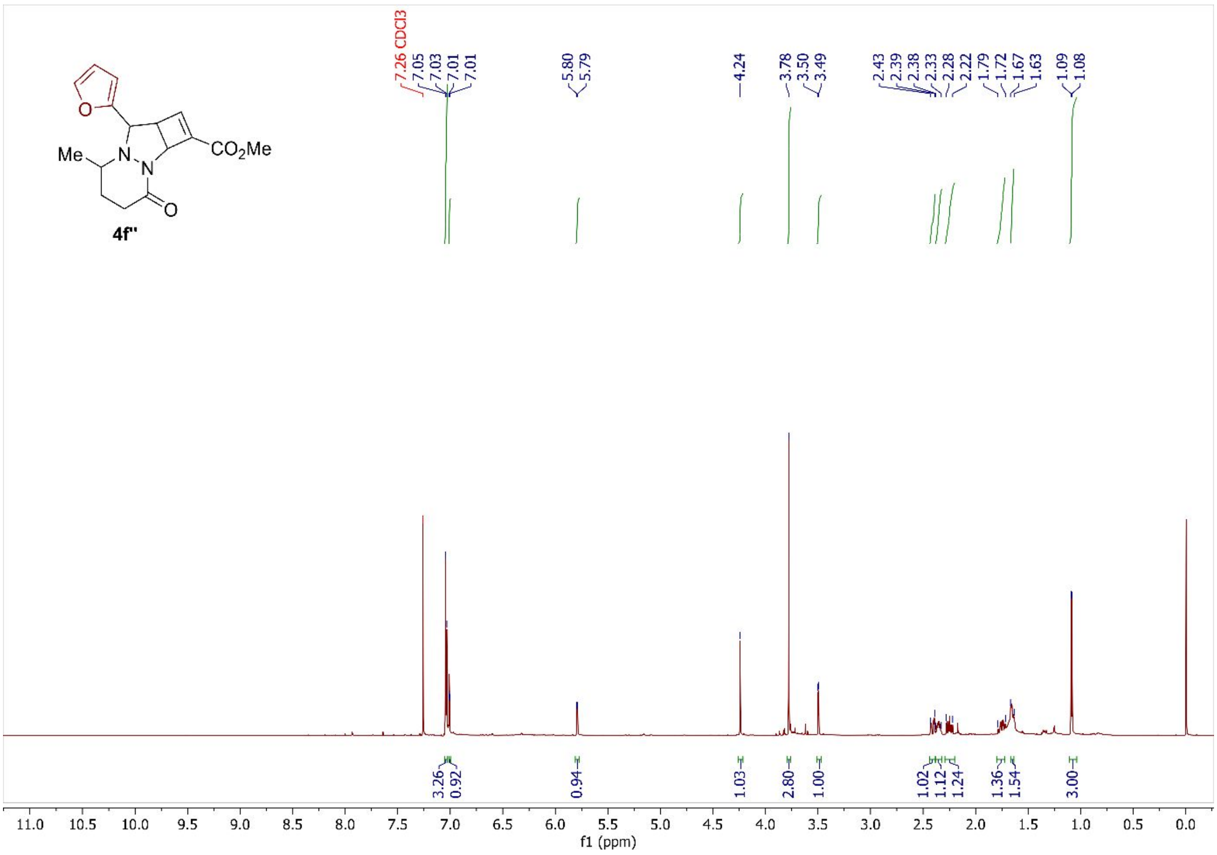

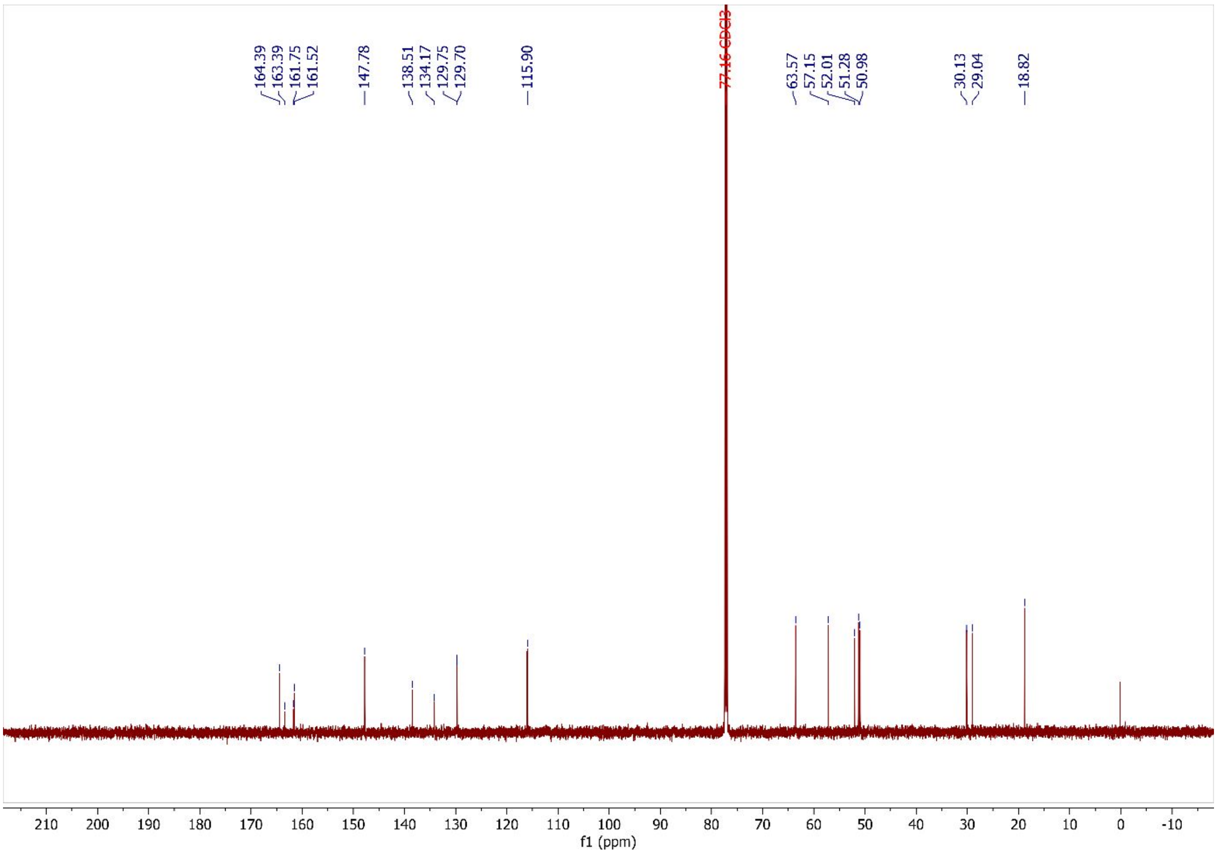

Figure S53. ^{1}H and $^{13}C\{1H\}$ NMR (600 MHz, $CDCl_3$) of compound **4f''**.

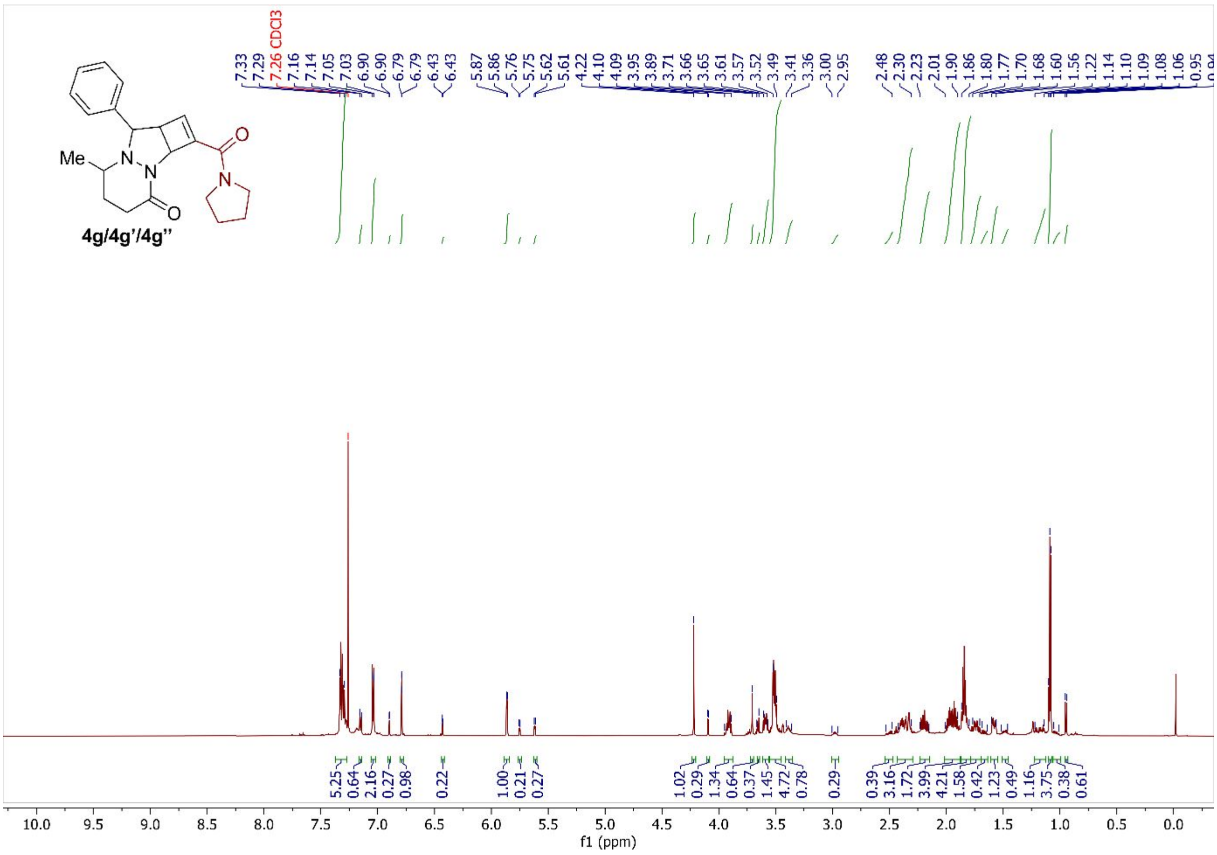

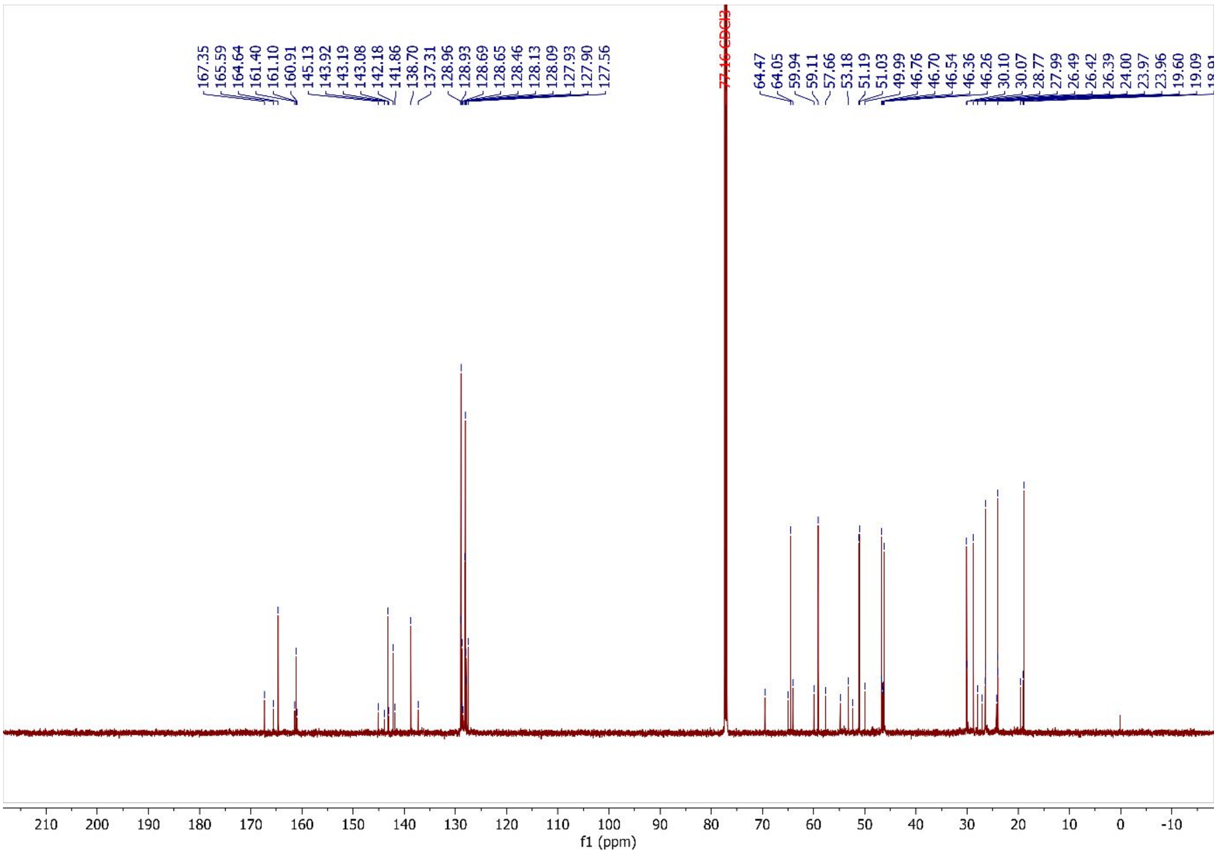

Figure S54. ^{1}H and ^{13}C{1H} NMR (600 MHz, $CDCl_3$) of compounds **4**g/4g'/4g''.

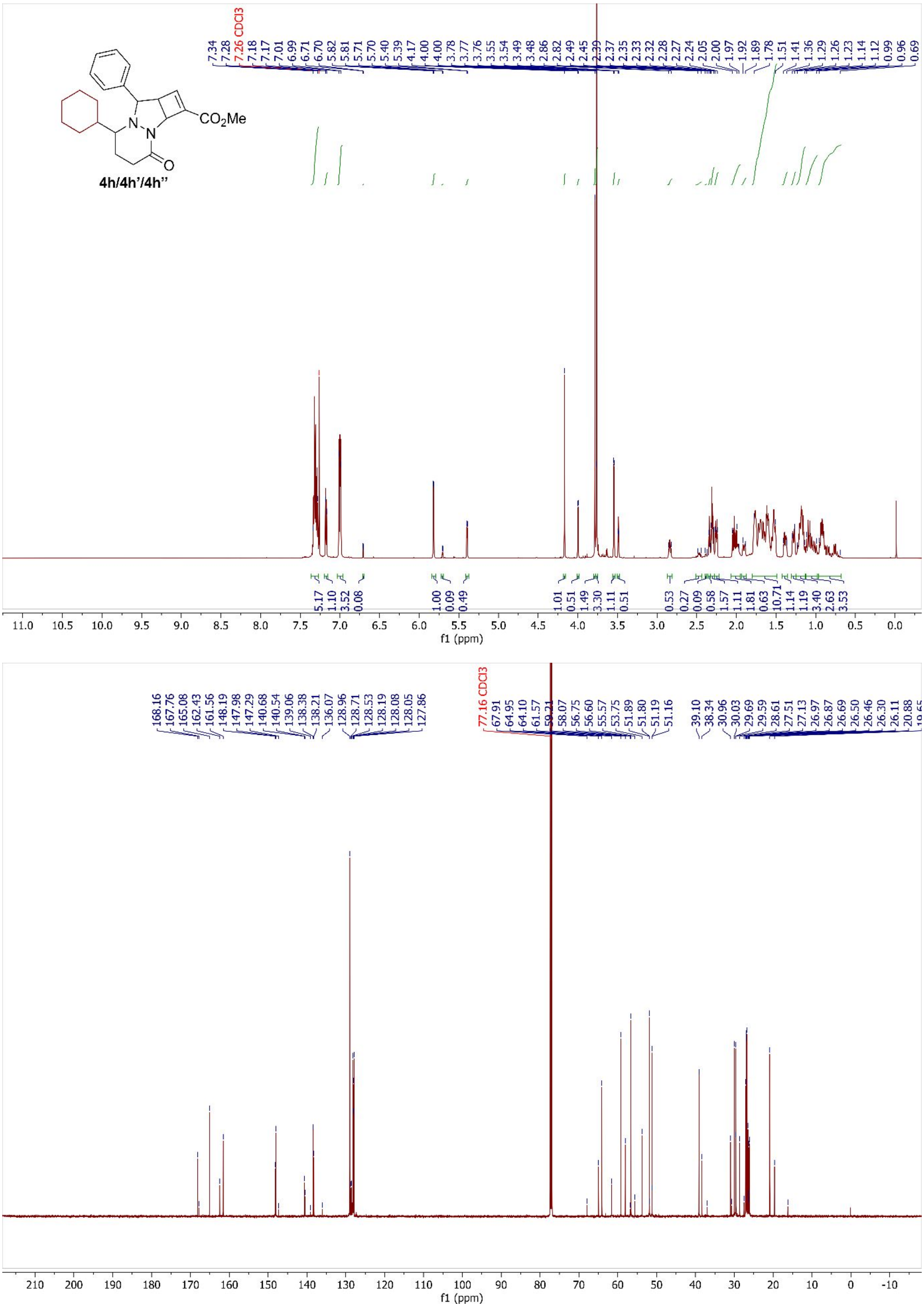

Figure S55. ^{1}H and $^{13}C\{1H\}$ NMR (600 MHz, $CDCl_3$) of compounds **4h**/**4h'**/**4h''**.

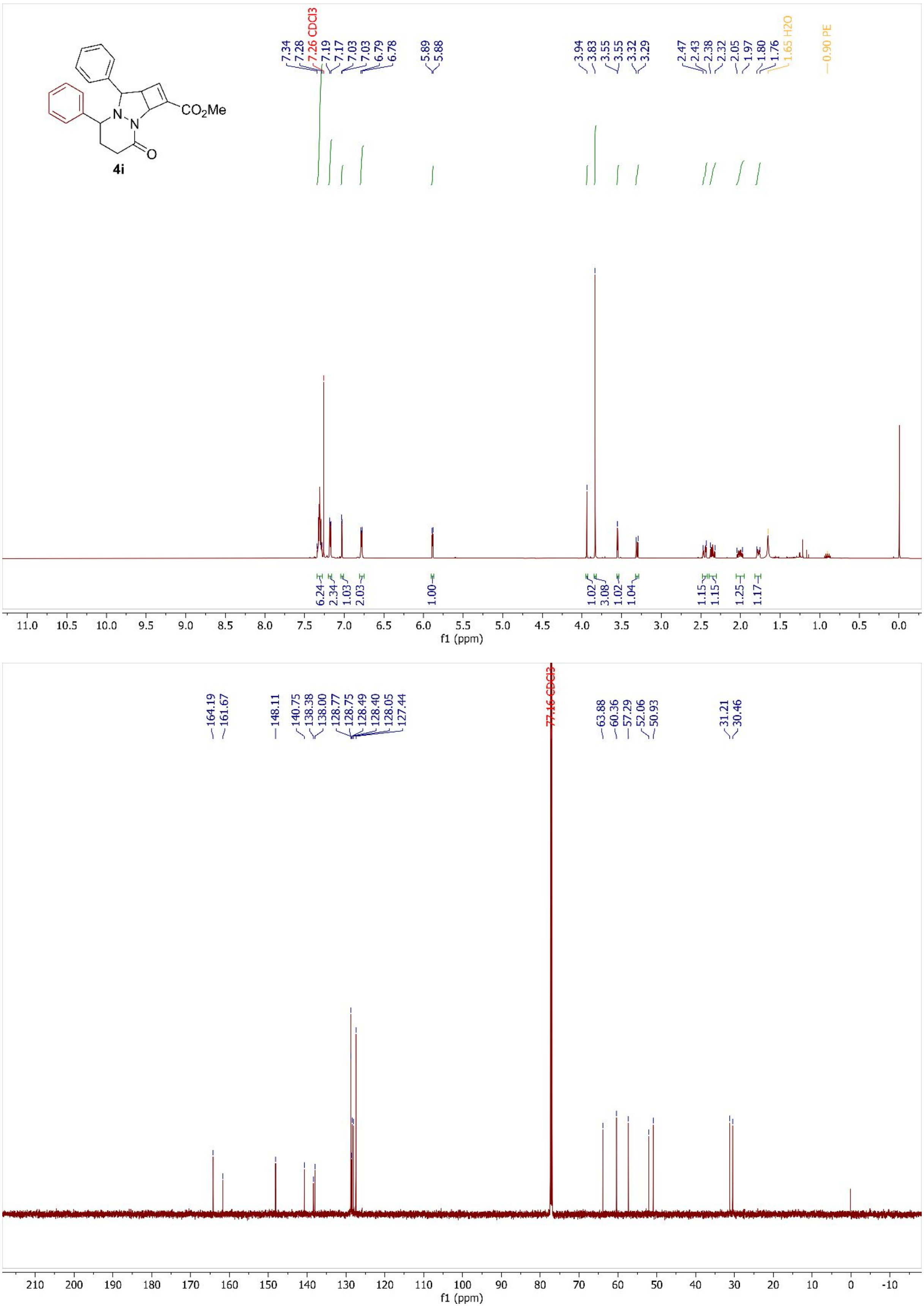

Figure S56. ^{1}H and ^{13}C{1H} NMR (600 MHz, $CDCl_3$) of compound **4i**.

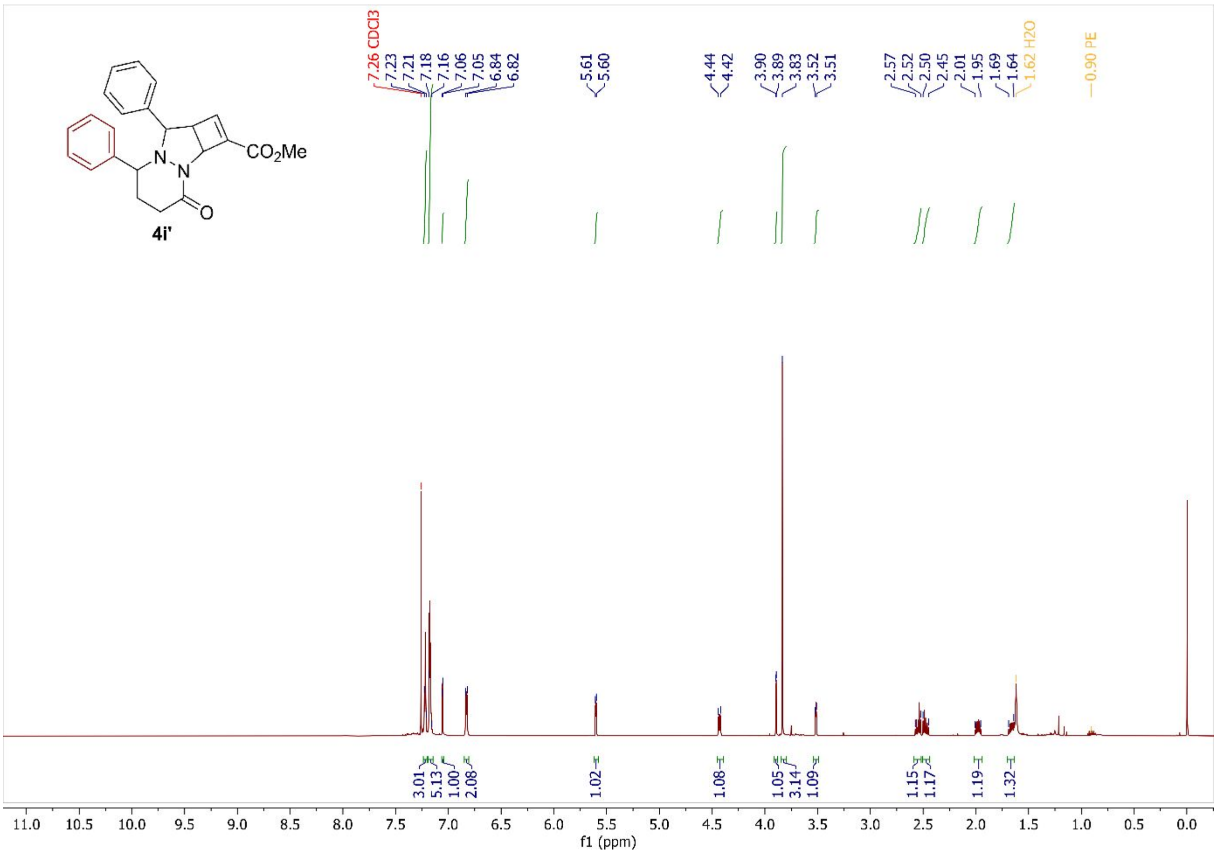

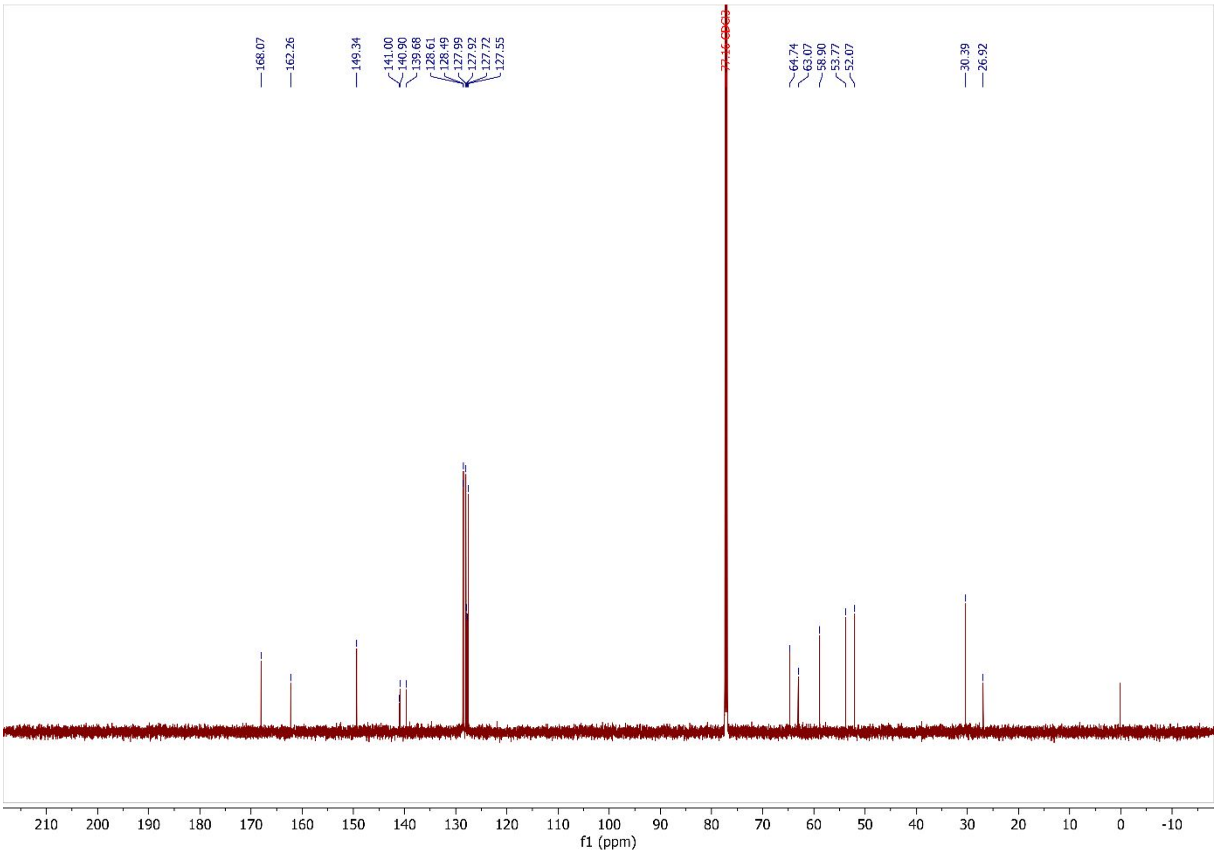

Figure S57. ^1H and $^{13}C\{1H\}$ NMR (600 MHz, $CDCl_3$) of compound **4i'**.

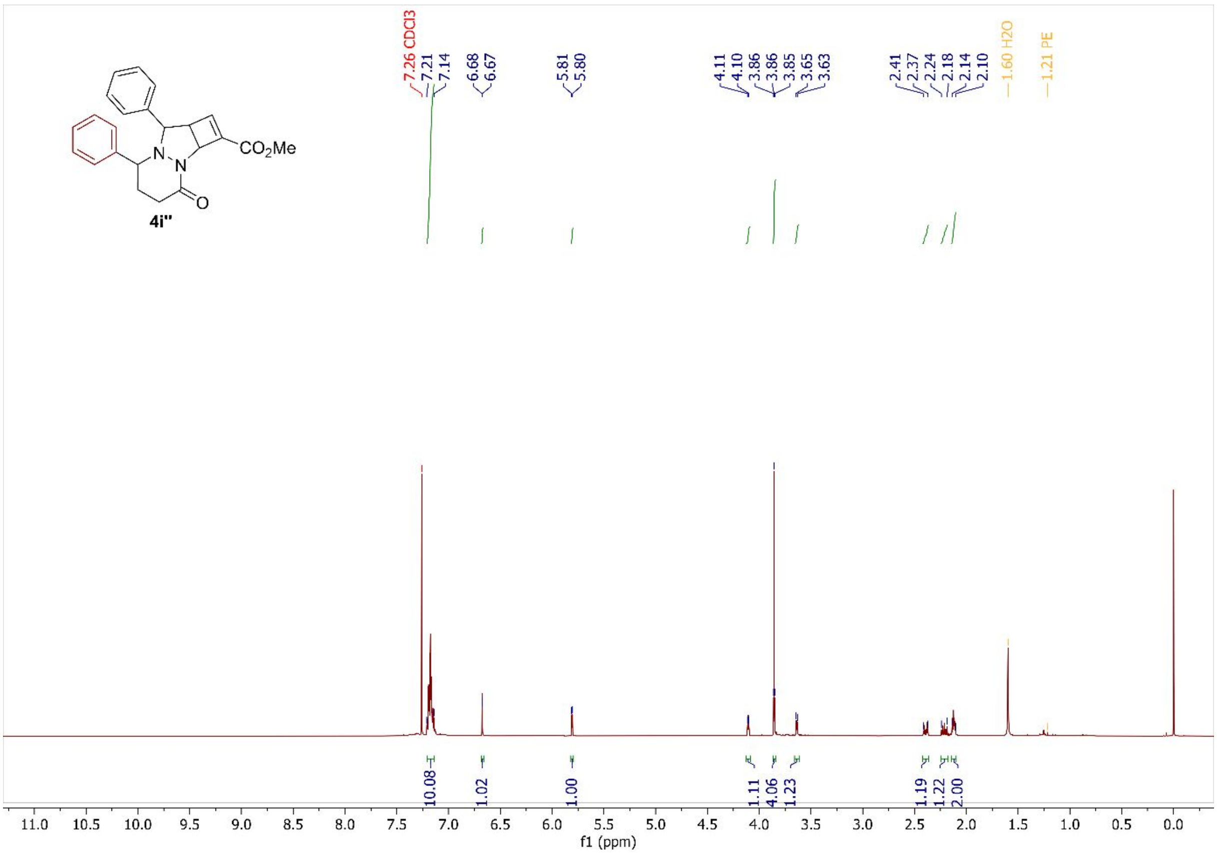

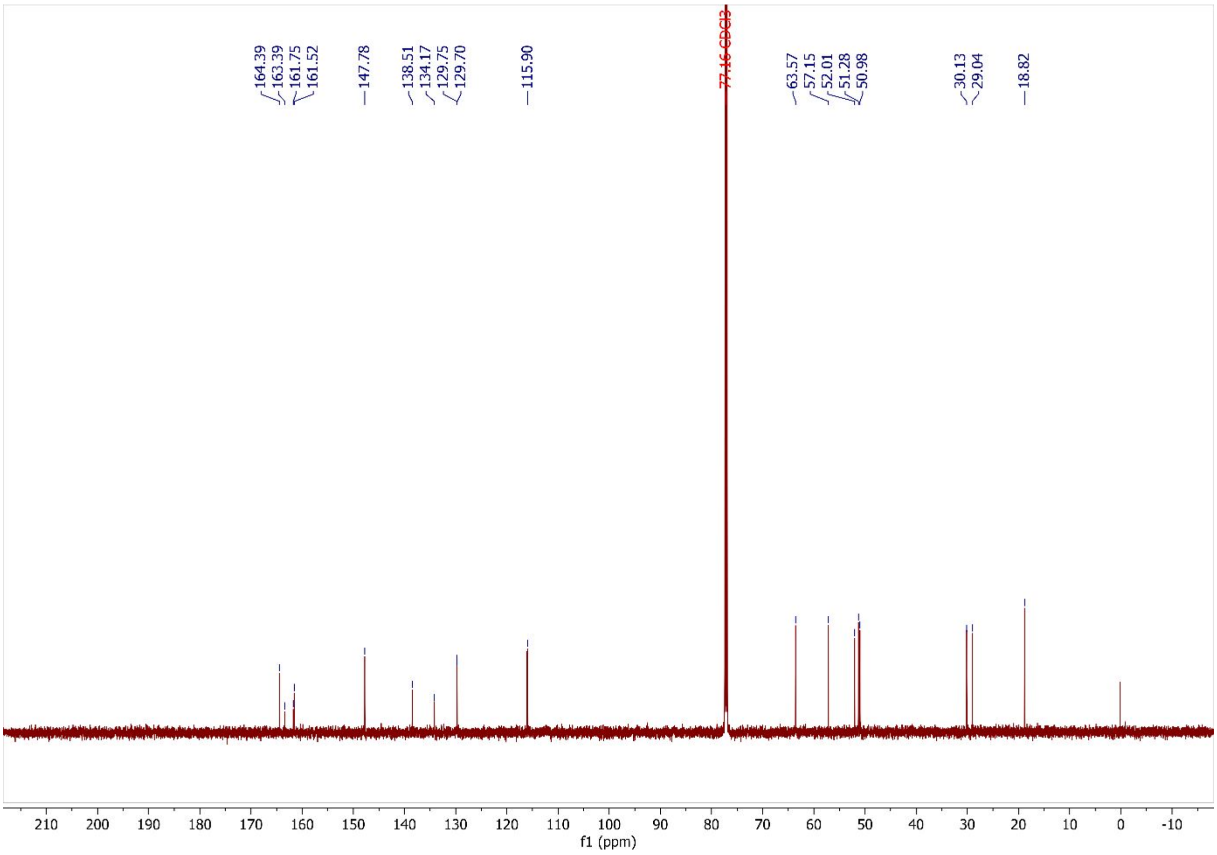

Figure S58. ^{1}H and $^{13}C\{^{1}H\}$ NMR (600 MHz, $CDCl_3$) of compound **4i''**.

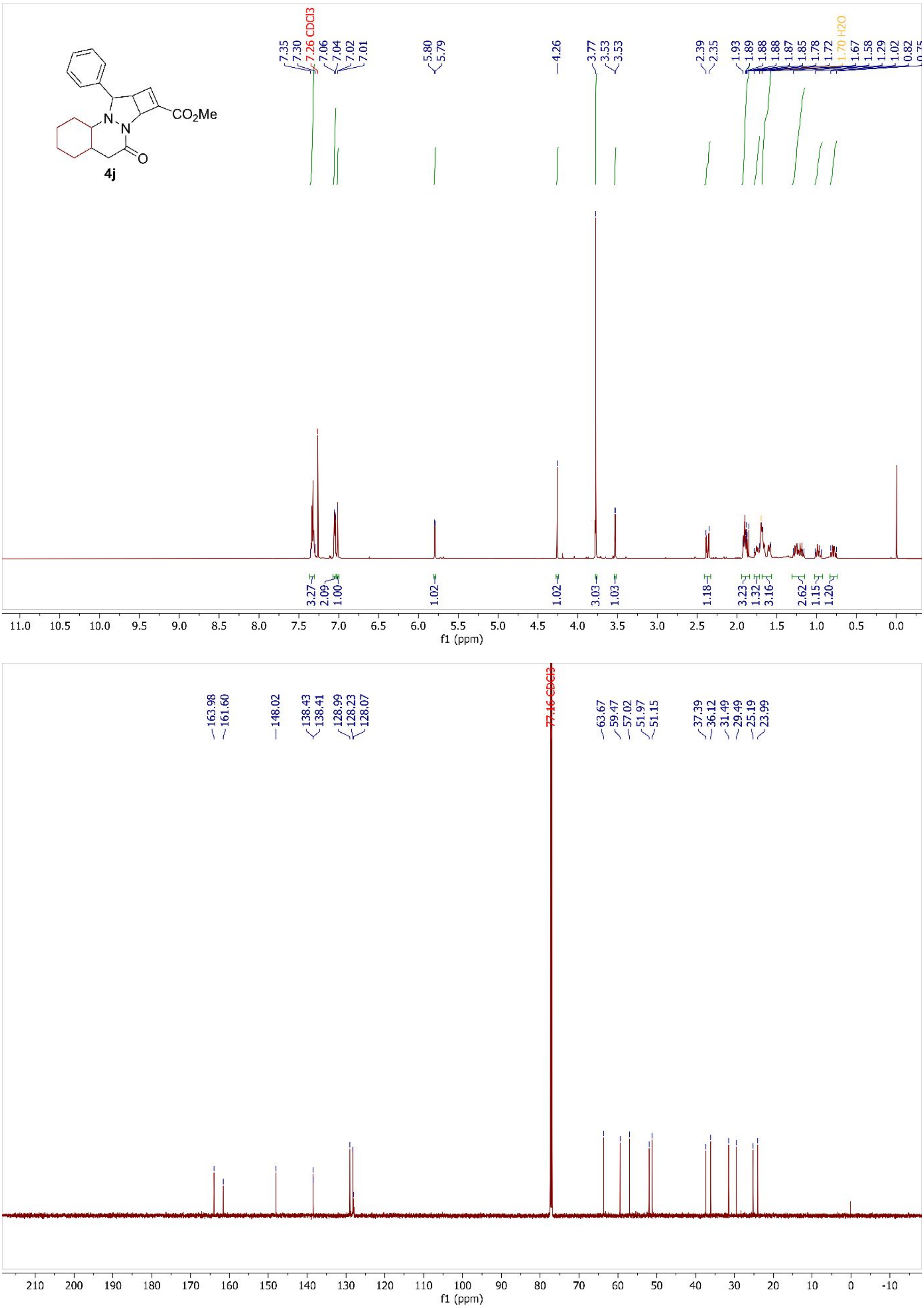

Figure S59. ^{1}H and $^{13}C\{1H\}$ NMR (600 MHz, $CDCl_3$) of compound **4j**.

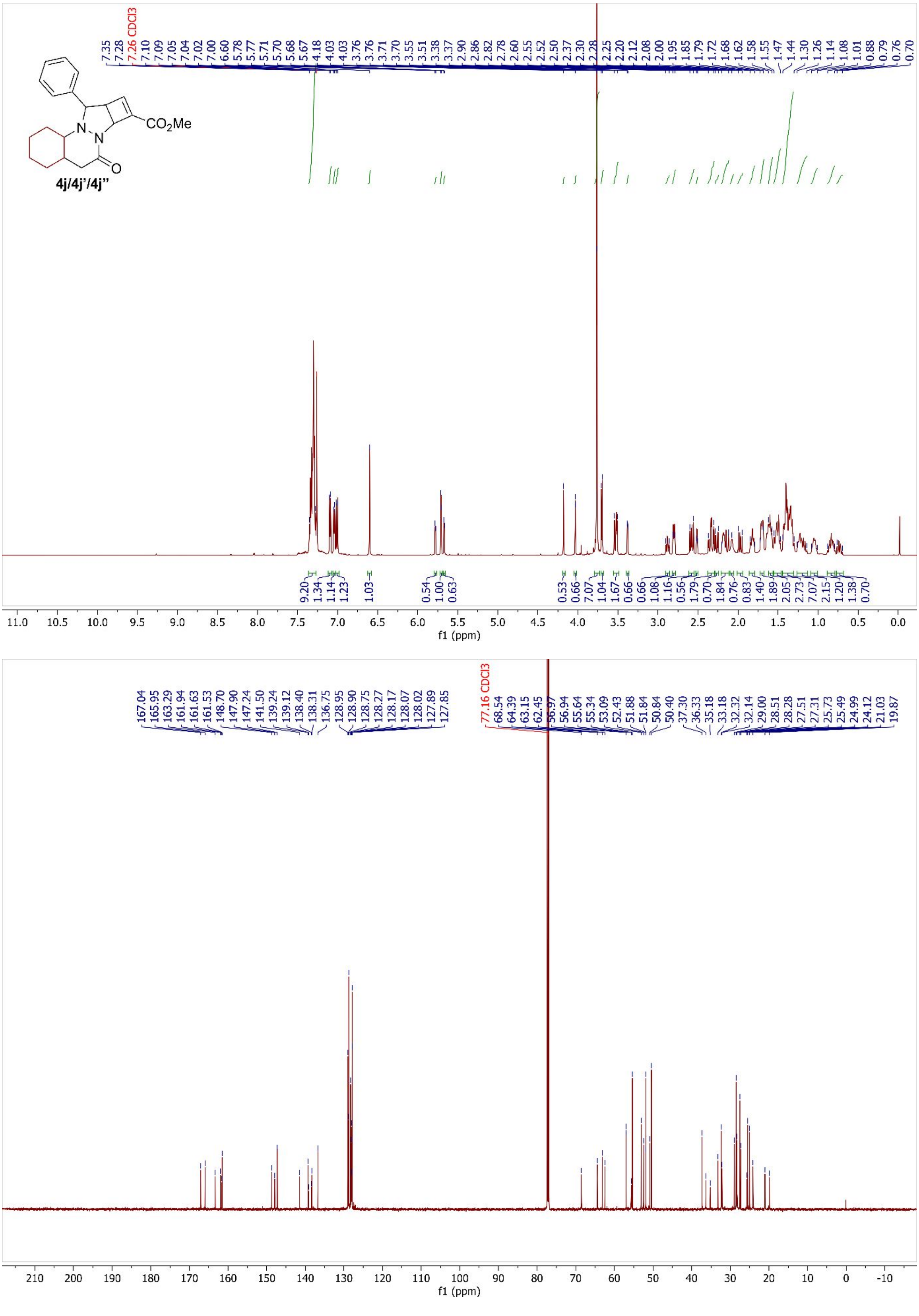

Figure S60. ¹H and $^{13}C\{1H\}$ NMR (600 MHz, $CDCl_3$) of compounds **4j/4j'/4j''**.

17.6 Products *E*-**2-OH** and *Z*-**2-OH**

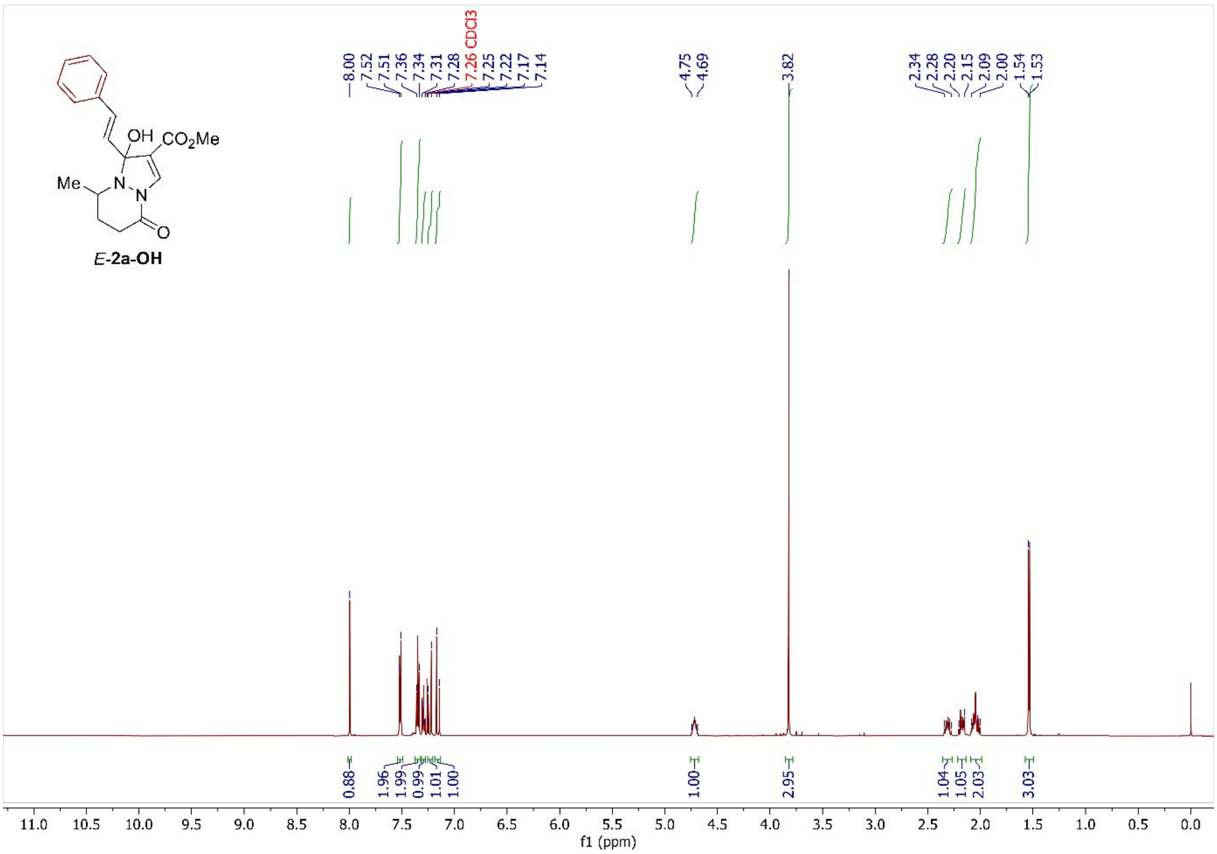

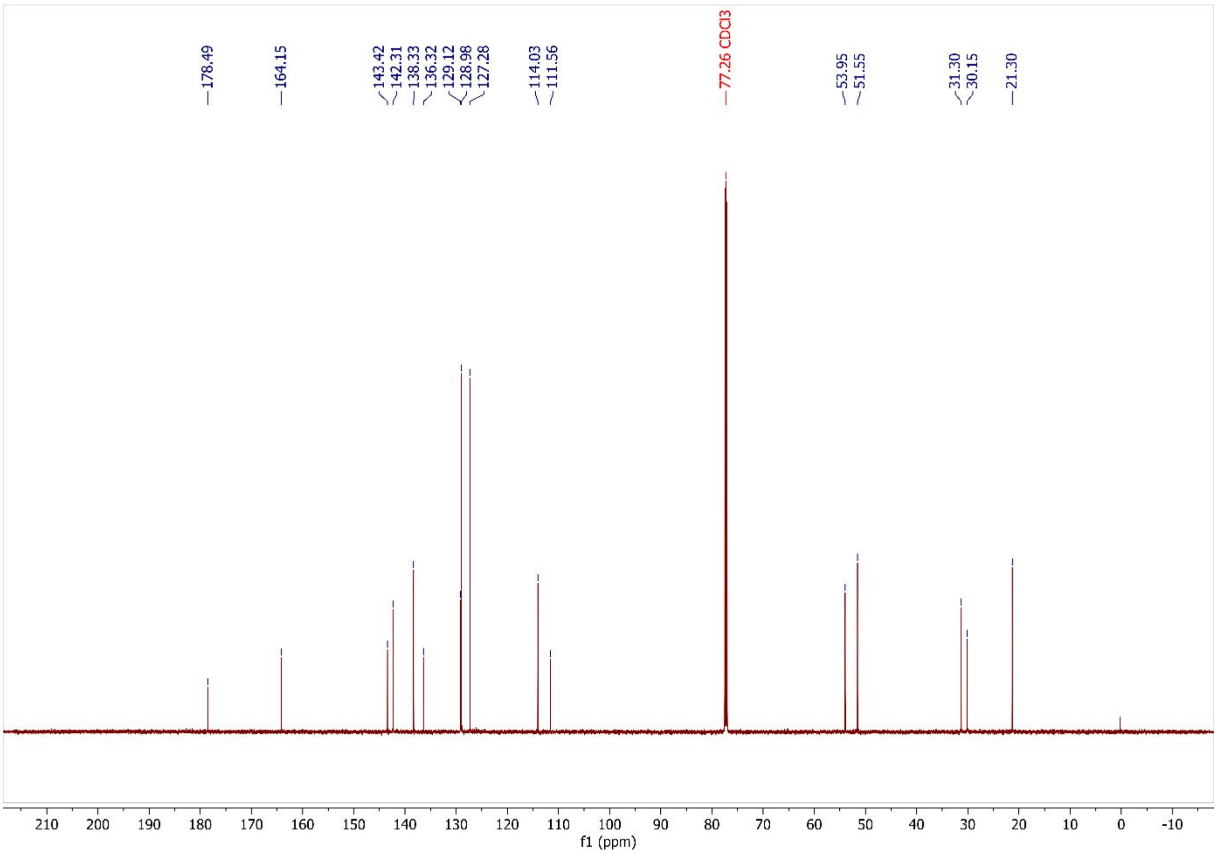

Figure S61. ^{1}H and ^{13}C{1H} NMR (600 MHz, $CDCl_3$) of compound *E*-**2a-OH**.

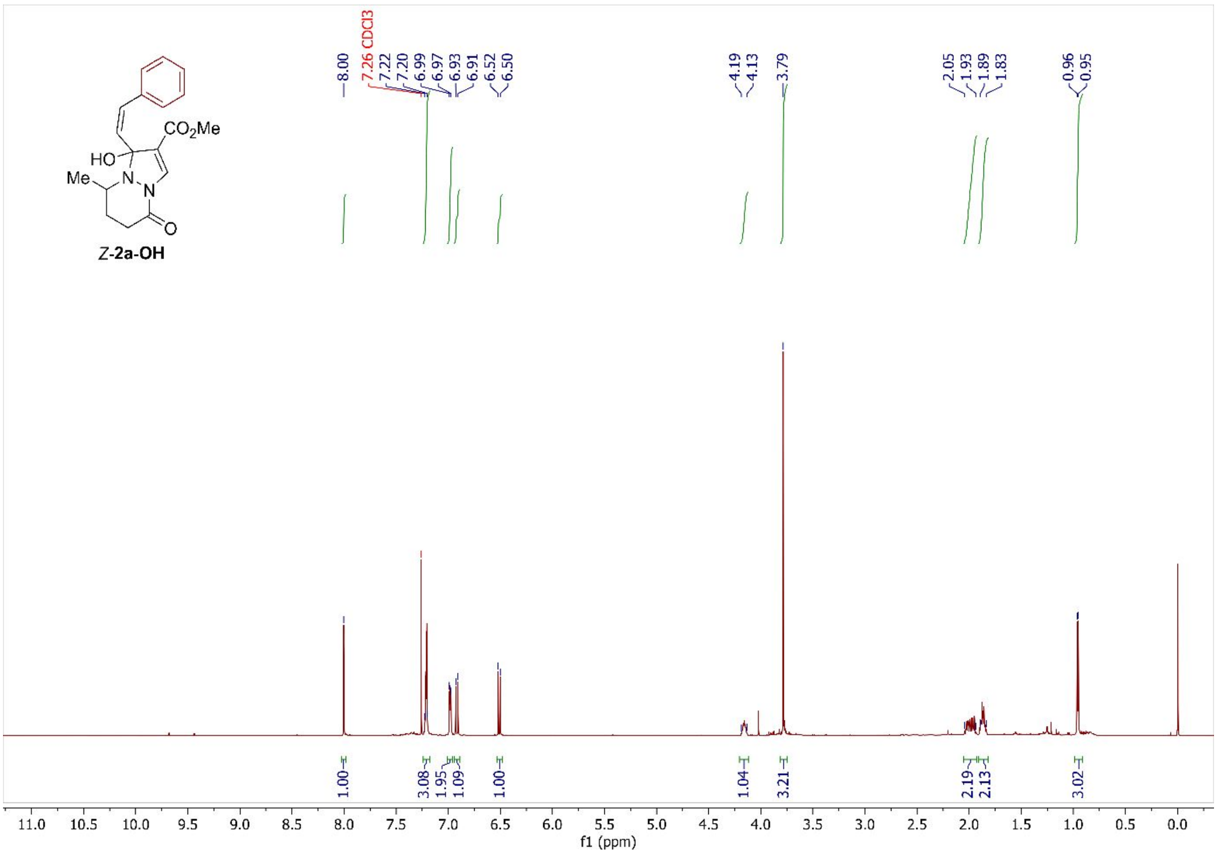

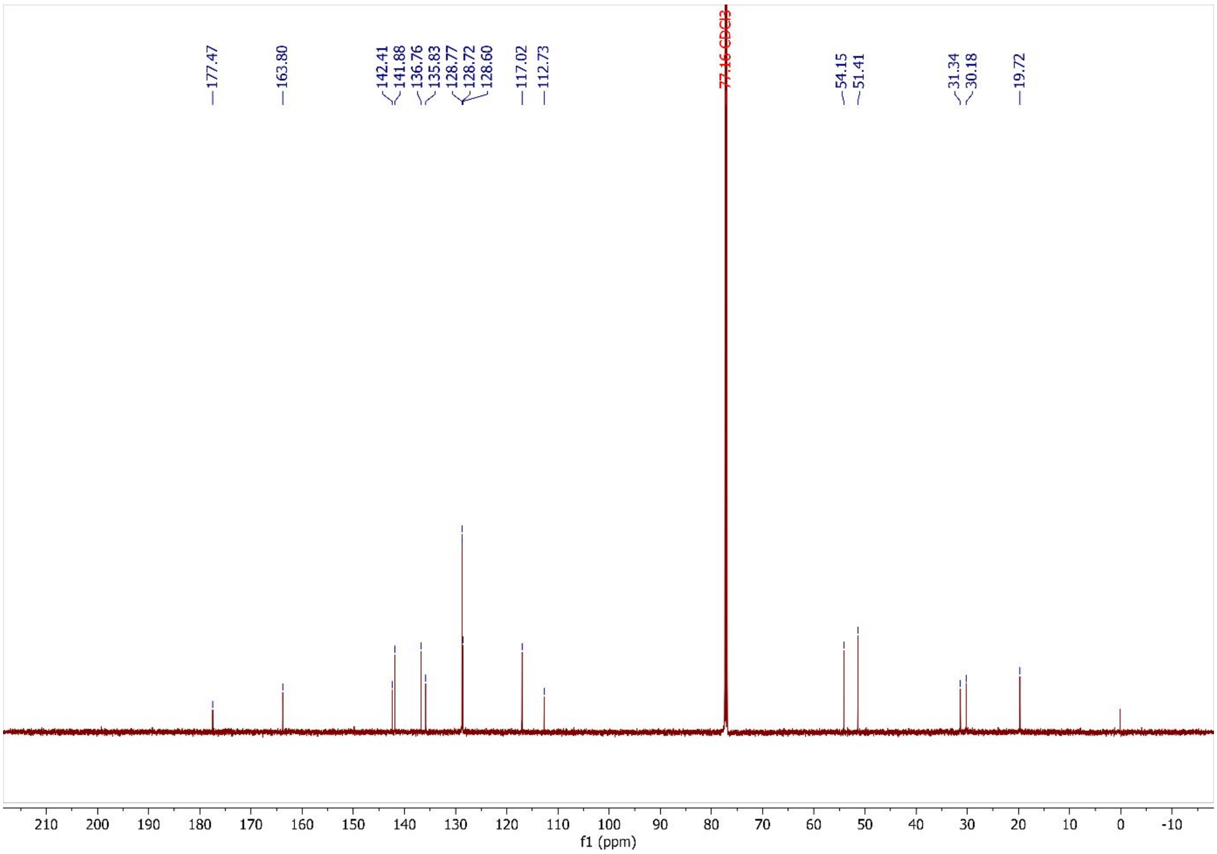

Figure S62. ^{1}H and ^{13}C{1H} NMR (600 MHz, $CDCl_3$) of compound *Z*-**2a-OH**.

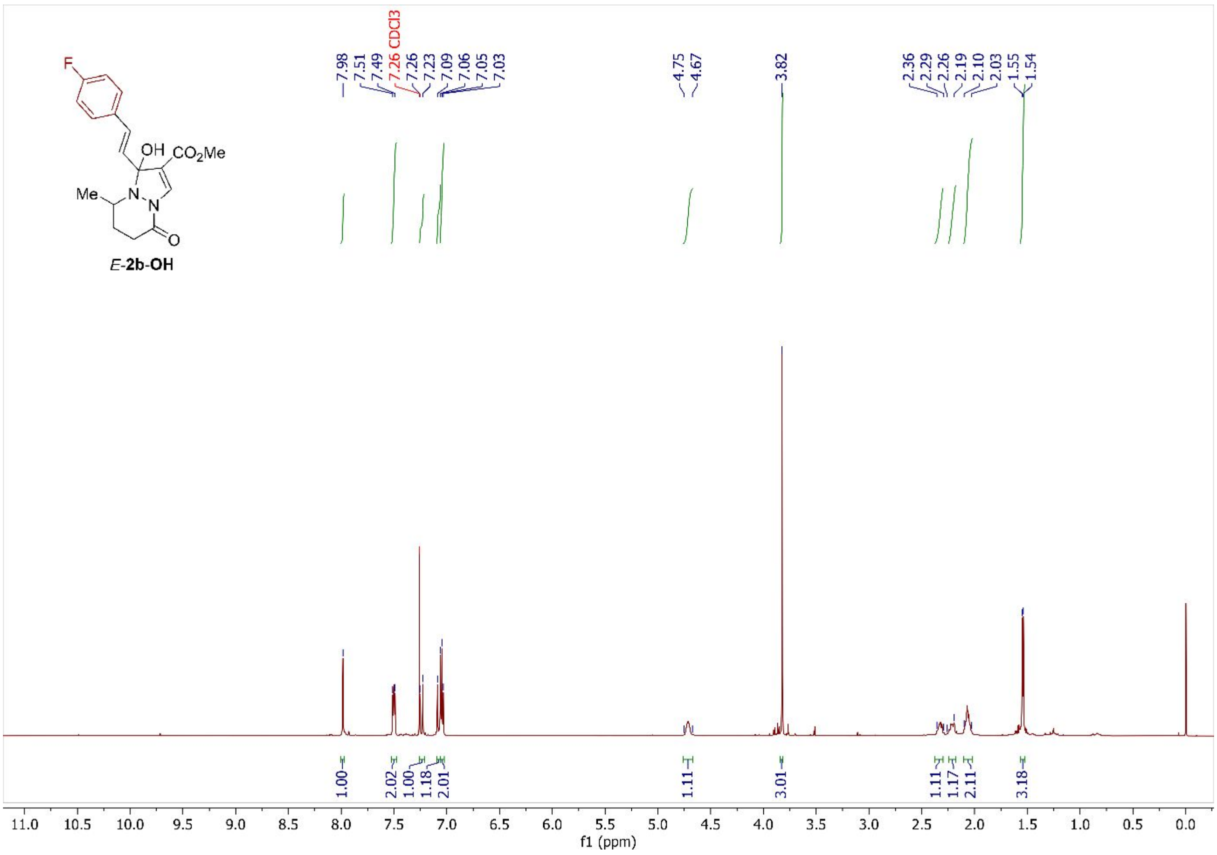

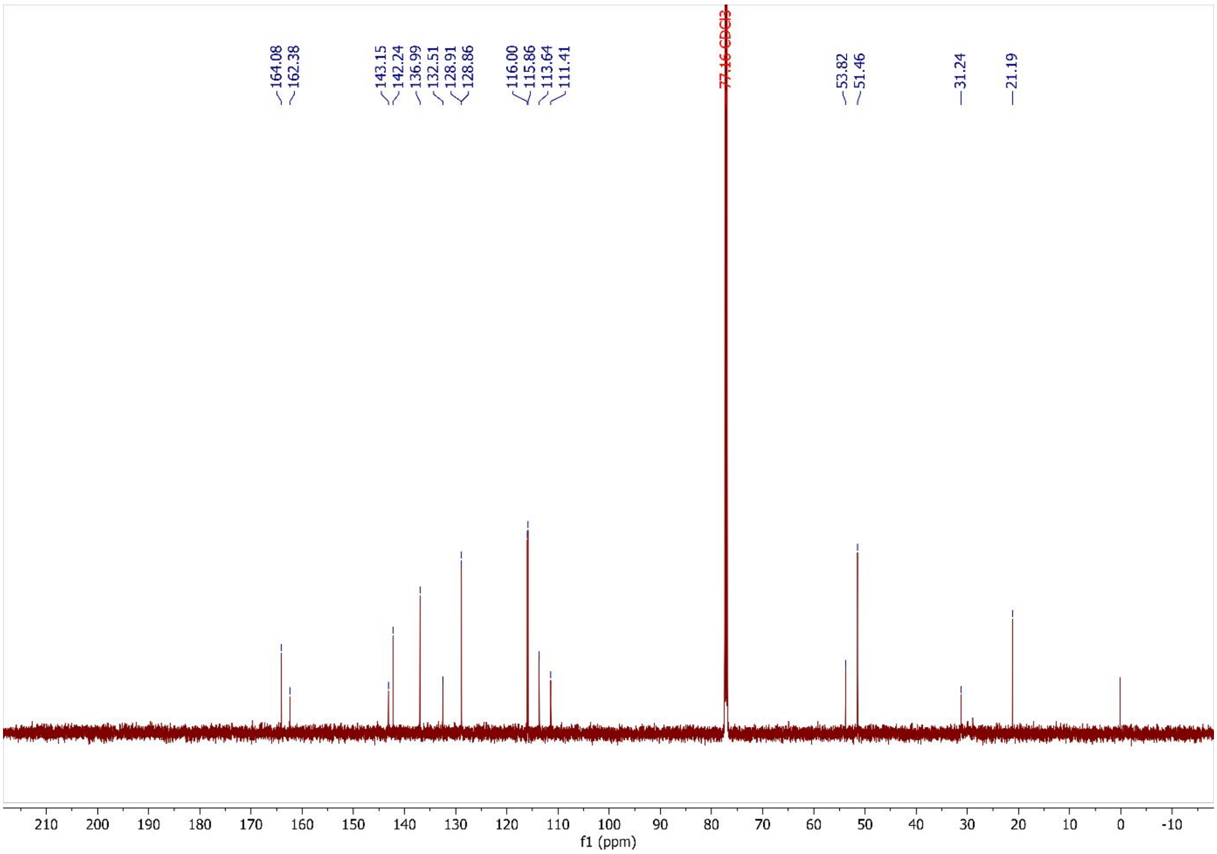

Figure S63. ^{1}H and ^{13}C{1H} NMR (600 MHz, $CDCl_3$) of compound *E*-**2b-OH**.

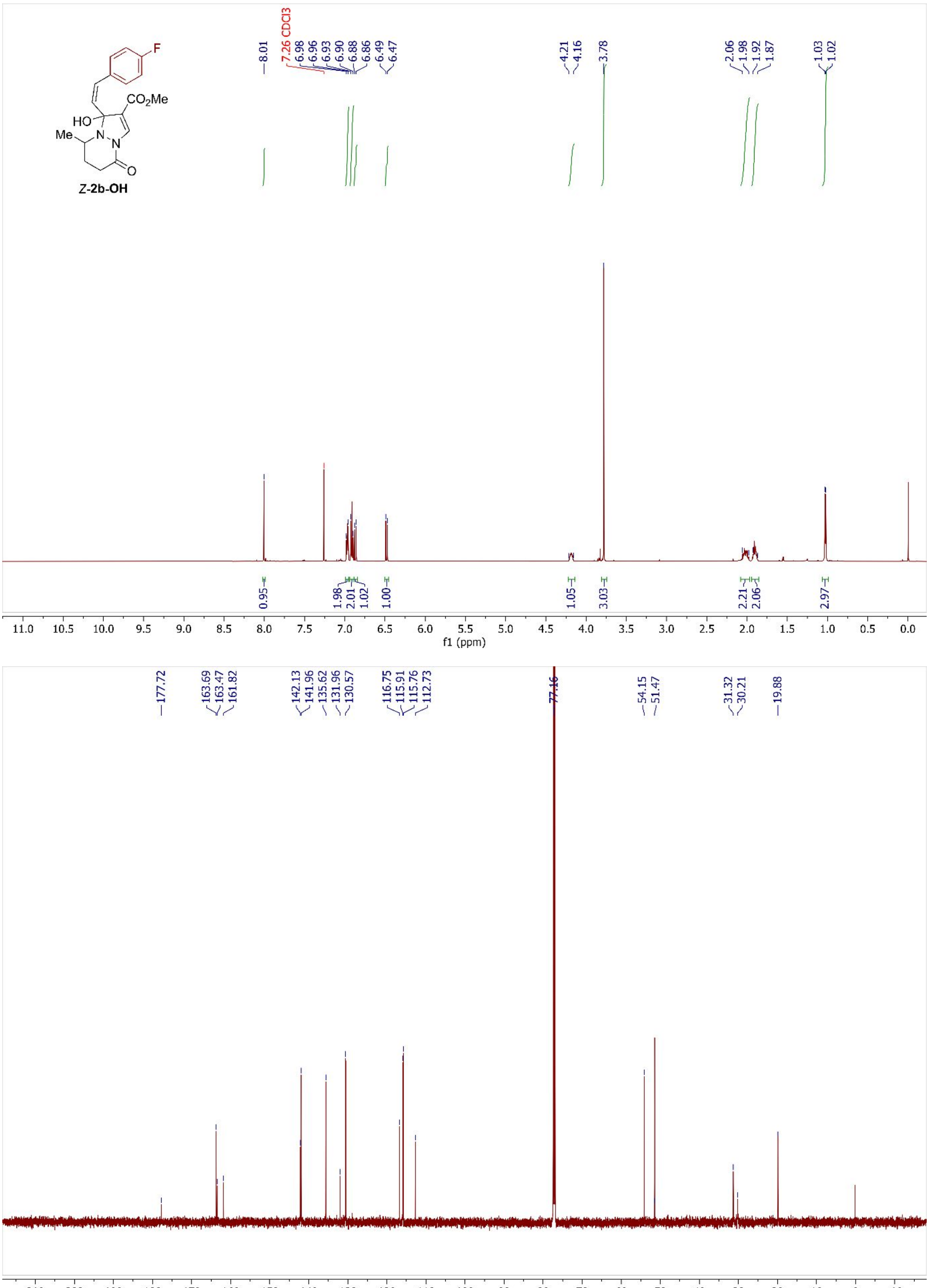

Figure S64. ^{1}H and $^{13}C\{1H\}$ NMR (600 MHz, $CDCl_3$) of compound *Z*-**2b-OH**.

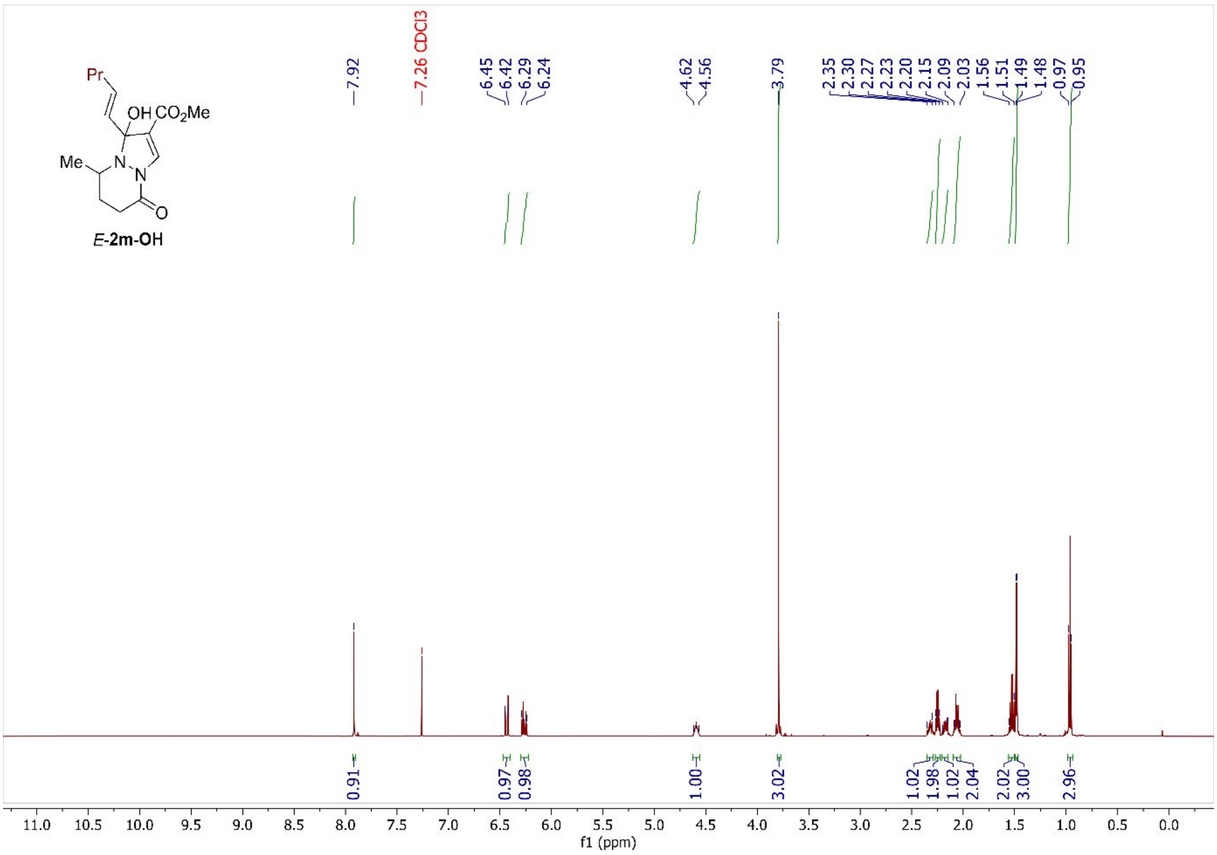

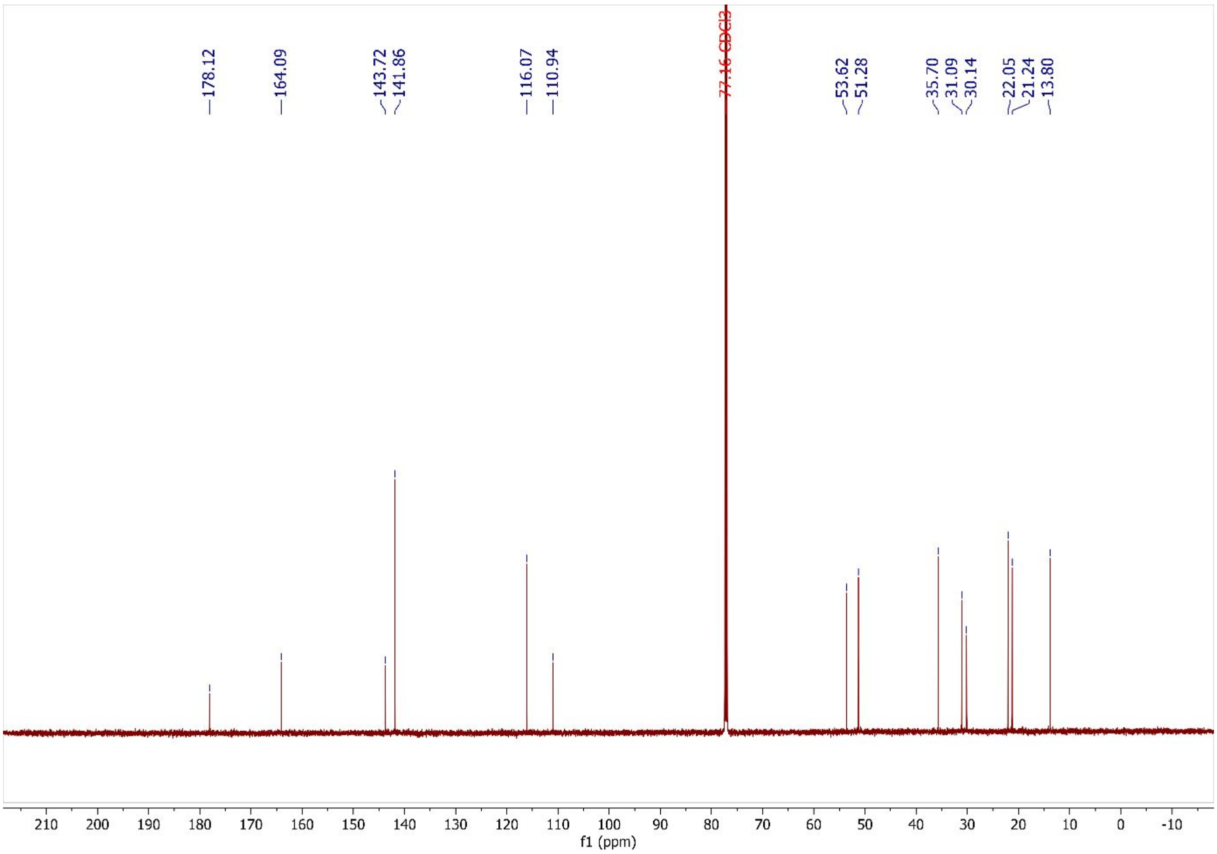

Figure S65. ^{1}H and $^{13}C\{1H\}$ NMR (600 MHz, $CDCl_3$) of compound *E*-**2m-OH**.

17.7 Pyrazolo[1,2-*a*]pyridazinones **5**

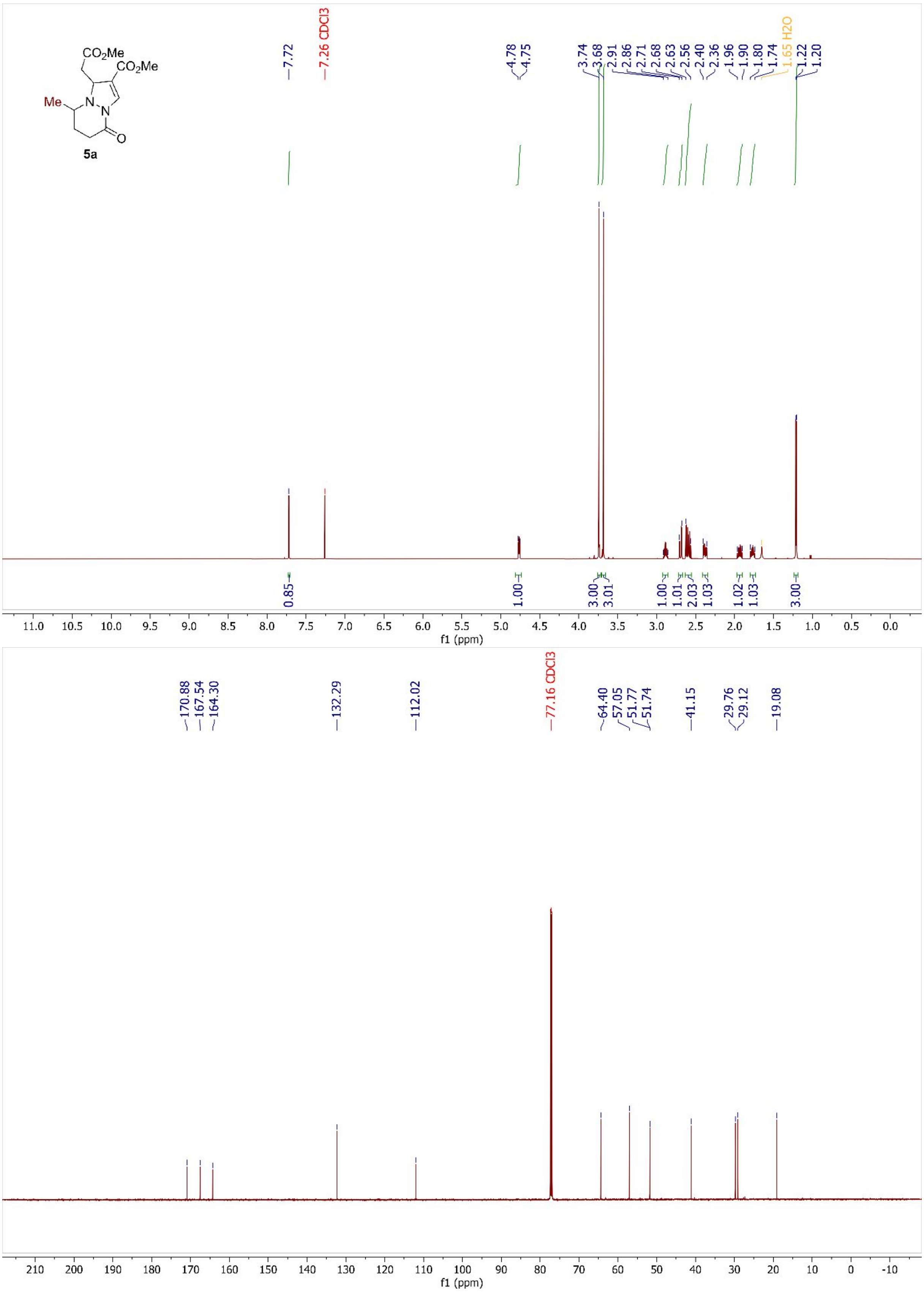

Figure S66. ^{1}H and $^{13}C\{1H\}$ NMR (600 MHz, $CDCl_3$) of compound **5a**.

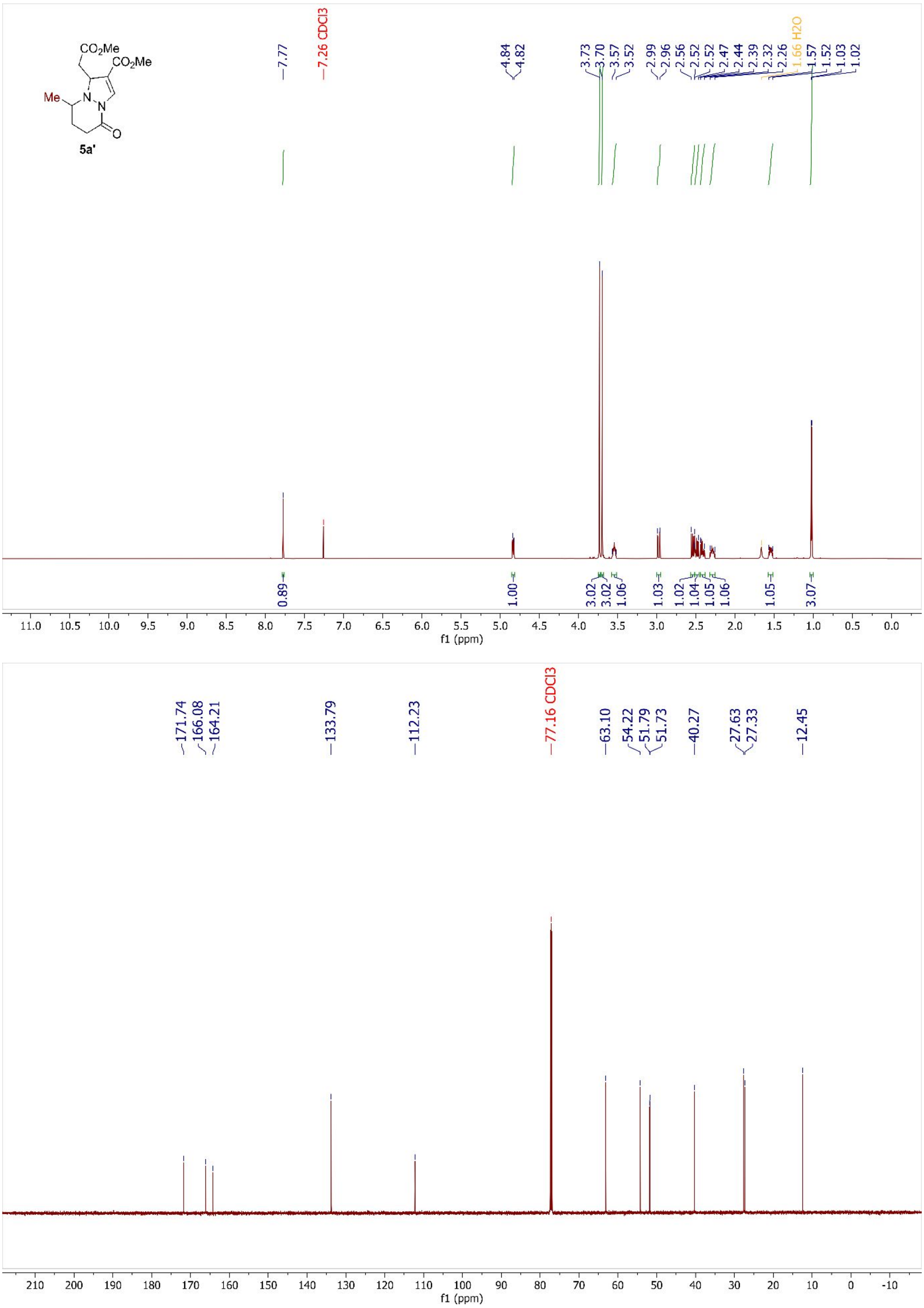

Figure S67. ^{1}H and ^{13}C{1H} NMR (600 MHz, $CDCl_3$) of compound **5a’**.

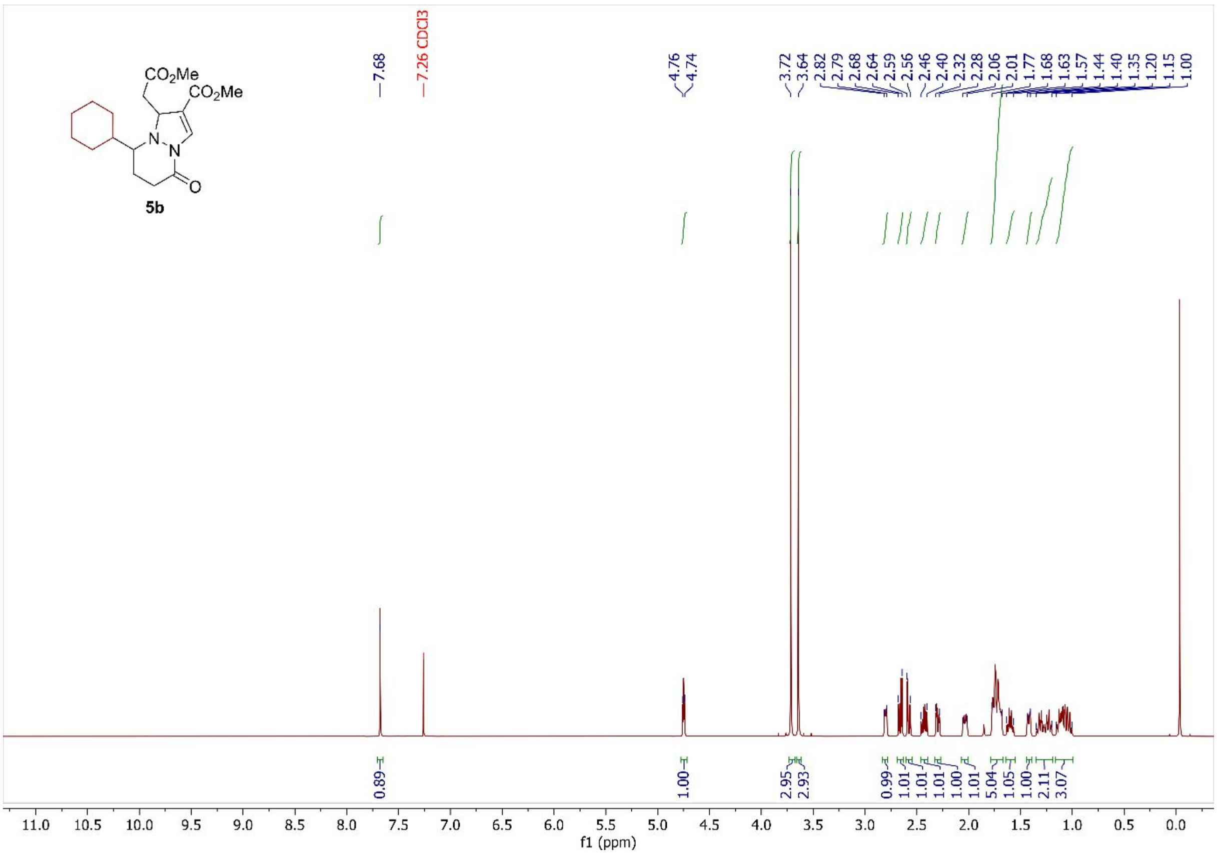

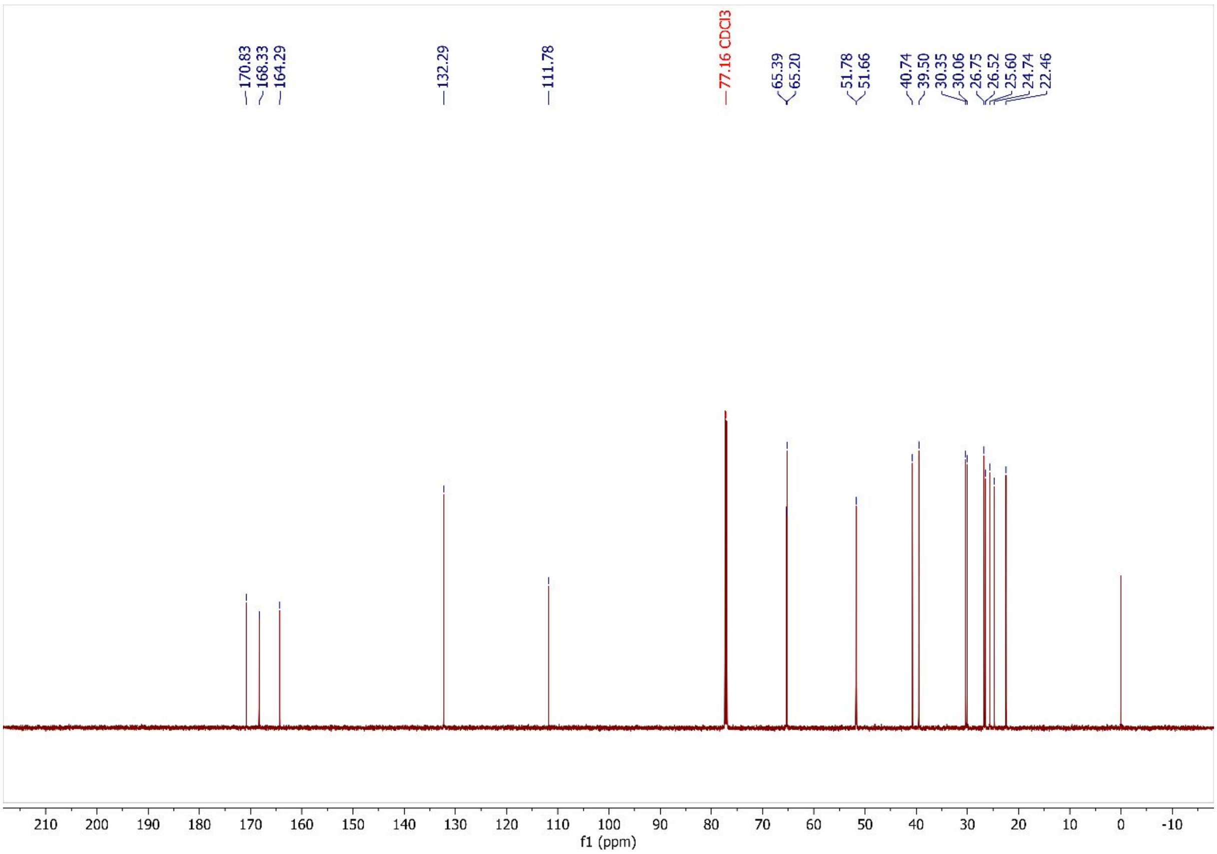

Figure S68. ^{1}H and $^{13}C\{1H\}$ NMR (600 MHz, $CDCl_3$) of compound **5b**.

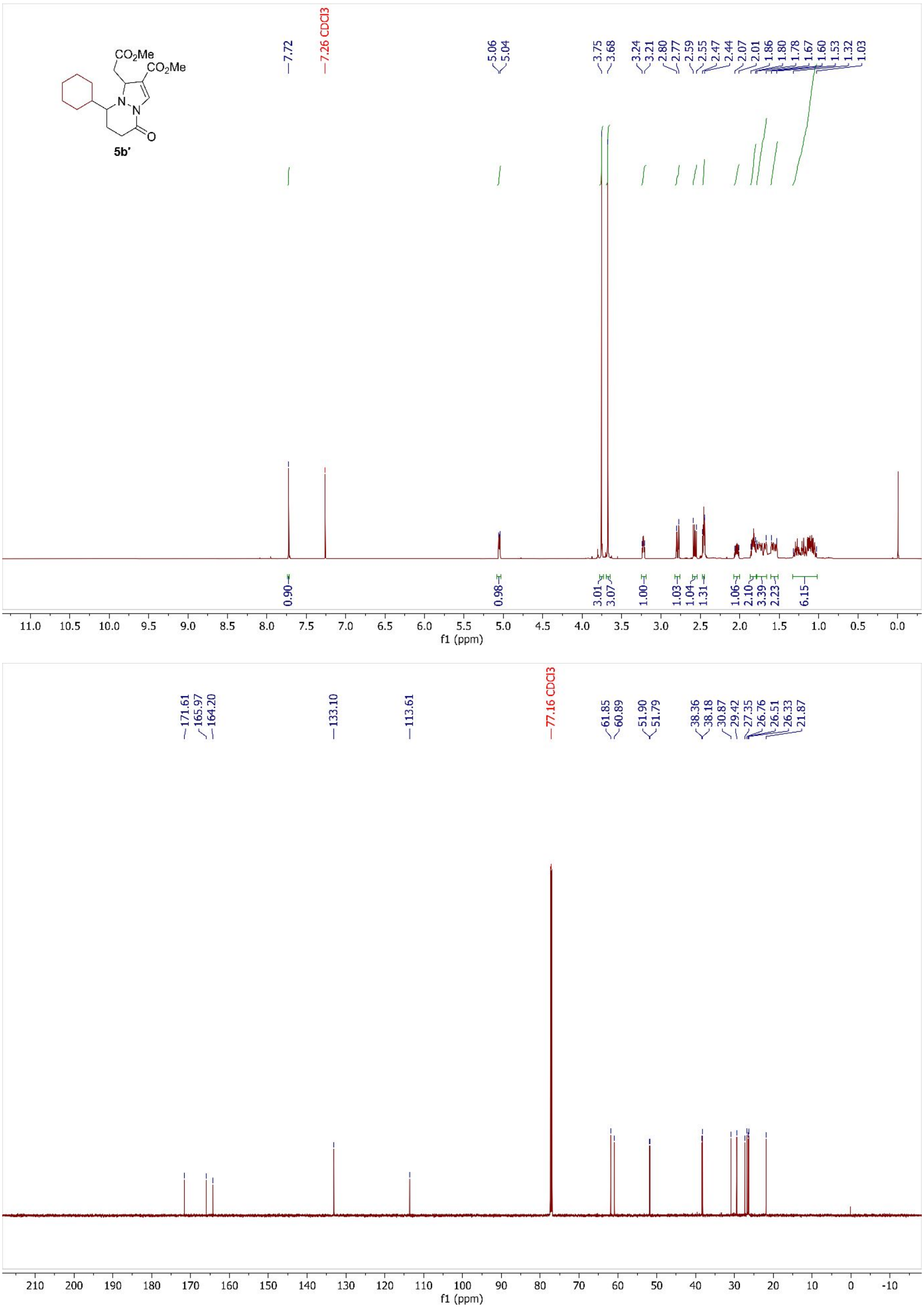

Figure S69. ^{1}H and $^{13}C\{1H\}$ NMR (600 MHz, $CDCl_3$) of compound **5b'**.

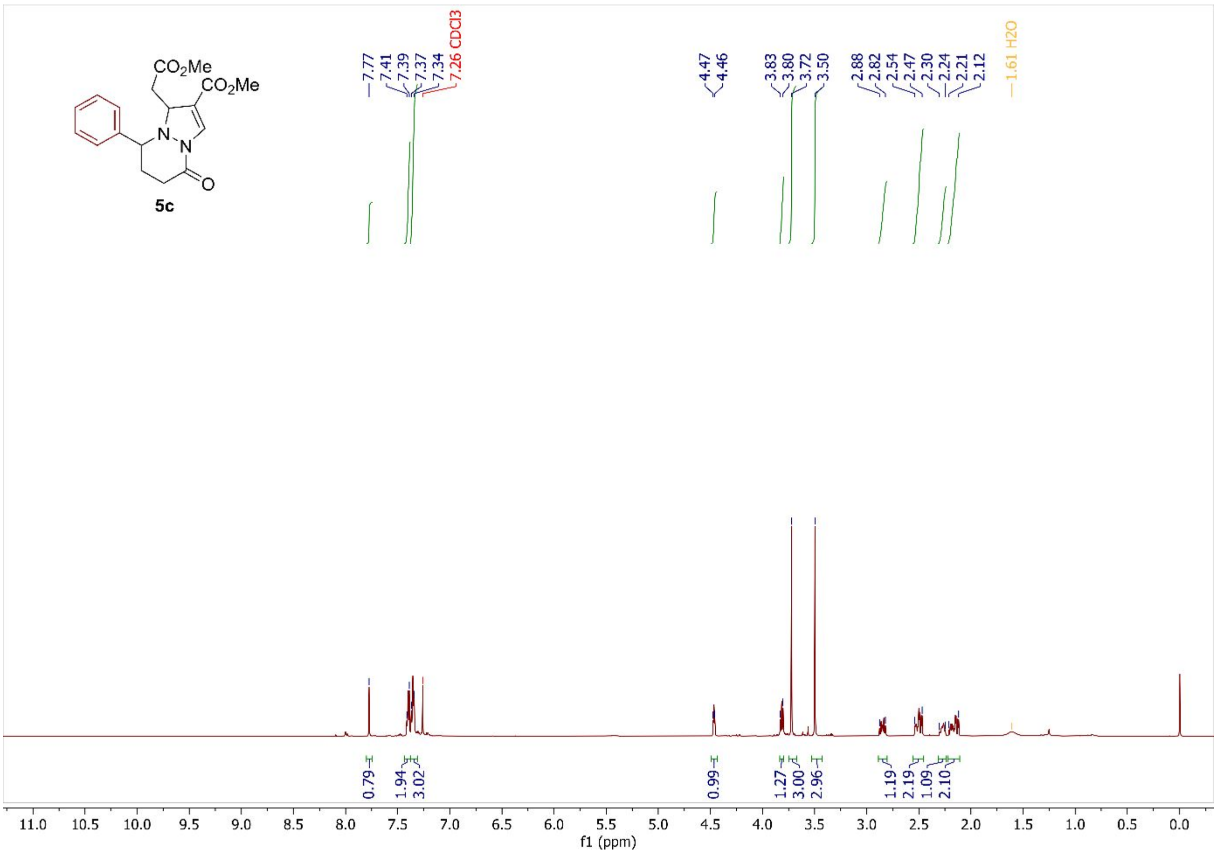

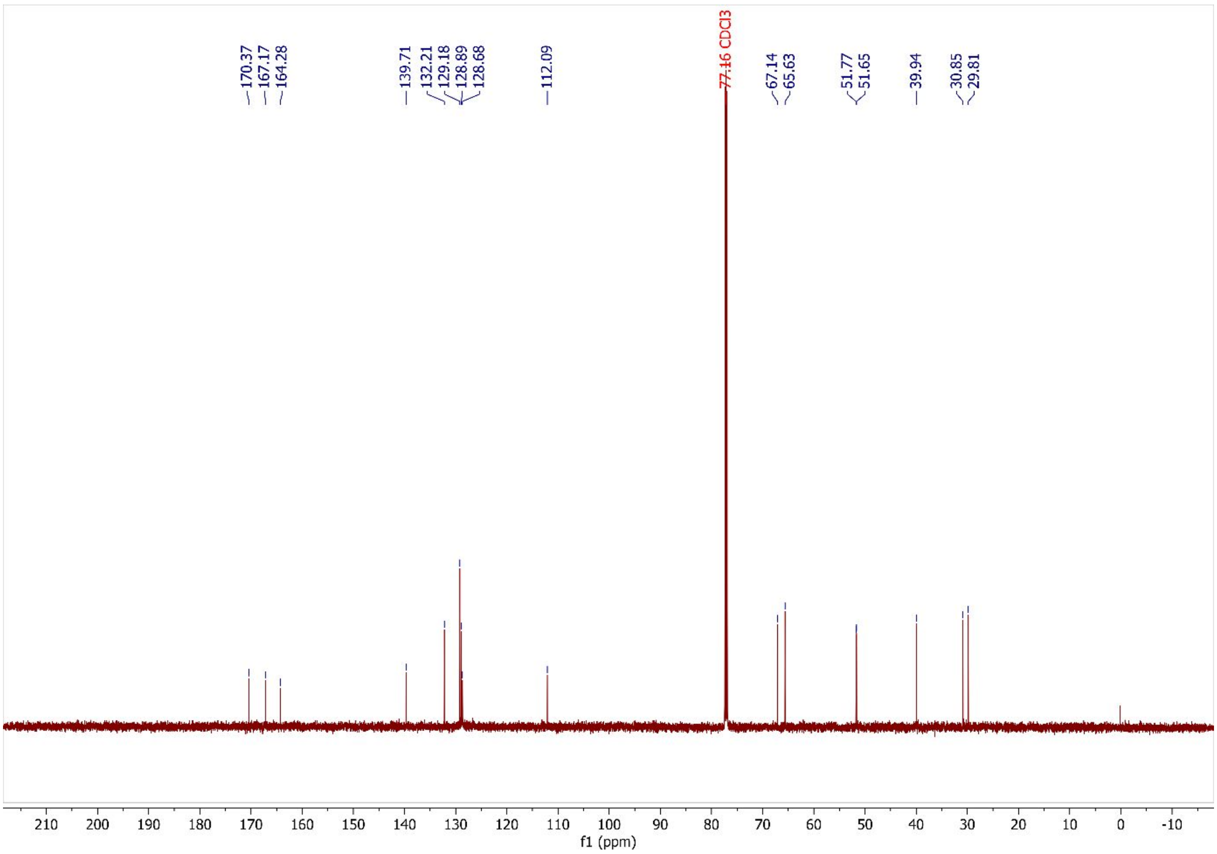

Figure S70. ^{1}H and $^{13}C\{1H\}$ NMR (600 MHz, $CDCl_3$) of compound **5c**.

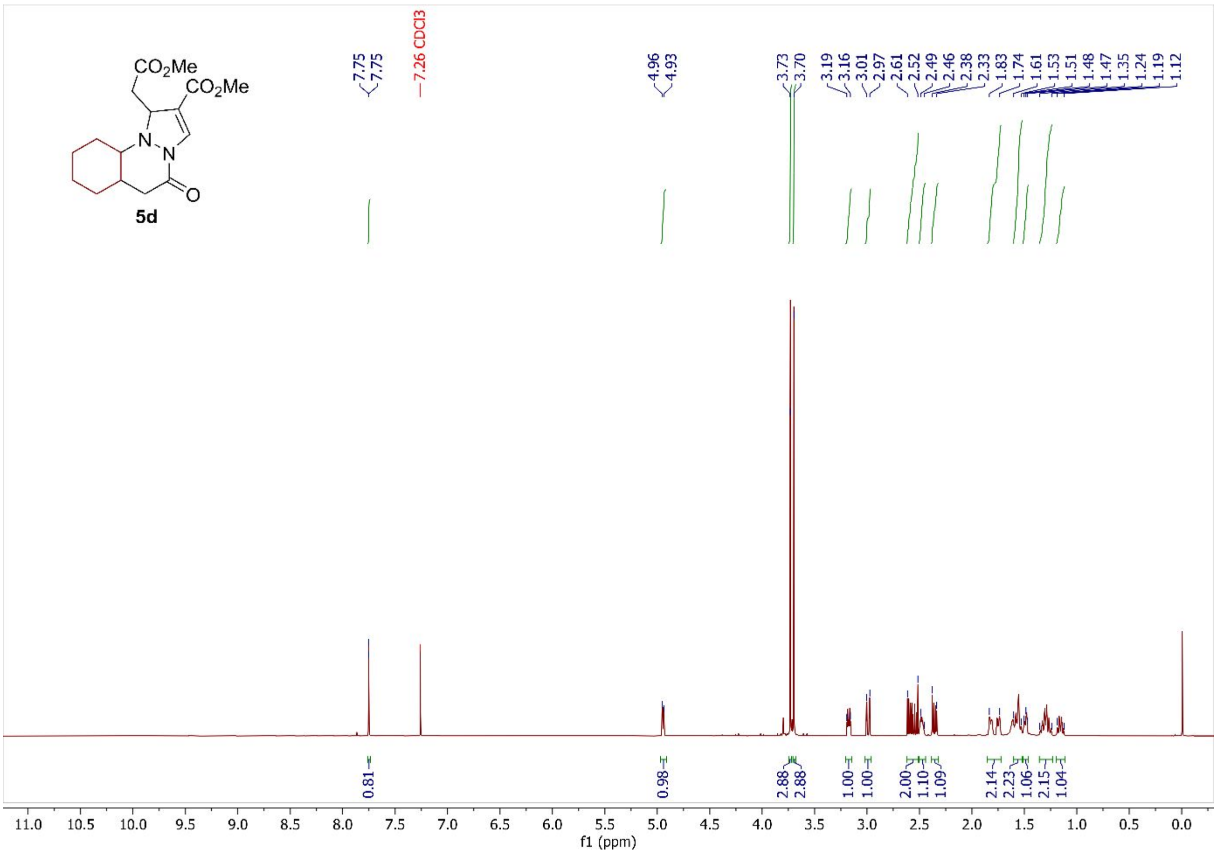

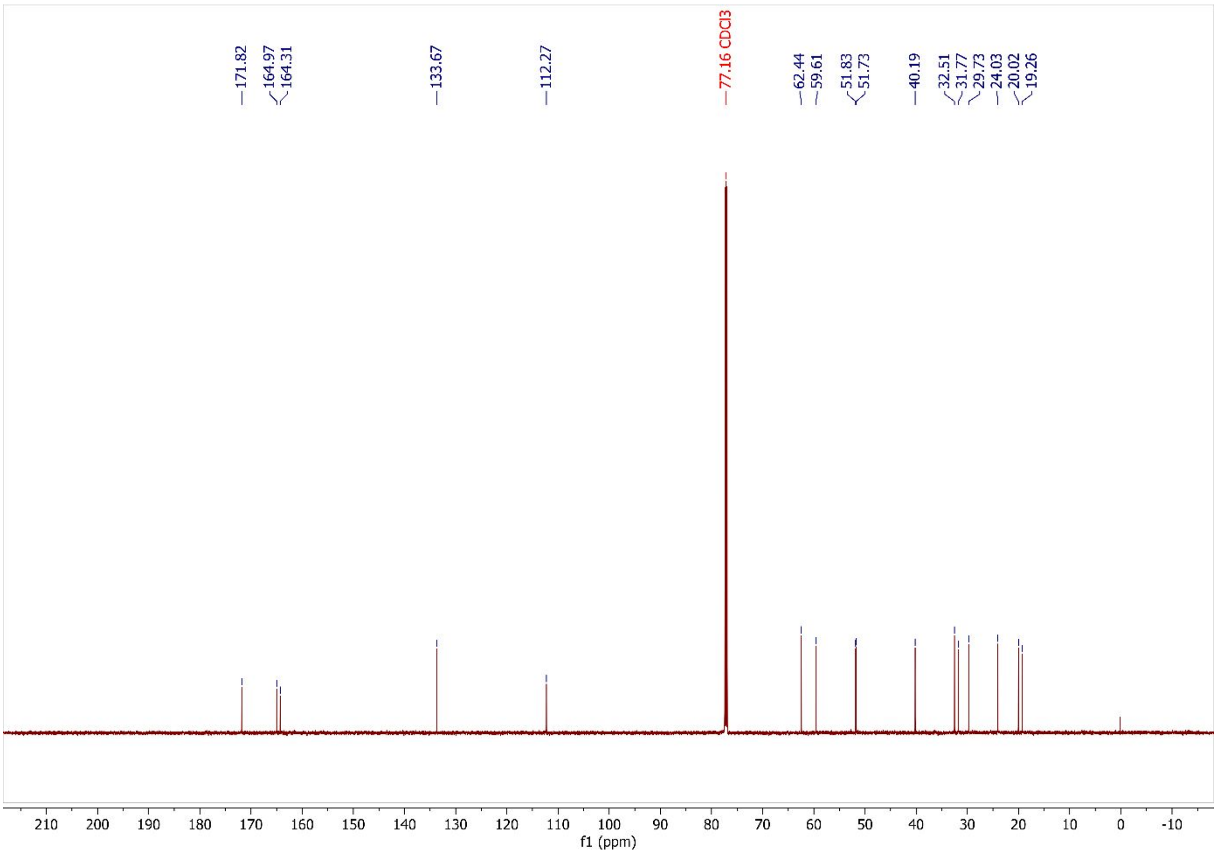

Figure S71. ^{1}H and $^{13}C\{1H\}$ NMR (600 MHz, $CDCl_3$) of compound **5d**.

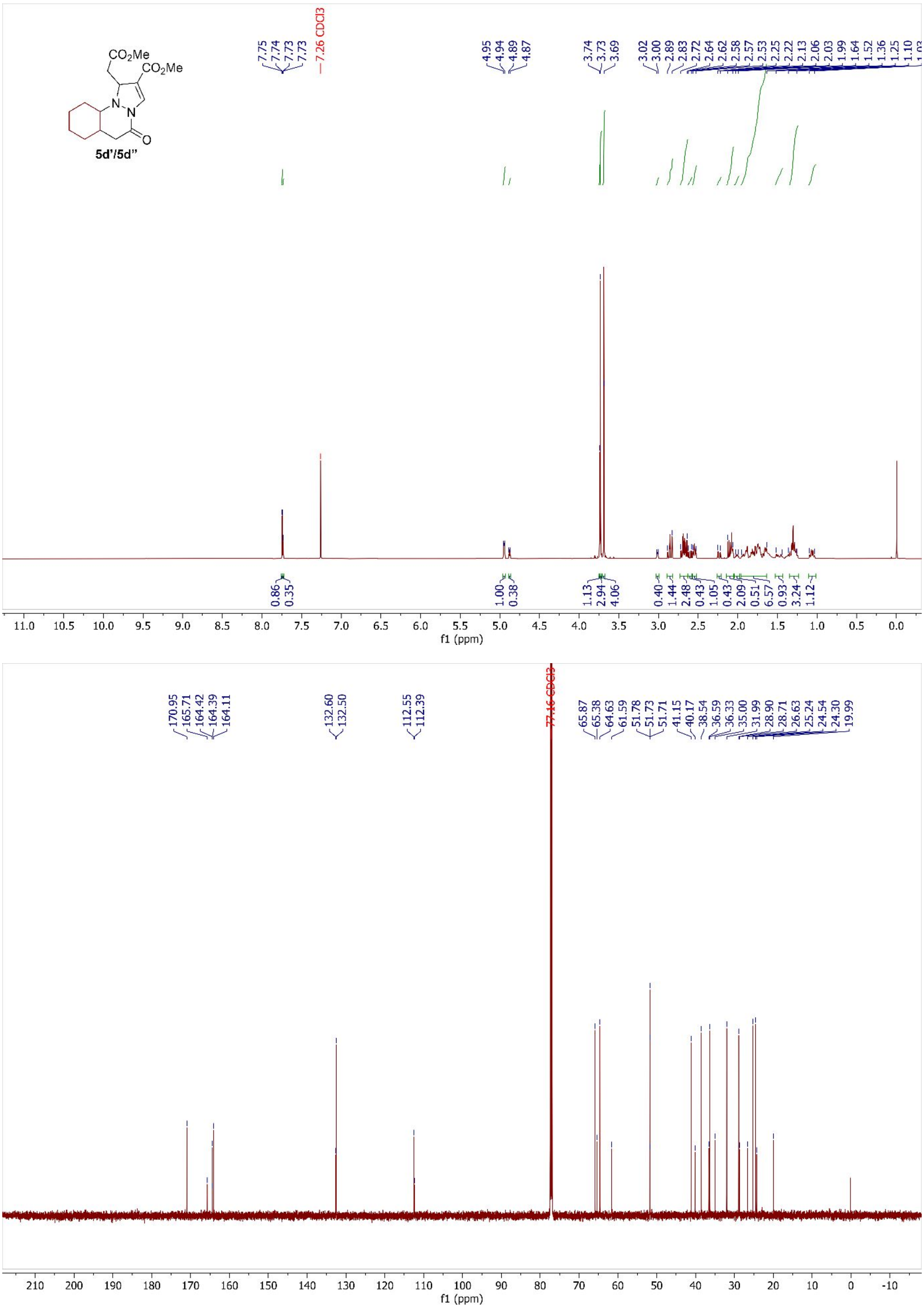

Figure S72. ^{1}H and $^{13}C\{1H\}$ NMR (600 MHz, $CDCl_3$) of compounds **5d/5d'.**

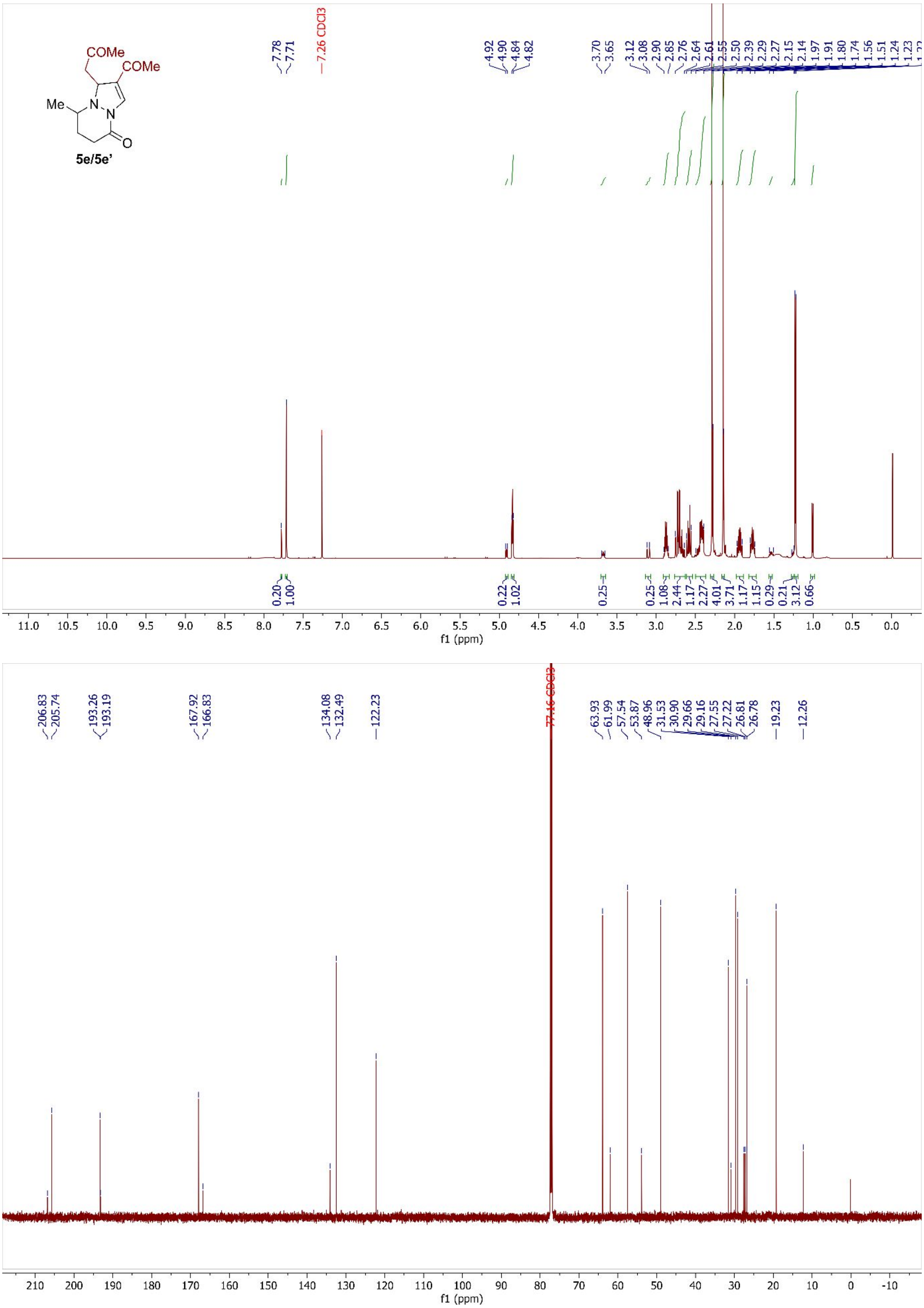

Figure S73. ^{1}H and $^{13}C\{1H\}$ NMR (600 MHz, $CDCl_3$) of compounds **5e/5e'.**

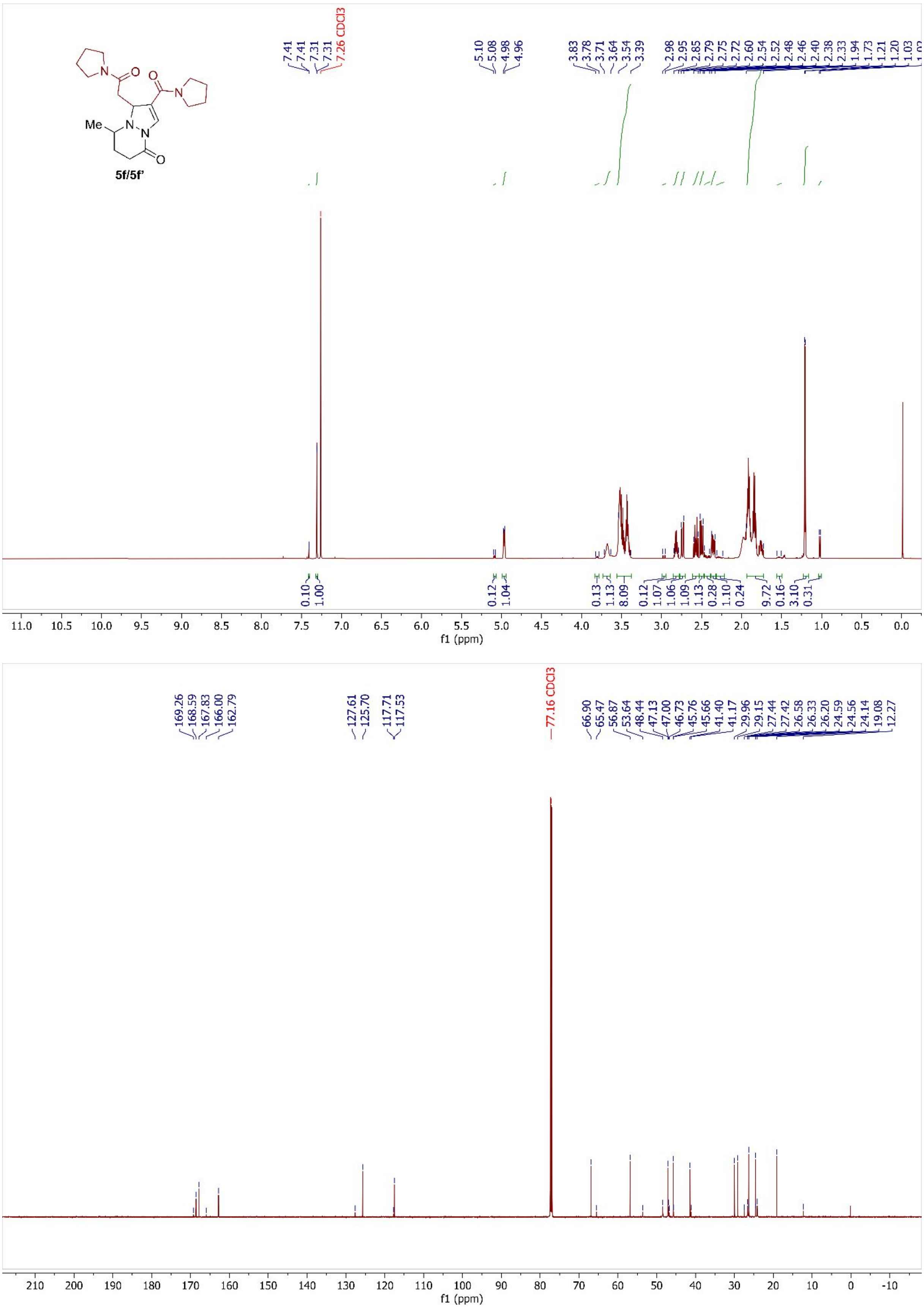

Figure S74. ^1H and $^{13}C\{1H\}$ NMR (600 MHz, $CDCl_3$) of compounds **5f/5f'**.

16.8 Intermediate **Int**

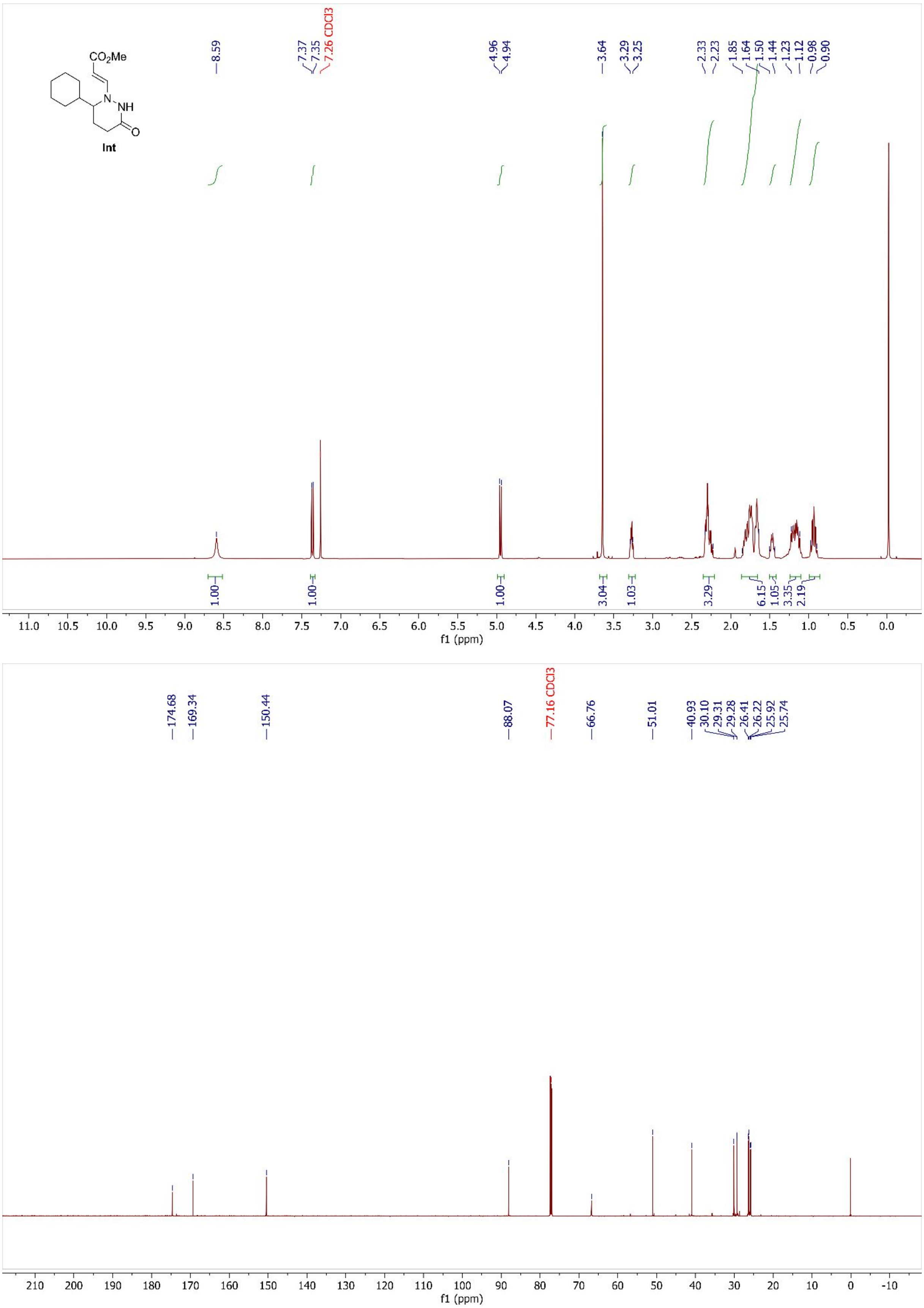

Figure S75. ^{1}H and ^{13}C{1H} NMR (600 MHz, $CDCl_3$) of compound **Int.**

17.9 Aldehydes **6**

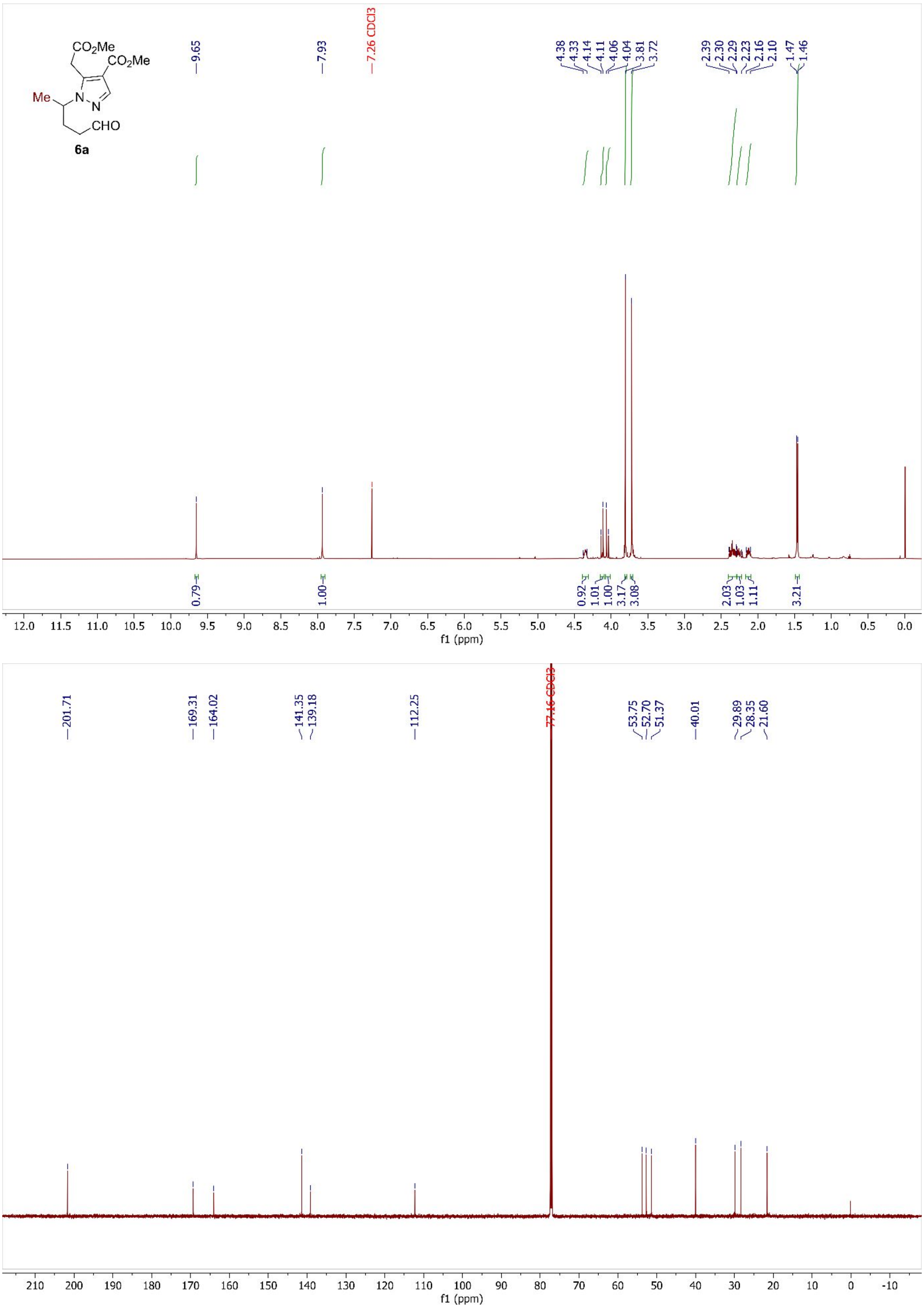

Figure S76. ^{1}H and $^{13}C\{1H\}$ NMR (600 MHz, $CDCl_3$) of compound **6a**.

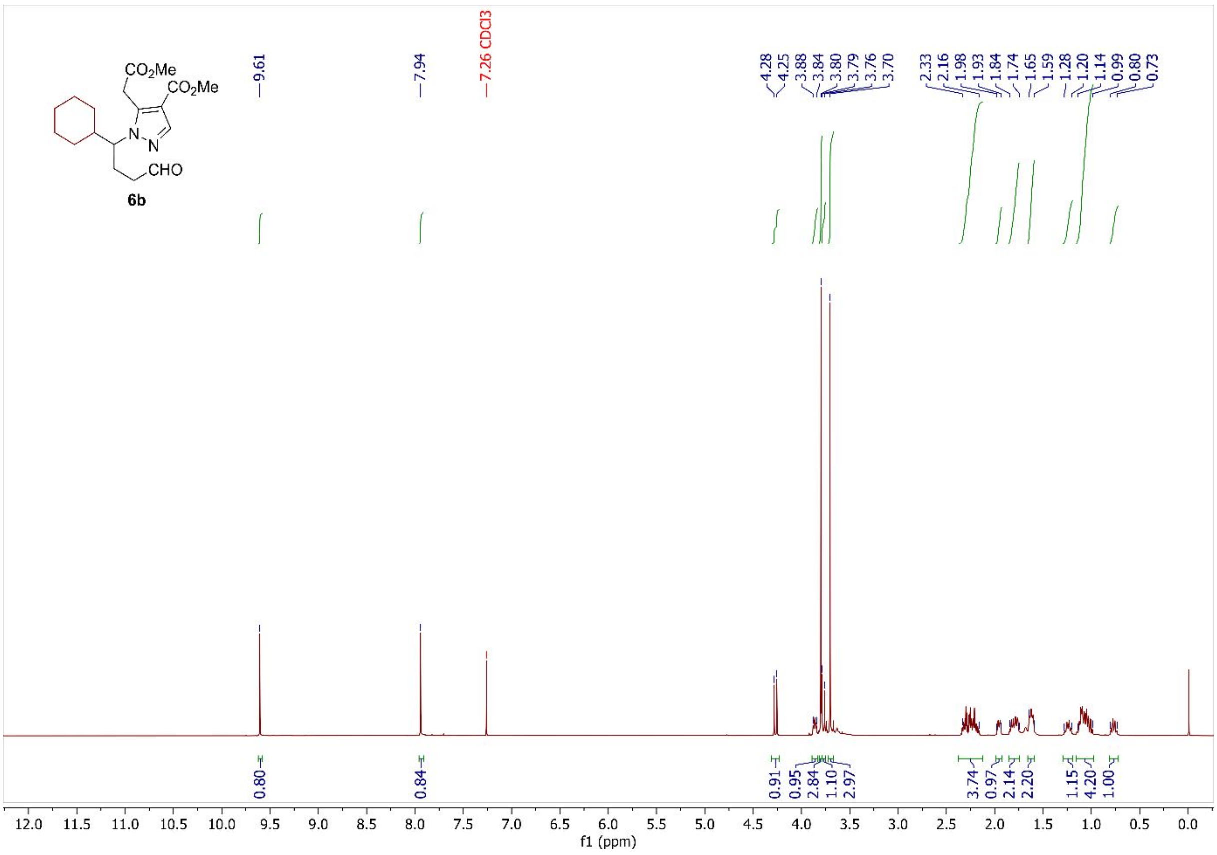

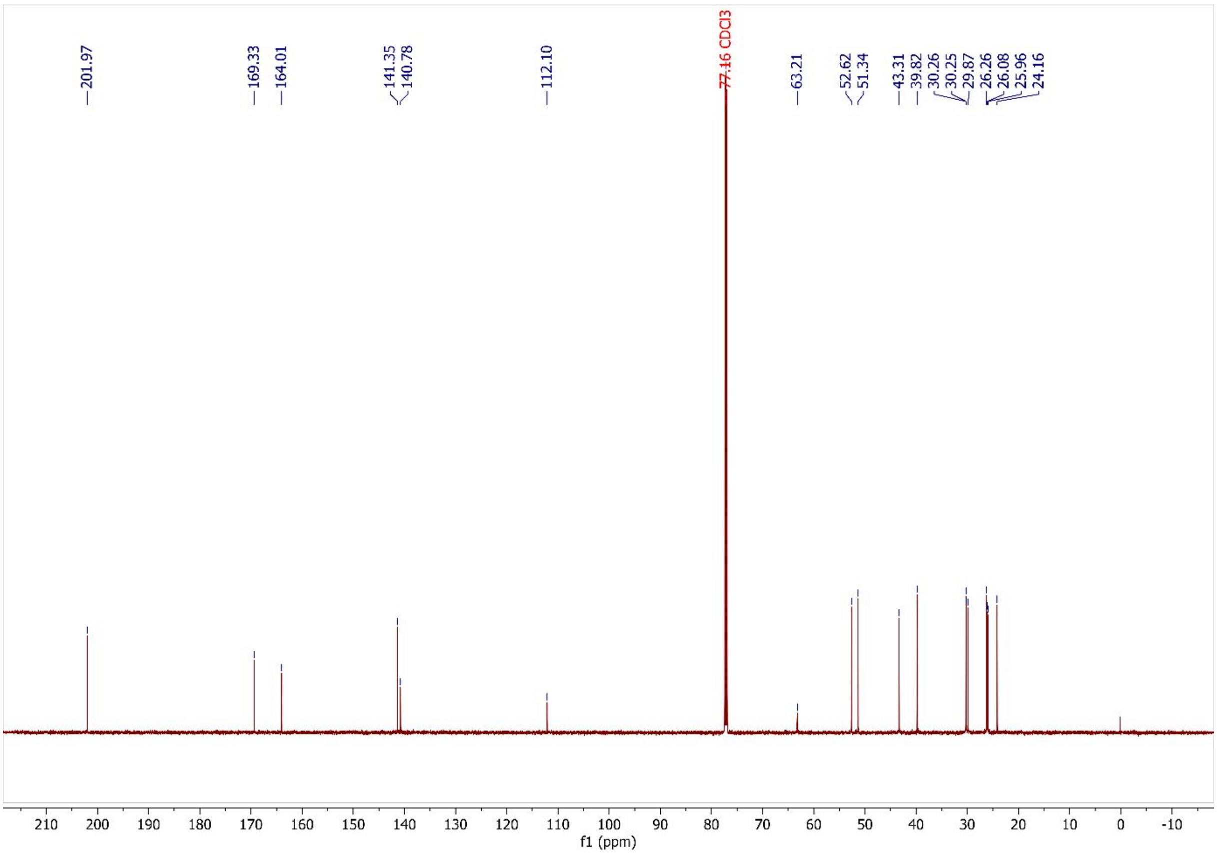

Figure S77. ^{1}H and $^{13}C\{1H\}$ NMR (600 MHz, $CDCl_3$) of compound **6b**.

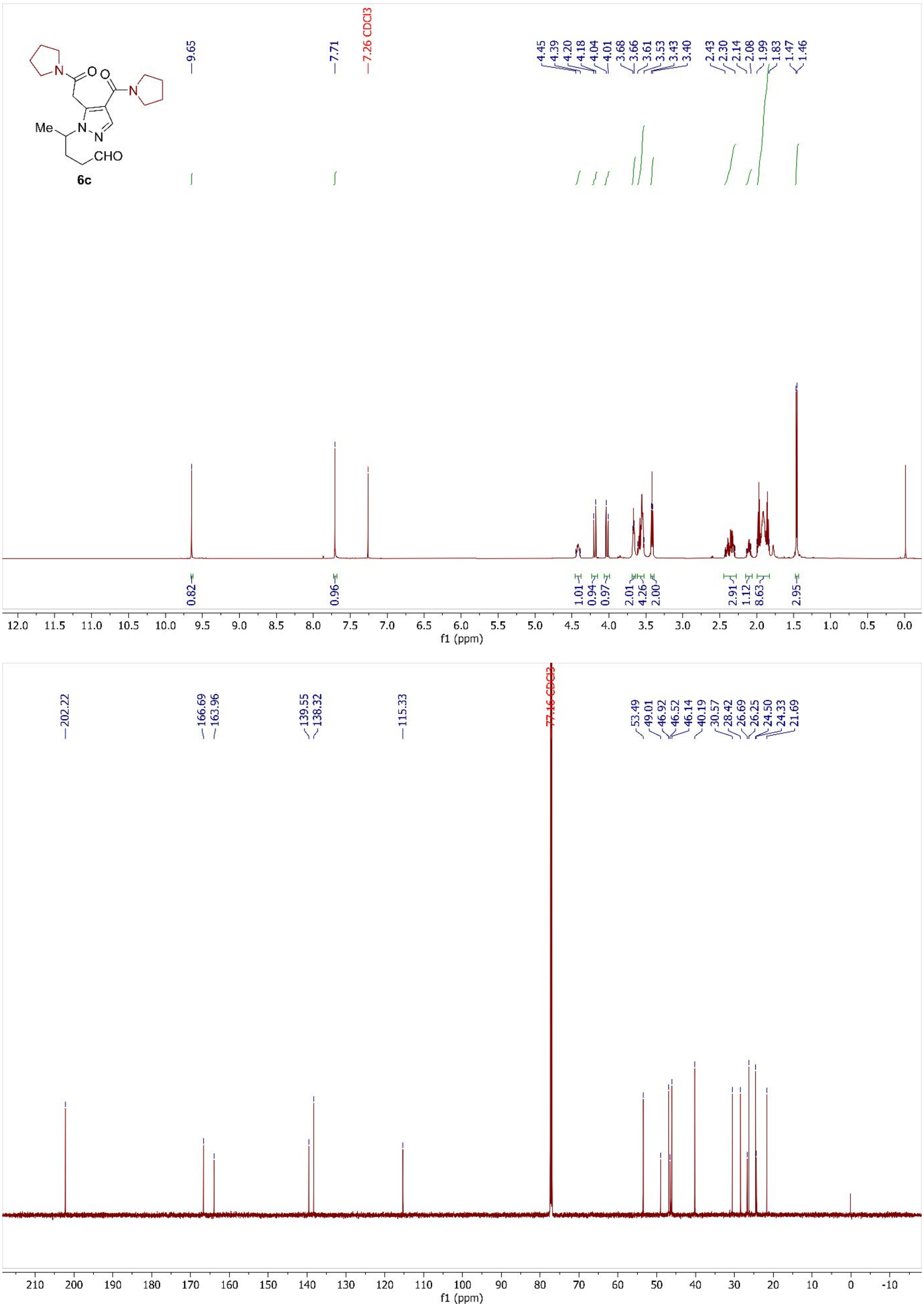

Figure S78. ^{1}H and ^{13}C{1H} NMR (600 MHz, $CDCl_3$) of compound **6c**.

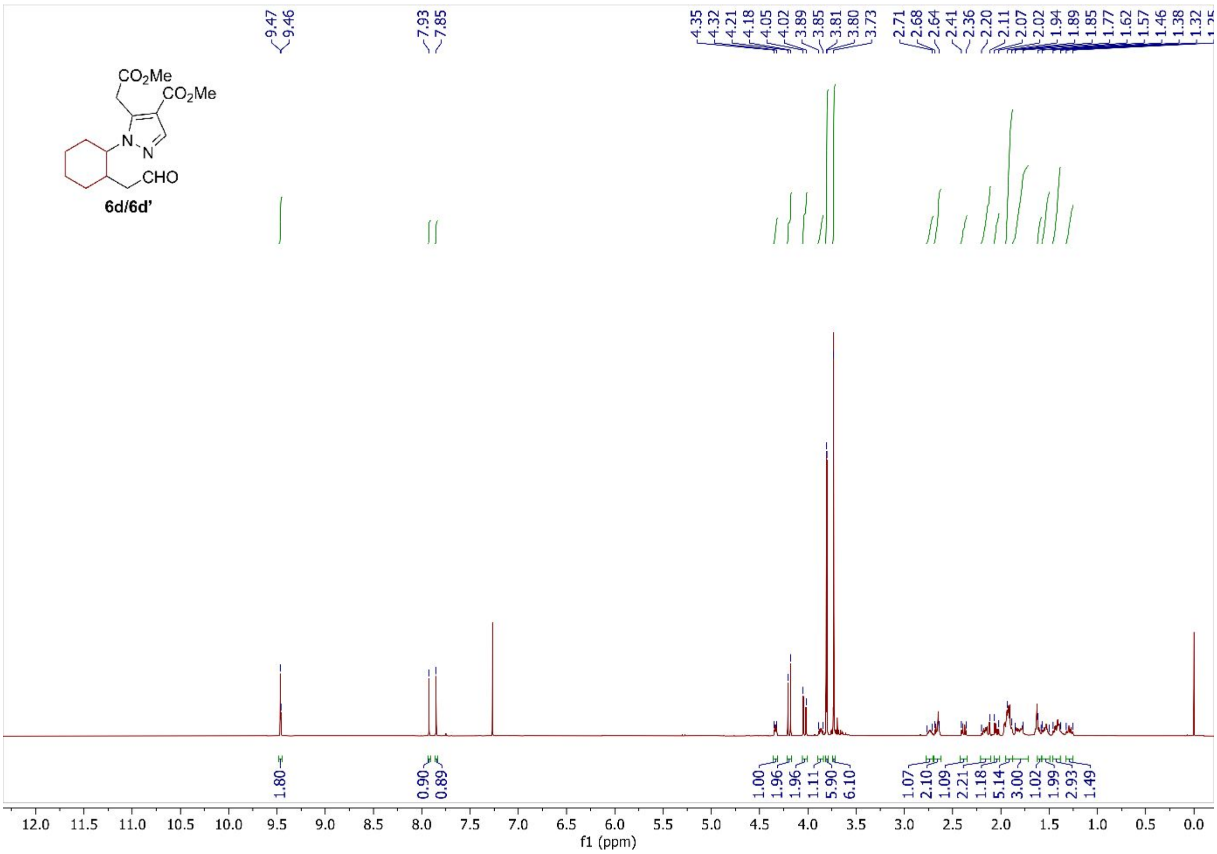

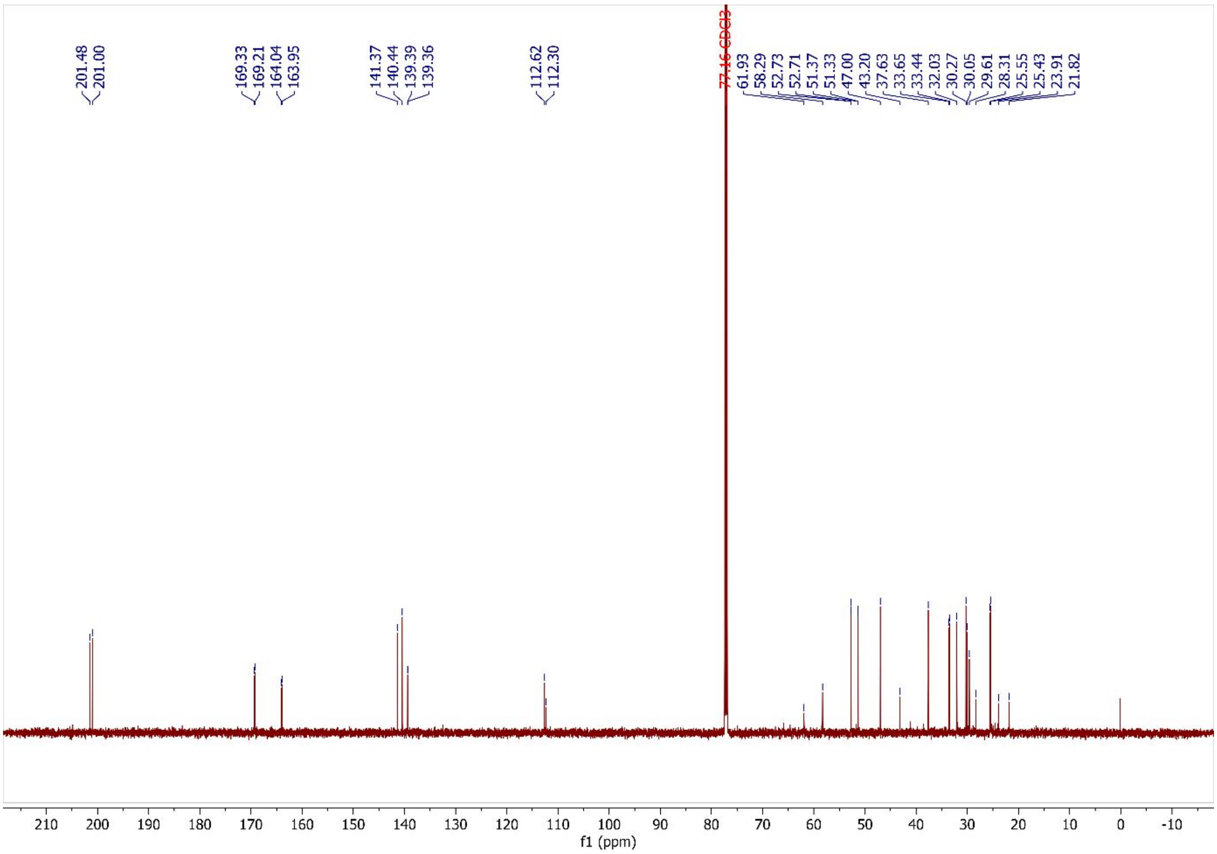

Figure S79. ^{1}H and $^{13}C\{1H\}$ NMR (600 MHz, $CDCl_3$) of compound **6d**/**6d'**.

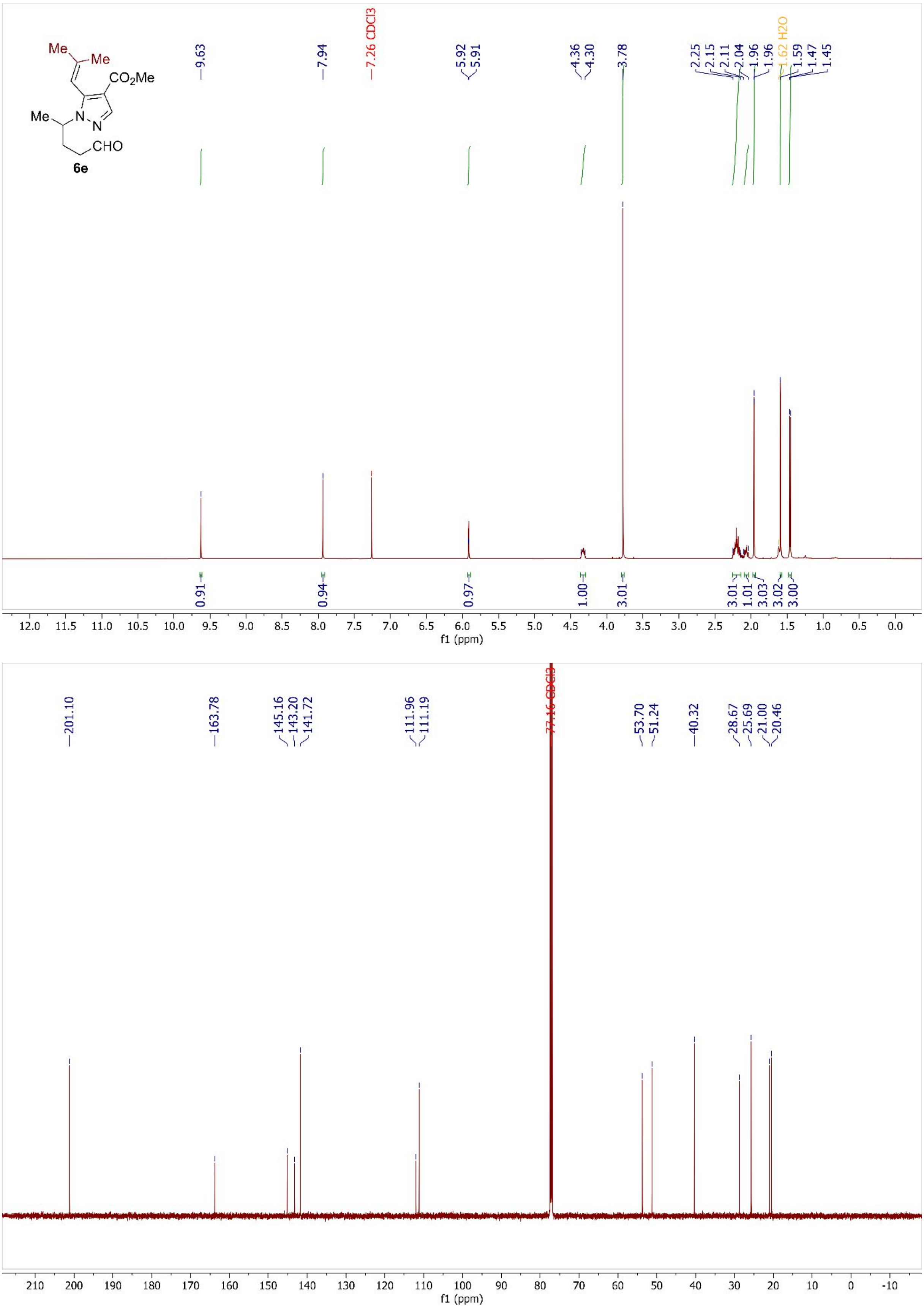

Figure S80. ^{1}H and $^{13}C\{1H\}$ NMR (500 MHz, $CDCl_3$) of compound **6e**.

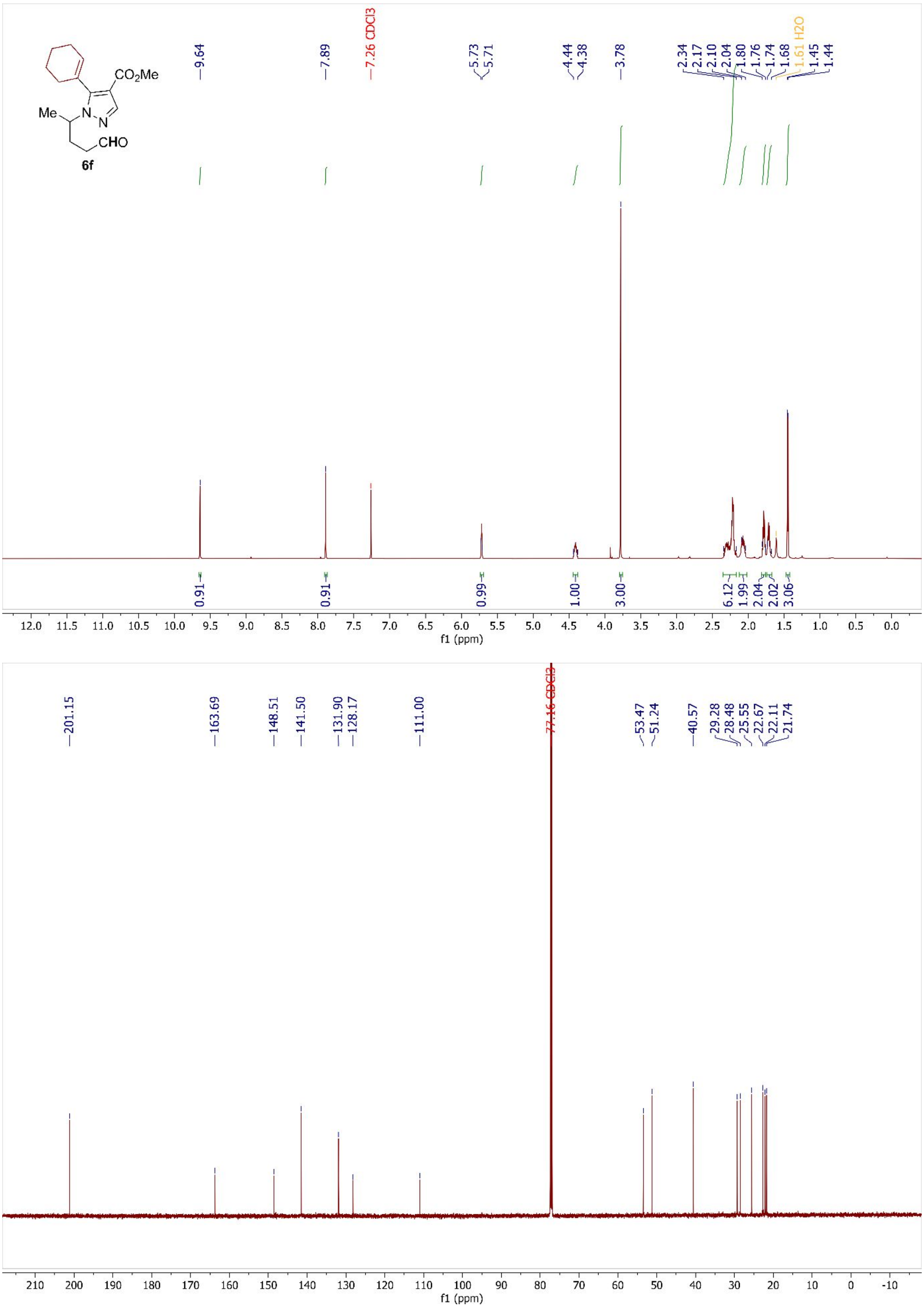

Figure S81. ^{1}H and $^{13}C\{1H\}$ NMR (600 MHz, $CDCl_3$) of compound **6f**.

16.10 Oxidized products **7**

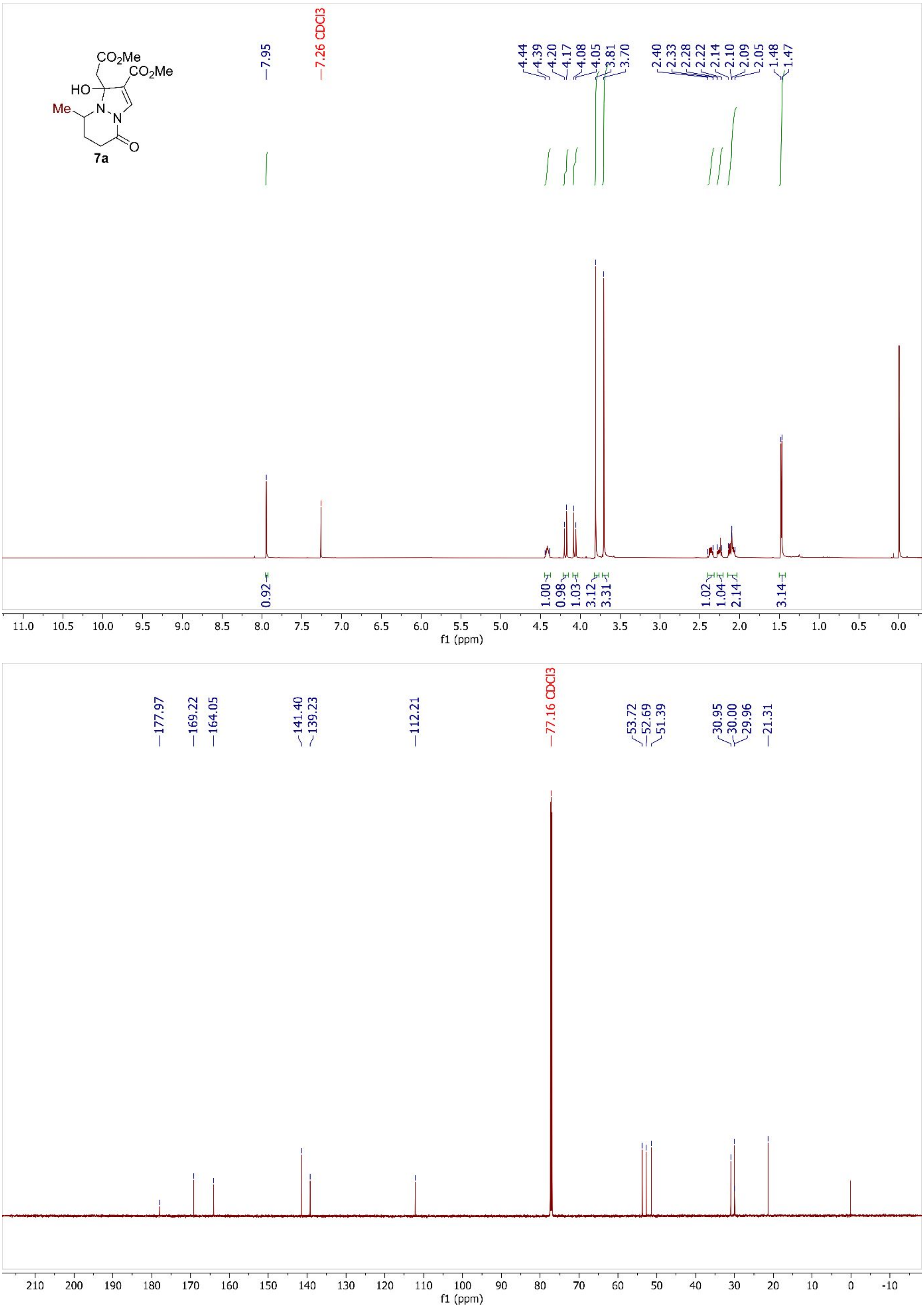

Figure S81. ^{1}H and $^{13}C\{1H\}$ NMR (600 MHz, $CDCl_3$) of compound **7a**.

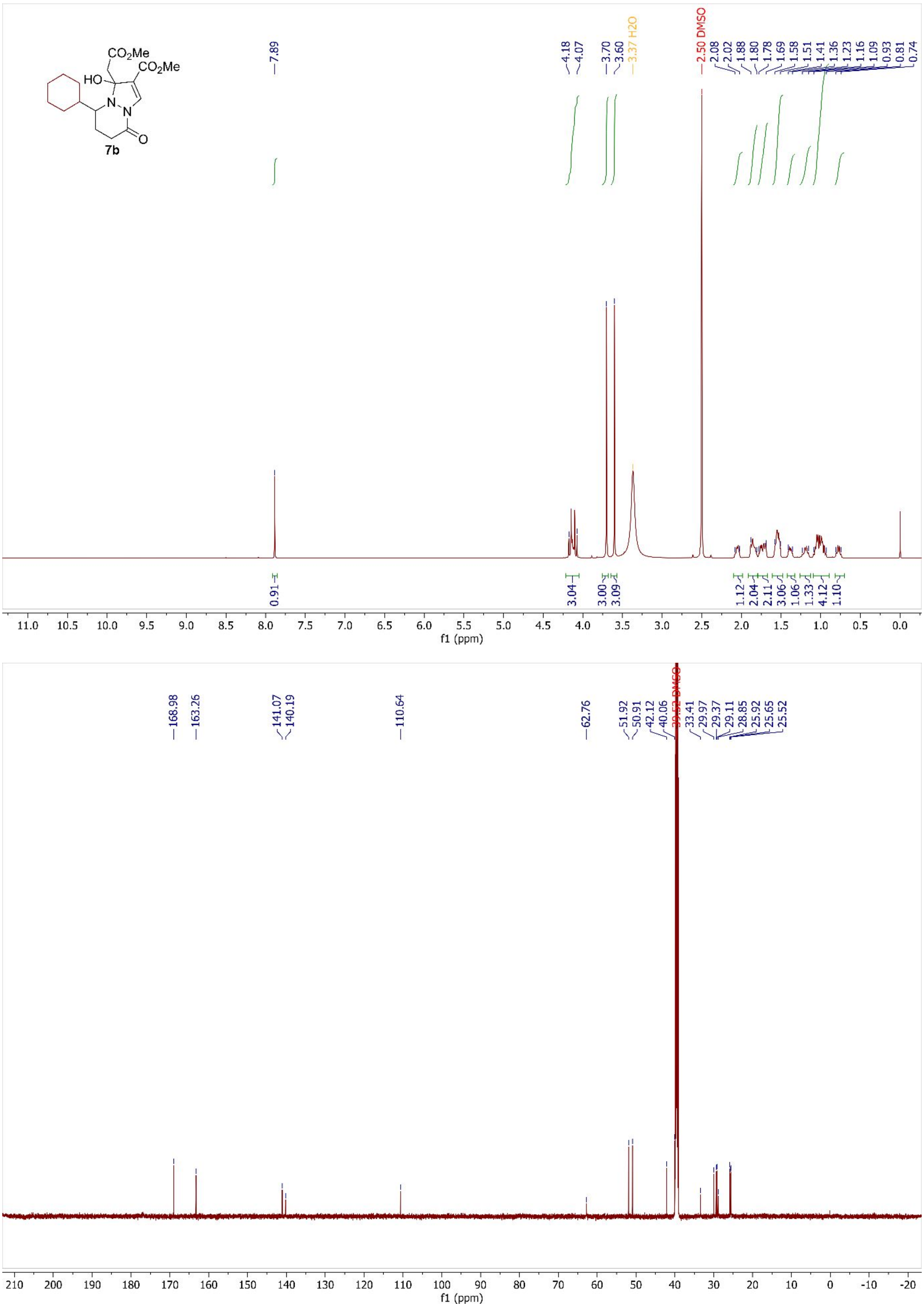

Figure S82. ^{1}H and $^{13}C\{1H\}$ NMR (600 MHz, DMSO-d_6) of compound **7b**.

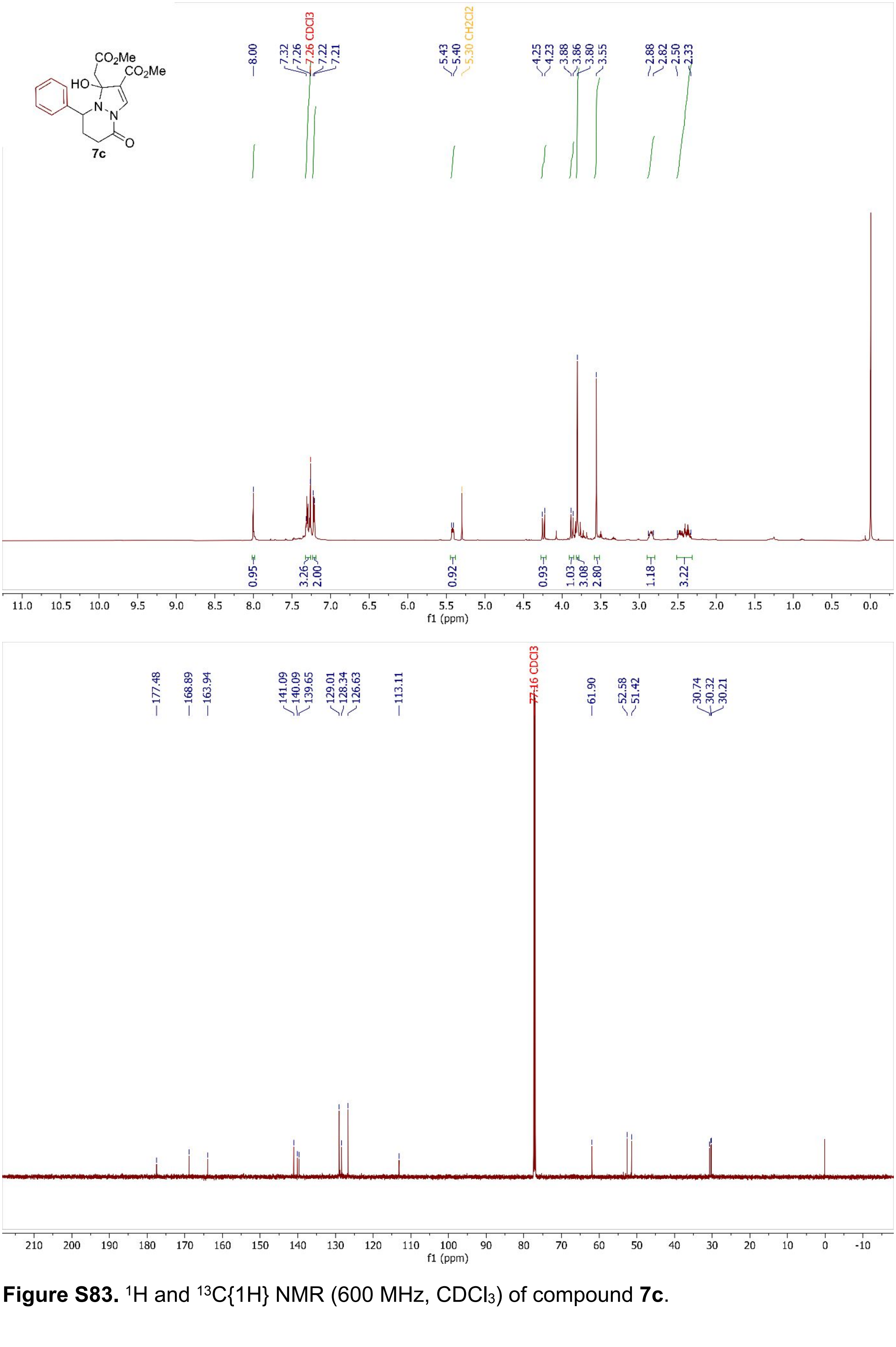

Figure S83. ^{1}H and ^{13}C{1H} NMR (600 MHz, $CDCl_3$) of compound **7c**.
